# Use of directed acyclic graphs (DAGs) to identify confounders in applied health research: review and recommendations

**DOI:** 10.1093/ije/dyaa213

**Published:** 2020-12-17

**Authors:** Peter W G Tennant, Eleanor J Murray, Kellyn F Arnold, Laurie Berrie, Matthew P Fox, Sarah C Gadd, Wendy J Harrison, Claire Keeble, Lynsie R Ranker, Johannes Textor, Georgia D Tomova, Mark S Gilthorpe, George T H Ellison

**Affiliations:** 1 Leeds Institute for Data Analytics, University of Leeds, Leeds, UK; 2 Faculty of Medicine and Health, University of Leeds, Leeds, UK; 3 Alan Turing Institute, British Library, London, UK; 4 Department of Epidemiology, School of Public Health, Boston University, Boston, MA, USA; 5 School of Geography, University of Leeds, Leeds, UK; 6 School of GeoSciences, University of Edinburgh, Edinburgh, UK; 7 Department of Global Health, Boston University, Boston, MA, USA; 8 Department of Tumour Immunology, Radboud University Medical Center, Nijmegen, The Netherlands; 9 Centre for Data Innovation, Faculty of Science and Technology, University of Central Lancashire, Preston, UK

**Keywords:** Directed acyclic graphs, graphical model theory, causal diagrams, causal inference, observational studies, confounding, covariate adjustment, reporting practices

## Abstract

**Background:**

Directed acyclic graphs (DAGs) are an increasingly popular approach for identifying confounding variables that require conditioning when estimating causal effects. This review examined the use of DAGs in applied health research to inform recommendations for improving their transparency and utility in future research.

**Methods:**

Original health research articles published during 1999–2017 mentioning ‘directed acyclic graphs’ (or similar) or citing DAGitty were identified from Scopus, Web of Science, Medline and Embase. Data were extracted on the reporting of: estimands, DAGs and adjustment sets, alongside the characteristics of each article’s largest DAG.

**Results:**

A total of 234 articles were identified that reported using DAGs. A fifth (*n* = 48, 21%) reported their target estimand(s) and half (*n* = 115, 48%) reported the adjustment set(s) implied by their DAG(s). Two-thirds of the articles (*n* = 144, 62%) made at least one DAG available. DAGs varied in size but averaged 12 nodes [interquartile range (IQR): 9–16, range: 3–28] and 29 arcs (IQR: 19–42, range: 3–99). The median saturation (i.e. percentage of total possible arcs) was 46% (IQR: 31–67, range: 12–100). 37% (*n* = 53) of the DAGs included unobserved variables, 17% (*n* = 25) included ‘super-nodes’ (i.e. nodes containing more than one variable) and 34% (*n* = 49) were visually arranged so that the constituent arcs flowed in the same direction (e.g. top-to-bottom).

**Conclusion:**

There is substantial variation in the use and reporting of DAGs in applied health research. Although this partly reflects their flexibility, it also highlights some potential areas for improvement. This review hence offers several recommendations to improve the reporting and use of DAGs in future research.

**Key Messages**

- Directed acyclic graphs (DAGs) are an increasingly popular approach for identifying confounding variables that require conditioning when estimating causal effects.
- We identified and reviewed 234 original health research articles from Scopus, Web of Science, Medline and Embase that were published during 1999–2017 and mentioned ‘graphical model theory’, ‘directed acyclic graph(s)’, ‘causal diagram(s)’, ‘causal graph(s)’, or ‘causal DAG(s)’ in their title, abstract or keywords, or cited the DAGitty software package.
- There was inconsistent reporting of several important technical details, such as the target estimand(s) of interest (reported by 21%), the DAG(s) (reported by 62%) and the DAG-implied adjustment set(s) (reported by 48%).
- Where DAGs were reported (62%), these varied substantially in size and structure: the average number of nodes was 12, the average number of arcs was 29, the median saturation (i.e. percentage of total possible arcs) was 46%; 37% included one or more unobserved variables; 17% included one or more ‘super-nodes’ (i.e. nodes containing more than one variable); and 34% were visually arranged so that the constituent arcs flowed in the same direction (e.g. top-to-bottom).
- We offer a list of eight simple recommendations to improve the transparency and utility of DAGs in future observational studies.

## Introduction

Estimating causal effects is a key aim of applied health research.^1^ One approach is to conduct a randomized controlled experiment, but practical and ethical constraints mean this is only possible for a limited range of exposures.^2^ Most causal effects must therefore be estimated from observational data; a notoriously difficult task that requires understanding, identifying and attempting to address the many sources of bias that arise in non-experimental data, including confounding bias, selection bias and information bias.^3^

Observational studies exploring causal effects are nevertheless extremely common in health and medical research, although their causal aims are rarely described explicitly.^4^ More often, observational studies adopt the language of ‘prediction’ or ‘association’, and they report ‘independent’ associations or ‘predictors’ after conditioning on one or more other related variables, typically by including them as covariates in a multivariable regression model. Many approaches are available to assist with deciding which variables to adjust for from a list of potential candidates, including various theory-free statistical criteria and algorithms.^5^ Unfortunately, few of these conventional approaches explicitly consider the role of each variable in relation to the exposure and outcome, and it is often unclear why some variables were chosen for consideration and others not. Without this information, many of the reported associations are uninterpretable, since estimating a specific causal effect requires conditioning on a specific set of variables that are determined by strong theoretical principles.^6^^,^^7^ This is exemplified by the ‘Table 2 fallacy’, which occurs when the coefficients for two or more ‘risk factors’ in a multivariable regression model are (mistakenly) interpreted as estimates for meaningful causal effects.^8^

Causal inference approaches, such as the potential outcomes framework, promote greater transparency by encouraging observational data scientists to formally define the causal effect(s) they seek (i.e. their causal ‘estimand’) before they begin their analysis.^9^ Estimating this effect is then informed by external knowledge of the data-generating process (i.e. how participants were identified and selected, how and when each variable was determined, and how each variable influenced each other), and by measuring and conditioning (whether directly or otherwise) on all mutual causes of the exposure and outcome (i.e. confounders).^10^^,^^11^ However, since the true data-generating process can never be known, it must be postulated from expert knowledge, relevant theory and plausible assumptions.^10^^,^^11^

Directed acyclic graphs (DAGs) provide a simple and transparent way for observational data scientists to identify and demonstrate their knowledge, theories and assumptions about the causal relationships between variables.^12^ The implied adjustment set for accurately estimating a causal effect can then be deduced by inspection or algorithmically, depending on the DAG’s structure and complexity.^13–15^ Although the accuracy of the resulting estimate is contingent on how closely the DAG matches the (true) data-generating process, the act of drawing and sharing a DAG makes these assumptions more explicit and open to scrutiny. Despite these benefits, DAGs are relatively rare within the wider setting of applied health research, likely due to a lack of awareness and cultural ambivalence.^16^^,^^17^

There is also limited practical guidance available on the use and reporting of DAGs in applied research. Sauer and VanderWeele have offered a list of core considerations and highlighted the need for a ‘disciplined approach to developing DAGs’.^18^ In response, Ferguson *et al*. offered a structured protocol to aid with building DAGs,^19^ but there remains little advice on various practical considerations, including the reporting of: estimands of interest, DAGs themselves, implied and analytical adjustment set(s), the spatial arrangement of variables, and the justification for including or omitting dependencies.

This review aims to examine and evaluate the use of DAGs in applied health research to motivate some simple recommendations to improve the transparency and utility of DAGs in future observational research.

## Methods

### Overview

The review sought to extract, examine and summarise information on the use, implementation and reporting of causal diagrams that satisfy the definition of a DAG, as provided below, in observational health research. We were primarily interested in reporting behaviours and technical features of DAG specification that could motivate subsequent recommendations. These included reporting of: estimands of interest, DAGs, implied adjustment sets, other adjustment set(s) and the estimates obtained from these adjustment sets. They also included: the size of each DAG, the inclusion of unobserved variables, the use of ‘super-nodes’ (i.e. nodes containing more than one variable), the assumptions and justifications for including or excluding causal relationships, and whether causal relationships were visually arranged in the same direction (e.g. top-to-bottom or left-to-right).

### Definitions

DAG**s** are non-parametric diagrammatic representations of the assumed data-generating process for a set of variables (and measurements thereof) in a specified context. Variables and their measurements are depicted as nodes (or vertices) connected by unidirectional arcs (or arrows; hence ‘directed’) depicting the hypothesized relationships between them. An arc between two nodes denotes the assumed existence and direction of a causal relationship, but it does not specify the sign (i.e. positive or negative), magnitude (i.e. large or small), shape (e.g. linear or non-linear) or form of that relationship (hence ‘non-parametric’). A node cannot be caused by itself (hence ‘acyclic’), because no variable can cause itself at an instantaneous moment in time, and the future cannot cause the past. A saturated DAG is a DAG that contains all possible arcs, such that each node in turn causes all future nodes.^20^

**Figure 1:**
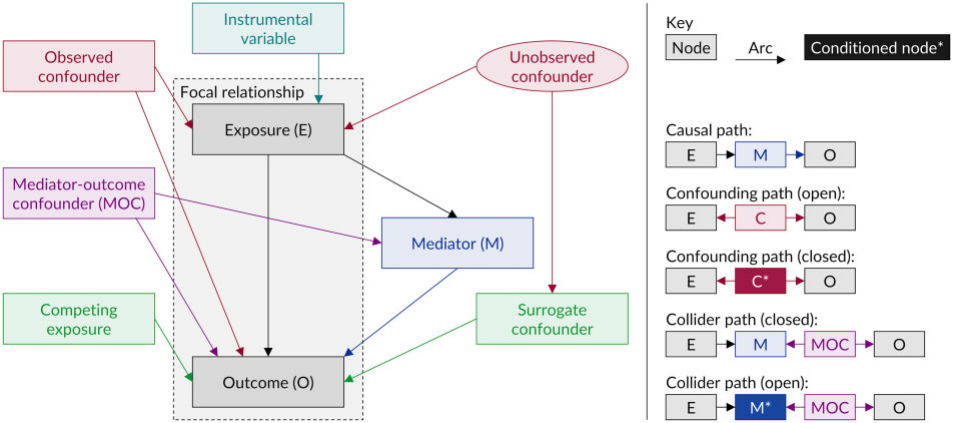
Illustration of the main components of a DAG, the most common types of contextual variables and the most common types of paths. The DAG has been visually arranged so that all constituent arcs flow from top-to-bottom.

A path is a collection of one or more arcs that connects two nodes. Paths may be either open or closed; open paths transmit statistical associations, closed paths do not. A causal path is one where all constituent arcs flow in the same direction from one node to another. The total causal effect of a specified exposure (i.e. cause) on a specified outcome (i.e. consequence), which together form the focal relationship, is the joint effect transmitted through all causal paths connecting the exposure to the outcome. With respect to the focal relationship, a confounder is a common cause of both the exposure and the outcome, a mediator is caused by the exposure and in turn causes the outcome (i.e. falls on a causal path between the exposure and outcome), and a competing exposure is a cause of the outcome that is neither caused by nor causes the exposure. A direct causal effect is the effect that does not act through one or more specified mediators. Figure 1 shows the main components of a DAG and the most common types of variable, defined in relation to the focal relationship.

The (causal) estimand is the desired causal effect of the exposure on the outcome (e.g. the total causal effect). A confounding path is an open path between the exposure and outcome that passes through one or more confounders (Figure 1). These paths introduce confounding bias, which may be reduced by conditioning on one or more of the nodes on that path such that it becomes closed; this is typically achieved by including those nodes as covariates in a multivariable regression model. A collider path is a closed path between the exposure and outcome that passes through one or more colliders, which are nodes that receive two or more arcs (Figure 1); the simplest example occurs when the exposure and outcome both cause another variable (i.e. the collider) directly. Collider paths do not transmit statistical associations unless the constituent colliders, or one of their descendants, have been conditioned on. Such conditioning can introduce collider bias. For example, conditioning on a mediator that is also caused by one or more common causes of the outcome (known as mediator-outcome confounders) is likely to introduce collider bias, since the mediator in this situation is also a collider on the path between the exposure and the outcome.^12^

A sufficient adjustment set for a particular estimand is any set of variables that, if fully conditioned on, will provide an unbiased estimate for that estimand by closing all paths that are not causal and leaving all causal paths open.^21^ Competing exposures are not always included in sufficient adjustment sets, unless they are caused by one or more unobserved confounders for which they can serve as a surrogate confounder.^22^

### Search and inclusion criteria

We searched Scopus, Web of Science, Medline and Embase for articles published between 1 January 1999 and 31 December 2017 inclusive that contained any of the following terms in their title, abstract, keywords or topic: ‘graphical model theory’, ‘directed acyclic graph(s)’, ‘causal diagram(s)’, ‘causal graph(s)’ or causal DAG’. The starting year was chosen to capture studies that appeared around or after the publication of Greenland *et al.* (1999).^12^ Articles citing DAGitty online or the DAGitty R software packages were also identified.^15^^,^^21^ Results were restricted to ‘original articles’ involving human participants (Medline and Embase) indexed within medicine, health, social science, psychology, dentistry, nursing and related fields.

### Screening and exclusion criteria

Duplicates were identified and removed before P.W.G.T. screened the titles and abstracts to determine eligibility. Articles not describing original research (such as teaching articles, review articles and commentaries) were excluded, as were articles not examining health or healthcare in human populations (such as gene ontology and protein hierarchy studies).

### Extraction process

Data were extracted from each article into a standardized database, which was designed, tested, refined, piloted, and further refined before extraction. P.W.G.T., K.F.A., M.S.G., W.J.H., L.B., S.C.G., C.K. and G.T.H.E. were each assigned a random sample of articles for data extraction. Where information was unclear, individual members of the study team consulted with one or more other members to reach consensus on the appropriate coding. All data were then re-extracted and double-checked by P.W.G.T., G.D.T. and G.T.H.E. Discordances between the original data extraction and the subsequent re-extraction were recorded and considered preliminary ‘errors’ in data extraction.

### Data of interest

Bibliographic information including author names, journal names and year of publication were obtained for each article. The topic of each article was approximated from the indexing categories of the host journal in the 2017 Journal Citation Reports from Clarivate Analytics (https://jcr.clarivate.com/).

Data were extracted on: the country of the lead author’s primary affiliation; the number of DAGs used and their availability within the manuscript/supplementary materials; and the analytical approach used (noting where mediation analyses or other less conventional methods were used). For the largest DAG available for each article (assessed by number of nodes; or number of arcs, if tied), we extracted data on the total number of nodes and total number of arcs, and we recorded whether: it was drawn with DAGitty, it contained any unobserved variables or ‘super-nodes’,^23^ it was saturated, the arcs were visually arranged to flow in the same direction (e.g. left-to-right), and citations were used to justify the inclusion or exclusion of arcs. To identify the estimand(s) of interest, we searched and examined occurrences of ‘estimand’, ‘effect’, ‘caus*’, ‘total’, ‘direct’, ‘indirect’. We inspected whether the implied (sufficient) adjustment set was reported for each estimand or apparent effect of interest, and whether estimates were reported for these sets explicitly. We also examined whether alternative adjustment sets were used and, if so, what approaches were used for covariate selection. Finally, we checked for attempts to evaluate the compatibility of the DAG(s) with the observed dataset (e.g. testing whether there are associations present in the dataset that are not implied by the DAG).^21^

### Errors in DAG data extraction

The probability of ‘error’ in the initially-extracted number of nodes and arcs was explored in relation to the most common diagrammatic features (i.e. whether drawn in DAGitty, whether unobserved variables or ‘super-nodes’ were included, and whether arcs were visually arranged in the same direction), and adjusted for number of arcs and nodes by log-linear regression with robust standard errors. Reported probabilities represent model marginal values, with 95% confidence intervals (CI) approximated by the delta-method.

## Results

### Sample description

**Figure 2:**
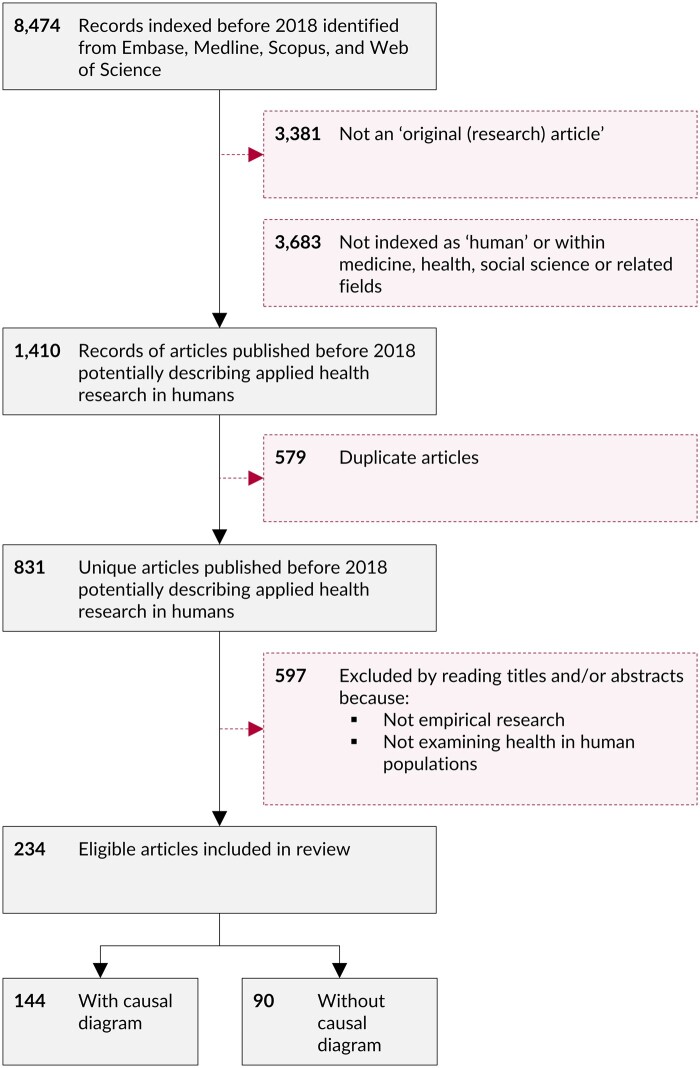
Flow of bibliographic records into the final sample of 234 articles.

**Figure 3.**
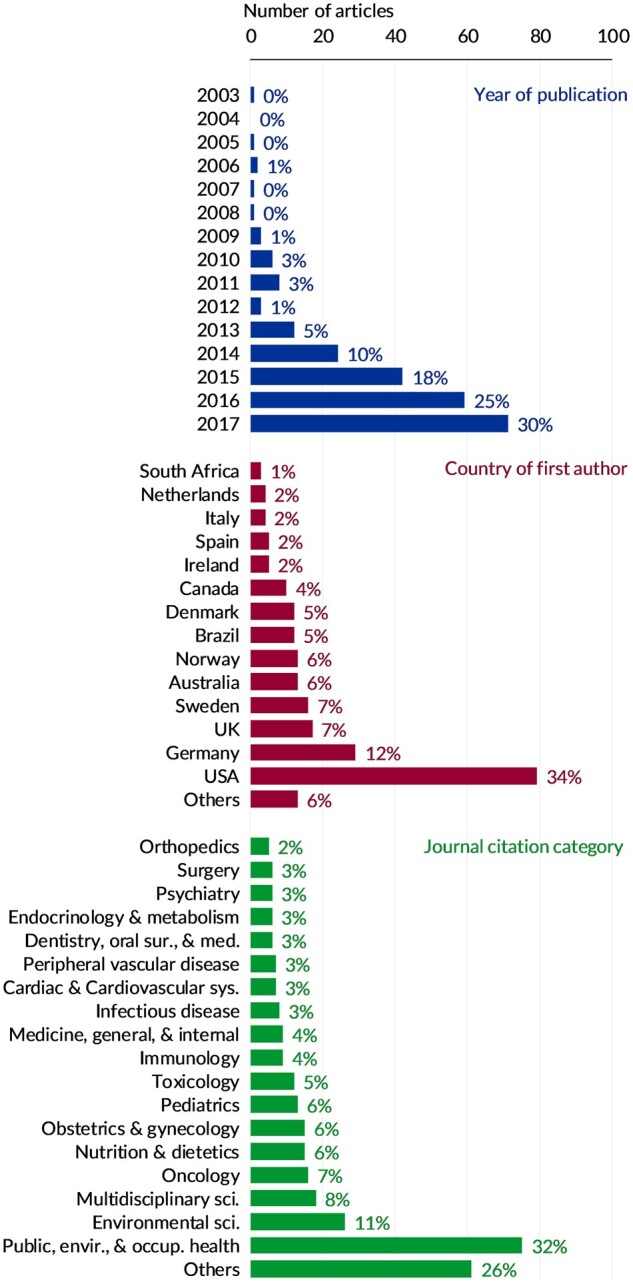
Distribution of the 234 articles included in the review sample, by year of publication, country of first author’s primary affiliation and journal citation category.

Figure 2 summarises the derivation of the study sample. A total of 234 eligible articles were identified, including 172 (73.5%) published since the start of 2015 (Figure 3). A total of 230 (98%) articles were written in English and four (2%) were written in German. Brief details of each paper are provided in Supplementary Table S1, available as Supplementary data at *IJE* online.

Figure 3 shows the number of articles published by the country of the first author's primary affiliation. The most prevalent countries were USA (*n* = 79, 34%), Germany (*n* = 29, 12%), UK (*n* = 17, 7%) and Sweden (*n* = 16, 7%). A total of 187 (80%) were led by North American or Northern European authors.

Articles were published in 152 distinct journals. A total of 33 (22%) journals published more than one article, the most appearing in *PLoS One* (*n* = 18), *Environmental Health Perspectives* (*n* = 10) and *Environmental Research* (*n* = 8). These 152 journals covered 50 citation categories, the most prevalent being Public, Environmental, and Occupational Health (*n* = 75, 32%), Environmental Science (*n* = 26, 11%) and Multidisciplinary Science (*n* = 18, 8%) (**Figure 3**).

### DAG availability, size and attributes

DAGs were available for 144 (62%) articles. Sixty (42% of those with one or more DAGs available) included the DAG in the manuscript directly, 81 (56%) in supplementary online material, 1 (<1%) provided the DAG on request,^24^ 1 (<1%) provided a link to the DAG on *www.dagitty.net*^25^ and 1 (<1%) referenced a DAG from a previous publication.^26^ No DAG was available for 90 (38%) articles, including 4 (1%) articles that referred to Supplementary online materials that were unavailable, 1 article where the DAG was missing from Supplementary Material, 1 (<1%) where the printed figure was incorrect, and 1 (<1%) that provided invalid weblinks to *www.dagitty.net*.

Of the 144 articles with available DAGs, 116 (81%) included a single DAG and 28 (19%) more than one. Full details of the largest DAG in each of these 144 articles are provided in Supplementary Table S2, available as Supplementary data at *IJE* online, and summary details are in Table 1. DAGs varied substantially in size and complexity, with the number of nodes ranging from 3 to 28 (median = 12, IQR = 9–16) and the number of arcs ranging from 3 to 99 (median = 29, IQR = 19–42). The median ratio of arcs to nodes was 2.3 (IQR = 1.8–3.0, range = 1.0–5.8) and the median saturation (i.e. percentage of total possible arcs) was 46% (IQR = 31–67, range 12–100). Just four (3%) DAGs were saturated (i.e. included all possible arcs).

**Table 1.** Summary information regarding the reporting of estimands and adjustment sets in the 234 included studies, and regarding the reporting and features of the largest DAG in the 144 studies with ≥1 DAG

DAG reporting and features^a^	*n*	% (*n* = 144)	
DAG available	144	100%	
Single DAG available	116	81%	
Multiple DAGs available	28	19%	
DAG includes one or more unobserved variables	53	37%	
DAG includes^a^ one or more specific unobserved variables	27	19%	
DAG includes^a^ one or more generic unobserved variables	29	20%	
Visually arranged so all arcs flow in the same direction	49	34%	
Top-to-bottom	5	3%	
Left-to-right	22	15%	
Corner-to-corner	22	15%	
Authors provide citations for one or more arcs	8	6%	

DAG nodes and arcs	Median	IQR	Range

Number of nodes	12	9–16	3–28
Number of arcs	29	19–42	3–99
Ratio of arcs-to-nodes	2.3	1.8–3.0	1.0–5.8
Saturation (%)^b^	46	31–67	12–100

Reporting of estimand(s) and adjustment set(s)	*n*	% (*n* = 234)	

Report one or more estimand(s) of interest	48	21%	
Report seeking total causal effects	18	8%	
Report seeking direct causal effects	12	5%	
Report seeking multiple effects	18	8%	
Report DAG-implied adjustment set(s)	115	49%	
Report results of DAG-implied adjustment set(s)	101	43%	
Report as primary results	95	41%	
Report results of other or unclear adjustment set(s)	171	73%	
Report as primary results	159	68%	
Use additional statistical criteria for variable selection	42	18%	

a Details are for the largest DAG reported in each study.

b The saturation percentage represents the proportion of all possible arcs that have been included.

Fifty-three (37%) DAGs included one or more unobserved variables [see Supplementary Figures S1a and b), available as Supplementary data at *IJE* online], 27 (19%) included one or more specific unobserved variables, and 29 (20%) included one or more generic unobserved variables. Twenty-five (17%) DAGs included one or more ‘super-nodes’ (see Supplementary Figure S1c, available as Supplementary data at *IJE* online). Forty-nine (34%) DAGs were visually arranged so that the constituent arcs flowed in the same direction (see Supplementary Figure S1d and S1e, available as Supplementary data at *IJE* online, for contrast; five (3%) flowed from top-to-bottom, 22 (15%) flowed from left-to-right, and 22 (15%) flowed diagonally from one corner to another. Eight (6%) provided citations to support the inclusion of one or more arcs (see Supplementary Figure S1f, available as Supplementary data at *IJE* online). No articles reported attempting to evaluate the compatibility of their DAG(s) with their data.

The 84 (36%) articles with no available DAG (by intent rather than error) generally offered limited information beyond stating that the DAG(s) had been ‘constructed’ or ‘used’ to ‘guide’, ‘identify’, ‘determine’ and/or ‘select’ ‘confounders’ or ‘covariates’ for adjustment [e.g. ‘Based on previous research, causal diagrams and directed acyclic graphs were constructed to identify a minimally sufficient adjustment set of confounders’^27^ (Supplementary Tables S4 and S5, available as Supplementary data at *IJE* online). Only 36 (43%) explicitly stated using their DAG to identify a sufficient adjustment set. A total of 41 (49%) explicitly reported using DAGitty, including 22 (26%) who explicitly stated using DAGitty to identify a sufficient adjustment set.

### Errors in DAG data extraction

First round data extraction errors occurred among 56 (39%) DAGs; including 2 (1%) where the number of nodes was miscounted, 48 (33%) where the number of arcs was miscounted, and 6 (4%) where both were miscounted. The proportion of errors increased with the number of nodes [4.2% per node (95% CI: 1.5, 7.0)] or arcs [1.6% per arc (95% CI: 1.0, 2.2)]. Conditional on these, the proportion of errors was lower among DAGs drawn in DAGitty [32% (95% CI: 23, 42) vs 50% (95% CI: 36, 64) among DAGs not drawn in DAGitty] or that had been visually arranged so that the constituent arcs flowed from top-to-bottom or left-to-right [30% (12, 47) vs 41% (95% CI: 31, 51) among DAGs without consistent direction] but not diagonally from corner-to-corner [44% (95% CI: 20, 67)] (Supplementary Table S3, available as Supplementary data at *IJE* online). There were negligible differences in the proportion of errors among DAGs that included unobserved variables [42% (95% CI: 28, 56) vs 37% (95% CI: 31, 51) without unobserved variables] or super-nodes [39% (95% CI: 17, 61) vs 39% (95% CI: 31, 48) without super-nodes] (Supplementary Table S3, available as Supplementary data at *IJE* online).

### Reporting of estimands and adjustment sets

Of the 234 included articles, 208 (89%) conducted multivariable regression analyses, 13 (6%) conducted mediation analyses, 4 (2%) conducted g-method analyses, and 9 (2%) conducted other or mixed analyses.

Full details regarding the reporting of estimands and adjustment sets are provided in Supplementary Table S4, available as Supplementary data at *IJE* online, whereas summary details are in Table 1. Only 48 (21%) articles explicitly reported one or more causal estimands of interest, comprising 18 (8%) that sought total causal effects, 12 (5%) that sought direct causal effects and 18 (8%) that sought multiple effects. A total of 115 (49%) articles clearly reported the adjustment set(s) implied by their DAG(s), including 90 (38%) who specifically stated one or more sufficient adjustment set(s).

Of the 115 articles that reported one or more DAG-implied adjustment set(s), 101 (88%) reported the causal effect estimate obtained from this adjustment set specifically (i.e. with no covariates added or removed), including 95 (76%) where these formed the primary analyses. Estimates from other (i.e. bespoke) or unclearly derived adjustment sets were reported in 171 (73%) of the total 234 articles, including 159 (68%) where these formed the primary analyses. A total of 42 (18%) studies applied additional statistical criteria or algorithms for covariate selection, including 28 (12%) that used change-in-estimate criteria and 14 (6%) that used *P*-value criteria.

## Discussion

### Summary of findings

This review examined the use of DAGs in applied health research. Although they remain very rare within the wider literature, an increasing absolute number of observational studies in health, medicine and related areas are using and presenting DAGs to aid confounder selection. There is however substantial variation in the way these DAGs are reported and used, with potential impacts on the study results and the contributions towards increasing transparency and scrutiny of the analytical assumptions.

DAGs were not always presented, and in some instances very little information was provided regarding their construction or use (see Supplementary Tables S4 and S5, available as Supplementary data at *IJE* online). Where DAGs were presented, they varied substantially in the number of variables and arcs included, the inclusion of unobserved variables, the inclusion of ‘super-nodes’, details on their design, and how they were used to inform the subsequent statistical modelling. Some studies presented simplified illustrations of the relationships between only a few variables considered key to the focal relationship (e.g.^28^) while others presented large schematics with multiple interconnected measured variables (e.g.^29^), but few DAGs incorporated many, if any, unobserved variables. In general, DAGs included relatively few arcs per node and very few were saturated.

Only half of the articles reported the adjustment set(s) implied by their DAG(s), and only a fifth stated their target estimands, with most instead referring to the ‘association’, ‘relationship’, or ‘effect’. Several studies used additional statistical criteria or algorithms to reduce the number of candidate variables, whereas others made bespoke modifications to their adjustment set(s) for other subjective reasons not implied by their DAG(s) (e.g. ‘An additional set of covariates was considered important to include in the full model because of their well-established association with exposure and outcome’^30^) (see Supplementary Tables S4 and S5, available as Supplementary data at *IJE* online). No articles reported attempting to evaluate the compatibility of their DAG(s) with their data.

### Observations and remarks

This review explicitly considered the use of DAGs in applied health research, where they were used not simply to discuss causal inference theory but to ‘identify variables necessary for adjustment’.^31^ Although such applications are widely marketed as a key benefit of DAGs, it is contested whether they actually lead to more accurate and/or reliable effect estimates.^16^^,^^17^

Harder to dispute are the potential benefits for transparency. Compared with traditional approaches to selecting candidate variables and adjustment sets, DAGs encourage observational data scientists to declare their assumptions about the data-generating process for the data they are analysing, which clearly facilitates external scrutiny. Reporting the estimand(s) of interest and DAG-implied adjustment set(s) offer similar benefits to the clarity of the study aim(s) and the model interpretability.

Unfortunately, many of the articles reviewed did not report their DAG(s) and fewer still declared their estimand(s) of interest. Reluctance to share such details may reflect a lack of awareness of the benefits of these details for readers, lack of confidence, fear of criticism, cultural reticence, and/or a lack of encouragement or facilitation from journals, editors and reviewers. These may also explain why some articles adopted hybrid approaches to variable selection that combined DAG-based approaches with traditional statistical approaches, or where investigators overruled the DAG-implied adjustment set(s) by adding or removing covariates. Such actions suggest a need for practical guidance and supporting materials that are accessible to data analysts, journal editors and reviewers. Regardless, several recommendations emerge from recognising the best practices among the studies reviewed (see below).

Further benefits may also be achieved by emphasising the different assumptions made from the inclusion and omission of arcs within DAGs. Although a small number of articles reported saturated DAGs, the majority of the available DAGs included less than half of all possible arcs. Since a confounder must cause both the exposure and outcome, inadvertently omitting either of these arcs may result in a confounder being mislabelled and omitted from the DAG-implied adjustment set, leading to unadjusted confounding. Such omissions are theoretically detectable by testing the DAG(s)-dataset consistency, but this was not attempted in any of the articles examined. Researchers may not be aware that such testing is possible or may be concerned about overfitting from *post hoc* modification;^32^ this indicates a need for additional clarity and/or guidance. The tendency to omit arcs may be explained by a cultural aversion to declaring potential causal relationships in observational data without a strong theoretical basis or definitive empirical evidence.^4^ This may also explain why some authors included citations to justify the inclusion of arcs within their DAGs. This is arguably unnecessary since omitting an arc invokes the greater assumption, because no arc between two variables declares there is no causal relationship between the two variables, regardless of direction, sign, strength or parametric form.^12^ Increasing the number of arcs does however bring important practical challenges that may also explain the absence of plausible arcs. In general, it is difficult to draw DAGs with multiple nodes and multiple arcs while ensuring that all potential arcs have been included and that the DAG remains decipherable to the reader. Larger and more saturated DAGs are often cluttered and unsightly, diminishing their appeal and utility. We observed that the proportion of data-extraction errors increased with the number of nodes and arcs, although this could also result from an increasing number of objects available for miscounting. Subjectively, we judged that DAGs that had been visually arranged so that their constituent arcs flowed consistently from left-to-right or top-to-bottom were easier to interpret and critique. This appears to be supported by the lower proportion of data-extraction errors among DAGs that had been arranged in either of these ways. Several algorithms exist to help with arranging a DAG into strict temporal ‘layers’,^33^ but they do not appear to have been widely adopted in applied settings. For saturated DAGs, it may be reasonable to simplify several details (e.g. using paired arrow-heads indicating ‘arcs to all future nodes’),^34^ because the sufficient adjustment set for any exposure–outcome relationship will simply include all variables that temporally precede the exposure. However, no simplification should compromise the level of thought and rigor given to considering the data-generating process. Additional guidance, notation or software may be needed to reconcile the task of drawing clear yet well-specified DAGs in applied health research.

### Recommendations

#### The focal relationship(s) and estimand(s) of interest should be stated in the study aims

Causal inference methods separate the process of identifying the quantity of interest (i.e. estimand) from the process of estimating that quantity, with the latter being informed by the former. To reflect this, all estimands of interest should be clearly stated in the study aims or at the beginning of the article’s methods section before details on how estimation was attempted (e.g. ‘This study aimed to estimate the total causal effect of type-2 diabetes at aged 50-years on the ten-year risk of myocardial infarction.’). Where the estimand does not describe the average (treatment) effect (ATE) in the population this should also be stated (e.g. ‘The average treatment effect in the treated, ATT, of type-2 diabetes on the ten-year risk of myocardial infarction would be the effect observed in the subgroup of people who develop type-2 diabetes.’).

#### The DAG(s) for each focal relationship and estimand of interest should be available

DAGs explicitly depict the investigators’ assumptions about the data-generating process of their dataset. The accuracy of any ensuing effect estimate is fundamentally contingent on the extent to which the DAG (as specified) reflects the true data-generating process. The DAG used to inform the model for estimating every causal effect estimate should therefore be available to all potential readers. This may be achieved by reproducing the DAG in the manuscript directly, in supplementary material or by providing functional weblinks to a well-established open-source platform such as *www.dagitty.net*.

#### DAGs should include all relevant variables, including those where direct measurements are unavailable

The DAG for a specific focal relationship should include all plausible confounding variables (i.e. that may plausibly cause both the exposure and the outcome), regardless of whether direct measurements are available or possible. Explicitly depicting unobserved variables helps to highlight potential sources of unobserved confounding.

#### Variables should be visually arranged so that all constituent arcs flow in the same direction

Arcs depict causal processes that occur over time. DAGs, and the relationships they symbolize, are therefore considerably easier to interpret when the constituent variables are arranged spatially in a way that clearly reflects the passage of time, with arcs flowing in the same direction from left-to-right or top-to-bottom.

#### Arcs should generally be assumed to exist between any two variables

Omitting an arc between two variables implies that there is precisely no causal effect of one on the other. This is a much stronger statistical assumption than is implied when an arc is included, which allows a causal effect of any sign, magnitude or parametric form (including a very small effect). Omitted arcs should therefore be carefully considered and ideally justified with theory and/or evidence.

#### The DAG-implied adjustment set(s) for the estimand(s) of interest should be clearly stated

The DAG-implied adjustment set(s) for every estimand of interest should be stated explicitly, including variables for which measurements are not available for conditioning.

#### The estimate(s) obtained from using the unmodified DAG-implied adjustment set(s)—or nearest approximation thereof—should be reported

The estimate(s) obtained from using the unmodified DAG-implied adjustment set(s) should be reported, even if not considered or interpreted as central to the study findings. If one or more variables are not available, the estimate(s) from the most complete subset should be reported; this will usually comprise all observed variables within the DAG-implied adjustment set. Where possible, bias analyses should be performed to quantify the impact of unobserved confounders and obtain more accurate causal effect estimates.^35^

#### Alternative adjustment set(s) should be justified and their estimate(s) reported separately

If alternative adjustment set(s) are used, they should be clearly described and justified, and the ensuing estimates should be reported separately to those reported using the DAG-implied adjustment set. Modifications to DAG-implied adjustment sets may comprise the inclusion of competing exposures, surrogate confounders or variables with ambiguous causal roles for the purposes of sensitivity analyses. Where direct causal effects are sought, these should be clearly stated in the aims as effects of interest, and their adjustment sets derived accordingly. We encourage researchers to explore the consistency of all DAGs with the observed data, but the details of, and estimates from, any subsequently modified DAGs should again be reported separately.^21^

Supplementary Table S6, available as Supplementary data at *IJE* online summarises these recommendations in the form of a checklist, to assist with preparing and reviewing articles that use DAGs to identify confounders.

### Strengths and weaknesses

This is the largest and most comprehensive review of the use of DAGs in applied research, whether in health, medicine or otherwise. We identified and examined >200 articles published over several years across a diverse range of fields. The sample is however somewhat ill-defined and should not be considered to reflect the population of observational health research studies that have used DAGs. It is not clear what proportion of such studies mention their use of DAGs in their abstract or keywords, but we suspect many do not. No articles were identified that reported using DAGs to aid with understanding or analysing randomized controlled trials or natural experiment approaches (such as instrumental variables analyses), indicating that users of these methods may be less familiar with, or enthusiastic towards, DAGs.

A large proportion of the sample was therefore identified from having cited the DAGitty.net software package. Studies that used DAGitty.net for drawing their DAG(s) or determining their adjustment set(s) are likely over-represented. A lower proportion of data-extraction errors occurred among DAGs that had been drawn using DAGitty, which may indicate some feature differences, but it may also indicate a training effect due to increased reviewer familiarity. We believe that the issues we describe, and their implications, are likely to be applicable to the use and reporting of DAGs irrespective of the tools used to construct and scrutinize these DAG(s).

Extracting data for the 234 articles included in this review was laborious and a substantial time therefore passed between when the sample was identified and extraction was completed. We nevertheless decided not to update our search to include more recent articles (i.e. published from 2018 onwards) because we do not believe there will have been any substantial changes in practice in the intervening time.

Because of the quantity of information sought and the diversity of the studies examined, it was impossible to develop a data-extraction form that was entirely compatible with all the included articles. Some data items therefore required subjective judgement, inter-rater discussion, and/or further simplification, and others simply could not be synthesised due to a lack of standardization in reporting. Even when using a reduced set of items for data extraction, clarity and transparency varied substantially between studies, making it challenging to accurately identify all relevant information. All data were therefore extracted in triplicate, with the first extraction used to evaluate the ‘readability’ of the most objective DAG features. Occasional data extraction errors or discordances between the authors’ intended message and our interpretation are nevertheless still inevitable; but these should not materially alter the review results, messages and/or recommendations.

We offer several recommendations for improving the reporting, specification and application of DAGs in applied health research where causal effect estimates are sought from observational data. These recommendations are supported by our innovative approach to data extraction, which allowed us to empirically identify features that led to fewer data-extraction errors and clearer DAGs. Studies that did not follow these recommendations should not however be considered necessarily less rigorous, less accurate or less valuable. DAGs represent the investigators’ assumptions and hypotheses about the data-generating process for a specific dataset. Where the DAG does not accurately reflect the true data-generating mechanism, the ensuing estimates are likely to be unreliable. However, since the true process can never be known, a DAG can arguably only be wrong if it fails to correctly represent those assumptions and hypotheses. We therefore did not attempt to evaluate the plausibility of individual DAGs and instead focussed on identifying those areas where the implications of the DAG may not have met the investigators’ intentions. We offer no negative judgements on the intentions of individual authors or the veracity of individual studies. On the contrary, we welcome the large and growing number of applied health researchers who have used DAGs to assist with estimating causal effects in observational data and explored their benefits for declaring their assumptions, identifying potential sources of bias, identifying data for collection and improving statistical analyses. These ‘early adopters’ have not only helped to reveal some potential pitfalls in the use of DAGs but have provided a growing wealth of innovative exemplars that will inspire future developments in this evolving field.

## Conclusion

DAGs are increasingly popular in applied health research as a transparent means of identifying confounding variables that require conditioning to estimate causal effects. This review examined their use in >200 empirical studies of health, medicine and related disciplines, and found substantial variation in their size, structure, complexity, availability and implementation. Whereas such variety partly reflects the inherent flexibility and subjectivity of DAGs, it also helps to highlight several potential pitfalls and aspirational practices. Consequently, we offer a list of simple recommendations for improving both the transparency and benefits of DAGs in observational research that we hope will help towards the ongoing development of these techniques.

## Supplementary data

Supplementary data are available at *IJE* online.

## Funding

This study received no specific funding. K.F.A. and S.C.G. are grateful for PhD funding from the Economic and Social Research Council, UK (ES/J500215/1 and ES/P000746/1, respectively). L.B. is grateful for PhD funding from the Medical Research Council, UK (MR/K501402/1). G.D.T. is grateful for PhD funding from The Alan Turing Institute (EP/N510129/1). M.S.G. and P.W.G.T. are both supported by The Alan Turing Institute (EP/N510129/1).

## Data availability

The data underlying this article are available in the article and in its online Supplementary Material, available as Supplementary data at *IJE* online.

## Supplementary Material

dyaa213_Supplementary_Data
