# Supplementary material for: Use of directed acyclic graphs (DAGs) to identify confounders in applied health research: review and recommendations
Source: Int J Epidemiol. 2020 Dec 17;50(2):620–32. doi: 10.1093/ije/dyaa213 (PMC8128477; doi:10.1093/ije/dyaa213)
Supplement: dyaa213_Supplementary_Data [file dyaa213_supplementary_data.zip › ije-2020-02-0279-File006.docx]

SUPPLEMENTARY MATERIAL

**Use of directed acyclic graphs (DAGs) to identify confounders in applied health research: review and recommendations**

#

# CONTENTS

[Supplementary Figure S1. 2](#_Toc51924433)

[(a) 2](#_Toc51924434)

[(b) 3](#_Toc51924435)

[(c) 4](#_Toc51924436)

[(d) 5](#_Toc51924437)

[(e) 6](#_Toc51924438)

[(f) 7](#_Toc51924439)

[Supplementary Table S1. 8](#_Toc51924440)

[Supplementary Table S2. 27](#_Toc51924441)

[Supplementary Table S3. 33](#_Toc51924442)

[Supplementary Table S4. 34](#_Toc51924443)

[Supplementary Table S5. 52](#_Toc51924444)

[Supplementary Table S6. 102](#_Toc51924445)

#

# **Supplementary Figure S1****.**

## (a)

Showing a DAG where several unobserved variables have been included; here denoted with the letter U. This helps to highlight potentially important sources of unobserved confounding.


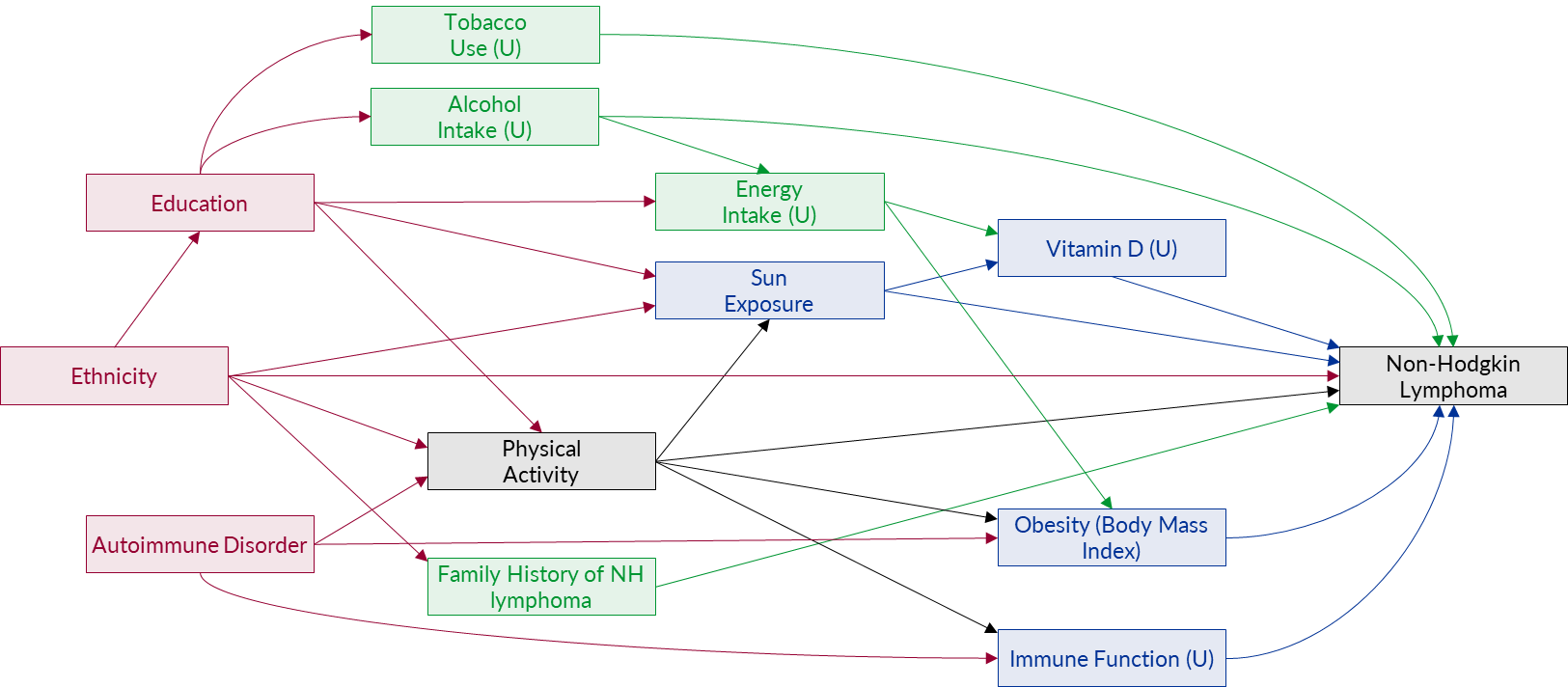


**ADAPTED FROM:** Boyle T, Gallagher RP, Gascoyne RD, Connors JM, Le ND, Spinelli JJ. Lifetime physical activity and the risk of non-Hodgkin lymphoma. *Cancer Epidemiol Biomarkers Prev*. 2015;24(5):873-7. DOI: 10.1158/1055-9965.EPI-14-1303 (Supplementary Figure 2). Freely available at: <https://cebp.aacrjournals.org/content/24/5/873.long>

.

## (b)

Showing a DAG where a (single) generic variable has been included to represent a cluster of unobserved variables; here denoted using Structural Equation Modelling notation with an ellipse. This helps to emphasise the collective influence of these unobserved variables but does less to indicate which of the underlying variables are thought most important.


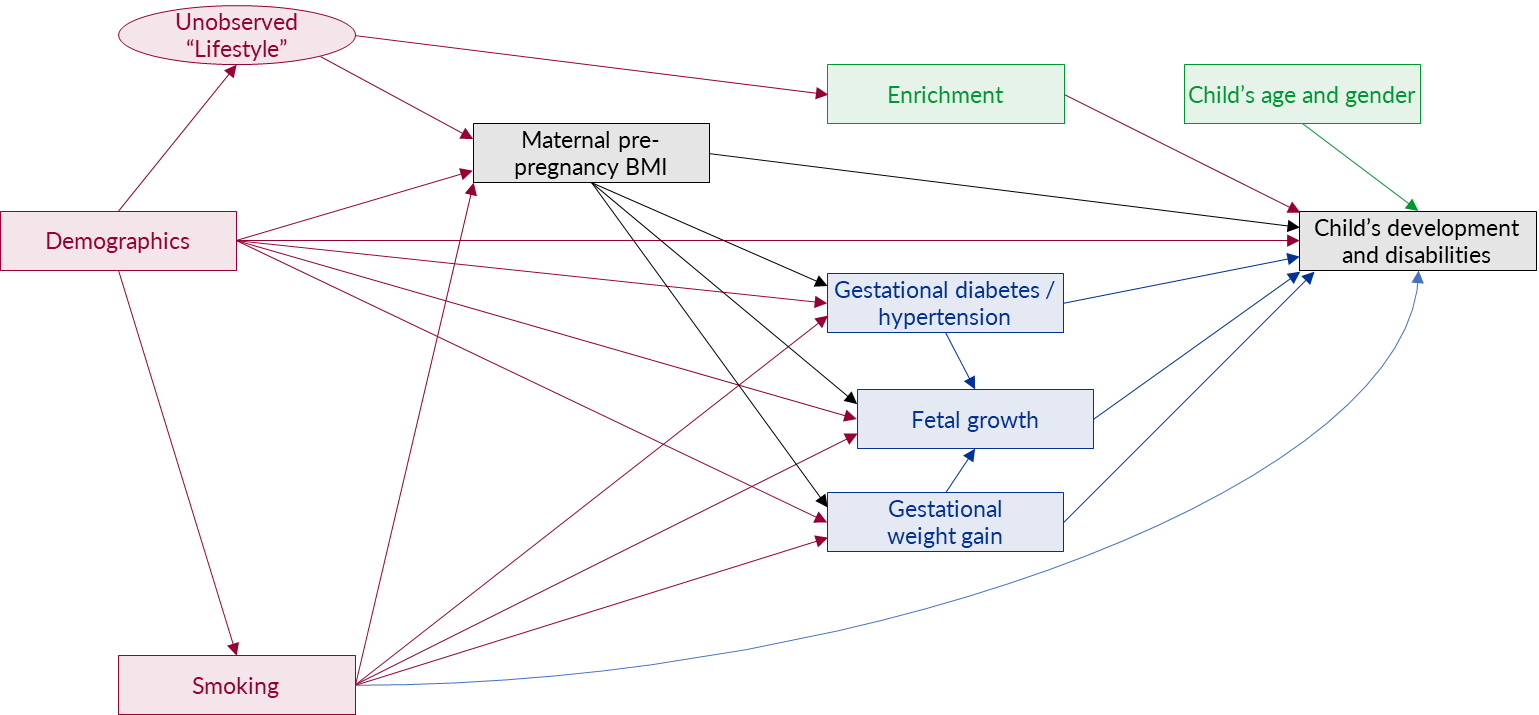


**ADAPTED FROM:** Hinkle SN, Sharma AJ, Kim SY, Schieve LA. Maternal prepregnancy weight status and associations with children’s development and disabilities at kindergarten. *Int J Obes (Lond).* 2013;37(10):1344-51. DOI: 10.1038/ijo.2013.128 (Figure 1)*.* Freely available at: <https://www.ncbi.nlm.nih.gov/pmc/articles/PMC4407562>

## (c)

Showing a DAG that includes super-nodes, i.e. nodes that contain more than one variable. In this diagram the 'demography' node includes 'age', 'sex', and 'having children ≤ 5 years', the socioeconomic status node includes 'education' and 'household income' and the 'lifestyle' node includes 'smoking status', 'alcohol use', 'caffeine use', 'physical activity', and 'night shift work'.


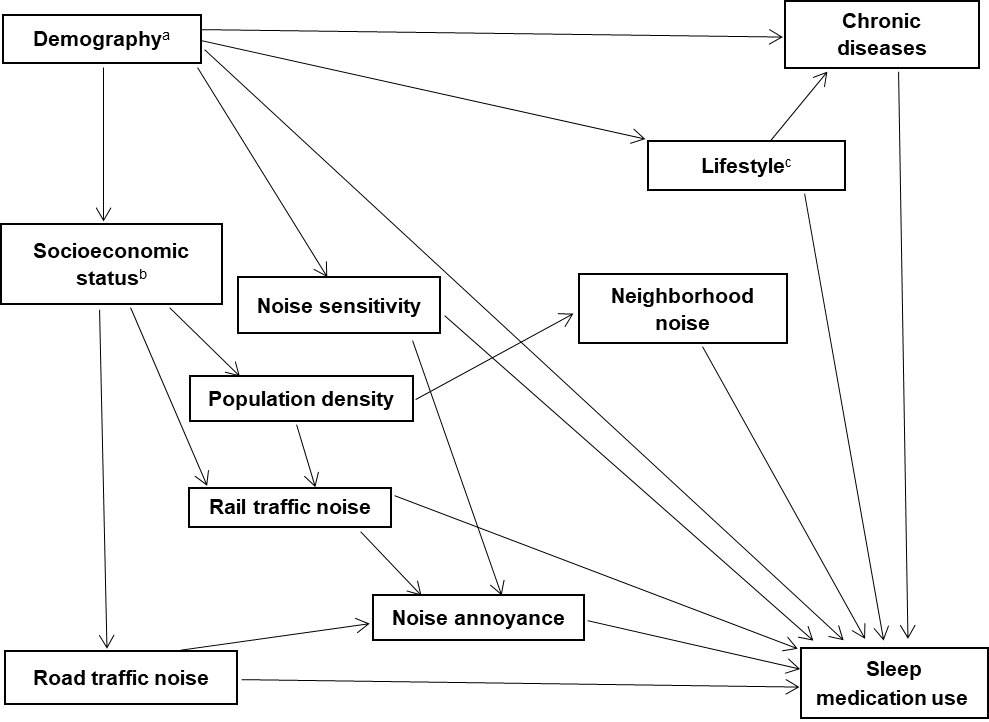


**REPRODUCED UNDER CC-BY 4.0 LICENSE FROM:** Evandt J, Oftedal B, Krog NH, et al. Road traffic noise and registry based use of sleep medication. *Environ Health.* 2017;16(1):110. DOI: 10.1186/s12940-017-0330-5 (Figure S1). Freely available at: <https://www.doi.org/10.1186/s12940-017-0330-5>

## (d)

Showing a DAG, which has been arranged so the constituent arcs flow consistently from left-to-right. Diagrams arranged in this manner are easier to interpret.


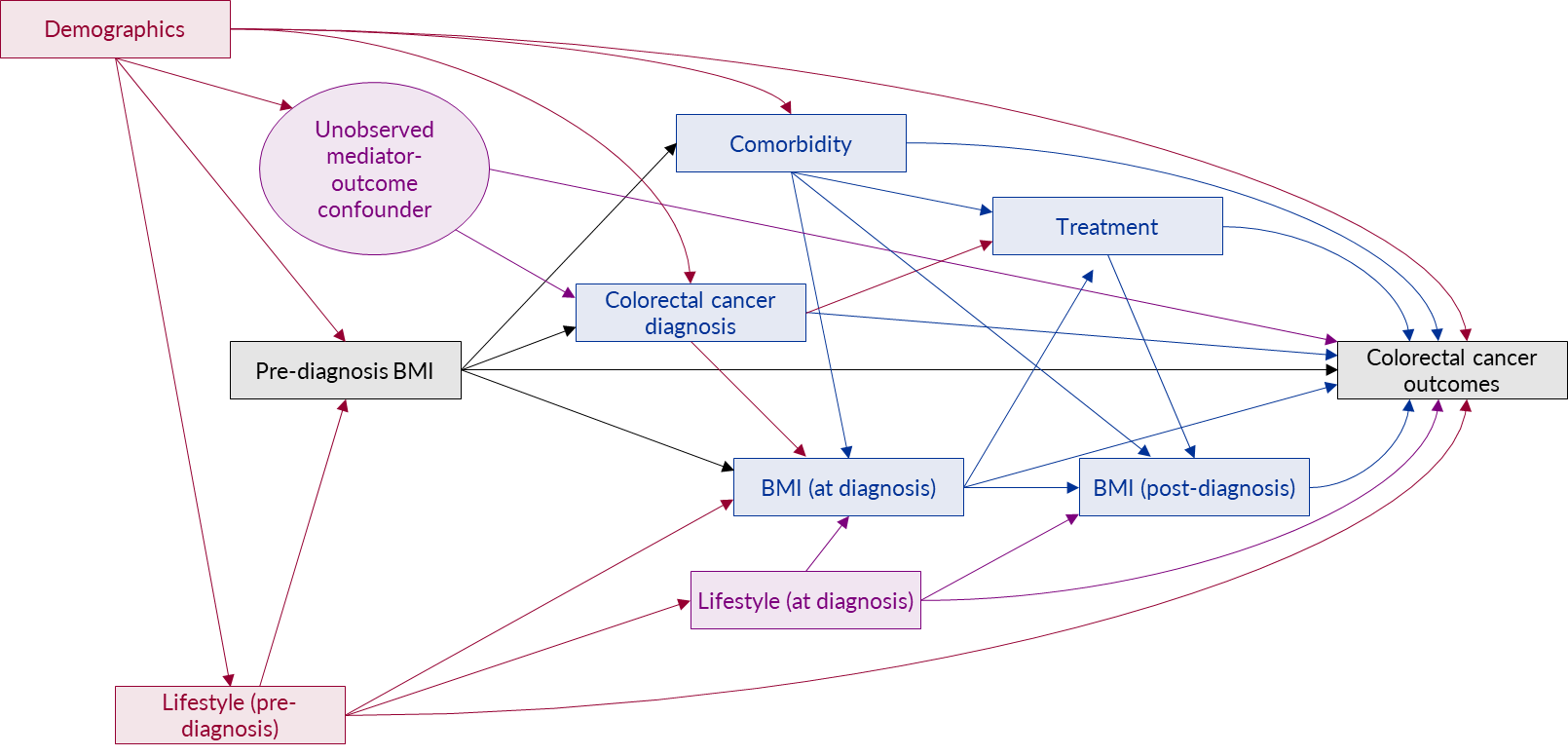


**ADAPTED FROM:** Kroenke CH, Neugebauer R, Meyerhardt J, et al. Analysis of Body Mass Index and Mortality in Patients With Colorectal Cancer Using Causal Diagrams. *JAMA Oncol.* 2016;2(9):1137-45. DOI: 10.1001/jamaoncol.2016.0732. Freely available at: <https://www.ncbi.nlm.nih.gov/pmc/articles/PMC5016213/>

## (e)

Showing a DAG where the arcs do not flow in a consistent direction. This has no impact on the plausibility or validity of the diagram but does make it harder to interpret.

**
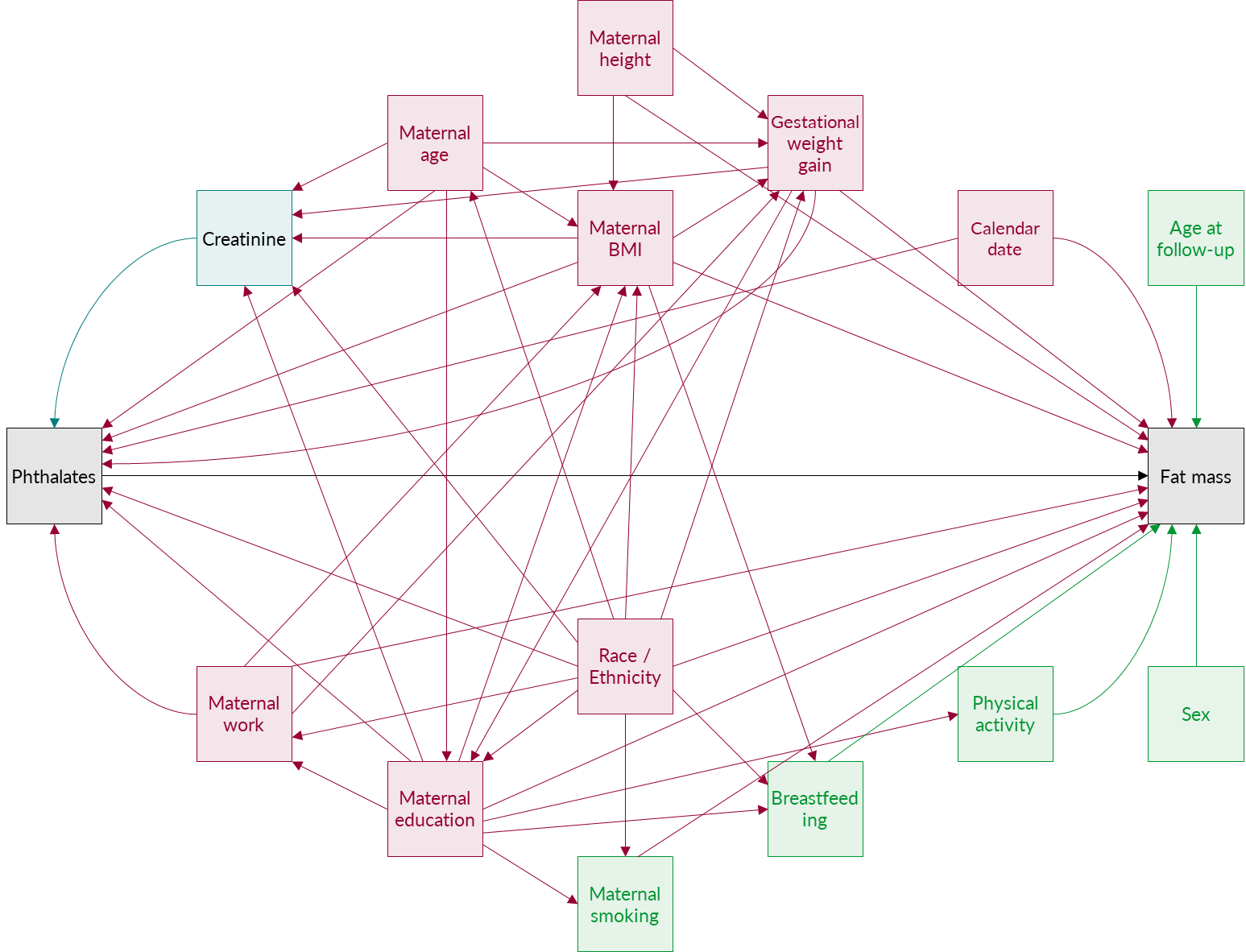
**

**ADAPTED FROM:** Buckley JP, Engel SM, Mendez MA, et al. Prenatal Phthalate Exposures and Childhood Fat Mass in a New York City Cohort. *Environ Health Perspect.* 2016; 124(4): 507–513. DOI: 10.1289/ehp.1509788. Freely available at: <https://www.ncbi.nlm.nih.gov/pmc/articles/PMC4829985/>

## (f)

Showing a DAG where references to previous empirical research have been added to individual arcs to justify their inclusion.


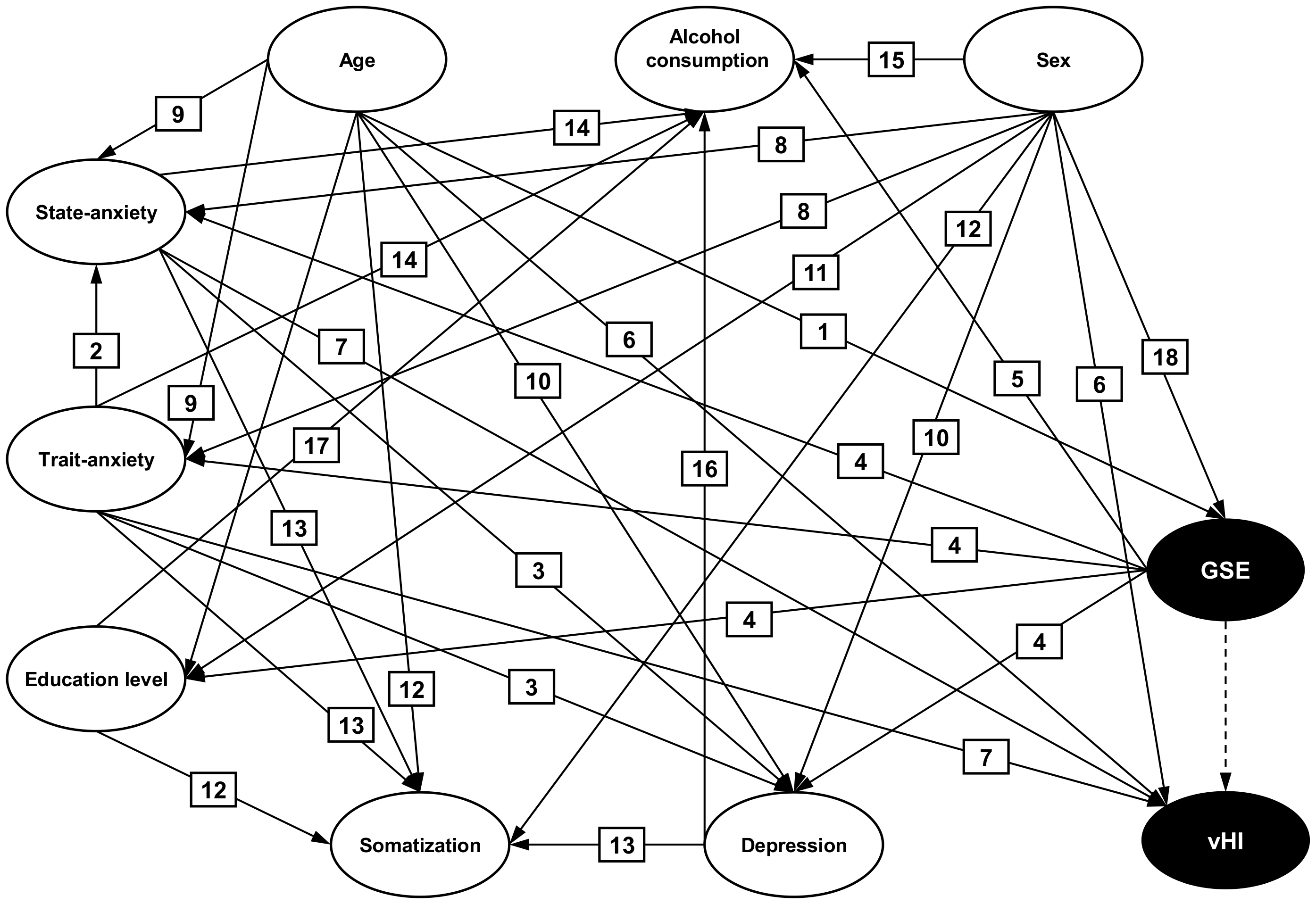


**REPRODUCED UNDER CC-BY 4.0 LICENSE FROM:** Grill E, Schäffler F, Huppert D, Müller M, Kapfhammer H-P, Brandt T. Self-Efficacy Beliefs Are Associated with Visual Height Intolerance: A Cross-Sectional Survey. *PLoS One*. 2014;9(12):e116220. doi: 10.1371/journal.pone.0116220: <https://doi.org/10.1371/journal.pone.0116220>

# **Supplementary Table S1.**

Summary details of the 234 articles included in the review.

| **Citation** | **Title** | **Journal** | **Journal citation categories** | **Language** | **Country (first author)** | **Number of DAGs available** | **DOI** |
| --- | --- | --- | --- | --- | --- | --- | --- |
| Åkerstedt *et al.* 2017 | Sleep duration, mortality and the influence of age | European Journal of Epidemiology | Public, environmental, & occupational health | English | Sweden | 1 - in supplementary material | [10.1007/s10654-017-0297-0](http://www.doi.org/10.1007/s10654-017-0297-0) |
| Al-Farsi *et al.* 2010 | Effect of high parity on the occurrence of prediabetes: A cohort study | Acta Obstetricia et Gynecologica Scandinavica | Obstetrics & Gynecology | English | Oman | 0 | [10.3109/00016349.2010.501854](http://www.doi.org/10.3109/00016349.2010.501854) |
| Al-Harbi & El Tantawi 2017 | Normative prosthodontic care need: Does it impact the daily life of young Saudis with high level of oral diseases? A cross sectional study | BMC Oral Health | Dentistry, oral surgery & medicine | English | Kingdom of Saudi Arabia | 2 - in manuscript | [10.1186/s12903-017-0418-x](http://www.doi.org/10.1186/s12903-017-0418-x) |
| Andell *et al.* 2014 | Impact of chronic obstructive pulmonary disease on morbidity and mortality after myocardial infarction | Open Heart | Not indexed | English | Sweden | 0 - supplementary material unavailable | [10.1136/openhrt-2013-000002](http://www.doi.org/10.1136/openhrt-2013-000002) |
| Andell *et al.* 2015 | β-Blocker Use and Mortality in COPD Patients After Myocardial Infarction: A Swedish Nationwide Observational Study | Journal of the American Heart Association | Cardiac & Cardiovascular systems | English | Sweden | 0 - supplementary material unavailable | [10.1161/JAHA.114.001611](http://www.doi.org/10.1161/JAHA.114.001611) |
| Andersen *et al.* 2014 | Dose-response relationship of total and leisure time physical activity to risk of heart failure a prospective cohort study | Circulation: Heart Failure | Cardiac & Cardiovascular systems | English | Sweden | 1 - in supplementary material | [10.1161/CIRCHEARTFAILURE.113.001010](http://www.doi.org/10.1161/CIRCHEARTFAILURE.113.001010) |
| Andreano *et al.* 2017 | Adherence to guidelines and breast cancer patients survival: a population-based cohort study analyzed with a causal inference approach | Breast Cancer Research and Treatment | Oncology | English | Italy | 1 - in supplementary material | [10.1007/s10549-017-4210-z](http://www.doi.org/10.1007/s10549-017-4210-z) |
| Arora *et al.* 2014 | Periodontal infection, impaired fasting glucose and impaired glucose tolerance: Results from the Continuous National Health and Nutrition Examination Survey 2009-2010 | Journal of Clinical Periodontology | Dentistry, oral surgery & medicine | English | USA | 2 - in manuscript | [10.1111/jcpe.12258](http://www.doi.org/10.1111/jcpe.12258) |
| Asgari *et al.* 2011 | Predictors of patient satisfaction with Mohs surgery: Analysis of preoperative, intraoperative, and postoperative factors in a prospective cohort | Archives of Dermatology | Dermatology | English | USA | 1 - in manuscript | [10.1001/archdermatol.2011.319](http://www.doi.org/10.1001/archdermatol.2011.319) |
| Ashley-Martin *et al.* 2015 | Prenatal exposure to phthalates, bisphenol A and perfluoroalkyl substances and cord blood levels of IgE, TSLP and IL-33 | Environmental Research | Environmental sciences; Public, environmental, & occupational health | English | Canada | 0 | [10.1016/j.envres.2015.04.010](http://www.doi.org/10.1016/j.envres.2015.04.010) |
| Bach *et al.* 2015 | Perfluoroalkyl acids and time to pregnancy revisited: An update from the Danish National Birth Cohort | Environmental Health: A Global Access Science Source | Environmental sciences; Public, environmental, & occupational health | English | Denmark | 1 - in supplementary material | [10.1186/s12940-015-0040-9](http://www.doi.org/10.1186/s12940-015-0040-9) |
| Badland *et al.* 2017 | Are Area-Level Measures of Employment Associated with Health Behaviours and Outcomes? | Social Indicators Research | Not indexed | English | Australia | 2 - in manuscript | [10.1007/s11205-016-1417-z](http://www.doi.org/10.1007/s11205-016-1417-z) |
| Bahls *et al.* 2017 | Statins are related to impaired exercise capacity in males but not females | PLoS One | Multidisciplinary sciences | English | Germany | 1 - in supplementary material | [10.1371/journal.pone.0179534](http://www.doi.org/10.1371/journal.pone.0179534) |
| Barcelona de Mendoza *et al.* 2016 | Acculturation and Adverse Birth Outcomes in a Predominantly Puerto Rican Population | Maternal and Child Health Journal | Nursing | English | USA | 0 | [10.1007/s10995-015-1901-0](http://www.doi.org/10.1007/s10995-015-1901-0) |
| Barcelona de Mendoza *et al.* 2016A | Acculturation and Intention to Breastfeed among a Population of Predominantly Puerto Rican Women | Birth | Psychiatry | English | USA | 0 | [10.1111/birt.12199](http://www.doi.org/10.1111/birt.12199) |
| Barcelona de Mendoza *et al.* 2016B | Effects of acculturation on prenatal anxiety among Latina women | Archives of Womens Mental Health | Psychiatry | English | USA | 0 | [10.1007/s00737-016-0605-9](http://www.doi.org/10.1007/s00737-016-0605-9) |
| Bernardi *et al.* 2015 | Cesarean delivery and metabolic risk factors in young adults: a Brazilian birth cohort study | American Journal of Clinical Nutrition | Nutrition & dietetics | English | Brazil | 1 - in supplementary material | [10.3945/ajcn.114.105205](http://www.doi.org/10.3945/ajcn.114.105205) |
| Birungi *et al.* 2017 | Assessing causal effects of early life-course factors on early childhood caries in 5-year-old Ugandan children using directed acyclic graphs (DAGs): A prospective cohort study | Community Dentistry and Oral Epidemiology | Dentistry, oral surgery & medicine; Public, environmental, & occupational health | English | Norway | 2 - in manuscript | [10.1111/cdoe.12314](http://www.doi.org/10.1111/cdoe.12314) |
| Bjertness *et al.* 2016 | Prevalence and determinants of hypertension in Myanmar - A nationwide cross-sectional study | BMC Public Health | Public, environmental, & occupational health | English | Norway | 0 | [10.1186/s12889-016-3275-7](http://www.doi.org/10.1186/s12889-016-3275-7) |
| Bliddal *et al.* 2016 | Association of Pre-Pregnancy Body Mass Index, Pregnancy-Related Weight Changes, and Parity With the Risk of Developing Degenerative Musculoskeletal Conditions | Arthritis & Rheumatology | Rheumatology | English | Denmark | 1 - on request | [10.1002/art.39565](http://www.doi.org/10.1002/art.39565) |
| Blighe *et al.* 2017 | Vitamin D prenatal programming of childhood metabolomics profiles at age 3 y | American Journal of Clinical Nutrition | Nutrition & dietetics | English | USA | 1 - in supplementary material | [10.3945/ajcn.117.158220](http://www.doi.org/10.3945/ajcn.117.158220) |
| Blom *et al.* 2015 | Patients presenting at the emergency department with acute abdominal pain are less likely to be admitted to inpatient wards at times of access block: A registry study | Scandinavian Journal of Trauma, Resuscitation and Emergency Medicine | Emergency Medicine | English | Sweden | 1 - in supplementary material | [10.1186/s13049-015-0158-3](http://www.doi.org/10.1186/s13049-015-0158-3) |
| Blomberg *et al.* 2013 | Prehospital trauma life support training of ambulance caregivers and the outcomes of traffic-injury victims in Sweden | Journal of the American College of Surgeons | Surgery | English | Sweden | 1 - in supplementary material | [10.1016/j.jamcollsurg.2013.08.002](http://www.doi.org/10.1016/j.jamcollsurg.2013.08.002) |
| Bodén *et al.* 2015 | Higher mortality after myocardial infarction in patients with severe mental illness: A nationwide cohort study | Journal of Internal Medicine | Medicine, general, & internal | English | Sweden | 1 - in supplementary material | [10.1111/joim.12329](http://www.doi.org/10.1111/joim.12329) |
| Bowatte *et al.* 2017 | Traffic-related air pollution exposure over a 5-year period is associated with increased risk of asthma and poor lung function in middle age | European Respiratory Journal | Respiratory system | English | Australia | 0 | [10.1183/13993003.02357-2016](http://www.doi.org/10.1183/13993003.02357-2016) |
| Bowatte *et al.* 2017A | Traffic-related air pollution exposure is associated with allergic sensitization, asthma, and poor lung function in middle age | Journal of Allergy and Clinical Immunology | Allergy; Immunology | English | Australia | 0 | [10.1016/j.jaci.2016.05.008](http://www.doi.org/10.1016/j.jaci.2016.05.008) |
| Boyle *et al.* 2015 | Lifetime physical activity and the risk of non-Hodgkin lymphoma | Cancer Epidemiology Biomarkers and Prevention | Oncology; Public, environmental, & occupational health | English | Canada | 1 - in supplementary material | [10.1158/1055-9965.EPI-14-1303](http://www.doi.org/10.1158/1055-9965.EPI-14-1303) |
| Boyle *et al.* 2016 | Sedentary work and the risk of breast cancer in premenopausal and postmenopausal women: a pooled analysis of two case-control studies | Occupational and Environmental Medicine | Public, environmental, & occupational health | English | Canada | 1 - in supplementary material | [10.1136/oemed-2015-103537](http://www.doi.org/10.1136/oemed-2015-103537) |
| Buchner & Rehfuess 2015 | Cooking and season as risk factors for acute lower respiratory infections in African children: A cross-sectional multi-country analysis | PLoS One | Multidisciplinary sciences | English | Germany | 1 - published elsewhere | [10.1371/journal.pone.0128933](http://www.doi.org/10.1371/journal.pone.0128933) |
| Buckley *et al.* 2016 | Prenatal phthalate exposures and childhood fat mass in a New York city cohort | Environmental Health Perspectives | Environmental sciences; Public, environmental, & occupational health; Toxicology | English | USA | 1 - in supplementary material | [10.1289/ehp.1509788](http://www.doi.org/10.1289/ehp.1509788) |
| Busert *et al.* 2016 | Dietary diversity is positively associated with deviation from expected height in rural nepal | Journal of Nutrition | Nutrition & dietetics | English | Germany | 1 - in supplementary material | [10.3945/jn.115.220137](http://www.doi.org/10.3945/jn.115.220137) |
| Byberg *et al.* 2016 | Body mass index and physical activity in early childhood are associated with atopic sensitization, atopic dermatitis and asthma in later childhood | Clinical and Translational Allergy | Allergy | English | Norway | 1 - in supplementary material | [10.1186/s13601-016-0124-9](http://www.doi.org/10.1186/s13601-016-0124-9) |
| Camelo *et al.* 2015 | Associations of life course socioeconomic position and job stress with carotid intima-media thickness. The Brazilian Longitudinal Study of Adult Health (ELSA-Brasil) | Social Science & Medicine | Public, environmental, & occupational health | English | Brazil | 1 - in manuscript | [10.1016/j.socscimed.2015.07.032](http://www.doi.org/10.1016/j.socscimed.2015.07.032) |
| Camlin *et al.* 2016 | Patient-reported factors associated with reengagement among HIV-infected patients disengaged from care in East Africa | AIDS | Immunology; Infectious disease; Virology | English | USA | 0 | [10.1097/QAD.0000000000000931](http://www.doi.org/10.1097/QAD.0000000000000931) |
| Carlson *et al.* 2005 | Tractor-related injuries: A population-based study of a five-state region in the midwest | American Journal of Industrial Medicine | Public, environmental, & occupational health | English | USA | 1 - in manuscript | [10.1002/ajim.20135](http://www.doi.org/10.1002/ajim.20135) |
| Carlson *et al.* 2006 | The association between parents' past agricultural injuries and their children's risk of injury: Analyses from the regional rural injury study-II | Archives of Pediatrics and Adolescent Medicine | Pediatrics | English | USA | 0 | [10.1001/archpedi.160.11.1137](http://www.doi.org/10.1001/archpedi.160.11.1137) |
| Casas *et al.* 2015 | Exposure to bisphenol A during pregnancy and child neuropsychological development in the INMA-Sabadell cohort | Environmental Research | Environmental sciences; Public, environmental, & occupational health | English | Spain | 2 - in supplementary material | [10.1016/j.envres.2015.07.024](http://www.doi.org/10.1016/j.envres.2015.07.024) |
| Chandee *et al.* 2017 | Critical Care Resource Utilization and Outcomes of Children with Moderate Traumatic Brain Injury | Pediatric Critical Care Medicine | Critical care medicine; Pediatrics | English | USA | 1 - in supplementary material | [10.1097/PCC.0000000000001350](http://www.doi.org/10.1097/PCC.0000000000001350) |
| Chattopadhyay *et al.* 2003 | The New York State Minority Health Survey: determinants of oral health care utilization | Journal of public health dentistry | Dentistry, oral surgery & medicine; Public, environmental, & occupational health | English | USA | 1 - in manuscript | [10.1111/j.1752-7325.2003.tb03494.x](http://www.doi.org/10.1111/j.1752-7325.2003.tb03494.x) |
| Cupul-Uicab *et al.* 2013 | Prenatal exposure to persistent organochlorines and childhood obesity in the U.S. collaborative perinatal project | Environmental Health Perspectives | Environmental sciences; Public, environmental, & occupational health; Toxicology | English | USA | 0 | [10.1289/ehp.1205901](http://www.doi.org/10.1289/ehp.1205901) |
| Cupul-Uicab *et al.* 2014 | Prenatal exposure to p,p '-DDE and p,p '-DDT in relation to lower respiratory tract infections in boys from a highly exposed area of Mexico | Environmental Research | Environmental sciences; Public, environmental, & occupational health | English | USA | 0 | [10.1016/j.envres.2014.03.017](http://www.doi.org/10.1016/j.envres.2014.03.017) |
| Curran *et al.* 2016 | Obstetric mode of delivery and attention-deficit/hyperactivity disorder: A sibling-matched study | International Journal of Epidemiology | Public, environmental, & occupational health | English | Ireland | 1 - in supplementary material | [10.1093/ije/dyw001](http://www.doi.org/10.1093/ije/dyw001) |
| Curran *et al.* 2017 | Birth by caesarean section and school performance in Swedish adolescents- a population-based study | BMC Pregnancy and Childbirth | Obstetrics & Gynecology | English | Ireland | 1 - in supplementary material | [10.1186/s12884-017-1304-x](http://www.doi.org/10.1186/s12884-017-1304-x) |
| Dahl *et al.* 2012 | Plasma Fibulin-1 Is Linked to Restrictive Filling of the Left Ventricle and to Mortality in Patients With Aortic Valve Stenosis | Journal of the American Heart Association | Cardiac & Cardiovascular systems | English | Denmark | 1 - in supplementary material | [10.1161/JAHA.112.003889](http://www.doi.org/10.1161/JAHA.112.003889) |
| Dalgaard *et al.* 2016 | Risk and prognosis of bacteremia and fungemia among peritoneal dialysis patients: A population-based cohort study | Peritoneal Dialysis International | Urology & Nephrology | English | Denmark | 0 - links to www.dagitty.net are invalid | [10.2215/CJN.02320317](http://www.doi.org/10.2215/CJN.02320317) |
| de Jonge *et al.* 2014 | Determinants and consequences of short birth interval in rural Bangladesh: a cross-sectional study | BMC Pregnancy & Childbirth | Obstetrics & Gynecology | English | UK | 1 - in supplementary material | [10.1186/s12884-014-0427-6](http://www.doi.org/10.1186/s12884-014-0427-6) |
| di Giuseppe *et al.* 2015 | Plasma fibroblast growth factor 23 and risk of cardiovascular disease: results from the EPIC-Germany case-cohort study | European Journal of Epidemiology | Public, environmental, & occupational health | English | Germany | 1 - in manuscript | [10.1007/s10654-014-9982-4](http://www.doi.org/10.1007/s10654-014-9982-4) |
| Dolatowski *et al.* 2016 | Preoperative posterior tilt of at least 20° increased the risk of fixation failure in Garden-I and -II femoral neck fractures: 322 patients followed for a mean of 3 years | Acta Orthopaedica | Orthopedics | English | Norway | 0 | [10.3109/17453674.2016.1155253](http://www.doi.org/10.3109/17453674.2016.1155253) |
| Downes *et al.* 2015 | Previous prelabor or intrapartum cesarean delivery and risk of placenta previa | American Journal of Obstetrics and Gynecology | Obstetrics & Gynecology | English | USA | 0 | [10.1016/j.ajog.2015.01.004](http://www.doi.org/10.1016/j.ajog.2015.01.004) |
| Dusingize *et al.* 2017 | Cigarette Smoking and the Risks of Basal Cell Carcinoma and Squamous Cell Carcinoma | Journal of Investigative Dermatology | Dermatology | English | Australia | 0 | [10.1016/j.jid.2017.03.027](http://www.doi.org/10.1016/j.jid.2017.03.027) |
| Dzhambov & Dimitrova 2015 | Road traffic noise exposure association with self-reported body mass index | Noise Control Engineering Journal | Acoustics; Engineering, multidisciplinary | English | Bulgaria | 0 | [10.3397/1/376351](http://www.doi.org/10.3397/1/376351) |
| Dzhambov & Dimitrova 2016 | Heart disease attributed to occupational noise, vibration and other co-exposure: Self-reported population-based survey among Bulgarian workers | Medycyna Pracy | Public, environmental, & occupational health; | English | Bulgaria | 0 | [10.13075/mp.5893.00437](http://www.doi.org/10.13075/mp.5893.00437) |
| Eichler *et al.* 2016 | Tobacco and alcohol consumption after total laryngectomy and survival: A German multicenter prospective cohort study | Head and Neck | Otorhinolaryngology; | English | Germany | 0 | [10.1002/hed.24436](http://www.doi.org/10.1002/hed.24436) |
| Ellison & De Wet 2016 | Poverty, disability and self-reported health amongst residents and migrants in Gauteng, South Africa | Annals of Human Biology | Biology; Public, environmental, & occupational health; | English | UK | 1 - in manuscript | [10.3109/03014460.2016.1147597](http://www.doi.org/10.3109/03014460.2016.1147597) |
| Emeny *et al.* 2014 | Mind over hormones; Sex differences in associations of well-being with IGF-I, IGFBP-3 and physical activity in the KORA-Age study | Experimental Gerontology | Geriatrics & Gerontology; | English | Germany | 1 - in supplementary material | [10.1016/j.exger.2014.08.001](http://www.doi.org/10.1016/j.exger.2014.08.001) |
| Erkal *et al.* 2009 | Horse-related injuries among agricultural household members: Regional rural injury study II (RRIS-II) | Journal of Rural Health | Health care sciences & services; Public, environmental, & occupational health; | English | USA | 2 - in supplementary material | [10.1111/j.1748-0361.2009.00254.x](http://www.doi.org/10.1111/j.1748-0361.2009.00254.x) |
| Escobar *et al.* 2017 | Risk of Acute Kidney Injury after Percutaneous Pharmacomechanical Thrombectomy Using AngioJet in Venous and Arterial Thrombosis | Annals of Vascular Surgery | Surgery; Peripheral vascular disease; | English | USA | 1 - in manuscript | [10.1016/j.avsg.2016.12.018](http://www.doi.org/10.1016/j.avsg.2016.12.018) |
| Evandt *et al.* 2017 | A population-based study on nighttime road traffic noise and insomnia | Sleep | Not indexed | English | Norway | 1 - in manuscript | [10.1093/sleep/zsw055](http://www.doi.org/10.1093/sleep/zsw055) |
| Evandt *et al.* 2017A | Road traffic noise and registry based use of sleep medication | Environmental Health: A Global Access Science Source | Environmental sciences; Public, environmental, & occupational health; | English | Norway | 2 - on www.dagitty.net | [10.1186/s12940-017-0330-5](http://www.doi.org/10.1186/s12940-017-0330-5) |
| Fan *et al.* 2016 | Alcohol drinking and HIV-related risk among men who have sex with men in Chongqing, China | Alcohol | Substance Abuse; Pharmacology & Pharmacy; Toxicology | English | USA | 0 | [10.1016/j.alcohol.2015.09.004](http://www.doi.org/10.1016/j.alcohol.2015.09.004) |
| Feda *et al.* 2010 | Written violence policies and risk of physical assault against Minnesota educators | Journal of Public Health Policy | Health care sciences & services; Public, environmental, & occupational health; | English | USA | 1 - in manuscript | [10.1057/jphp.2010.32](http://www.doi.org/10.1057/jphp.2010.32) |
| Ferraro *et al.* 2017 | Contributions of relative linear growth and adiposity accretion from birth to adulthood to adult hypertension | Scientific Reports | Not indexed | English | Brazil | 1 - in supplementary material | [10.1038/s41598-017-09027-1](http://www.doi.org/10.1038/s41598-017-09027-1) |
| Ferraro *et al.* 2017A | The specific and combined role of domestic violence and mental health disorders during pregnancy on new-born health | Bmc Pregnancy and Childbirth | Obstetrics & Gynecology; | English | Brazil | 0 | [10.1186/s12884-017-1438-x](http://www.doi.org/10.1186/s12884-017-1438-x) |
| Figueiredo *et al.* 2017 | Association between plasma concentrations of vitamin D metabolites and depressive symptoms throughout pregnancy in a prospective cohort of Brazilian women | Journal of Psychiatric Research | Psychiatry; | English | Brazil | 1 - in supplementary material | [10.1016/j.jpsychires.2017.07.009](http://www.doi.org/10.1016/j.jpsychires.2017.07.009) |
| Filippidis *et al.* 2017 | Association of cigarette price differentials with infant mortality in 23 European Union countries | JAMA Pediatrics | Pediatrics; | English | UK | 1 - in supplementary material | [10.1001/jamapediatrics.2017.2536](http://www.doi.org/10.1001/jamapediatrics.2017.2536) |
| Filteau *et al.* 2016 | Associations of Vitamin D status, bone health and anthropometry, with gross motor development and performance of school-aged Indian children who were born at term with low birth weight | BMJ Open | Medicine, general, & internal; | English | UK | 1 - in manuscript | [10.1136/bmjopen-2015-009268](http://www.doi.org/10.1136/bmjopen-2015-009268) |
| Frederiksen *et al.* 2009 | Do patient characteristics, disease, or treatment explain social inequality in survival from colorectal cancer? | Social Science & Medicine | Public, environmental, & occupational health; | English | Denmark | 3 - in manuscript | [10.1016/j.socscimed.2009.07.040](http://www.doi.org/10.1016/j.socscimed.2009.07.040) |
| Gagliardi *et al.* 2009 | Bronchopulmonary dysplasia and brain white matter damage in the preterm infant: A complex relationship | Paediatric and Perinatal Epidemiology | Public, environmental, & occupational health; Obstetrics & Gynecology; Pediatrics | English | Italy | 2 - in manuscript | [10.1111/j.1365-3016.2009.01069.x](http://www.doi.org/10.1111/j.1365-3016.2009.01069.x) |
| Gascon *et al.* 2015 | Prenatal exposure to bisphenol A and phthalates and childhood respiratory tract infections and allergy | Journal of Allergy and Clinical Immunology | Allergy; Immunology; | English | Spain | 2 - in supplementary material | [10.1016/j.jaci.2014.09.030](http://www.doi.org/10.1016/j.jaci.2014.09.030) |
| Gaspar *et al.* 2017 | Levels and Determinants of DDT and DDE Exposure in the VHEMBE Cohort | Environmental Health Perspectives | Environmental sciences; Public, environmental, & occupational health; Toxicology | English | USA | 1 - in supplementary material | [10.1289/EHP353](http://www.doi.org/10.1289/EHP353) |
| Gerberich *et al.* 2011 | Violence against educators: A population-based study | Journal of Occupational and Environmental Medicine | Public, environmental, & occupational health; | English | USA | 0 - manuscript contains incorrect figure | [10.1097/JOM.0b013e31820c3fa1](http://www.doi.org/10.1097/JOM.0b013e31820c3fa1) |
| Gerberich *et al.* 2014 | Case-control study of student-perpetrated physical violence against educators | Annals of Epidemiology | Public, environmental, & occupational health; | English | USA | 0 | [10.1016/j.annepidem.2014.02.006](http://www.doi.org/10.1016/j.annepidem.2014.02.006) |
| Gillam *et al.* 2017 | Heart failure after conventional metal-on-metal hip replacements: A retrospective cohort study | Acta Orthopaedica | Orthopedics; | English | Australia | 0 | [10.1080/17453674.2016.1246276](http://www.doi.org/10.1080/17453674.2016.1246276) |
| Gillott *et al.* 2017 | South Asian ethnicity is associated with a lower prevalence of atrial fibrillation despite greater prevalence of established risk factors: A population-based study in Bradford Metropolitan District | Europace | Cardiac & Cardiovascular systems; | English | UK | 0 | [10.1093/europace/euw010](http://www.doi.org/10.1093/europace/euw010) |
| Gocke *et al.* 2014 | Abdominal obesity modifies long-term associations between periodontitis and markers of systemic inflammation | Atherosclerosis | Peripheral Vascular Disease; | English | Germany | 1 - in supplementary material | [10.1016/j.atherosclerosis.2014.05.926](http://www.doi.org/10.1016/j.atherosclerosis.2014.05.926) |
| Gray *et al.* 2017 | Determinants of early-life lung function in African infants | Thorax |  | English | South Africa | 1 - in supplementary material | [10.1136/thoraxjnl-2015-207401](http://www.doi.org/10.1136/thoraxjnl-2015-207401) |
| Gray *et al.* 2017A | Lung Function in African Infants in the Drakenstein Child Health Study | American Journal of Respiratory and Critical Care Medicine | Critical care medicine; Respiratory system; | English | South Africa | 0 | [10.1164/rccm.201601-0188OC](http://www.doi.org/10.1164/rccm.201601-0188OC) |
| Grice *et al.* 2007 | Giving Birth and Returning to Work: The Impact of Work-Family Conflict on Women's Health After Childbirth | Annals of Epidemiology | Public, environmental, & occupational health; | English | USA | 3 - in supplementary material | [10.1016/j.annepidem.2007.05.002](http://www.doi.org/10.1016/j.annepidem.2007.05.002) |
| Grice *et al.* 2010 | Past violence and future work-related violence: A case-control study of Minnesota nurses | Italian Journal of Public Health | Not indexed | English | USA | 2 - in supplementary material | [10.2427/5727](http://www.doi.org/10.2427/5727) |
| Grill *et al.* 2014 | Self-Efficacy Beliefs Are Associated with Visual Height Intolerance: A Cross-Sectional Survey | PLoS One | Multidisciplinary sciences; | English | Germany | 1 - in manuscript | [10.1371/journal.pone.0116220](http://www.doi.org/10.1371/journal.pone.0116220) |
| Grundy *et al.* 2017 | Rotating shift work associated with obesity in men from Northeastern Ontario | Health Promotion and Chronic Disease Prevention in Canada | Public, environmental, & occupational health; | English | Canada | 1 - in manuscript | [10.24095/hpcdp.37.8.02](http://www.doi.org/10.24095/hpcdp.37.8.02) |
| Guimarães *et al.* 2016 | Social mobility and subclinical atherosclerosis in a middle-income country: Association of intra-and inter-generational social mobility with carotid intima-media thickness in the Brazilian Longitudinal Study of Adult Health (ELSA-Brasil) | Social Science & Medicine | Public, environmental, & occupational health; | English | Brazil | 1 - in manuscript | [10.1016/j.socscimed.2016.09.021](http://www.doi.org/10.1016/j.socscimed.2016.09.021) |
| Gunathilake *et al.* 2016 | The association between obesity and cognitive function in older persons: How much is mediated by inflammation, fasting plasma glucose, and hypertriglyceridemia? | Journals of Gerontology - Series A Biological Sciences and Medical Sciences | Geriatrics & Gerontology; | English | Australia | 1 - in supplementary material | [10.1093/gerona/glw070](http://www.doi.org/10.1093/gerona/glw070) |
| Harris-Adamson *et al.* 2016 | Biomechanical and psychosocial exposures are independent risk factors for carpal tunnel syndrome: Assessment of confounding using causal diagrams | Occupational and Environmental Medicine | Public, environmental, & occupational health; | English | USA | 2 - in manuscript | [10.1136/oemed-2016-103634](http://www.doi.org/10.1136/oemed-2016-103634) |
| Harskamp-van Ginkel *et al.* 2015 | A Study on Mediation by Offspring BMI in the Association between Maternal Obesity and Child Respiratory Outcomes in the Amsterdam Born and Their Development Study Cohort | PLoS One | Multidisciplinary sciences; | English | Netherlands | 1 - in supplementary material | [10.1371/journal.pone.0140641](http://www.doi.org/10.1371/journal.pone.0140641) |
| Harville *et al.* 2017 | Indicators of fetal growth and adult liver enzymes: the Bogalusa Heart Study and the Cardiovascular Risk in Young Finns Study | Journal of Developmental Origins of Health and Disease | Public, environmental, & occupational health; | English | USA | 0 | [10.1017/S2040174416000635](http://www.doi.org/10.1017/S2040174416000635) |
| Herich *et al.* 2017 | Maternal Education Is Associated with Disparities in Breastfeeding at Time of Discharge but Not at Initiation of Enteral Feeding in the Neonatal Intensive Care Unit | Journal of Pediatrics | Pediatrics; | English | Italy | 1 - in manuscript | [10.1016/j.jpeds.2016.10.046](http://www.doi.org/10.1016/j.jpeds.2016.10.046) |
| Hickson *et al.* 2017 | Mental health inequalities among gay and bisexual men in England, Scotland and Wales: A large community-based cross-sectional survey | Journal of Public Health (United Kingdom) | Public, environmental, & occupational health; | English | UK | 1 - in manuscript | [10.1093/pubmed/fdw021](http://www.doi.org/10.1093/pubmed/fdw021) |
| Hinkle *et al.* 2013 | Maternal prepregnancy weight status and associations with children's development and disabilities at kindergarten | International Journal of Obesity | Endocrinology & metabolism; Nutrition & dietetics; | English | USA | 1 - in manuscript | [10.1038/ijo.2013.128](http://www.doi.org/10.1038/ijo.2013.128) |
| Hirsch *et al.* 2016 | Destinations That Older Adults Experience Within Their GPS Activity Spaces: Relation to Objectively Measured Physical Activity | Environment and Behavior | Environmental sciences; psychology; multidisciplinary | English | USA | 0 | [10.1177/0013916515607312](http://www.doi.org/10.1177/0013916515607312) |
| Hirsch *et al.* 2017 | The influence of walkability on broader mobility for Canadian middle aged and older adults: An examination of Walk Score™ and the Mobility Over Varied Environments Scale (MOVES) | Preventive Medicine | Public, environmental, & occupational health; Occupational health; Medicine, general & internal | English | USA + Canada | 0 | [10.1016/j.ypmed.2016.09.036](http://www.doi.org/10.1016/j.ypmed.2016.09.036) |
| Hoyer *et al.* 2015 | Anthropometry in 5-to 9-Year-Old Greenlandic and Ukrainian Children in Relation to Prenatal Exposure to Perfluorinated Alkyl Substances | Environmental Health Perspectives | Environmental sciences; Public, environmental, & occupational health; Toxicology | English | Denmark | 0 | [10.1289/ehp.1408881](http://www.doi.org/10.1289/ehp.1408881) |
| Jacobson *et al.* 2017 | Growth at 2 years of age in HIV-exposed uninfected children in the United States by trimester of maternal antiretroviral initiation | Pediatric Infectious Disease Journal | Immunology; Infectious disease; Pediatrics | English | USA | 0 | [10.1097/INF.0000000000001387](http://www.doi.org/10.1097/INF.0000000000001387) |
| Jämsä *et al.* 2017 | Risk factors associated with acute kidney injury in a cohort of 20,575 arthroplasty patients | Acta Orthopaedica | Orthopedics; | English | Finland | 1 - in supplementary material | [10.1080/17453674.2017.1301743](http://www.doi.org/10.1080/17453674.2017.1301743) |
| Janitz *et al.* 2017 | Benzene and childhood acute leukemia in Oklahoma | Environmental Research | Environmental sciences; Public, environmental, & occupational health; | English | USA | 0 | [10.1016/j.envres.2017.06.015](http://www.doi.org/10.1016/j.envres.2017.06.015) |
| Jankowiak *et al.* 2016 | Current and cumulative night shift work and subclinical atherosclerosis: results of the Gutenberg Health Study | International Archives of Occupational and Environmental Health | Public, environmental, & occupational health; | English | Germany | 0 | [10.1007/s00420-016-1150-6](http://www.doi.org/10.1007/s00420-016-1150-6) |
| Johri *et al.* 2016 | Maternal Health Literacy Is Associated with Early Childhood Nutritional Status in India | Journal of Nutrition | Nutrition & dietetics; | English | Canada | 2 - in supplementary material | [10.3945/jn.115.226290](http://www.doi.org/10.3945/jn.115.226290) |
| Jonker *et al.* 2012 | Iron status predicts malaria risk in Malawian preschool children | PLoS One | Multidisciplinary sciences; | English | Netherlands | 1 - in manuscript | [10.1371/journal.pone.0042670](http://www.doi.org/10.1371/journal.pone.0042670) |
| Jusko *et al.* 2010 | A cohort study of developmental polychlorinated biphenyl (PCB) exposure in relation to post-vaccination antibody response at 6-months of age | Environmental Research | Environmental sciences; Public, environmental, & occupational health; | English | USA | 0 | [10.1016/j.envres.2010.02.010](http://www.doi.org/10.1016/j.envres.2010.02.010) |
| Kalapatapu *et al.* 2017 | Does trauma exposure predict prescription drug problems beyond the contribution of post-traumatic stress disorder and depression? An analysis of the Mind Your Heart cohort study | Journal of Addictive Diseases | Substance Abuse; | English | USA | 1 - in manuscript | [10.1080/10550887.2017.1314697](http://www.doi.org/10.1080/10550887.2017.1314697) |
| Kalapatapu *et al.* 2017A | Lifetime alcohol use and cognitive performance in older adults | Journal of Addictive Diseases | Substance Abuse; | English | USA | 1 - in manuscript | [10.1080/10550887.2016.1245029](http://www.doi.org/10.1080/10550887.2016.1245029) |
| Karahalios *et al.* 2014 | Change in Body Size and Mortality: Results from the Melbourne Collaborative Cohort Study | PLoS One | Multidisciplinary sciences; | English | Australia | 1 - in supplementary material | [10.1371/journal.pone.0099672](http://www.doi.org/10.1371/journal.pone.0099672) |
| Karahalios *et al.* 2016 | Change in weight and waist circumference and risk of colorectal cancer: Results from the Melbourne Collaborative Cohort Study | BMC Cancer | Oncology; | English | Australia | 1 - in supplementary material | [10.1186/s12885-016-2144-1](http://www.doi.org/10.1186/s12885-016-2144-1) |
| Karlsen *et al.* 2017 | Early-life exposures to persistent organic pollutants in relation to overweight in preschool children | Reproductive Toxicology | Reproductive Biology; Toxicology; | English | USA | 0 | [10.1016/j.reprotox.2016.08.002](http://www.doi.org/10.1016/j.reprotox.2016.08.002) |
| Kebede *et al.* 2017 | Association of Periodontal Destruction and Diabetes with Mortality | Journal of Dental Research | Journal of Dental Research; | English | Germany | 1 - in supplementary material | [10.1177/0022034516668839](http://www.doi.org/10.1177/0022034516668839) |
| Kendrick *et al.* 2016 | Risk and protective factors for falls on stairs in young children: Multicentre case-control study | Archives of Disease in Childhood | Pediatrics; | English | UK | 0 | [10.1136/archdischild-2015-308486](http://www.doi.org/10.1136/archdischild-2015-308486) |
| Kerschberger *et al.* 2012 | The Effect of Complete Integration of HIV and TB Services on Time to Initiation of Antiretroviral Therapy: A Before-After Study | PLoS One | Multidisciplinary sciences; | English | South Africa | 4 - in supplementary material | [10.1371/journal.pone.0046988](http://www.doi.org/10.1371/journal.pone.0046988) |
| Khalifa *et al.* 2016 | Determinants of postnatal depression in Sudanese women at 3 months postpartum: A cross-sectional study | BMJ Open | Medicine, general, & internal; | English | Norway | 2 - in manuscript | [10.1136/bmjopen-2015-009443](http://www.doi.org/10.1136/bmjopen-2015-009443) |
| Kharmats *et al.* 2014 | Relation between the Supplemental Nutritional Assistance Program cycle and dietary quality in low-income African Americans in Baltimore, Maryland | American Journal of Clinical Nutrition | Nutrition & dietetics; | English | USA | 1 - in supplementary material | [10.3945/ajcn.113.075994](http://www.doi.org/10.3945/ajcn.113.075994) |
| Kim *et al.* 2013 | Association between Urinary Prostaglandin E-2 Metabolite and Breast Cancer Risk: A Prospective, Case-Cohort Study of Postmenopausal Women | Cancer Prevention Research | Oncology; | English | USA | 0 | [10.1158/1940-6207.CAPR-13-0040](http://www.doi.org/10.1158/1940-6207.CAPR-13-0040) |
| Kim *et al.* 2015 | Fruit and vegetable intake and urinary levels of prostaglandin E2 metabolite in postmenopausal women | Nutrition and Cancer | Oncology; Nutrition & dietetics; | English | USA | 0 | [10.1080/01635581.2015.1011787](http://www.doi.org/10.1080/01635581.2015.1011787) |
| Klassen *et al.* 2014 | Cardiorespiratory fitness in breast cancer patients undergoing adjuvant therapy | Acta Oncologica | Oncology; | English | Germany | 0 | [10.3109/0284186X.2014.899435](http://www.doi.org/10.3109/0284186X.2014.899435) |
| Kobayashi *et al.* 2017 | Effects of prenatal perfluoroalkyl acid exposure on cord blood IGF2/H19 methylation and ponderal index: The hokkaido study | Journal of Exposure Science and Environmental Epidemiology | Environmental sciences; Public, environmental, & occupational health; Toxicology | English | Japan | 0 | [10.1038/jes.2016.50](http://www.doi.org/10.1038/jes.2016.50) |
| Kowall *et al.* 2016 | Associations between sleep characteristics and weight gain in an older population: results of the Heinz Nixdorf Recall Study | Nutrition & Diabetes | Endocrinology & metabolism; Nutrition & dietetics; | English | Germany | 0 | [10.1038/nutd.2016.32](http://www.doi.org/10.1038/nutd.2016.32) |
| Kowall *et al.* 2016A | Associations among sleep disturbances, nocturnal sleep duration, daytime napping, and incident prediabetes and type 2 diabetes: the Heinz Nixdorf Recall Study | Sleep Medicine | Clinical neurology; | English | Germany | 0 | [10.1016/j.sleep.2015.12.017](http://www.doi.org/10.1016/j.sleep.2015.12.017) |
| Kroenke *et al.* 2016 | Analysis of body mass index and mortality in patients with colorectal cancer using causal diagrams | JAMA Oncology | Oncology; | English | USA | 1 - in supplementary material | [10.1001/jamaoncol.2016.0732](http://www.doi.org/10.1001/jamaoncol.2016.0732) |
| Kverneng Hultberg *et al.* 2017 | Nonsteroidal anti-inflammatory drugs and the risk of anastomotic leakage after anterior resection for rectal cancer | European Journal of Surgical Oncology | Oncology; Surgery; | English | Sweden | 1 - in supplementary material | [10.1016/j.ejso.2017.06.010](http://www.doi.org/10.1016/j.ejso.2017.06.010) |
| Lai *et al.* 2016 | Ambient air pollution and risk of tuberculosis: A cohort study | Occupational and Environmental Medicine | Public, environmental, & occupational health; | English | Taiwan | 1 - in supplementary material | [10.1136/oemed-2015-102995](http://www.doi.org/10.1136/oemed-2015-102995) |
| Launay *et al.* 2014 | Why Children with Severe Bacterial Infection Die: A Population-Based Study of Determinants and Consequences of Suboptimal Care with a Special Emphasis on Methodological Issues | PLoS One | Multidisciplinary sciences; | English | France | 3 - in supplementary material | [10.1371/journal.pone.0107286](http://www.doi.org/10.1371/journal.pone.0107286) |
| Lehnich *et al.* 2016 | Do patients with intake of drugs labelled as sleep disturbing really sleep worse? A population based assessment from the Heinz Nixdorf Recall Study | British Journal of Clinical Pharmacology | Pharmacology & Pharmacy; | English | Germany | 0 | [10.1111/bcp.13015](http://www.doi.org/10.1111/bcp.13015) |
| Liang *et al.* 2017 | Association between Cesarean Section and Weight Status in Chinese Children and Adolescents: A National Survey | International Journal of Environmental Research and Public Health | Environmental sciences; Public, environmental, & occupational health; | English | China | 1 - in supplementary material | [10.3390/ijerph14121609](http://www.doi.org/10.3390/ijerph14121609) |
| Liebers *et al.* 2011 | Causal Graph (Directed Acyclic Graph – DAG) of a Secondary Data Analysis Regarding Sickness Absence due to Musculoskeletal Disorders | Gesundheitswesen | Public, environmental, & occupational health | German | Germany | 1 – in manuscript | [10.1055/s-0031-1291191](http://www.doi.org/10.1055/s-0031-1291191) |
| Lima *et al.* 2017 | Association between maternal depressive symptoms with child malnutrition or child excess weight | Revista Brasileira de Saude Materno Infantil | Not indexed | English | Brazil | 2 - in manuscript | [10.1590/1806-93042017000300010](http://www.doi.org/10.1590/1806-93042017000300010) |
| Linde *et al.* 2017 | The relation between given volume and heart rate during newborn resuscitation | Resuscitation | Not indexed | English | Norway | 0 | [10.1016/j.resuscitation.2017.06.007](http://www.doi.org/10.1016/j.resuscitation.2017.06.007) |
| Lupattelli *et al.* 2015 | Medication Use before, during, and after Pregnancy among Women with Eating Disorders: A Study from the Norwegian Mother and Child Cohort Study | PLoS One | Multidisciplinary sciences; | English | Norway | 0 | [10.1371/journal.pone.0133045](http://www.doi.org/10.1371/journal.pone.0133045) |
| Lytsy *et al.* 2013 | Endothelial function and risk of hypertension and blood pressure progression: The prospective investigation of the vasculature in Uppsala seniors | Journal of Hypertension | Peripheral Vascular Disease; | English | Sweden | 0 | [10.1097/HJH.0b013e32835ed5a0](http://www.doi.org/10.1097/HJH.0b013e32835ed5a0) |
| Magadi & Magadi 2017 | HIV/AIDS and contraceptive use: factors associated with contraceptive use among sexually active HIV-positive women in Kenya | Contraception | Obstetrics & Gynecology; | English | UK | 1 - in manuscript | [10.1016/j.contraception.2016.10.013](http://www.doi.org/10.1016/j.contraception.2016.10.013) |
| Maier *et al.* 2015 | Effect of Beta-Blocker Therapy on the Risk of Infections and Death after Acute Stroke - A Historical Cohort Study | PLoS One | Multidisciplinary sciences; | English | Germany | 0 | [10.1371/journal.pone.0116836](http://www.doi.org/10.1371/journal.pone.0116836) |
| Maika *et al.* 2015 | Effect on child cognitive function of increasing household expenditure in Indonesia: Application of a marginal structural model and simulation of a cash transfer programme | International Journal of Epidemiology | Public, environmental, & occupational health; | English | Australia | 1 - in manuscript | [10.1093/ije/dyu264](http://www.doi.org/10.1093/ije/dyu264) |
| Maretty-Nielsen *et al.* 2014 | Relative mortality in soft tissue sarcoma patients: A Danish population-based cohort study | BMC Cancer | Oncology; | English | Denmark | 1 - in manuscript | [10.1186/1471-2407-14-682](http://www.doi.org/10.1186/1471-2407-14-682) |
| Maretty-Nielsen *et al.* 2014A | Prognostic factors for local recurrence and mortality in adult soft tissue sarcoma of the extremities and trunk wall | Acta Orthopaedica | Orthopedics; | English | Denmark | 1 - in supplementary material | [10.3109/17453674.2014.908341](http://www.doi.org/10.3109/17453674.2014.908341) |
| Martin *et al.* 2017 | Racial disparities in the utilization of preventive health services among older women with early-stage endometrial cancer enrolled in Medicare | Cancer Medicine | Oncology; | English | USA | 1 - in supplementary material | [10.1002/cam4.1141](http://www.doi.org/10.1002/cam4.1141) |
| Matser *et al.* 2013 | Higher Chlamydia trachomatis Prevalence in Ethnic Minorities Does Not Always Reflect Higher Sexual Risk Behaviour | PLoS One | Multidisciplinary sciences; | English | Netherlands | 1 - in manuscript | [10.1371/journal.pone.0067287](http://www.doi.org/10.1371/journal.pone.0067287) |
| McCulloh *et al.* 2015 | Evaluating the Use of Blood Cultures in the Management of Children Hospitalized for Community-Acquired Pneumonia | PLoS One | Multidisciplinary sciences; | English | USA | 0 | [10.1371/journal.pone.0117462](http://www.doi.org/10.1371/journal.pone.0117462) |
| Mebrahtu *et al.* 2015 | Effects of birth weight and growth on childhood wheezing disorders: findings from the Born in Bradford Cohort | Bmj Open | Medicine, general, & internal; | English | UK | 2 - in supplementary material | [10.1136/bmjopen-2015-009553](http://www.doi.org/10.1136/bmjopen-2015-009553) |
| Medenwald *et al.* 2014 | Inflammation and prolonged QT time: Results from the Cardiovascular Disease, Living and Ageing in Halle (CARLA) study | PLoS One | Multidisciplinary sciences; | English | Netherlands | 1 - in supplementary material | [10.1371/journal.pone.0095994](http://www.doi.org/10.1371/journal.pone.0095994) |
| Medenwald *et al.* 2015 | Educational level and chronic inflammation in the elderly - the role of obesity: results from the population-based CARLA study | Clinical Obesity | Not indexed | English | Germany | 1 - in manuscript | [10.1111/cob.12107](http://www.doi.org/10.1111/cob.12107) |
| Medenwald *et al.* 2016 | QT interval, general mortality and the role of echocardiographic parameters of left ventricular hypertrophy: Results from the prospective, population-based CARLA study | European Journal of Preventive Cardiology | Cardiac & Cardiovascular systems; | English | Germany | 0 | [10.1177/2047487315587271](http://www.doi.org/10.1177/2047487315587271) |
| Medenwald *et al.* 2016A | Educational status and differences in left ventricular mass and ejection fraction - The role of BMI and parameters related to the metabolic syndrome: A longitudinal analysis from the population-based CARLA cohort | Nutrition Metabolism and Cardiovascular Diseases | Cardiac & Cardiovascular systems; Endocrinology & metabolism; Nutrition & dietetics | English | Germany | 1 - in manuscript | [10.1016/j.numecd.2016.05.001](http://www.doi.org/10.1016/j.numecd.2016.05.001) |
| Messerlian *et al.* 2017 | Paternal and maternal preconception urinary phthalate metabolite concentrations and child behavior | Environmental Research | Environmental sciences; Public, environmental, & occupational health; | English | USA | 1 - in supplementary material | [10.1016/j.envres.2017.07.032](http://www.doi.org/10.1016/j.envres.2017.07.032) |
| Møller *et al.* 2016 | Multi-wave cohort study of sedentary work and risk of ischemic heart disease | Scandinavian Journal of Work, Environment and Health |  | English | Denmark | 1 - in manuscript | [10.5271/sjweh.3540](http://www.doi.org/10.5271/sjweh.3540) |
| Mook *et al.* 2016 | Food security status and barriers to fruit and vegetable consumption in two economically deprived communities of Oakland, California, 2013-2014 | Preventing Chronic Disease | Public, environmental, & occupational health; | English | USA | 0 | [10.5888/pcd13.150402](http://www.doi.org/10.5888/pcd13.150402) |
| Murphy *et al.* 2015 | Racial differences in obesity measures and risk of colorectal adenomas in a large screening population | Nutrition and Cancer | Oncology; Nutrition & dietetics; | English | USA | 0 | [10.1080/01635581.2015.976316](http://www.doi.org/10.1080/01635581.2015.976316) |
| Napier *et al.* 2017 | Exposure to human-associated fecal indicators and self-reported illness among swimmers at recreational beaches: A cohort study | Environmental Health: A Global Access Science Source | Environmental sciences; Public, environmental, & occupational health; | English | USA | 0 | [10.1186/s12940-017-0308-3](http://www.doi.org/10.1186/s12940-017-0308-3) |
| Ng *et al.* 2017 | The relationship between weight change and daytime sleepiness: the Sleep Heart Health Study | Sleep Medicine | Not indexed | English | Australia | 1 - in supplementary material | [10.1016/j.sleep.2017.05.004](http://www.doi.org/10.1016/j.sleep.2017.05.004) |
| Ng *et al.* 2017A | Change in Use of Sleep Medications After Gastric Bypass Surgery or Intensive Lifestyle Treatment in Adults with Obesity | Obesity | Endocrinology & metabolism; Nutrition & dietetics; | English | Sweden | 1 - in supplementary material | [10.1002/oby.21908](http://www.doi.org/10.1002/oby.21908) |
| Ngueta *et al.* 2016 | Use of a cumulative exposure index to estimate the impact of tap water lead concentration on blood lead levels in 1- to 5-year-old children (Montréal, Canada) | Environmental Health Perspectives | Environmental sciences; Public, environmental, & occupational health; Toxicology | English | Canada | 1 - in supplementary material | [10.1289/ehp.1409144](http://www.doi.org/10.1289/ehp.1409144) |
| Nobre *et al.* 2016 | Association between maternal lifestyle and preschool nutrition | Revista da Associacao Medica Brasileira | Medicine, general, & internal; | English | Brazil | 0 | [10.1590/1806-9282.62.06.494](http://www.doi.org/10.1590/1806-9282.62.06.494) |
| Norris *et al.* 2017 | Do changing levels of maternal exercise during pregnancy affect neonatal adiposity? Secondary analysis of the babies after SCOPE: evaluating the longitudinal impact using neurological and nutritional endpoints (BASELINE) birth cohort (Cork, Ireland) | Bmj Open | Medicine, general, & internal; | English | UK | 1 - in supplementary material | [10.1136/bmjopen-2017-017987](http://www.doi.org/10.1136/bmjopen-2017-017987) |
| Nourbakhsh *et al.* 2016 | Longitudinal associations between brain structural changes and fatigue in early MS | Multiple Sclerosis and Related Disorders | Clinical neurology; | English | USA | 1 - in manuscript | [10.1016/j.msard.2015.10.006](http://www.doi.org/10.1016/j.msard.2015.10.006) |
| Nowak *et al.* 2016 | Prevalence of Anal High-Risk Human Papillomavirus Infections Among HIV-Positive and HIV-Negative Men Who Have Sex With Men in Nigeria | Sexually Transmitted Diseases | Infectious disease; | English | USA | 1 - in supplementary material | [10.1097/OLQ.0000000000000431](http://www.doi.org/10.1097/OLQ.0000000000000431) |
| Nygaard *et al.* 2014 | Lifetime physical activity and pelvic organ prolapse in middle-aged women | American Journal of Obstetrics and Gynecology | Obstetrics & Gynecology; | English | USA | 0 | [10.1016/j.ajog.2014.01.035](http://www.doi.org/10.1016/j.ajog.2014.01.035) |
| Nygaard *et al.* 2015 | Lifetime physical activity and female stress urinary incontinence | American Journal of Obstetrics and Gynecology | Obstetrics & Gynecology; | English | USA | 0 | [10.1016/j.ajog.2015.01.044](http://www.doi.org/10.1016/j.ajog.2015.01.044) |
| Oddo *et al.* 2017 | The weight of work: The association between maternal employment and overweight in low- and middle-income countries | International Journal of Behavioral Nutrition and Physical Activity | Nutrition & dietetics; Physiology; | English | USA | 0 | [10.1186/s12966-017-0522-y](http://www.doi.org/10.1186/s12966-017-0522-y) |
| Oddo *et al.* 2017A | Maternal employment and childhood overweight in low- and middle-income countries | Public Health Nutrition | Public, environmental, & occupational health; Occupational health; Nutrition & dietetics | English | USA | 1 - in manuscript | [10.1017/S1368980017001720](http://www.doi.org/10.1017/S1368980017001720) |
| Olsen *et al.* 2015 | Socioeconomic position and stage at diagnosis of head and neck cancer - A nationwide study from DAHANCA | Acta Oncologica | Oncology; | English | Denmark | 1 - in manuscript | [10.3109/0284186X.2014.998279](http://www.doi.org/10.3109/0284186X.2014.998279) |
| Olsson *et al.* 2017 | Vitamin D is not associated with incident dementia or cognitive impairment: An 18-y follow-up study in community-living old men | American Journal of Clinical Nutrition | Nutrition & dietetics; | English | Sweden | 0 | [10.3945/ajcn.116.141531](http://www.doi.org/10.3945/ajcn.116.141531) |
| O'Neill *et al.* 2016 | Birth by caesarean section and the risk of adult psychosis: A population-based cohort study | Schizophrenia Bulletin | Psychiatry; | English | Ireland | 0 - supplementary material unavailable | [10.1093/schbul/sbv152](http://www.doi.org/10.1093/schbul/sbv152) |
| Orban *et al.* 2016 | Residential Road Traffic Noise and High Depressive Symptoms after Five Years of Follow-up: Results from the Heinz Nixdorf Recall Study | Environmental Health Perspectives | Environmental sciences; Public, environmental, & occupational health; Toxicology | English | Germany | 1 - in supplementary material | [10.1289/ehp.1409400](http://www.doi.org/10.1289/ehp.1409400) |
| Osler *et al.* 2015 | The Impact of Comorbid Depression on Educational Inequality in Survival after Acute Coronary Syndrome in a Cohort of 83 062 Patients and a Matched Reference Population | PLoS One | Multidisciplinary sciences; | English | Denmark | 1 - in manuscript | [10.1371/journal.pone.0141598](http://www.doi.org/10.1371/journal.pone.0141598) |
| Oswald *et al.* 2017 | Active trachoma and community use of sanitation, Ethiopia | Bulletin of the World Health Organization | Public, environmental, & occupational health; | English | UK | 0 | [10.2471/BLT.16.177758](http://www.doi.org/10.2471/BLT.16.177758) |
| Oswald *et al.* 2017A | Association of community sanitation usage with soil-transmitted helminth infections among school-aged children in Amhara Region, Ethiopia | Parasites and Vectors | Parasitology; | English | UK | 0 | [10.1186/s13071-017-2020-0](http://www.doi.org/10.1186/s13071-017-2020-0) |
| Pattloch *et al.* 2017 | The first biologic for rheumatoid arthritis: factors influencing the therapeutic decision | Zeitschrift für Rheumatologie | Rheumatology | German | Germany | 0 | [10.1007/s00393-016-0174-3](http://www.doi.org/10.1007/s00393-016-0174-3) |
| Paulson *et al.* 2006 | Fall-related injuries among agricultural household members: Regional Rural Injury Study II (RRIS-II) | Journal of Occupational and Environmental Medicine | Public, environmental, & occupational health; | English | USA | 1 - in manuscript | [10.1097/01.jom.0000225110.89501.a3](http://www.doi.org/10.1097/01.jom.0000225110.89501.a3) |
| Phillips *et al.* 2017 | Sarcopenia is associated with disability status-results from the KORA-Age study | Osteoporosis International | Endocrinology & metabolism; | English | Germany | 1 - in supplementary material | [10.1007/s00198-017-4027-y](http://www.doi.org/10.1007/s00198-017-4027-y) |
| Pink *et al.* 2015 | Longitudinal effects of systemic inflammation markers on periodontitis | Journal of Clinical Periodontology | Dentistry, oral surgery & medicine; | English | Germany | 1 - in supplementary material | [10.1111/jcpe.12473](http://www.doi.org/10.1111/jcpe.12473) |
| Preußel *et al.* 2015 | Risk Factors for Sporadic Non-Pregnancy Associated Listeriosis in Germany-Immunocompromised Patients and Frequently Consumed Ready-To-Eat Products.[Erratum appears in PLoS One. 2016 Oct 27;11(10 ):e0165990; PMID: 27788259] | PLoS One | Multidisciplinary sciences; | English | Germany | 1 - in manuscript | [10.1371/journal.pone.0142986](http://www.doi.org/10.1371/journal.pone.0142986) |
| Protudjer *et al.* 2015 | The influence of childhood asthma on puberty and height in Swedish adolescents | Pediatric Allergy and Immunology | Allergy; Immunology; Pediatrics | English | Sweden | 2 - in supplementary material | [10.1111/pai.12398](http://www.doi.org/10.1111/pai.12398) |
| Pyko *et al.* 2015 | Exposure to traffic noise and markers of obesity | Occupational and Environmental Medicine | Public, environmental, & occupational health; | English | Sweden | 1 - in supplementary material | [10.1136/oemed-2014-102516](http://www.doi.org/10.1136/oemed-2014-102516) |
| Pyko *et al.* 2017 | Long-Term Exposure to Transportation Noise in Relation to Development of Obesity-a Cohort Study | Environmental Health Perspectives | Environmental sciences; Public, environmental, & occupational health; Toxicology | English | Sweden | 1 - in supplementary material | [10.1289/EHP1910](http://www.doi.org/10.1289/EHP1910) |
| Rajappan *et al.* 2017 | Maternal body mass index: Relation with infant respiratory symptoms and infections | Pediatric Pulmonology | Pediatrics; Respiratory system; | English | UK | 2 - in supplementary material | [10.1002/ppul.23779](http://www.doi.org/10.1002/ppul.23779) |
| Rancière *et al.* 2017 | Early Exposure to Traffic-Related Air Pollution, Respiratory Symptoms at 4 Years of Age, and Potential Effect Modification by Parental Allergy, Stressful Family Events, and Sex: A Prospective Follow-up Study of the PARIS Birth Cohort | Environmental Health Perspectives | Environmental sciences; Public, environmental, & occupational health; Toxicology | English | France | 1 - in supplementary material | [10.1289/EHP239](http://www.doi.org/10.1289/EHP239) |
| Ratanawongsa *et al.* 2013 | Communication and medication refill adherence the diabetes study of Northern California | JAMA Internal Medicine | Medicine, general, & internal; | English | USA | 1 - in manuscript | [10.1001/jamainternmed.2013](http://www.doi.org/10.1001/jamainternmed.2013) |
| Rebelo *et al.* 2016 | Plasma adiponectin and depressive symptoms during pregnancy and the postpartum period: A prospective cohort study | Journal of Affective Disorders | Clinical neurology; Psychiatry; | English | Brazil | 1 - in supplementary material | [10.1016/j.jad.2016.01.012](http://www.doi.org/10.1016/j.jad.2016.01.012) |
| Rebelo *et al.* 2016A | Changes in Maternal Plasma Adiponectin from Late Pregnancy to the Postpartum Period According to the Mode of Delivery: Results from a Prospective Cohort in Rio de Janeiro, Brazil | PLoS One | Multidisciplinary sciences; | English | Brazil | 1 - in supplementary material | [10.1371/journal.pone.0158886](http://www.doi.org/10.1371/journal.pone.0158886) |
| Rêgo *et al.* 2016 | Physical activity in pregnancy and adverse birth outcomes | Cadernos de saude publica | Public, environmental, & occupational health; | English | Brazil | 1 - in manuscript | [10.1590/0102-311X00086915](http://www.doi.org/10.1590/0102-311X00086915) |
| Reiner *et al.* 2016 | Large machinery-related agricultural injuries across a five-state region in the Midwest | Journal of Occupational and Environmental Medicine | Public, environmental, & occupational health; | English | USA | 1 - in manuscript | [10.1097/JOM.0000000000000584](http://www.doi.org/10.1097/JOM.0000000000000584) |
| Rhea *et al.* 2014 | Risk factors for hospitalization after dog bite injury: A case-cohort study of emergency department visits | Academic Emergency Medicine | Emergency Medicine; | English | USA | 0 | [10.1111/acem.12312](http://www.doi.org/10.1111/acem.12312) |
| Ribeiro *et al.* 2017 | Overweight, obese, underweight, and frequency of sugar consumption as risk indicators for early childhood caries in Brazilian preschool children | International Journal of Paediatric Dentistry | Dentistry, oral surgery & medicine; Pediatrics; | English | Brazil | 1 - in manuscript | [10.1111/ipd.12292](http://www.doi.org/10.1111/ipd.12292) |
| Risch *et al.* 2014 | Risk for Child Maltreatment Among Infants Discharged From a Neonatal Intensive Care Unit: A Sibling Comparison | Child Maltreatment | Family studies; Social work; | English | USA | 0 | [10.1177/1077559514539387](http://www.doi.org/10.1177/1077559514539387) |
| Rusconi *et al.* 2011 | Paracetamol and antibiotics in childhood and subsequent development of wheezing/asthma: Association or causation? | International Journal of Epidemiology | Public, environmental, & occupational health; | English | Italy | 1 - in manuscript | [10.1093/ije/dyq263](http://www.doi.org/10.1093/ije/dyq263) |
| Rutegård *et al.* 2016 | Current use of diverting stoma in anterior resection for cancer: population-based cohort study of total and partial mesorectal excision | International Journal of Colorectal Disease | Gastroenterology & hepatology; Surgery; | English | Sweden | 0 | [10.1007/s00384-015-2465-6](http://www.doi.org/10.1007/s00384-015-2465-6) |
| Rutegård *et al.* 2016A | Non-Steroidal Anti-Inflammatory Drug Use and Risk of Anastomotic Leakage after Anterior Resection: A Protocol-Based Study | Digestive Surgery | Gastroenterology & hepatology; Surgery; | English | Sweden | 0 | [10.1159/000443216](http://www.doi.org/10.1159/000443216) |
| Sage *et al.* 2010 | School resources, resource allocation, and risk of physical assault against Minnesota educators | Accident Analysis and Prevention | Ergonomics; Public, environmental, & occupational health; Social sciences, interdisciplinary | English | USA | 0 | [10.1016/j.aap.2009.04.019](http://www.doi.org/10.1016/j.aap.2009.04.019) |
| Salti *et al.* 2017 | Estimating effects of craniofacial morphology on gingival recession and clinical attachment loss | Journal of Clinical Periodontology | Journal of Dental Research; | English | Germany | 1 - in supplementary material | [10.1111/jcpe.12661](http://www.doi.org/10.1111/jcpe.12661) |
| Samson *et al.* 2016 | Understanding the association of type 2 diabetes mellitus in breast cancer among african american and european american populations in south carolina | Journal of Racial and Ethnic Health Disparities | Not indexed | English | USA | 1 - in manuscript | [10.1007/s40615-015-0173-0](http://www.doi.org/10.1007/s40615-015-0173-0) |
| Schipf *et al.* 2011 | Applying a Directed Acyclic Graph (DAG): Testosterone as a Risk Factor for Development of Type 2 Diabetes Mellitus in the Study of Health in Pomerania (SHIP) | Gesundheitswesen | Public, environmental, & occupational health | German | Germany | 1 – in manuscript | [10.1055/s-0031-1291193](http://www.doi.org/10.1055/s-0031-1291193) |
| Schliep *et al.* 2011 | Effect of male and female body mass index on pregnancy and live birth success after in vitro fertilization | Fertility and Sterility | Obstetrics & Gynecology; Reproductive Biology; | English | USA | 0 | [10.1016/j.fertnstert.2014.10.048](http://www.doi.org/10.1016/j.fertnstert.2014.10.048) |
| Schmidt *et al.* 2013 | Association of pre-diagnosis physical activity with recurrence and mortality among women with breast cancer | International Journal of Cancer | Oncology; | English | Germany | 0 | [10.1002/ijc.28130](http://www.doi.org/10.1002/ijc.28130) |
| Schwahn *et al.* 2013 | Missing, unreplaced teeth and risk of all-cause and cardiovascular mortality | International Journal of Cardiology | Cardiac & Cardiovascular systems; | English | Germany | 1 - in supplementary material | [10.1016/j.ijcard.2012.04.061](http://www.doi.org/10.1016/j.ijcard.2012.04.061) |
| Sehrndt *et al.* 2011 | Investigation of the Influence of the Socioeconomic Status on the Health-Related Quality of Life in Patients Before and After Coronary Artery Bypass Grafting – An Example of the Use of Causal Diagrams (DAGs) | Gesundheitswesen | Public, environmental, & occupational health | German | Germany | 2 – in manuscript | [10.1055/s-0031-1291196](http://www.doi.org/10.1055/s-0031-1291196) |
| Senkomago *et al.* 2015 | Acquisition and Persistence of Human Papillomavirus 16 (HPV-16) and HPV-18 Among Men With High-HPV Viral Load Infections in a Circumcision Trial in Kisumu, Kenya | Journal of Infectious Diseases | Immunology; Infectious disease; Microbiology | English | USA | 0 | [10.1093/infdis/jiu535](http://www.doi.org/10.1093/infdis/jiu535) |
| Senkomago *et al.* 2016 | Higher HPV16 and HPV18 Penile Viral Loads Are Associated With Decreased Human Papillomavirus Clearance in Uncircumcised Kenyan Men | Sexually Transmitted Diseases | Infectious disease; | English | USA | 0 | [10.1097/OLQ.0000000000000500](http://www.doi.org/10.1097/OLQ.0000000000000500) |
| Seward *et al.* 2015 | Using Observational Data to Estimate the Effect of Hand Washing and Clean Delivery Kit Use by Birth Attendants on Maternal Deaths after Home Deliveries in Rural Bangladesh, India and Nepal | PLoS One | Multidisciplinary sciences; | English | UK | 2 - in supplementary material | [10.1371/journal.pone.0136152](http://www.doi.org/10.1371/journal.pone.0136152) |
| Shah *et al.* 2015 | Correlates of prediabetes and type II diabetes in US South Asians: Findings from the Mediators of Atherosclerosis in South Asians Living in America (MASALA) study | Annals of Epidemiology | Public, environmental, & occupational health; | English | USA | 0 | [10.1016/j.annepidem.2014.10.013](http://www.doi.org/10.1016/j.annepidem.2014.10.013) |
| Shaw *et al.* 2018 | What can fuel price increases tell us about the air pollution health co-benefits of a carbon price? | Journal of Transport and Health | Public, environmental, & occupational health; Transportation; | English | New Zealand | 1 - in supplementary material | [10.1016/j.jth.2017.11.002](http://www.doi.org/10.1016/j.jth.2017.11.002) |
| Sheikh *et al.* 2014 | Role of respondents' education as a mediator and moderator in the association between childhood socio-economic status and later health and wellbeing | BMC Public Health | Public, environmental, & occupational health; | English | Norway | 1 - in manuscript | [10.1186/1471-2458-14-1172](http://www.doi.org/10.1186/1471-2458-14-1172) |
| Shoaff *et al.* 2016 | Prenatal phthalate exposure and infant size at birth and gestational duration | Environmental Research | Pharmacology & Pharmacy; | English | USA | 0 – missing from supplementary material | [10.1016/j.envres.2016.05.033](http://www.doi.org/10.1016/j.envres.2016.05.033) |
| Shoaff *et al.* 2017 | Early-Life Phthalate Exposure and Adiposity at 8 Years of Age | Environmental Health Perspectives | Environmental sciences; Public, environmental, & occupational health; | English | USA | 1 - in supplementary material | [10.1289/EHP1022](http://www.doi.org/10.1289/EHP1022) |
| Skretteberg *et al.* 2013 | Triglycerides-diabetes association in healthy middle-aged men: modified by physical fitness? A long term follow-up of 1962 Norwegian men in the Oslo Ischemia Study | Diabetes Research & Clinical Practice | Endocrinology & metabolism; | English | Norway | 0 | [10.1016/j.diabres.2013.06.001](http://www.doi.org/10.1016/j.diabres.2013.06.001) |
| Smallwood *et al.* 2017 | Relationship between urinary nitrate excretion and blood pressure in the in Chianti Cohort | American Journal of Hypertension | Peripheral Vascular Disease; | English | UK | 1 - in supplementary material | [10.1093/ajh/hpx035](http://www.doi.org/10.1093/ajh/hpx035) |
| Sohn *et al.* 2015 | Atypical antipsychotic initiation and the risk of type II diabetes in children and adolescents | Pharmacoepidemiology and Drug Safety | Public, environmental, & occupational health; Occupational health; Pharmacology & pharmacy | English | USA | 0 | [10.1002/pds.3768](http://www.doi.org/10.1002/pds.3768) |
| Solmi *et al.* 2017 | Curiosity killed the cat: No evidence of an association between cat ownership and psychotic symptoms at ages 13 and 18 years in a UK general population cohort | Psychological Medicine | Psychiatry; Psychology; | English | UK | 2 - in supplementary material | [10.1017/S0033291717000125](http://www.doi.org/10.1017/S0033291717000125) |
| Spillane *et al.* 2013 | A cohort study of metformin exposure and survival in patients with stage I-III colorectal cancer | Cancer Epidemiology Biomarkers and Prevention | Oncology; Public, environmental, & occupational health; | English | Ireland | 0 - supplementary material unavailable | [10.1158/1055-9965.EPI-13-0347](http://www.doi.org/10.1158/1055-9965.EPI-13-0347) |
| Spillane *et al.* 2014 | Metformin exposure and disseminated disease in patients with colorectal cancer | Cancer Epidemiology | Oncology; Public, environmental, & occupational health; | English | Ireland | 0 | [10.1016/j.canep.2013.12.003](http://www.doi.org/10.1016/j.canep.2013.12.003) |
| Ssewanyana *et al.* 2015 | Condom use in the context of romantic relationships: A study among university students from 12 universities in four Central and Eastern European countries | European Journal of Contraception and Reproductive Health Care | Public, environmental, & occupational health; Obstetrics & Gynecology; | English | Denmark | 1 - in manuscript | [10.3109/13625187.2014.1001024](http://www.doi.org/10.3109/13625187.2014.1001024) |
| Starling *et al.* 2014 | Perfluoroalkyl substances and lipid concentrations in plasma during pregnancy among women in the Norwegian Mother and Child Cohort Study | Environment International | Environmental sciences; | English | USA | 0 | [10.1016/j.envint.2013.10.004](http://www.doi.org/10.1016/j.envint.2013.10.004) |
| Starling *et al.* 2017 | Blood pressure during pregnancy, neonatal size and altered body composition: The Healthy Start study | Journal of Perinatology | Obstetrics & Gynecology; Pediatrics; | English | USA | 1 - in supplementary material | [10.1038/jp.2016.261](http://www.doi.org/10.1038/jp.2016.261) |
| Stratakis *et al.* 2016 | Fish Intake in Pregnancy and Child Growth A Pooled Analysis of 15 European and US Birth Cohorts | Jama Pediatrics | Pediatrics; | English | Greece | 1 - in supplementary material | [10.1001/jamapediatrics.2015.4430](http://www.doi.org/10.1001/jamapediatrics.2015.4430) |
| Strutz *et al.* 2014 | Selected preconception health indicators and birth weight disparities in a national study | Women's Health Issues | Public, environmental, & occupational health; Womens Studies; | English | USA | 1 - in manuscript | [10.1016/j.whi.2013.10.001](http://www.doi.org/10.1016/j.whi.2013.10.001) |
| Sunol *et al.* 2015 | Implementation of Departmental Quality Strategies Is Positively Associated with Clinical Practice: Results of a Multicenter Study in 73 Hospitals in 7 European Countries | PLoS One | Multidisciplinary sciences; | English | Spain | 1 - in manuscript | [10.1371/journal.pone.0141157](http://www.doi.org/10.1371/journal.pone.0141157) |
| Sunyer *et al.* 2015 | Association between Traffic-Related Air Pollution in Schools and Cognitive Development in Primary School Children: A Prospective Cohort Study | PLoS Medicine | Medicine, general, & internal; | English | Spain | 0 | [10.1371/journal.pmed.1001792](http://www.doi.org/10.1371/journal.pmed.1001792) |
| Tamimi *et al.* 2017 | Effect of acetylcholinesterase inhibitors on post-surgical complications and mortality following a hip fracture: A cohort study | Journal of Musculoskeletal Neuronal Interactions | Not indexed | English | Canada | 0 | N/A |
| Tassiopoulos *et al.* 2017 | Blood Lead Levels and Neurodevelopmental Function in Perinatally HIV-Exposed, Uninfected Children in a U.S.-Based Longitudinal Cohort Study | AIDS Research and Human Retroviruses | Immunology; Infectious disease; Virology | English | USA | 0 | [10.1089/AID.2016.0265](http://www.doi.org/10.1089/AID.2016.0265) |
| Tedla & Bautista 2016 | Drug Side Effect Symptoms and Adherence to Antihypertensive Medication | American Journal of Hypertension | Peripheral Vascular Disease; | English | USA | 1 - in manuscript | [10.1093/ajh/hpv185](http://www.doi.org/10.1093/ajh/hpv185) |
| Tedla *et al.* 2017 | Association between long-term blood pressure control and ten-year progression in carotid arterial stiffness among hypertensive individuals: themultiethnic study of atherosclerosis | Journal of Hypertension | Peripheral Vascular Disease; | English | USA | 0 | [10.1097/HJH.0000000000001199](http://www.doi.org/10.1097/HJH.0000000000001199) |
| Tedla *et al.* 2017A | Association between Long-Term Blood Pressure Variability and 10-Year Progression in Arterial Stiffness | Hypertension | Peripheral Vascular Disease; | English | USA | 0 | [10.1161/HYPERTENSIONAHA.116.08427](http://www.doi.org/10.1161/HYPERTENSIONAHA.116.08427) |
| Traeger *et al.* 2016 | Emotional distress drives health services overuse in patients with acute low back pain: a longitudinal observational study | European Spine Journal | Clinical neurology; Orthopedics; | English | Australia | 4 - in supplementary material | [10.1007/s00586-016-4461-0](http://www.doi.org/10.1007/s00586-016-4461-0) |
| Upson *et al.* 2013 | Phthalates and risk of endometriosis | Environmental Research | Environmental sciences; Public, environmental, & occupational health; | English | USA | 1 - in supplementary material | [10.1016/j.envres.2013.07.003](http://www.doi.org/10.1016/j.envres.2013.07.003) |
| Urquia *et al.* 2011 | Experiences of violence before and during pregnancy and adverse pregnancy outcomes: An analysis of the Canadian Maternity Experiences Survey | BMC Pregnancy and Childbirth | Obstetrics & Gynecology; | English | Canada | 0 | [10.1186/1471-2393-11-42](http://www.doi.org/10.1186/1471-2393-11-42) |
| Valvi *et al.* 2015 | Prenatal phthalate exposure and childhood growth and blood pressure: Evidence from the spanish inma-sabadell birth cohort study | Environmental Health Perspectives | Environmental sciences; Public, environmental, & occupational health; Toxicology | English | Spain | 1 - in supplementary material | [10.1289/ehp.1408887](http://www.doi.org/10.1289/ehp.1408887) |
| Wang & Bautista 2015 | Serum bilirubin and the risk of hypertension | International Journal of Epidemiology | Public, environmental, & occupational health; | English | China | 1 - in manuscript | [10.1093/ije/dyu242](http://www.doi.org/10.1093/ije/dyu242) |
| Waterhouse *et al.* 2016 | Determinants of Outcomes Following Resection for Pancreatic Cancer-a Population-Based Study | Journal of Gastrointestinal Surgery | Gastroenterology & hepatology; Surgery; | English | Australia | 0 | [10.1007/s11605-016-3157-4](http://www.doi.org/10.1007/s11605-016-3157-4) |
| Webb *et al.* 2015 | Circulating 25-hydroxyvitamin D and survival in women with ovarian cancer | American Journal of Clinical Nutrition | Nutrition & dietetics; | English | Australia | 0 | [10.3945/ajcn.114.102681](http://www.doi.org/10.3945/ajcn.114.102681) |
| Wei *et al.* 2016 | The Impact of Homophobia and HIV Stigma on HIV Testing Uptake among Chinese Men Who Have Sex with Men: A Mediation Analysis | Journal of Acquired Immune Deficiency Syndromes | Immunology; Infectious disease; | English | USA | 2 - in manuscript | [10.1097/QAI.0000000000000815](http://www.doi.org/10.1097/QAI.0000000000000815) |
| Weyde *et al.* 2017 | Nocturnal Road Traffic Noise Exposure and Children's Sleep Duration and Sleep Problems | International Journal of Environmental Research and Public Health | Environmental sciences; Public, environmental, & occupational health; | English | Norway | 1 - in supplementary material | [10.3390/ijerph14050491](http://www.doi.org/10.3390/ijerph14050491) |
| Weyde *et al.* 2017A | Road traffic noise and children's inattention | Environmental Health: A Global Access Science Source | Environmental sciences; Public, environmental, & occupational health; | English | Norway | 2 - in supplementary material | [10.1186/s12940-017-0337-y](http://www.doi.org/10.1186/s12940-017-0337-y) |
| Williams *et al.* 2016 | Antiretroviral exposure during pregnancy and adverse outcomes in HIV-exposed uninfected infants and children using a trigger-based design | AIDS | Immunology; Infectious disease; Virology | English | USA | 1 - in supplementary material | [10.1097/QAD.0000000000000916](http://www.doi.org/10.1097/QAD.0000000000000916) |
| Wilson *et al.* 2016 | Factors related to HPV vaccine uptake and 3-dose completion among women in a low vaccination region of the USA: An observational study | BMC Women's Health | Obstetrics & Gynecology; | English | USA | 1 - in manuscript | [10.1186/s12905-016-0323-5](http://www.doi.org/10.1186/s12905-016-0323-5) |
| Windsor *et al.* 2016 | No association between exacerbation frequency and stroke in patients with COPD | International Journal of COPD | Respiratory system; | English | UK | 0 | [10.2147/COPD.S95775](http://www.doi.org/10.2147/COPD.S95775) |
| Yau *et al.* 2015 | Does employee resistance during a robbery increase the risk of customer injury? | Journal of Occupational and Environmental Medicine | Public, environmental, & occupational health; | English | USA | 0 | [10.1097/JOM.0000000000000376](http://www.doi.org/10.1097/JOM.0000000000000376) |
| Yin *et al.* 2014 | Using CD4 Percentage and Age to Optimize Pediatric Antiretroviral Therapy Initiation | Pediatrics | Pediatrics; | English | USA | 0 | [10.1542/peds.2014-0527](http://www.doi.org/10.1542/peds.2014-0527) |
| Zhang *et al.* 2008 | Antibiotic use and the risk of lung cancer | Cancer Epidemiology Biomarkers and Prevention | Oncology; Public, environmental, & occupational health; | English | USA | 4 - in manuscript | [10.1158/1055-9965.EPI-07-2817](http://www.doi.org/10.1158/1055-9965.EPI-07-2817) |
| Zhang *et al.* 2010 | Reduced birthweight in short or primiparous mothers: Physiological or pathological? | BJOG: An International Journal of Obstetrics and Gynaecology | Obstetrics & Gynecology; | English | Canada | 1 - in manuscript | [10.1111/j.1471-0528.2010.02642.x](http://www.doi.org/10.1111/j.1471-0528.2010.02642.x) |

# Supplementary **Table S2.**

Summary details of the largest causal diagram reported in the 144 studies with at least one diagram available.

| **Article** | **Diagram examined** | **Nodes** | **Arcs** | **Ratio of arcs to nodes** | **Percent saturationa** | **Drawn in DAGitty** | **Includes super-nodesb** | **Includes unobserved variables** | **Arcs flow in consistent direction** | **Citations used to justify arcs** |
| --- | --- | --- | --- | --- | --- | --- | --- | --- | --- | --- |
| Åkerstedt *et al.* 2017 | Figure S1 | 15 | 65 | 4.3 | 62% | Yes | No | No | No | No |
| Al-Harbi & El Tantawi 2017 | Figure 1(a) | 18 | 41 | 2.3 | 27% | Yes | No | Yes – specific | No | No |
| Andersen *et al.* 2014 | Supplemental Figure III | 16 | 83 | 5.2 | 69% | Yes | No | No | No | No |
| Andreano *et al.* 2017 | Online Resource 1 | 12 | 39 | 3.3 | 59% | Yes | No | No | No | No |
| Arora *et al.* 2014 | Figure 1(A) | 6 | 15 | 2.5 | 100% | No | Yes | No | No | No |
| Asgari *et al.* 2011 | Figure | 11 | 26 | 2.4 | 47% | No | Yes | No | Yes - Left-to-Right | Yes - inclusion |
| Bach *et al.* 2015 | Additional Figure 1 | 11 | 18 | 1.6 | 33% | Yes | No | Yes - specific | No | No |
| Badland *et al.* 2017 | Figure 3 | 12 | 20 | 1.7 | 30% | No | No | Yes - specific | Yes - Left-to-Right | No |
| Bahls *et al.* 2017 | Supplementary Figure 1 | 12 | 24 | 2.0 | 36% | Yes | No | No | No | No |
| Bernardi *et al.* 2015 | Online Supplemental Material | 12 | 31 | 2.6 | 47% | Yes | No | No | Yes - Top-Left-to-Bottom-Right | No |
| Birungi *et al.* 2017 | Figure 1 | 12 | 37 | 3.1 | 56% | No | No | Yes - specific | No | Yes - inclusion |
| Bliddal *et al.* 2016 | N/A (provided on request) | 10 | 23 | 2.3 | 51% | Yes | No | Yes - generic | No | No |
| Blighe *et al.* 2017 | Online Supplemental Material | 8 | 9 | 1.1 | 32% | Yes | No | No | Yes - Left-to-Right | Yes - inclusion |
| Blom *et al.* 2015 | Additional File 1 | 27 | 49 | 1.8 | 14% | Yes | No | Yes - specific | No | No |
| Blomberg *et al.* 2013 | Appendix 1 | 16 | 33 | 2.1 | 28% | Yes | No | Yes - specific | Yes - Top Left-to-Bottom Right | No |
| Bodén *et al.* 2015 | Figure S1 | 13 | 41 | 3.2 | 53% | Yes | No | Yes - specific | No | No |
| Boyle *et al.* 2015 | Supplementary Figure 2 | 13 | 28 | 2.2 | 36% | No | No | Yes – specific | Yes - Left-to-Right | No |
| Boyle *et al.* 2016 | Supplementary Figure 2 | 21 | 99 | 4.7 | 47% | Yes | No | No | No | No |
| Buchner & Rehfuess 2015 | Figure 5 in Rehfuess et al. 2013 | 28 | 46 | 1.6 | 12% | No | Yes | Yes - specific | No | No |
| Buckley *et al.* 2016 | Figure S11 | 16 | 48 | 3.0 | 40% | Yes | No | No | No | No |
| Busert *et al.* 2016 | Supplementary Figure 1 | 19 | 53 | 2.8 | 31% | No | No | Yes - specific | No | No |
| Byberg *et al.* 2016 | Additional Figure 1 | 20 | 61 | 3.1 | 32% | Yes | No | Yes - generic & specific | No | No |
| Camelo *et al.* 2015 | Figure 1 | 7 | 12 | 1.7 | 57% | No | Yes | Yes – generic | Yes - Left-to-Right | No |
| Carlson *et al.* 2005 | Figure 1 | 9 | 27 | 3.0 | 75% | No | No | Yes – generic | Yes - Top-to-Bottom | No |
| Casas *et al.* 2015 | Figure S1 | 15 | 37 | 2.5 | 35% | Yes | No | No | No | No |
| Chandee *et al.* 2017 | Supplementary Figure 1 | 12 | 44 | 3.7 | 67% | Yes | No | No | No | No |
| Chattopadhyay *et al.* 2003 | Figure 1 | 10 | 17 | 1.7 | 38% | No | No | No | Yes - Bottom Right-to-Top Left | No |
| Curran *et al.* 2016 | Supplementary Figure 1 | 18 | 31 | 1.7 | 20% | Yes | No | Yes – specific | No | No |
| Curran *et al.* 2017 | Figure S1 | 13 | 31 | 2.4 | 40% | Yes | No | Yes – specific | No | No |
| Dahl *et al.* 2012 | Figure S1 | 14 | 29 | 2.1 | 32% | No | Yes | Yes – generic & specific | No | No |
| de Jonge *et al.* 2014 | Additional Figure 1 | 9 | 27 | 3.0 | 75% | Yes | No | No | No | No |
| di Giuseppe *et al.* 2015 | Figure 2 | 12 | 50 | 4.2 | 76% | Yes | Yes | No | No | No |
| Ellison & De Wet 2016 | Figure 1 | 13 | 75 | 5.8 | 96% | No | No | No | Yes - Left-to-Right | No |
| Emeny *et al.* 2014 | Supplementary Figure 1 | 11 | 21 | 1.9 | 38% | Yes | No | No | No | No |
| Erkal *et al.* 2009 | Figure 2 | 10 | 28 | 2.8 | 62% | No | No | Yes – generic | No | No |
| Escobar *et al.* 2017 | Figure 7 | 9 | 16 | 1.8 | 44% | Yes | No | No | No | No |
| Evandt *et al.* 2017 | Figure 1 | 8 | 15 | 1.9 | 54% | No | No | No | Yes - Top Left-to-Bottom Right | No |
| Evandt *et al.* 2017A | Figure S1 | 11 | 23 | 2.1 | 42% | No | Yes | No | Yes - Top Left-to-Bottom Right | No |
| Feda *et al.* 2010 | Figure I | 9 | 26 | 2.9 | 72% | No | No | No | No | No |
| Ferraro *et al.* 2017 | Supplemental Figure S2 | 24 | 74 | 3.1 | 27% | Yes | No | No | No | No |
| Figueiredo *et al.* 2017 | Supplemental Figure 2 | 19 | 40 | 2.1 | 23% | Yes | No | Yes – specific | No | No |
| Filippidis *et al.* 2017 | eFigure 2 | 17 | 34 | 2.0 | 25% | No | No | No | No | No |
| Filteau *et al.* 2016 | Figure 1 | 14 | 26 | 1.9 | 29% | No | Yes | No | No | No |
| Frederiksen *et al.* 2009 | Figure 3 | 9 | 31 | 3.4 | 86% | No | No | No | Yes - Left-to-Right | No |
| Gagliardi *et al.* 2009 | Figure 2 | 10 | 19 | 1.9 | 42% | No | No | Yes – generic | Yes - Left-to-Right | No |
| Gascon *et al.* 2015 | Figure E1 | 18 | 26 | 1.4 | 17% | Yes | No | No | Yes - Top Left-to-Bottom Right | No |
| Gaspar *et al.* 2017 | Figure S1 | 23 | 70 | 3.0 | 28% | No | No | No | Yes - Left-to-Right | No |
| Gocke *et al.* 2014 | Supplementary Figure 1 | 13 | 65 | 5.0 | 83% | Yes | No | No | No | No |
| Gray *et al.* 2017 | Figure S1 | 14 | 31 | 2.2 | 34% | Yes | No | No | No | No |
| Grice *et al.* 2007 | Figure C | 16 | 29 | 1.8 | 24% | No | No | No | No | No |
| Grice *et al.* 2010 | Figure 1 | 11 | 47 | 4.3 | 85% | No | No | No | No | No |
| Grill *et al.* 2014 | Figure 1 | 10 | 33 | 3.3 | 73% | No | No | No | No | Yes - inclusion |
| Grundy *et al.* 2017 | Figure 1 | 11 | 19 | 1.7 | 35% | Yes | No | No | No | No |
| Guimarães *et al.* 2016 | Figure 1 | 6 | 10 | 1.7 | 67% | No | Yes | Yes – generic | Yes - Left-to-Right | No |
| Gunathilake *et al.* 2016 | Supplementary Figure 1 | 14 | 44 | 3.1 | 48% | Yes | No | No | No | No |
| Harris-Adamson *et al.* 2016 | Figure 1 | 6 | 10 | 1.7 | 67% | No | No | Yes – generic | Yes - Left-to-Right | No |
| Harskamp-van Ginkel *et al.* 2015 | S1 Figure | 15 | 39 | 2.6 | 37% | Yes | No | No | No | No |
| Herich *et al.* 2017 | Appendix Figure 1 | 16 | 34 | 2.1 | 28% | No | No | Yes – specific | No | No |
| Hickson *et al.* 2017 | Figure 1 | 8 | 20 | 2.5 | 71% | No | No | No | Yes - Left-to-Right | No |
| Hinkle *et al.* 2013 | Figure 1 | 10 | 25 | 2.5 | 56% | No | No | Yes – generic | Yes - Top Left-to-Bottom Right | No |
| Jämsä *et al.* 2017 | Supplementary data | 19 | 50 | 2.6 | 29% | Yes | No | Yes – generic & specific | No | No |
| Johri *et al.* 2016 | Supplemental Figure 1 | 24 | 61 | 2.5 | 22% | Yes | No | Yes- specific | No | Yes - inclusion |
| Jonker *et al.* 2012 | Figure 1 | 16 | 37 | 2.3 | 31% | No | No | Yes – generic | No | No |
| Kalapatapu *et al.* 2017 | Figure 2 | 7 | 11 | 1.6 | 52% | Yes | No | No | Yes - Top Left-to-Bottom Right | No |
| Kalapatapu *et al.* 2017A | Figure 2 | 12 | 20 | 1.7 | 30% | Yes | No | No | No | No |
| Karahalios *et al.* 2014 | Figure S1 | 8 | 17 | 2.1 | 61% | No | Yes | No | No | No |
| Karahalios *et al.* 2016 | Additional File 1 | 8 | 18 | 2.3 | 64% | No | Yes | No | No | No |
| Kebede *et al.* 2017 | Supplementary Figure 2 | 15 | 73 | 4.9 | 70% | Yes | No | No | No | No |
| Kerschberger *et al.* 2012 | Figure S1 – DAG 1 | 10 | 17 | 1.7 | 38% | No | No | No | No | No |
| Khalifa *et al.* 2016 | Figure 2 | 15 | 26 | 1.7 | 25% | Yes | No | No | No | No |
| Kharmats *et al.* 2014 | Supplementary Figure 2 | 17 | 28 | 1.6 | 21% | Yes | No | Yes – specific | Yes - Top Left-to-Bottom Right | No |
| Kroenke *et al.* 2016 | eFigure 1 | 11 | 33 | 3.0 | 60% | No | Yes | Yes – generic | Yes - Left-to-Right | No |
| Kverneng Hultberg *et al.* 2017 | Supplementary Figure 1 | 14 | 31 | 2.2 | 34% | Yes | No | No | No | No |
| Lai *et al.* 2016 | eFigure 2 | 13 | 22 | 1.7 | 28% | Yes | No | Yes – specific | No | No |
| Launay *et al.* 2014 | Figure S2 | 6 | 10 | 1.7 | 67% | Yes | Yes | No | Yes - Top Left-to-Bottom Right | No |
| Liang *et al.* 2017 | Figure S1 | 16 | 42 | 2.6 | 35% | Yes | No | No | No | No |
| Liebers *et al.* 2011 | App. 1 | 11 | 23 | 2.1 | 42% | No | Yes | No | Yes - top-left-to-bottom-right | No |
| Lima *et al.* 2017 | Figure 1A | 12 | 20 | 1.7 | 30% | Yes | No | No | No | No |
| Magadi & Magadi 2017 | Figure 1 | 10 | 20 | 2.0 | 44% | Yes | No | No | No | No |
| Maika *et al.* 2015 | Figure 2 | 7 | 19 | 2.7 | 90% | Yes | No | Yes - generic | Yes - Left-to-Right | No |
| Maretty-Nielsen *et al.* 2014 | Figure 1 | 13 | 38 | 2.9 | 49% | No | No | No | No | No |
| Maretty-Nielsen *et al.* 2014A | Figure 2 (Supplementary) | 14 | 42 | 3.0 | 46% | No | No | Yes - generic | No | No |
| Martin *et al.* 2017 | Appendix 3 | 12 | 20 | 1.7 | 30% | Yes | No | Yes – generic | No | No |
| Matser *et al.* 2013 | Figure 1 | 6 | 11 | 1.8 | 73% | No | Yes | No | No | No |
| Mebrahtu *et al.* 2015 | Figure S2 | 15 | 36 | 2.4 | 34% | Yes | No | No | No | No |
| Medenwald *et al.* 2014 | Figure S1 | 10 | 29 | 2.9 | 64% | No | No | Yes – specific | No | No |
| Medenwald *et al.* 2015 | Figure 1 | 5 | 7 | 1.4 | 70% | No | Yes | Yes - generic | Yes - Top Left-to-Bottom Right | No |
| Medenwald *et al.* 2016A | Figure 1 | 5 | 8 | 1.6 | 80% | No | No | Yes - generic | Yes - Top Left-to-Bottom Right | No |
| Messerlian *et al.* 2017 | Supplementary Figure 2A | 5 | 10 | 2.0 | 100% | Yes | No | No | No | No |
| Møller *et al.* 2016 | Figure 1 | 11 | 21 | 1.9 | 38% | No | No | No | Yes - Left-to-Right | No |
| Ng *et al.* 2017 | Figure S1 | 17 | 57 | 3.4 | 42% | Yes | Yes | No | Yes - Top Left-to-Bottom Right | No |
| Ng *et al.* 2017A | Figure S1 | 27 | 62 | 2.3 | 18% | Yes | No | Yes – specific | Yes - Top Left-to-Bottom Right | No |
| Ngueta *et al.* 2016 | Figure S2 | 18 | 37 | 2.1 | 24% | No | No | No | No | No |
| Norris *et al.* 2017 | Supplementary Figure 1 | 15 | 49 | 3.3 | 47% | Yes | No | No | No | No |
| Nourbakhsh *et al.* 2016 | Figure 1 | 6 | 7 | 1.2 | 47% | Yes | No | No | No | No |
| Nowak *et al.* 2016 | Online Digital Content 1 - Figure | 8 | 22 | 2.8 | 79% | No | No | No | Yes - Top Left-to-Bottom Right | No |
| Oddo *et al.* 2017A | Figure 1 | 17 | 30 | 1.8 | 22% | Yes | No | Yes – specific | No | No |
| Olsen *et al.* 2015 | Figure 2 | 8 | 13 | 1.6 | 46% | No | Yes | No | Yes - Left-to-Right | No |
| Orban *et al.* 2016 | Figure S1 | 10 | 27 | 2.7 | 60% | Yes | No | No | No | No |
| Osler *et al.* 2015 | Figure 1 | 8 | 19 | 2.4 | 68% | No | No | No | No | No |
| Paulson *et al.* 2006 | Figure 1 | 10 | 32 | 3.2 | 71% | No | No | Yes – generic | Yes - Top-to-Bottom | No |
| Phillips *et al.* 2017 | Online Resource Figure 2 | 21 | 62 | 3.0 | 30% | No | No | No | No | Yes - inclusion |
| Pink *et al.* 2015 | Suppl Figure 1 | 11 | 45 | 4.1 | 82% | Yes | Yes | No | No | No |
| Preußel *et al.* 2015 | Figure 1 | 9 | 25 | 2.8 | 69% | No | No | No | No | Yes - inclusion |
| Protudjer *et al.* 2015 | Figure S2 | 7 | 11 | 1.6 | 52% | Yes | No | No | No | No |
| Pyko *et al.* 2015 | eFigure 1 | 22 | 69 | 3.1 | 30% | Yes | No | Yes – specific | No | No |
| Pyko *et al.* 2017 | Supplementary Figure S2 | 22 | 64 | 2.9 | 28% | Yes | No | Yes – specific | No | No |
| Rajappan *et al.* 2017 | Figure S1B | 8 | 21 | 2.6 | 75% | Yes | No | Yes – generic | No | No |
| Rancière *et al.* 2017 | Figure S1 | 18 | 52 | 2.9 | 34% | Yes | Yes | Yes – generic | No | No |
| Ratanawongsa *et al.* 2013 | Figure 2 | 12 | 33 | 2.8 | 50% | No | No | No | No | No |
| Rebe~~l~~o *et al.* 2016 | Figure S1 | 21 | 60 | 2.9 | 29% | Yes | No | No | No | No |
| Rebelo *et al.* 2016A | Figure S1 | 17 | 48 | 2.8 | 35% | Yes | No | No | No | No |
| Rêgo *et al.* 2016 | Figure 1 | 6 | 11 | 1.8 | 73% | Yes | No | No | Yes - Bottom Left-to-Top Right | No |
| Reiner *et al.* 2016 | Figure 1 | 9 | 28 | 3.1 | 78% | No | No | Yes – generic | Yes - Top-to-Bottom | No |
| Ribeiro *et al.* 2017 | Figure 2 | 7 | 19 | 2.7 | 90% | Yes | No | No | No | No |
| Rusconi *et al.* 2011 | Figure 1 | 6 | 6 | 1.0 | 40% | No | No | Yes - generic | Yes - Left-to-Right | No |
| Salti *et al.* 2017 | Supplementary Figure 1 | 12 | 45 | 3.8 | 68% | Yes | No | No | No | No |
| Samson *et al.* 2016 | Figure 1 | 6 | 9 | 1.5 | 60% | Yes | No | Yes - generic | Yes - Top Left-to-Bottom Right | No |
| Schipft *et al.* 2011 | Abb. 1 | 7 | 15 | 2.1 | 71% | No | No | No | No | No |
| Schwahn *et al.* 2013 | eFigure 1 | 16 | 61 | 3.8 | 51% | Yes | No | Yes – generic | Yes - Top-to-Bottom | No |
| Sehrndt *et al.* 2011 | Abb. 2 | 11 | 37 | 3.4 | 67% | No | No | No | No | No |
| Seward *et al.* 2015 | SI1 Figure 1 | 7 | 18 | 2.6 | 86% | No | Yes | No | Yes - Left-to-Right | No |
| Shaw *et al.* 2018 | Appendix A Fig. A1 | 7 | 12 | 1.7 | 57% | No | No | Yes - specific | Yes - Top Left-to-Bottom Right | No |
| Sheikh *et al.* 2014 | Figure 1 | 6 | 10 | 1.7 | 67% | Yes | No | No | Yes - Top Left-to-Bottom Right | No |
| Shoaff *et al.* 2017 | Figure S5 | 13 | 31 | 2.4 | 40% | No | Yes | No | No | No |
| Smallwood *et al.* 2017 | Appendix A | 20 | 53 | 2.7 | 28% | Yes | No | No | No | No |
| Solmi *et al.* 2017 | Figure S2 | 16 | 28 | 1.8 | 23% | Yes | No | No | No | No |
| Ssewanyana *et al.* 2015 | Figure 1 | 11 | 25 | 2.3 | 45% | Yes | No | No | No | No |
| Starling *et al.* 2017 | Supplemental Figure 2 | 9 | 27 | 3.0 | 75% | Yes | No | No | No | No |
| Stratakis *et al.* 2016 | eFigure 2 | 18 | 39 | 2.2 | 25% | Yes | No | No | No | No |
| Strutz *et al.* 2014 | Figure 1 | 4 | 5 | 1.3 | 83% | No | Yes | No | Yes - Left-to-Right | No |
| Sunol *et al.* 2015 | Figure 1 | 10 | 33 | 3.3 | 73% | No | No | Yes – generic | Yes - Top-to-Bottom | No |
| Tedla & Bautista 2016 | Figure 1 | 17 | 41 | 2.4 | 30% | No | No | No | No | No |
| Traeger *et al.* 2016 | Online Resource 1: Figure 3 | 12 | 29 | 2.4 | 44% | No | Yes | Yes – specific | No | No |
| Upson *et al.* 2013 | Supplemental Material Figure 1 | 11 | 18 | 1.6 | 33% | No | No | Yes – generic | No | No |
| Valvi *et al.* 2015 | Figure S1 | 14 | 46 | 3.3 | 51% | Yes | No | No | No | No |
| Wang & Bautista 2015 | Figure 1 | 19 | 65 | 3.4 | 38% | Yes | No | No | No | Yes - inclusion |
| Wei *et al.* 2016 | Figure 1 | 3 | 3 | 1.0 | 100% | No | No | No | Yes - Left-to-Right | No |
| Weyde *et al.* 2017 | Figure S1 | 13 | 28 | 2.2 | 36% | Yes | No | Yes – specific | No | No |
| Weyde *et al.* 2017A | Additional File 2 | 15 | 33 | 2.2 | 31% | Yes | No | No | No | No |
| Williams *et al.* 2016 | Supplemental Figure 1 | 6 | 9 | 1.5 | 60% | Yes | No | No | Yes - Left-to-Right | No |
| Wilson *et al.* 2016 | Figure 2 | 23 | 36 | 1.6 | 14% | Yes | No | Yes – specific | Yes - Top Left-to-Bottom Right | No |
| Zhang *et al.* 2008 | Figure 2(d) | 5 | 5 | 1.0 | 50% | No | No | No | Yes - Left-to-Right | No |
| Zhang *et al.* 2010 | Figure 1 | 3 | 3 | 1.0 | 100% | No | Yes | No | Yes - Top Left-to-Bottom Right | No |

aThe degree of saturation represents the ratio of all included arcs to all possible arcs. The total possible arcs may be calculated from 0.5×N×(N-1) where N is the total number of nodes. **b**Nodes containing more than one variable

# **Supplementary Table S3.**

Relationship between common diagram features and probability of data extraction error by the first data extractor.

| **Continuous variable** | **Unit** | **Relative probability**  **of error (95% CI)** |
| --- | --- | --- |
| Total arcs | (per arc) | 1.016 (1.008, 1.024) |
| Total nodes | (per node) | 1.042 (1.011, 1.075) |
| **Categorical variable** | **Category** | **Model marginal probability (%)**  **of errora (95% CI)** |
| Drawn in DAGitty | No | 50.1 (37.7, 62.6) |
|  | Yes | 32.4 (23.2, 41.8) |
| Arrangement of arcs | No consistent direction | 40.7 (31.1, 50.2) |
|  | Top-to-bottom or left-to-right | 29.5 (11.1, 48.0) |
|  | Corner-to-corner | 43.9 (19.7, 68.1) |
| Includes unobserved variables | No | 37.5 (27.9, 47.1) |
|  | Yes | 42.4 (29.1, 55.8) |
| Includes super-nodesb | No | 39.3 (30.9, 47.8) |
|  | Yes | 38.8 (17.8, 59.7) |

aAdjusted for total number of nodes and arcs bNodes containing more than one variable

# **Supplementary Table S4.**

Summary details of the analytical approach, estimand(s) of interest, and adjustment set(s) used by the 234 articles included in the review.

| **Citation** | **Analytical approach** | **Report adjustment set(s)** | **Report estimand(s) of interest** | **Report estimate(s) from DAG-implied adjustment set** | **Report estimate(s) from other - or unclearly defined - adjustment set(s)** | **Use other variable reduction algorithm / criteria** |
| --- | --- | --- | --- | --- | --- | --- |
| Åkerstedt *et al.* 2017 | Multivariable regression | Yes – report SASa | Total effect | Yes - sensitivity analysis | Yes - primary results. Adjustment set did not include two confounders '*due to a large proportion of missingness and degree of potential misclassification*' | No |
| Al-Farsi *et al.* 2010 | Multivariable regression | Yes – though not stated as SASa | Not reported  (*'effect*') | Yes - primary results | Yes – sensitivity analyses performed with additional adjustments | No |
| Al-Harbi & El Tantawi 2017 | Multivariable regression | No – not explicitly reported | Not reported ('*relationship*') | N/A | Yes - primary results. '*Confounders were detected using the software algorithm and a set of them was selected containing the minimal number of variables that needed to be controlled in multivariable analysis to produce unbiased estimates*' | No |
| Andell *et al.* 2014 | Multivariable regression | No – adjustment set(s) stated, but relationship to DAG(s) and/or SASa is not explicit | Not reported (’*relationship*') | N/A | Yes - primary results. '*The selection of covariates included in these models was performed with the use of a direct acyclical graph*' | No |
| Andell *et al.* 2015 | Multivariable regression | No – adjustment set(s) stated, but relationship to DAG(s) and/or SASa is not explicit | Not reported  ('*effect*') | N/A | Yes - primary results. '*Potential confounders were identified using an a priori direct acyclic graph*' | No |
| Andersen *et al.* 2014 | Multivariable regression | Yes – report SASa | Total and direct effects | Yes - primary results | No | No |
| Andreano *et al.* 2017 | IPWc of MSMd | Yes – report SASa | Not reported  ('*causal effect*') | Yes - primary results | No | No |
| Arora *et al.* 2014 | Multivariable regression | Yes – report SASa | Not reported ('*association*') | Yes - primary results | Yes – primary results include five different adjustment sets | Yes – change-in-estimate for sensitivity analyses |
| Asgari *et al.* 2011 | Multivariable regression | No – adjustment set(s) stated in table footer(s), but relationship to DAG(s) and/or SASa is not explicit | Not reported ('*association*') | N/A | Yes - primary results. *'According to the assumptions in the DAG, we determined a set of adjusting variables for each exposure and used standard logistic regression analysis of the outcome on the exposure, including all covariates in the adjustment set'* | No |
| Ashley-Martin *et al.* 2015 | Multivariable regression | No – adjustment set(s) stated in table footer(s), but relationship to DAG(s) and/or SASa is not explicit | Not reported ('*association*') | N/A | Yes - primary results. *'The minimal adjustment sets for confounding were identified using DAGitty... Specific gravity was forced into the adjusted phthalate/BPA model to account for heterogeneity in urinary dilution...analyses were also stratified by sex'* | No |
| Bach *et al.* 2015 | Multivariable regression | No – adjustment set(s) stated, but relationship to DAG(s) and/or SASa is not explicit | Not reported ('*association*') | N/A | Yes - primary results. *'Selection of covariates was based on a DAG... For the pooled analyses... we additionally adjusted for the sample'* | No |
| Badland *et al.* 2017 | Multivariable regression | No – adjustment set(s) stated in table footer(s), but relationship to DAG(s) and/or SASa is not explicit | Not reported ('*associations*') | N/A | Yes - primary results*. 'We constructed two directed acyclic graphs (DAGs) to map hypothesised pathways and temporal contributions between variables... models were adjusted for individual-level sociodemographic confounders'* | No |
| Bahls *et al.* 2017 | Multivariable regression and propensity score matching | No – not explicitly reported | Not reported ('*associations*') | N/A | Yes - primary results. Two models are reported, one informed by the DAG and a *'clinical model'* with several additional variables. | No |
| Barcelona de Mendoza *et al.* 2016 | Multivariable regression | No – adjustment set(s) stated, but relationship to DAG(s) and/or SASa is not explicit | Not reported (*'relationship'*) | N/A | Yes - primary results. *'Potential confounders were identified via a priori knowledge and Directed Acyclic Graphs (DAGs)... Based on DAGs, the following covariates were included'* | Yes – change-in-estimate criteria |
| Barcelona de Mendoza *et al.* 2016A | Multivariable regression | No - reported adjustment set is after variable reduction methods have been applied | Not reported  ('*effects*') | N/A | Yes- primary results. Adjustment set built with variable reduction. | Yes – change-in-estimate criteria |
| Barcelona de Mendoza *et al.* 2016B | Multivariable regression | No – adjustment set(s) stated, but relationship to DAG(s) and/or SASa is not explicit | Not reported  (*'effect'*) | N/A | Yes - primary results. *'We considered social and demographic risk factors that could confound the relationship between acculturation and anxiety via Directed Acyclic Graphs'* | No |
| Bernardi *et al.* 2015 | Multivariable regression | Yes – report SASa | Not reported ('*association*') | Yes - primary results | No | No |
| Birungi *et al.* 2017 | Multivariable regression | Yes – report SASa | Total and direct effects | Yes - primary results | No | No |
| Bjertness *et al.* 2016 | Multivariable regression | No – adjustment set(s) stated, but relationship to DAG(s) and/or SASa is not explicit | Not reported ('*exposure-outcome-relationships*') | N/A | Yes - primary results. Variables were selected for adjustment *'based on the DAG'*. | No |
| Bliddal *et al.* 2016 | Multivariable regression | No – adjustment set(s) stated in table footer(s), but relationship to DAG(s) and/or SASa is not explicit | Not reported ('*associations*') | N/A | Yes - primary results. *'Potential confounders were chosen according to a directed acyclic graph'* | No |
| Blighe *et al.* 2017 | Multivariable regression | No – adjustment set(s) stated in table footer(s), but relationship to DAG(s) and/or SASa is not explicit | Not reported  (*'effect'*) | N/A | Yes - primary results. *'We built a directed acyclic graph... to identify the appropriate confounding variables to adjust for'* | No |
| Blom *et al.* 2015 | Multivariable regression | Yes – report SASa | Not reported ('*association*') | Yes - primary results | Yes - sensitivity results includes additional covariates *'to improve face validity'.* | No |
| Blomberg *et al.* 2013 | Multivariable regression and Bayesian hierarchical modelling | Yes – report SASa | Total effect | Yes - primary results | Yes - primary results of two other adjustment sets are reported alongside DAG-implied set | No |
| Bodén *et al.* 2015 | Multivariable regression | Yes – report SASa | Direct effect | Yes - primary results | Yes - primary results of three other adjustment sets are reported alongside DAG-implied set | No |
| Bowatte *et al.* 2017 | Multivariable regression | No – adjustment set(s) stated, but relationship to DAG(s) and/or SASa is not explicit | Not reported ('*association*') | N/A | Yes - primary results. *'Covariates were selected for the models after considering alternative causal models using a directed acyclic graph'* | No |
| Bowatte *et al.* 2017A | Multivariable regression | Yes – though not stated as SASa | Not reported ('*association*') | Yes - primary results | Yes - secondary or sensitivity results | No |
| Boyle *et al.* 2015 | Multivariable regression | Yes – report SASa | Not reported ('*association*') | No | Yes – primary results. Adjustment set built with variable reduction. | Yes - covariate retention based on change-in-estimate criteria |
| Boyle *et al.* 2016 | Multivariable regression | Yes – report SASa | Not reported ('*association*') | Yes - primary results | No | No |
| Buchner & Rehfuess 2015 | Multivariable regression | No - reported adjustment set is after variable reduction methods have been applied | Not reported  (*'risk factor'*) | N/A | Yes - primary results. Adjustment sets built with variable reduction. | Yes - covariates retention based on information criteria |
| Buckley *et al.* 2016 | Multivariable regression | No – adjustment set(s) stated, but relationship to DAG(s) and/or SASa is not explicit | Not reported ('*association*') | N/A | Yes - primary results. *'We adjusted for potential confounding variables identified using directed acyclic graphs... We also included strong predictors of the outcome, including maternal height and child physical activity at follow-up (active/inactive), to improve precision'* | No |
| Busert *et al.* 2016 | Multivariable regression | Yes – report SASa | Not reported ('*association*') | Yes - primary results | No | No |
| Byberg *et al.* 2016 | Bayesian hierarchical modelling | No – adjustment set(s) stated, but relationship to DAG(s) and/or SASa is not explicit | Not reported ('*association*') | N/A | Yes - primary results. Adjustment set built using variable reduction. | Yes - covariable retention based on p-value criteria |
| Camelo *et al.* 2015 | Mediation analysis with multivariable regression | No - reported adjustment set is after variable reduction methods have been applied | Not reported ('*associations*') | N/A | Yes - primary results. Adjustment sets built with variable reduction. | No |
| Camlin *et al.* 2016 | Multivariable regression | Yes – report SASa | Total effect | Yes - primary results | No | No |
| Carlson *et al.* 2005 | Multivariable regression | Yes – report SASa | Not reported  (*'effect'*) | Yes - primary results | No | No |
| Carlson *et al.* 2006 | Multivariable regression | No – adjustment set(s) stated in table footer(s), but relationship to DAG(s) and/or SASa is not explicit | Not reported ('*association*') | N/A | Yes - primary results. *'To determine potential confounders, we used a causal model...to guide the design and use of directed acyclic graphs'* | No |
| Casas *et al.* 2015 | Multivariable regression | No – adjustment set(s) stated, but relationship to DAG(s) and/or SASa is not explicit | Not reported  (*'effect'*) | N/A | Yes - primary results. Adjustment set built using variable reduction. | Yes - covariate selection based on change-in-estimate criteria |
| Chandee *et al.* 2017 | Multivariable regression | Yes – report SASa | Not reported  (*'effect'*) | Yes - primary results | No | No |
| Chattopadhyay *et al.* 2003 | Multivariable regression | No – not explicitly reported | Not reported ('*association*') | N/A | Yes - primary results come from adjustment set built using variable reduction (*'Final model'*). | Yes - covariate selection based on log-likelihood criteria |
| Cupul-Uicab *et al.* 2013 | Multivariable regression | Yes – though not stated as SASa | Not reported ('*association*') | Yes - primary results | Yes - primary results include an additional covariate for one of the outcomes | Yes - covariate retention based on change-in-estimate criteria |
| Cupul-Uicab *et al.* 2014 | Multivariable regression | Yes – report SASa | Not reported ('*association*') | Yes - primary results | No | Yes - covariate selection based on change-in-estimate criteria |
| Curran *et al.* 2016 | Multivariable regression | No – adjustment set(s) stated, but relationship to DAG(s) and/or SASa is not explicit | Not reported ('*association*') | N/A | Yes - primary results. *'Potential confounders were identified using previous literature and examined for inclusion using a directed acyclic graph'* | No |
| Curran *et al.* 2017 | Multivariable regression | No – adjustment set(s) stated, but relationship to DAG(s) and/or SASa is not explicit | Not reported ('*association*') | N/A | Yes - primary results. *'Based on previous literature and the use of a directed acyclic graph... the following a priori co-variates were included...though not identified as confounders in the DAG, further co-variates that were identified based on previous literature were also assessed, including...'* | Yes - covariate selection based on change-in-estimate criteria |
| Dahl *et al.* 2012 | Multivariable regression | Yes – though not stated as SASa | Not reported ('*association*') | Yes - primary results | No | No |
| Dalgaard *et al.* 2016 | Multivariable regression | Yes – report SASa | Total effect | Yes - primary results | No | No |
| de Jonge *et al.* 2014 | Multivariable regression | Yes – though not stated as SASa | Not reported  ('*risk factors*') | Yes - primary results | Yes - primary results. Unclear relationship with DAG as the same adjustment set was used for different combinations of exposures and outcomes | No |
| di Giuseppe *et al.* 2015 | Multivariable regression | Yes – report SASa | Direct effect | No | Yes - primary results. Adjustment set includes a mediator. (*'According to the theory of DAG, it would be sufficient to adjust the analyses for... physical activity'*) | No |
| Dolatowski *et al.* 2016 | Multivariable regression | No – not explicitly reported | Not reported ('*association*') | N/A | Yes - primary results. *'A minimal adjustment set of covariates was selected using directed acyclic graphs'* | No |
| Downes *et al.* 2015 | Multivariable regression | No - reported adjustment set is after variable reduction methods have been applied | Not reported ('*association*') | N/A | Yes – primary results. Adjustment set built with variable reduction | Yes - covariate retention based on change-in-estimate criteria |
| Dusingize *et al.* 2017 | Multivariable regression | Yes – report SASa | Total effect | Yes - primary results | No | No |
| Dzhambov & Dimitrova 2015 | Multivariable regression | Yes – report SASa | Total and direct effects | Yes - primary results | Yes - primary results. Stratification by *'residence at the current address'* | No |
| Dzhambov & Dimitrova 2016 | Multivariable regression | Yes – report SASa | Total effect | No | Yes - primary results. Adjustment set included additional variables. *'We additionally forced into the models (as proxies for participants’ socioeconomic status) the number of household members and the age at which full-time education was finished'* | No |
| Eichler *et al.* 2016 | Multivariable regression | No – adjustment set(s) stated, but relationship to DAG(s) and/or SASa is not explicit | Not reported (*'relation'*) | N/A | Yes - primary results. *'Potentially confounding variables were integrated using Directed Acyclic Graph'* | No |
| Ellison & De Wet 2016 | Multivariable regression | No – not explicitly reported | Not reported ('*association*') | N/A | Yes - primary results. *'A causal path model was then developed… to distinguish between confounders and mediators in any relationships between each of the sociodemographic and socioeconomic variables and health, assuming that only variables relating to pre-existing characteristics and past events could act as potential con- founders'* | No |
| Emeny *et al.* 2014 | Multivariable regression | Yes – report SASa | Not reported  ('*direct association*') | Yes - primary results | Yes - primary results include *'increasing confounder adjustment'* | No |
| Erkal *et al.* 2009 | Multivariable regression | No – adjustment set(s) stated in table footer(s), but relationship to DAG(s) and/or SASa is not explicit | Not reported ('*association*') | N/A | Yes - primary results. *'Selection of confounders for each exposure of interest/respective model was based on directed acyclic graphs'* | No |
| Escobar *et al.* 2017 | Multivariable regression | Yes – report SASa | Total and direct effects | Yes - primary results | No | No |
| Evandt *et al.* 2017 | Multivariable regression | Yes – though not stated as SASa | Not reported ('*association*') | No | Yes - primary results. Socio-economic factors (educational level and household income) were added to the DAG-implied set | No |
| Evandt *et al.* 2017A | Multivariable regression | Yes – report SASa | Not reported ('*association*') | No | Yes - primary results. Socio-economic factors (educational level and household income) were added to the DAG-implied set | No |
| Fan *et al.* 2016 | Multivariable regression | Yes – report SASa | Not reported ('*association*') | Yes - primary results | No | No |
| Feda *et al.* 2010 | Multivariable regression | No – adjustment set(s) stated in table footer(s), but relationship to DAG(s) and/or SASa is not explicit | Not reported  ('*impact*') | N/A | Yes - primary results. *'Directed acyclic graphs (DAGs) served as a visual guide to identify variables pertinent to the multivariate models and potential confounders on the outcome of interest... During the analysis, each policy used a separate analytical model, based on the assumptions illustrated in the DAG'* | No |
| Ferraro *et al.* 2017 | Multivariable regression | No – adjustment set(s) stated in table footer(s), but relationship to DAG(s) and/or SASa is not explicit | Not reported ('*association*') | N/A | Yes - primary results. *'Covariates were selected with the aid of a directed acyclic graph (DAG)...Each variable was ordered according to its specific temporal relationship...We then built pathways based on temporality and theoretical assumptions'* | No |
| Ferraro *et al.* 2017A | Multivariable regression | No – adjustment set(s) stated in table footer(s), but relationship to DAG(s) and/or SASa is not explicit | Not reported ('*association*') | N/A | Yes - primary results. *'Confounders for which models were adjusted were chosen based on the assumption that they must be antecedent of exposure and outcome'* | No |
| Figueiredo *et al.* 2017 | Multivariable regression | Yes – report SASa | Not reported ('*association*') | Yes - primary results | No | No |
| Filippidis *et al.* 2017 | Multivariable regression | No – adjustment set(s) stated, but relationship to DAG(s) and/or SASa is not explicit | Not reported ('*association*') | N/A | Yes - primary results. *'We have drawn a detailed directed acyclic graph with DAGitty to illustrate potential causal pathways from cigarette prices to infant mortality, identify potential sources of confounding, and guide covariate selection'* | No |
| Filteau *et al.* 2016 | Multivariable regression | Yes – report SASa | Direct effect | Yes - primary results | No | No |
| Frederiksen *et al.* 2009 | Mediation analysis by multivariable regression | Yes – though not stated as SASa | Total and direct effects | Yes - primary results | Yes- primary results of direct effect, several models reported with different adjustment sets. | No |
| Gagliardi *et al.* 2009 | Multivariable regression | No – adjustment set(s) stated, but relationship to DAG(s) and/or SASa is not explicit | Described as '*direct' effect*', but appears to be total effect | N/A | Yes - primary results. *'We adjusted for the possible confounders listed in Table 1 and in Fig. 1. This was done in successive steps, to help understand the effect of various adjustments'* | No |
| Gascon *et al.* 2015 | Multivariable regression | Yes – though not stated as SASa | Not reported ('*association*') | Yes - primary results | No | No |
| Gaspar *et al.* 2017 | TMLEb following machine learning | No – not explicitly reported | Total effect written as E[E(Y|A=1,W)-E(Y|A=0,W)] | N/A | Yes - primary results. Adjustment sets built using an ensamble machine learning algorithm. | Yes - covariate selection by an ensemble machine learning algorithm |
| Gerberich *et al.* 2011 | Multivariable regression | No – adjustment set(s) stated in table footer(s), but relationship to DAG(s) and/or SASa is not explicit | Not reported ('*association*') | N/A | Yes - primary results. *'Directed acyclic graphs (DAGs) were derived from the causal model and used to select the minimum sufficient set of potential confounders for the relevant exposures'* | No |
| Gerberich *et al.* 2014 | Multivariable regression | Yes – report SASa | Not reported  (*'risks'*) | Yes - primary results | No | No |
| Gillam *et al.* 2017 | Multivariable regression | No - reported adjustment set is after variable reduction methods have been applied | Not reported ('*association*') | N/A | Yes - primary resuts. Adjustment sets built with variable reduction. | Yes – change in estimate criteria |
| Gillott *et al.* 2017 | Multivariable regression | Yes – report SASa | Direct effect | Yes - primary results | No | No |
| Gocke *et al.* 2014 | Multivariable regression | Yes – report SASa | Not reported ('*association*') | Yes - primary results | Yes - primary results of three other adjustment sets are reported alongside DAG-implied set | No |
| Gray *et al.* 2017 | Multivariable regression | No – not explicitly reported | Not reported ('*association*') | N/A | Yes - primary results. Adjustment sets built with variable reduction. | Yes - covariate retention based on p-value criteria |
| Gray *et al.* 2017A | Multivariable regression | Yes – report SASa | Not reported ('*association*') | No | Yes - primary results include additional 'exposures of interest' added to the SASa | Yes - covariates 'assessed individually' (unclear) |
| Grice *et al.* 2007 | Multivariable regression | No – adjustment set(s) stated in table footer(s), but relationship to DAG(s) and/or SASa is not explicit | Not reported  (*'effect'*) | N/A | Yes - primary results. '*A priori causal models and directed acyclic graphs (DAGs) guided selection of potentially confounding variables'* | No |
| Grice *et al.* 2010 | Multivariable regression | No – adjustment set(s) stated in table footer(s), but relationship to DAG(s) and/or SASa is not explicit | Not reported ('*associated*') | N/A | Yes - primary results. *'A priori causal models and directed acyclic graphs (DAGs) guided selection of potentially confounding covariates'* | No |
| Grill *et al.* 2014 | Multivariable regression | Yes – report SASa | Not reported  (*'effect'*) | Yes - primary results | No | No |
| Grundy *et al.* 2017 | Multivariable regression | Yes – report SASa | Not reported ('*associations*') | Yes - primary results | No | No |
| Guimarães *et al.* 2016 | Multivariable regression | Yes – report SASa | Total and direct effects | Yes - primary results | Yes - primary results of direct effect. Adjustment set built with variable reduction. | Yes - covariate selection based on p-value criteria |
| Gunathilake *et al.* 2016 | Mediation analysis with multivariable regression | Yes – report SASa | Total and direct effects | Yes - primary results | No | No |
| Harris-Adamson *et al.* 2016 | Multivariable regression | No - reported adjustment set is after variable reduction methods have been applied | Not reported ('*association*') | N/A | Yes – primary results. Adjustment set built with variable reduction | Yes - covariate selection based on change-in-estimate criteria |
| Harskamp-van Ginkel *et al.* 2015 | Mediation analysis with multivariable regression | Yes – report SASa | Total and indirect effects | Yes - primary results | Yes - primary results of one other adjustment set are reported alongside DAG-implied set | No |
| Harville *et al.* 2017 | Multivariable regression | No – adjustment set(s) stated, but relationship to DAG(s) and/or SASa is not explicit | Not reported ('*association*') | N/A | Yes - primary results. *'Three sets of models were examined. The first was unadjusted...The second included covariates identified based on a directed acyclic graph (DAG) using the DAGitty program... The final set of models includes the BMI adjustment.*' | No |
| Herich *et al.* 2017 | Mediation analysis with multivariable regression | No – adjustment set(s) stated, but relationship to DAG(s) and/or SASa is not explicit | Total and direct effects | N/A | Yes - primary results. *'To identify the covariates to be included in the model, we drafted a causal diagram (directed acyclic graph) representing the hypothesized causal relations relevant to our main research question'* | No |
| Hickson *et al.* 2017 | Multivariable regression | Yes – report SASa | Not reported ('*association*') | Yes - primary results | No | No |
| Hinkle *et al.* 2013 | Multivariable regression | Yes – report SASa | Not reported ('*association*') | No | Yes - primary results include competing exposures. Sensitivity analysis conducted with children's weight status at kindergarten entry. | No |
| Hirsch *et al.* 2016 | Multivariable regression | No – adjustment set(s) stated, but relationship to DAG(s) and/or SASa is not explicit | Not reported ('*association*') | N/A | Yes - primary results. *'We selected model covariates (age, gender, education, and having access to a vehicle) using a Directed Acyclic Graph (DAG) and a priori knowledge on sociodemographic and resource characteristics that act as confounders'* | No |
| Hirsch *et al.* 2017 | Multivariable regression | No – adjustment set(s) stated, but relationship to DAG(s) and/or SASa is not explicit | Not reported ('*association*') | N/A | Yes - primary results. *'Adjustment for confounding variables was determined a priori using first Directed Acyclic Graphs... and then statistical tests of associations with both the exposure and outcomes'* | No |
| Hoyer *et al.* 2015 | Multivariable regression | No – adjustment set(s) stated, but relationship to DAG(s) and/or SASa is not explicit | Not reported ('*association*') | N/A | Yes - primary results. '*Directed acyclic graphs (DAGs) were used to select the confounders included in the final models using DAGitty software... All final multivariate models were adjusted for...'* | No |
| Jacobson *et al.* 2017 | Multivariable regression | No - reported adjustment set is after variable reduction methods have been applied | Not reported  (*'effect'*) | N/A | Yes – primary results. Adjustment set built with variable reduction | Yes - covariate selection based on change-in-estimate criteria |
| Jämsä *et al.* 2017 | Multivariable regression | Yes – report SASa | Not reported (*'relationship'*) | Yes - sensitivity analysis | Yes - primary results. Adjustment set included additional variables '*according to clinical experience... and because these variables were interesting for our study hypothesis.'* | No |
| Janitz *et al.* 2017 | Multivariable regression | No - reported adjustment set is after variable reduction methods have been applied | Not reported ('*association*') | N/A | Yes – primary results. Adjustment set built with variable reduction | Yes - covariate selection based on change-in-estimate criteria |
| Jankowiak *et al.* 2016 | Multivariable regression | No – adjustment set(s) stated, but relationship to DAG(s) and/or SASa is not explicit | Not reported ('*association*') | N/A | Yes - primary results. *'Based upon this literature overview, relevant sets of confounders for night shift work and the cardiovascular outcomes were identified using acyclic directed graphs'* | No |
| Johri *et al.* 2016 | Multivariable regression | Yes – report SASa | Not reported ('*association*') | Yes - primary results | Yes - primary results of two other adjustment set are reported alongside DAG-implied set | No |
| Jonker *et al.* 2012 | IPWc of MSMd | Yes – report SASa | Not reported (*'relationship'*) | Yes - primary results | No | No |
| Jusko *et al.* 2010 | Multivariable regression | Yes – report SASa | Not reported (*'association'*) | Yes - primary results | No | No |
| Kalapatapu *et al.* 2017 | Multivariable regression | No – adjustment set(s) stated, but relationship to DAG(s) and/or SASa is not explicit | Not reported  ('*causal effect*') | N/A | Yes - primary results. *'The variables selected for inclusion in the adjusted analyses were based on the previous publication on this topic and depicted using a directed acyclic graph'* | No |
| Kalapatapu *et al.* 2017A | Multivariable regression | No – adjustment set(s) stated, but relationship to DAG(s) and/or SASa is not explicit | Not reported  ('*causal effect*') | No | Yes - primary results. *'We used a directed acyclic graph (DAG)-based approach to select the variables to be included in the regression models.. We conceptualized age, education, sex, race, socioeconomic status... and smoking to confound the association... most of these variables were adjusted for in the regression models '* | No |
| Karahalios *et al.* 2014 | Multivariable regression | No – adjustment set(s) stated, but relationship to DAG(s) and/or SASa is not explicit | Not reported ('*association*') | N/A | Yes - primary results. *'A causal diagram was used to choose confounding variables; these were...'* | No |
| Karahalios *et al.* 2016 | Multivariable regression | No – adjustment set(s) stated, but relationship to DAG(s) and/or SASa is not explicit | Not reported ('*association*') | N/A | Yes - primary results. *'A causal diagram was developed and the following confounding variables were included...'* | No |
| Karlsen *et al.* 2017 | Multivariable regression | No – adjustment set(s) stated, but relationship to DAG(s) and/or SASa is not explicit | Not reported ('*association*') | No | Yes - primary results include additional covariates | Yes - covariate selection based on change-in-estimate criteria |
| Kebede *et al.* 2017 | Mediation analysis with multivariable regression | Yes – report SASa | Total and direct effects | Yes - primary results | Yes – mediation analysis conducted with further adjustment for mediator (*'incident diabetes'*) | No |
| Kendrick *et al.* 2016 | Multivariable regression | No – adjustment set(s) stated, but relationship to DAG(s) and/or SASa is not explicit | Not reported ('*association*') | N/A | Yes - primary results. *'Odds ratios... were estimated using conditional logistic regression adjusted for neighborhood deprivation, distance from hospital, and confounders identified from DAGs'* | No |
| Kerschberger *et al.* 2012 | Multivariable regression | No – adjustment set(s) stated in table footer(s), but relationship to DAG(s) and/or SASa is not explicit | Not reported  (*'effect'*) | N/A | Yes - primary results. *'Potential confounders were determined a priori using directed acyclic graphs... and were included in a multivariate proportional hazards Cox regression model'* | No |
| Khalifa *et al.* 2016 | Multivariable regression | No - reported adjustment set is after variable reduction methods have been applied | Direct effect | N/A | Yes - primary results. Adjustment set built using variable reduction. | Yes - covariate selection based on p-value criteria |
| Kharmats *et al.* 2014 | Multivariable regression | Yes – report SASa | Not reported (*'relation'*) | Yes - primary results | No | No |
| Kim *et al.* 2013 | Multivariable regression | Yes – report SASa | Not reported (*'association'*) | Yes - primary results | Yes - primary results were also stratified by use of NSAIDs | No |
| Kim *et al.* 2015 | Multivariable regression | No – adjustment set(s) stated, but relationship to DAG(s) and/or SASa is not explicit | Not reported ('*relations*') | N/A | Yes - primary results. *'Multiple linear regression models were used to adjust for the following potential confounding factors identified using a directed acyclic graph (DAG) analysis'* | No |
| Klassen *et al.* 2014 | Multivariable regression | No – adjustment set(s) stated, but relationship to DAG(s) and/or SASa is not explicit | Not reported (*'association'*) | N/A | Yes - primary results. Adjustment set built with variable reduction from list of candidates variables identified '*on the basis of the theory of directed acyclic graphs'* | Yes - covariate selection based on change-in-estimate criteria (variables remained unchainged) |
| Kobayashi *et al.* 2017 | Mediation analysis by multivariable regression | No – adjustment set(s) stated, but relationship to DAG(s) and/or SASa is not explicit | Total, direct, and indirect effects | N/A | Yes - primary results. *'Covariates were selected based on associations between dependent and independent variables observed in our data or previous studies...We additionally implemented directed acyclic graphs... for covariate selection and confirmed that the covariates selected above were neither colliders nor intermediates'* | No |
| Kowall *et al.* 2016 | Multivariable regression | Yes – report SASa | Not reported (*'association'*) | Yes - primary results | Yes - primary results also include adjustment for a subset of the SASa | No |
| Kowall *et al.* 2016A | Multivariable regression | Yes – report SASa | Not reported (*'association'*) | Yes - primary results | No | No |
| Kroenke *et al.* 2016 | Multivariable regression | No – adjustment set(s) stated, but relationship to DAG(s) and/or SASa is not explicit | Not reported  (*'effect'*) | N/A | Yes - primary results. *'Potential confounding variables in models were selected based on subject matter expertise encoded in directed acyclic graphs...Adjustment for chemotherapy and radiation were not suggested by the directed acyclic graph... We nonetheless included these variables in models based on convention'* | No |
| Kverneng Hultberg *et al.* 2017 | Multivariable regression | No – adjustment set(s) stated, but relationship to DAG(s) and/or SASa is not explicit | Total effect | N/A | Yes - primary results. *'The covariates included in the adjustment set were determined from a causal diagram... using directed acyclic graphs'* | No |
| Lai *et al.* 2016 | Multivariable regression | No – adjustment set(s) stated in table footer(s), but relationship to DAG(s) and/or SASa is not explicit | Not reported  (*'effect'*) | N/A | Yes - primary results. *'We used the causal diagram to decide which variables to include in the multivariable model'* | No |
| Launay *et al.* 2014 | Multivariable regression | No – not explicitly reported | Not reported  (*'effect'*) | N/A | Yes - primary results. *'Relevant variables according to the causal diagram were included in multivariate analyses'* | No |
| Lehnich *et al.* 2016 | Multivariable regression | Yes – report SASa | Not reported  (*'effect'*) | Yes - primary results | No | No |
| Liang *et al.* 2017 | Multivariable regression | Yes – report SASa | Not reported (*'association'*) | Yes - primary results | No | No |
| Liebers *et al.* 2011 | Multivariable regression | Yes – report SASa | Direct effect | Yes - primary results | No | No |
| Lima *et al.* 2017 | Multivariable regression | Yes – report SASa | Not reported (*'association'*) | Yes - primary results | No | No |
| Linde *et al.* 2017 | Multivariable regression | No – adjustment set(s) stated, but relationship to DAG(s) and/or SASa is not explicit | Not reported (*'association'*) | N/A | Yes - primary results. Adjustment set built using variable reduction. | Yes - covariate retention based on p-value criteria |
| Lupattelli *et al.* 2015 | Multivariable regression | Yes – report SASa | Total and direct effects | Yes - primary results | Yes - sensitivity analyses include adjusting for a mediator (BMI at conception) *'because of the uncertainty in the direction of the association'*) | No |
| Lytsy *et al.* 2013 | Multivariable regression | No – adjustment set(s) stated, but relationship to DAG(s) and/or SASa is not explicit | Not reported (*'association'*) | N/A | Yes - primary results. *'In order to minimize potential bias, the directed acyclic graph approach...was used to identify appropriate models'* | No |
| Magadi & Magadi 2017 | Multivariable regression | No – adjustment set(s) stated in table footer(s), but relationship to DAG(s) and/or SASa is not explicit | Not reported (*'association'*) | N/A | Yes - primary results. Two models built by *'introducing various background demographic and socioeconomic characteristics ... and proximate factors ...use in the models in successive stages to investigate potential pathways of the relationships'* | No |
| Maier *et al.* 2015 | Multivariable regression | No - reported adjustment set is after variable reduction methods have been applied | Not reported  (*'effect'*) | N/A | Yes - primary results. Adjustment set built with variable reduction. | Yes - covariate selection based on change-in-estimate |
| Maika *et al.* 2015 | IPWc of MSMd | Yes – though not stated as SASa | Total effect written as | Yes - primary results | No | No |
| Maretty-Nielsen *et al.* 2014 | Propensity score matching and multivariable regression | No – adjustment set(s) stated in table footer(s), but relationship to DAG(s) and/or SASa is not explicit | Not reported ('*prognostic factors*') | N/A | Yes - primary results. *'The adjustment covariates were selected based on a modified version... of a directed acyclic graph constructed by Maretty-Nielsen et al. and included as seen in Tables 1 and 2'* | No |
| Maretty-Nielsen *et al.* 2014A | Multivariable regression | No – adjustment set(s) stated in table footer(s), but relationship to DAG(s) and/or SASa is not explicit | Not reported ('*estimate*') | N/A | Yes - primary results. *'Directed acyclic graphs were used to depict a possible causal relationship between the prognostic factors selected, possible confounding variables, and the outcomes'* | No |
| Martin *et al.* 2017 | Multivariable regression | Yes – though not stated as SASa | Not reported  (*'objective was to assess differences'*) | Yes - primary results | No | No |
| Matser *et al.* 2013 | Multivariable regression | No – adjustment set(s) stated, but relationship to DAG(s) and/or SASa is not explicit | Not reported  ('*main route*', '*direct route*', '*indirect route*') | N/A | Yes - primary results. *'We constructed a causal DAG in which to map the assumed pathways between ethnicity and CT diagnosis'* | No |
| McCulloh *et al.* 2015 | Multivariable regression | No – not explicitly reported | Not reported (*'association'*) | N/A | Yes - primary results. *'Models used subject-matter knowledge from published literature and a priori clinical assumptions, analyzed by directed acyclic graphs (DAG), to guide statistical modeling assumptions.'* | No |
| Mebrahtu *et al.* 2015 | Multivariable regression | Yes – report SASa | Not reported  (*'effect'*) | Yes - primary results | No | No |
| Medenwald *et al.* 2014 | Multivariable regression | Yes – report SASa | Total effect | Yes - primary results | No | No |
| Medenwald *et al.* 2015 | Mediation analysis by multivariable regression | No – adjustment set(s) stated, but relationship to DAG(s) and/or SASa is not explicit | Total, direct, and indirect effects | N/A | Yes - primary results. *'Considering directed acyclic graphs, we adjusted models of the education–anthropometric parameter association... for the parameters listed in Table 1'* | No |
| Medenwald *et al.* 2016 | Multivariable regression | No – adjustment set(s) stated, but relationship to DAG(s) and/or SASa is not explicit | Not reported ('*prognostic impact*') | N/A | Yes - primary results. *'Covariates were identified using directed acyclic graphs'* | No |
| Medenwald *et al.* 2016A | Mediation analysis by multivariable regression | No – adjustment set(s) stated, but relationship to DAG(s) and/or SASa is not explicit | Total, direct, and indirect effects | N/A | Yes - primary results. *'Variable selection and modelling was based on directed acyclic graphs'* | No |
| Messerlian *et al.* 2017 | Multivariable regression | Yes – though not stated as SASa | Not reported (*'association'*) | Yes - primary results | No | No |
| Møller *et al.* 2016 | Multivariable regression | No – adjustment set(s) stated, but relationship to DAG(s) and/or SASa is not explicit | Not reported (*'association'*) | N/A | Yes - primary results. Adjustment set includes a mediator. (*'According to the theory of DAG, it would be sufficient to adjust the analyses for... physical activity'*) | No |
| Mook *et al.* 2016 | Multivariable regression | Yes – report SASa | Not reported (*'association'*) | Yes - sensitivity analysis | Yes - primary results. Adjustment set built with variable reduction. | Yes - covariate retention based on p-value criteria |
| Murphy *et al.* 2015 | Multivariable regression | Yes – report SASa | Not reported (*'relationship'*) | Yes - primary results | No | No |
| Napier *et al.* 2017 | Multivariable regression | No – adjustment set(s) stated, but relationship to DAG(s) and/or SASa is not explicit | Not reported (*'association'*) | N/A | Yes - primary results. *'We used directed acyclic graphs (visualized using DAGity) to evaluate potential confounding factors plausibly associated with poor water quality and illness'* | No |
| Ng *et al.* 2017 | Mediation analysis with multivariable regression | Yes – report SASa | Total, direct, and indirect effects | Yes - primary results | No | No |
| Ng *et al.* 2017A | Multivariable regression | Yes – report SASa | Total effect | No | Yes - primary results include the SASa in which *'defined daily doses (of medication)'* was substituted for *'sleep problems prior to treatment'* | No |
| Ngueta *et al.* 2016 | Multivariable regression | Yes – report SASa | Not reported (*'relation'*) | Yes - primary results | Yes - primary results include an additional adjustment set, however reasons are unclear | No |
| Nobre *et al.* 2016 | Multivariable regression | Yes – report SASa | Not reported (*'association'*) | Yes - primary results | No | No |
| Norris *et al.* 2017 | Multivariable regression | Yes – report SASa | Not reported (*'association'*) | Yes - primary results | No | No |
| Nourbakhsh *et al.* 2016 | Multivariable regression | No – adjustment set(s) stated, but relationship to DAG(s) and/or SASa is not explicit | Not reported (*'association'*) | No | Yes - primary results. *'We used a directed acyclic graph (DAG) to decide about the confounding factors that had to be adjusted for in the models'* | No |
| Nowak *et al.* 2016 | Multivariable regression | Yes – though not stated as SASa | Not reported (*'association'*) | No | Yes - primary results. Adjustment set built with variable reduction from a larger set of variables. | Yes - covariate retention based on change-in-estimate criteria |
| Nygaard *et al.* 2014 | Multivariable regression | Yes – though not stated as SASa | Not reported (*'association'*) | N/A | Yes - primary results. Adjusted set included additional variables *'based on past literature, which was permissible per the DAG'* | No |
| Nygaard *et al.* 2015 | Multivariable regression | Yes – though not stated as SASa | Not reported (*'association'*) | N/A | Yes - primary results. Adjusted set included additional variables *'based on past literature, which was permissible per the DAG'* | No |
| Oddo *et al.* 2017 | Multivariable regression | Yes – report SASa | Not reported (*'association'*) | No | Yes - primary results. DAG appeared to imply an empty adjustment set so *'the fully adjusted model controlled for a priori defined variables'* | No |
| Oddo *et al.* 2017A | Multivariable regression | No – adjustment set(s) stated, but relationship to DAG(s) and/or SASa is not explicit | Not reported (*'association'*) | N/A | Yes - primary results. *'We identified confounding factors a priori using a directed acyclic graph, which is a causal diagram used to characterize the relationship between the exposure and outcome based on theorized relationships and relationships documented in the literature'* | No |
| Olsen *et al.* 2015 | Multivariable regression | No – adjustment set(s) stated, but relationship to DAG(s) and/or SASa is not explicit | Not reported (*'association'*) | N/A | Yes - primary results. *'We identified confounding factors a priori using a directed acyclic graph, which is a causal diagram used to characterize the relationship between the exposure and outcome based on theorized relationships and relationships documented in the literature'* | No |
| Olsson *et al.* 2017 | Multivariable regression | No – adjustment set(s) stated in table footer(s), but relationship to DAG(s) and/or SASa is not explicit | Not reported (*'association'*) | N/A | Yes - primary results included three models, the first including *'age, gender and period of diagnosis'*, the second included *'relevant socioeconomic factors according to the causal diagram' and the third 'were further adjusted for comorbidity'* | No |
| O'Neill *et al.* 2016 | Multivariable regression | No – adjustment set(s) stated in table footer(s), but relationship to DAG(s) and/or SASa is not explicit | Not reported (*'association'*) | N/A | Yes - primary results. *'Hypothesised confounders and mediators were identified in a causal diagram'* | No |
| Orban *et al.* 2016 | Multivariable regression | No – adjustment set(s) stated, but relationship to DAG(s) and/or SASa is not explicit | Not reported (*'association'*) | N/A | Yes - primary results. Three adjustment sets reported *'were selected a priori based on a directed acyclic graph'* | No |
| Osler *et al.* 2015 | Multivariable regression | Yes – though not stated as SASa | Not reported (*'association'*) | Yes - primary results | Yes - primary results. Results of a second adjustment set (including mediators) is presented alongside DAG-implied set. | No |
| Oswald *et al.* 2017 | Multivariable regression | No - reported adjustment set is after variable reduction methods have been applied | Not reported (*'association'*) | N/A | Yes - primary results. *'We used a sequential modelling approach to explore confounding –indicated by change in exposure estimates – and changes in residual variance'* | Yes - covariate selection based on change-in-estimate criteria |
| Oswald *et al.* 2017A | Multivariable regression | No - reported adjustment set is after variable reduction methods have been applied | Not reported (*'association'*) | N/A | Yes – primary results. Adjustment set built with variable reduction | Yes - covariate selection based on change-in-estimate criteria |
| Pattloch *et al.* 2017 | Machine learning and multivariable regression | No – adjustment set(s) stated in table footer(s), but relationship to DAG(s) and/or SASa is not explicit | Not reported ('assoziiert' [associated]) | N/A | Yes - primary results. *'Die notwendigen Adjustierung Variablen wurden mit gerichteten azyklischen Graphen ausgewählt, unterstützt durch das webbasierte Hilfsmittel DAGitty'* [The necessary adjustment variables were selected with directed acyclic graphs, supported by the web-based tool DAGitty] | Yes - covariate selection by a machine learning algorithm |
| Paulson *et al.* 2006 | Multivariable regression | No – adjustment set(s) stated in table footer(s), but relationship to DAG(s) and/or SASa is not explicit | Not reported ('*magnitude and consequences*') | N/A | Yes - primary results. *'Selection of confounders for each exposure of interest was based on directed acyclic graphs'* | No |
| Phillips *et al.* 2017 | Multivariable regression | Yes – report SASa | Not reported (*'association'*) | Yes - primary results | No | No |
| Pink *et al.* 2015 | Multivariable regression | Yes – report SASa | Not reported  (*'effect'*) | Yes - primary results | Yes - primary results also include a subset of the SASa | No |
| Preußel *et al.* 2015 | Multivariable regression | No – adjustment set(s) stated in table footer(s), but relationship to DAG(s) and/or SASa is not explicit | Not reported (*'association'*) | N/A | Yes - primary results. Adjustment set built using variable reduction. | Yes - covariate selection based on p-value criteria |
| Protudjer *et al.* 2015 | Multivariable regression | Yes – though not stated as SASa | Not reported (*'association'*) | Yes - sensitivity analysis | Yes - primary results are not adjusted for one of the stated confounders (BMI z-score) | No |
| Pyko *et al.* 2015 | Multivariable regression | Yes – report SASa | Total effect | Yes - primary results | Yes - sensitivity analysis include *'adjustment for contextual confounding, other sources of noise as well as air pollution from local road traffic'* | No |
| Pyko *et al.* 2017 | Multivariable regression | No – adjustment set(s) stated, but relationship to DAG(s) and/or SASa is not explicit | Direct effect | N/A | Yes - primary results. *'The covariates evaluated as confounders were identified based on a literature search and by development of a directed acyclic graph'* | No |
| Rajappan *et al.* 2017 | Multivariable regression | Yes – report SASa | Direct effect and '*overall impact*' | Yes - primary results | Yes – two secondary analyses; one adjusting for a further confounder (*'paternal BMI'*) to capture unobserved confounding and one for *'potential postnatal mediators'* to estimate direct effects. | No |
| Rancière *et al.* 2017 | Multivariable regression | Yes – report SASa | Direct effect | Yes - primary results | No | No |
| Ratanawongsa *et al.* 2013 | Multivariable regression | Yes – though not stated as SASa | Direct effect | Yes - primary results | No | No |
| Rebelo *et al.* 2016 | Multivariable regression | Yes – report SASa | Direct effect | Yes - primary results | No | Yes - covariate selection based on p-value and change-in-estimate criteria (however SASa model remained unchanged) |
| Rebelo *et al.* 2016A | Multivariable regression | Yes – report SASa | Direct effect | Yes - primary results | No | Yes - covariate selection based on p-value and change-in-estimate criteria (however SASa model remained unchanged) |
| Rêgo *et al.* 2016 | Multivariable regression | Yes – report SASa | Not reported (*'association'*) | Yes - primary results | No | No |
| Reiner *et al.* 2016 | Multivariable regression | No – adjustment set(s) stated in table footer(s), but relationship to DAG(s) and/or SASa is not explicit | Not reported (*'association'*) | N/A | Yes - primary results. *'An a priori causal model or directed acyclic graph... was used to identify potential confounders for each exposure'* | No |
| Rhea *et al.* 2014 | Multivariable regression | No – not explicitly reported | Not reported (*'association'*) | N/A | Yes - primary results. *'Directed acyclic graph analysis indicated that for each risk factor, the model should be adjusted for all other risk factors and for patient age'* | No |
| Ribeiro *et al.* 2017 | Multivariable regression | Yes – report SASa | Total effect | Yes - primary results | No | No |
| Risch *et al.* 2014 | Multivariable regression | Yes – report SASa | Not reported (*'association'*) | Yes - primary results | No | No |
| Rusconi *et al.* 2011 | Multivariable regression | No – adjustment set(s) stated, but relationship to DAG(s) and/or SASa is not explicit | Not reported (*'association'*; '*causal effect*') | N/A | Yes - primary results. *'We also try to explore the relationship between paracetamol and antibiotics administration and wheezing by using causal diagrams'* | No |
| Rutegård *et al.* 2016 | Multivariable regression | Yes – report SASa | Not reported  ('*impact*') | Yes - primary results | No | No |
| Rutegård *et al.* 2016A | Multivariable regression | Yes – report SASa | Not reported (*'association'*) | No | Yes - primary results of an adjustment set without age and sex. These were included in a sensitivity analyses as *'using this adjustment set, the variables sex and age were redundant'* | No |
| Sage *et al.* 2010 | Multivariable regression | No – adjustment set(s) stated in table footer(s), but relationship to DAG(s) and/or SASa is not explicit | Not reported ('*relations*') | N/A | Yes - primary results. *'Directed acyclic graphs (DAGs) were developed for each of the exposures of interest to facilitate selection of potential confounding variables for control'* | No |
| Salti *et al.* 2017 | Multivariable regression | Yes – report SASa | Not reported (*'association'*) | Yes - primary results | No | No |
| Samson *et al.* 2016 | Multivariable regression | No – not explicitly reported | Not reported ('*estimating direct effects*') | N/A | Yes - primary results. 'Nonmodifiable risk factors were fit in the model to reduce the effect of confounding' | No |
| Schipf *et al.* 2011 | Multivariable regression | Yes – report SASa | Total effect | Yes - primary results | No | No |
| Schliep *et al.* 2011 | Multivariable regression | Yes – report SASa | Not reported (*'association'*) | Yes - primary results | Yes - secondary analysis; adjusting for additional covariates without clear explanation. | No |
| Schmidt *et al.* 2013 | Multivariable regression | No – adjustment set(s) stated in table footer(s), but relationship to DAG(s) and/or SASa is not explicit | Not reported (*'associations'*) | N/A | Yes - primary results. *'Analyses were stratified by study center and adjusted for covariables selected on the basis of the theory of directed acyclic graphs'* | No |
| Schwahn *et al.* 2013 | Multivariable regression | Yes – report SASa | Not reported (*'relationship'*) | Yes - primary results | No | No |
| Sehrndt *et al.* 2011 | Multivariable regression | Yes – report SASa | Total effect | Yes - primary results | No | No |
| Senkomago *et al.* 2015 | Multivariable regression | No – not explicitly reported | Not reported | N/A | Yes - primary results. *'Potential confounders of the association between circumcision and HPV viral load were identified from the literature and analyzed using a directed acyclic graph'* | No |
| Senkomago *et al.* 2016 | Multivariable regression | No – not explicitly reported | Not reported (*'association'*) | N/A | Yes - primary results. *'Potential confounders were identified from the literature and analyzed using a directed acyclic graph'* | No |
| Seward *et al.* 2015 | Multivariable regression | No – adjustment set(s) stated in table footer(s), but relationship to DAG(s) and/or SASa is not explicit | Not reported (*'association'*) | N/A | Yes - primary results. *'Directed acyclic graphs (DAGs) were used to inform the statistical modelling of the relationships... (and) to ensure that the confounders selected were appropriate'* | No |
| Shah *et al.* 2015 | Multivariable regression | Yes – report SASa | Not reported  ('*overall effect*') | No | Yes - primary results. All models are additionally adjusted for study site as *'a strong independent correlate of these outcomes, and a potential marker for unmeasured confounding'.* | No |
| Shaw *et al.* 2018 | Multivariable regression and meta-analysis | No – adjustment set(s) stated, but relationship to DAG(s) and/or SASa is not explicit | Not reported  ('*impact*') | Yes - primary results | Yes - primary results. *'We used the theoretical framework of pathways between fuel price and health... to inform a causal diagram for the analysis... The causal diagram guided the choice of covariates'* | No |
| Sheikh *et al.* 2014 | Mediation analysis by multivariable regression | Yes – though not stated as SASa | Total, direct, and indirect effects | Yes - primary results | No | No |
| Shoaff *et al.* 2016 | Multivariable regression | No – adjustment set(s) stated, but relationship to DAG(s) and/or SASa is not explicit | Not reported (*'relationship'*) | N/A | Yes - primary results. *'A directed acyclic graph (DAG) was drawn a priori to assess potential confounders associated with both phthalate exposure and birth outcomes'* | No |
| Shoaff *et al.* 2017 | Multivariable regression | No – adjustment set(s) stated, but relationship to DAG(s) and/or SASa is not explicit | Not reported (*'association'*) | N/A | Yes – primary results. *'We used a directed acyclic graph to select covariates and considered maternal sociodemographic, perinatal, nutritional, environmental, and child factors'*. Various secondary and sensitivity analyses performed. | No |
| Skretteberg *et al.* 2013 | Multivariable regression | No - reported adjustment set is after variable reduction methods have been applied | Not reported (*'association'*) | N/A | Yes – primary results. Adjustment set built with variable reduction. Sensitivity analysis adjusted for potential mediator (BMI) '*to obtain results that could be comparable to previous studies'* | Yes – covariate selection based on on p-value criteria and retention based on p-value and change-in-estimate criteria |
| Smallwood *et al.* 2017 | Multivariable regression | No – not explicitly reported | Not reported (*'association'*) | N/A | Yes – primary results. Unclear what was included in *'Fully adjusted'* model, but refers to variables not included in DAG (*'highest educational attainment'* and *'alcohol intake'*) | No |
| Sohn *et al.* 2015 | Propensity score matching following multivariable regression | No – adjustment set(s) stated, but relationship to DAG(s) and/or SASa is not explicit | Not reported (*'associated'*) | N/A | Yes – primary results. '*We used causal diagrams to select important covariates for inclusion…the following covariates were included:'* | No |
| Solmi *et al.* 2017 | Multivariable regression | Yes – report SASa | Total effect | Yes - primary results | No | No |
| Spillane *et al.* 2013 | Multivariable regression | No – not explicitly reported | Not reported (*'associations'*) | N/A | Yes – primary results. *'Prior knowledge, literature review, and causal diagrams were used to identify potential covariates…. The final multivariate model was selected using backward elimination'* | Yes, change-in-estimate criteria |
| Spillane *et al.* 2014 | Multivariable regression | No – not explicitly reported | Not reported (*'associations'*) | N/A | Yes – primary results. *'Prior knowledge, literature review and causal diagrams were used to identify potential covariates…The final multivariate model was then selected using backwards elimination'* | Yes, change-in-estimate criteria |
| Ssewanyana *et al.* 2015 | Multivariable regression | Yes – report SASa | Not reported  (*'effect'*) | Yes - primary results | No | No |
| Starling *et al.* 2014 | Multivariable regression | Yes – report SASa | Not reported (*'association'*) | Yes - primary results | Yes – sensitivity analyses included *'weight gain (kg) from pre-pregnancy to mid-pregnancy…although this variable was not part of the original DAG'* | No |
| Starling *et al.* 2017 | Multivariable regression | Yes – report SASa | Not reported  (*'effect'*) | Yes - primary results | Yes - primary results of two other adjustment sets are reported alongside DAG-implied set | No |
| Stratakis *et al.* 2016 | Multivariable regression and meta-analysis | No – adjustment set(s) stated, but relationship to DAG(s) and/or SASa is not explicit | Not reported (*'association'*) | N/A | Yes – primary results. Adjustment set includes a variable (birthweight) that is depicted as a mediator in their DAG. | No |
| Strutz *et al.* 2014 | Multivariable regression | No – not explicitly reported | Not reported (*'associations'*) | N/A | Yes – primary results. Report results from three adjustment sets with unclear relation to DAG. | No |
| Sunol *et al.* 2015 | Multivariable regression | Yes – though not stated as SASa | Not reported (*'association'*) | Yes - primary results | No | No |
| Sunyer *et al.* 2015 | Multivariable regression | No – adjustment set(s) stated, but relationship to DAG(s) and/or SASa is not explicit | Not reported (*'associated'*) | N/A | Yes - primary results. *'This model was further adjusted for potential confounders selected with directed acyclic graphs. Based on all socio-demographic and contextual covariables mentioned above, we used the program DAGitty 2.0, with a priori definition of the temporal direction of the events, to draw causal diagrams. The final adjusted model (model 2) included additional coefficients for...'* | No |
| Tamimi *et al.* 2017 | Multivariable regression | Yes – though not stated as SASa | Not reported  (*'effect'*) | Yes - primary results | No | No |
| Tassiopoulos *et al.* 2017 | Multivariable regression | No – not explicitly reported | Not reported (*'associations'*) | N/A | Yes – primary results. Adjustment set built with variable reduction. | Yes – covariate selection based on change-in-estimate criteria |
| Tedla & Bautista 2016 | Multivariable regression | Yes – report SASa | Not reported (*'association'*) | No | Yes - primary results. Adjustment set additionally included '*age, sex, and race'*, which had not been depicted as confounders. | No |
| Tedla *et al.* 2017 | Multivariable regression | Yes – though not stated as SASa | Not reported (*'association'*) | Yes - primary results | No | No |
| Tedla *et al.* 2017A | Multivariable regression | Yes – though not stated as SASa | Not reported (*'association'*) | Yes - primary results | Yes - primary results of two other adjustment sets are reported alongside DAG-implied set | No |
| Traeger *et al.* 2016 | Multivariable regression | Yes – though not stated as SASa | Not reported  ('*Main effects*' and '*moderating effects*') | Yes - primary results | Yes – sensitivity analysis performed to explore '*moderating effects*' | No |
| Upson *et al.* 2013 | Multivariable regression | Yes – though not stated as SASa | Not reported (*'association'*) | Yes - primary results | No | No |
| Urquia *et al.* 2011 | Multivariable regression | No – adjustment set(s) stated, but relationship to DAG(s) and/or SASa is not explicit | Not reported  ('*causal effect estimate*') | N/A | Yes – primary results. *'We based our choice of covariates for confounder control based on a theoretical model assisted with the use of directed acyclic graphs (DAGs)'* | No |
| Valvi *et al.* 2015 | Multivariable regression | No – not explicitly reported | Not reported (*'associations'*) | N/A | Yes – primary results. Adjustment set built with variable reduction. | Yes – change-in-estimate criteria |
| Wang & Bautista 2015 | Multivariable regression | Yes – report SASa | Not reported  ('*causal effect*') | Yes - primary results | Yes – some sensitivity analyses performed to explore residual confounding | No |
| Waterhouse *et al.* 2016 | Multivariable regression | No – adjustment set(s) stated, but relationship to DAG(s) and/or SASa is not explicit | Not reported (*'association'*) | N/A | Yes – primary results. '*Directed acyclic graphs (used to) guide selection of potential confounding factors to be included in adjusted models*' | No |
| Webb *et al.* 2015 | Multivariable regression | Yes – though not stated as SASa | Not reported (*'association'*) | Yes - primary results | Yes – sensitivity analysis adjusts for outcome heterogeneity from '*time of blood collection*' | No |
| Wei *et al.* 2016 | Mediation analysis by multivariable regression | No – not explicitly reported | Total, direct, and indirect effects | N/A | Yes – primary results. Adjustment set '*guided by Poundstone et al’s socioepidemiologic framework, literature review, and causal diagrams*' | Yes - covariate selection based on p-value criteria |
| Weyde *et al.* 2017 | Multivariable regression | Yes – report SASa | Not reported (*'association'*) | Yes - preliminary results | Yes – primary results. Adjustment set included '*an additional set of variables…because of their well-established association with exposure and outcome*' | No |
| Weyde *et al.* 2017A | Mediation analysis by multivariable regression | Yes – report SASa | Not reported (*'association'* and '*mediation effect*') | Yes - preliminary results | Yes – primary results. Adjustment set included '*an additional set of covariates… because of their well-established association with exposure and outcome*'. Mediation analysis adjusts for mediator. | No |
| Williams *et al.* 2016 | Multivariable regression | No - reported adjustment set is after variable reduction methods have been applied | Not reported (*'associations'*) | N/A | Yes – primary results. Adjustment set built with variable reduction | Yes – covariate selection based on p-value criteria and retention on p-value and change-in-estimate criteria |
| Wilson *et al.* 2016 | Multivariable regression | Yes – report SASa | Total effect | Yes - primary results | No | No |
| Windsor *et al.* 2016 | Multivariable regression | No – not explicitly reported | Not reported (*'association'*) | N/A | Yes – primary results. Adjustment set built with variable reduction | Yes – covariate selection based on p-value criteria |
| Yau *et al.* 2015 | Multivariable regression | Yes – report SASa | Not reported  (*'effect'*) | Yes - primary results | No | No |
| Yin *et al.* 2014 | Multivariable regression | No – not explicitly reported | Not reported  ('*main effects*') | N/A | Yes – primary results. Adjustment set built with variable reduction | Yes – covariate retention based on p-value and change-in-estimate criteria |
| Zhang *et al.* 2008 | Multivariable regression | No – not explicitly reported | Not reported (*'associated'*) | N/A | Yes – primary results. Jointly report five bespoke adjustment sets | No |
| Zhang *et al.* 2010 | Mediation analysis by IPWc of MSMd | No – not explicitly reported | Total, direct, and indirect effects | N/A | Yes – primary results. Variable selection process is unclear | No |

aSAS = Sufficient adjustment set bTLME = Targeted maximum likelihood cIPW = inverse probability weight dMSM = marginal structural model

# Supplementary Table S5.

Quoted details of the adjustment set(s) and approach to covariate selection for the 234 articles included in the review

| **Citation** | **Approach to covariate selection and details of adjustment set(s)** |
| --- | --- |
| Åkerstedt *et al.* 2017 | **Page 883:**  '*Based on our a priori assumptions on the relationships between confounders, intermediate variables, exposure, and outcome variables, we were able to draw a directed acyclic graph (DAG), with the aim to identify the minimal set of variables to adjust for the total effect (presented as supplementary material). To draw the DAG, the web-based application DAGitty (http://www.dagitty.net) was used. In the multivariable analyses we first controlled for the following potential confounders: sex, body mass index…level of education…smoking status…alcohol consumption…total physical activity…and major diseases…. In addition, two more variables suggested by the DAG were considered, namely occupational status and depression. However, these two variables were not included due to a large proportion of missingness and degree of potential misclassification*'  **Supplementary material:**  '*Minimal sufficient adjustment sets for estimating the total effect of sleep duration on mortality: Age, Alcohol, BMI, Depression, Education, Major diseases, Physical activity, Sex, Smoking, Work.*' |
| Al-Farsi *et al.* 2010 | **Page 1183:**  '*We developed DAGs for all cofactors that emerged from the literature that could be potential confounders in the relation between parity and prediabetes…We found that we needed to adjust for six potential confounding factors: (i) maternal age, (ii) educational status, (iii) family income, (iv) year of delivery, (v) physical activity and (vi) eating disorders. Although BMI and hypertension are considered risk factors for the development of prediabetes and diabetes, we did not adjust for those cofactors because they are unlikely risk factors for HP. Therefore, it was more appropriate to consider it an intermediate in the causal pathway, and made it unnecessary to adjust for it in the analysis*'  '*We could not adjust for physical activity and eating disorders because of lack of sufficient information about those cofactors*'  '*In the full models, we adjusted in addition to maternal age for potential confounding factors resulting from the DAGs: educational level, family income and year of delivery.*' |
| Al-Harbi & El Tantawi 2017 | **Page 4:**  '*We developed a framework (Additional file 1) to visualize the relationship between the study variables using Directed Acyclic Graphs (DAGs) (Fig. 1a and b) plotted by Dagitty (http://dagitty.net/). In the 1st framework, the outcome was the need for prosthodontic care and the exposures were untreated decay and need for periodontal care. Several factors were included because they were related to these two oral diseases and were divided into practices that increase their risk and demographic factors. The other framework showed the association between the impact on daily life and need for prosthodontic care in addition to untreated decay…Univariate regression models were developed to include each exposure/ confounder followed by a multivariable model controlling for the identified confounders*' |
| Andell *et al.* 2014 | **Page 2:**  '*Adjustments for potential confounders were performed stepwise in two models, the first including age, sex, smoking status and comorbidities (previous MI, previous stroke, heart failure, renal failure, hypertension, diabetes, peripheral artery disease, cancer and previous bleeding). The second model also included treatments during hospitalisation and at discharge (heparin, fondaparinux, dalteparin, enoxaparin, GPIIbIIIa-inhibitors, β-blockers, balloon angioplasty, coronary stenting, as well as discharge medications including ACE inhibitors, angiotensin II receptor blockers, aspirin, clopidogrel, prasugrel, β-blockers, calcium channel blockers, digoxin, diuretics, statins, nitrates and warfarin). The selection of covariates included in these models was performed with the use of a direct acyclical graph via a web-based tool (http://www. dagitty.net), as illustrated in online supplementary figure S1*' |
| Andell *et al.* 2015 | **Page 2:**  '*Potential confounders were identified using an a priori direct acyclic graph via a web-based tool (http://www.dagitty.net). The multivariate model included the following covariates: age, sex, smoking status, comorbidities (previous MI, previous stroke, heart failure, renal failure, hypertension, diabetes, and cancer), in-hospital characteristics (STEMI, angiography, coronary stenting), b-blocker therapy at presentation, COPD medication at presentation, and discharge medications (angiotensin-converting enzyme inhibitors, angiotensin-II receptor blockers, aspirin, clopidogrel, statins, calcium channel blockers, and diuretics)*'  **Page 3:**  '*A second adjustment method using a propensity score as a continuous covariate in a Cox proportional hazard model was tested to ascertain whether a different adjustment model would impact the result differently. The propensity score was calculated using a logistic regression model, and using the direct acyclic graph, the following covariates were identified as dependent determinants for the exposure of being discharged with b-blockers: age, sex, smoking status, previous stroke, previous MI, heart failure, diabetes, hypertension, renal failure, cancer, b-blockers therapy at presentation, STEMI, coronary angiography, coronary stenting, and COPD medications at presentation.*' |
| Andersen *et al.* 2014 | **Page 703:**  '*To reduce risk of potential bias, models were identified using directed acyclic graphs based on known risk factors of heart failure, drawn using the web-based application DAGitty (http://www.dagitty.net; Figure III in the Data Supplement). For each exposure (total physical activity and leisure time activity), 3 models were investigated: (1) crude models adjusting for age and sex; (2) models for estimating total causal effects, adjusting for age, sex, education, and previous myocardial infarction; and (3) models for estimating direct causal effects, adjusting for age, sex, alcohol use, BMI, diabetes mellitus, hypertension, myocardial infarction during follow-up, previous myocardial infarction, smoking, snuff use, and waist–hip ratio.*'  **Supplementary material:**  '*Minimal sufficient adjustment sets for estimating the total effect of PA on HF: Education, Previous MI, Sex… Minimal sufficient adjustment sets for estimating the direct effect of PA on HF: Alcohol, BMI, Diabetes, Hypertension, MI_follow_up, Previous_MI, Sex, Smoking, Snuff, WHR*' |
| Andreano *et al.* 2017 | **Page 121:**  '*We constructed a directed acyclic graph (DAG) to represent assumptions regarding the underlying causal relationships between guideline adherence, survival and a set of clinical and socioeconomic variables. The DAG (Online Resource 1) utilizes these assumptions to select the potential confounders, rather than relying on the statistical associations observed in the data at hand. The selected confounders are then used in the statistical analysis aiming at evaluating the ‘causal’ impact of receiving a care adherent to guidelines on survival.*'  **Page 122:**  '*To investigate the ‘causal’ relationship between survival and adherence (dichotomous variable with a 80% cut-off), we fitted an inverse probability weighted (IPW) marginal structural model. After selecting potential confounders based on the DAG, we created a pseudo-population by the use of (stabilized) inverse probability of adherence weights to mitigate the differences between patients with adherent and nonadherent care. These weights were estimated through a multivariable logistic regression model on adherence, including as covariates the potential confounders: molecular type, stage, grade, their first-degree interactions, age (always as a continuous variable with restricted cubic spline functions), Charlson comorbidity index, number of positive nodes, marital status, employment, education, and deprivation index.*' |
| Arora *et al.* 2014 | **Page 645:**  '*A series of multivariable models were developed to better assess the influence of confounding. On the basis of data availability, we focus on confounding by sociodemographic indicators (age, sex, race/ethnicity and educational level), health behaviours (smoking status, caloric intake, alcohol consumption and physical activity) and adiposity (body mass index). We also consider variables that might mediate the association between periodontal infection and pre-diabetes (blood pressure, cholesterol profile, WBC and CRP). Models were additionally informed by two Directed Acyclic Graphs (DAGs) constructed using, Dagitty. Figure 1A assumes more complex causal structures in which potential sociodemographic confounders relate to periodontal infection and pre-diabetes through multiple mechanisms (e.g. confounding can act through health behaviours and adiposity but also through other mechanisms not represented in our data); this causal structure necessitates adjustment for all sociodemographic variables. Alternatively, the DAG in Figure 1B assumes that all confounding effects act through either health behaviours or adiposity; this causal structure does not require adjustment for sociodemographic variables if health behaviours and adiposity adjustments are made…*  '*The following multivariable models were considered. Model 2 adjusted for only health behaviours (smoking status, caloric intake, alcohol consumption and physical activity) and a marker of adiposity (body mass index) based on assumptions inherent in Figure 1B. Model 3 adjusted for only the sociodemographic variables age, sex, race/ethnicity and educational level. Model 4 adjusted for health behaviours and sociodemographic variables. Model 5 adjusted for sociodemographics, health behaviours and adiposity. Finally, model 6 expanded model 5 by additionally adjusting for variables that could be considered as either a confounder or mediator of associations between periodontal infection and pre-diabetes depending on the causal structure hypothesized; this model included adjustment for systolic blood pressure, total cholesterol-to-HDL ratio, WBC and CRP as previous reports suggest periodontal infection as a possible risk factor for these outcomes. Unless otherwise stated, ORs reported in the main text were derived from model 5 as we believe this provides the best combination of parsimony and validity…*  '*To provide additional information regarding the magnitude and direction of confounding by the aforementioned variables considered individually, we also constructed tables summarizing the difference between parameter estimates from logistic models with more versus less covariable adjustment using a* '*change-in-estimate*' *approach; changein- estimate was defined as follows: [(LN (more adjusted OR) LN (less adjusted OR))/LN (adjusted OR)] × 100, yielding the percent change in the OR resulting from lack of adjustment. The approach uses 13 model selection iterations (the number of possible covariables). Iteration 1 started with the unadjusted parameter estimate for periodontal infection and then ran 13 separate regressions considering the influence of all potential confounders on the unadjusted parameter estimate. The confounder that produced the greatest change-in-estimate was added to the regression to form an* '*intermediate*' *model and another modelling iteration was repeated; each modelling iteration increases the number of independent variables in the intermediate model by 1 and decreases the number of remaining confounders by 1. Four variables hypothesized as possible mediators were assessed in the last four interactions. Results from our change-in-estimate analysis did not suggest that the aforementioned models 1–6 would be inappropriate.*' |
| Asgari *et al.* 2011 | **Page 1389:**  '*The causal DAG used in this study is shown in the Figure. We hypothesized causal relationships among variables based on previously published literature about patient satisfaction relative to age, gender, race/ethnicity, socioeconomic variables (education status, income, marital status, and site of care), mental health, comorbidities, preoperative skin-related QOL, tumor-related factors (size, location, invasiveness, recurrence, and type), and postoperative variables. Also hypothesized to influence patient satisfaction and included in the model were intraoperative variables, such as the number of stages, defect size, and repair type, some of which been shown to be associated with long-term cosmesis. Postoperative variables, such as time lost for treatment, bother from bleeding, included in the model because they were deemed likely to influence patient satisfaction. Although it may be possible to draw different DAGs based on varying causal assumptions, we believed that this DAG best captured the causal relationships among the factors based on known temporal ordering of the variables, the existing literature on patient satisfaction, and clinical judgment. According to the assumptions in the DAG, we determined a set of adjusting variables for each exposure and used standard logistic regression analysis of the outcome on the exposure, including all covariates in the adjustment set (no interaction terms were considered). The associations between the exposure and the outcome revealed by our models (ie, the coefficient in front of the exposure variables) rely on the aforementioned causal DAG assumptions, as well as on correct specification of the parametric logistic regression model (eg, the absence of interaction terms between exposures and covariates).*'  **Table 2 (footer):**  '*aModels for each demographic variable were adjusted for the remaining demographic variables. Models for each socioeconomic factor variable were adjusted for the remaining socioeconomic factor variables and all demographic variables. Model for the site-of-care variable was adjusted for all socioeconomic factor variables. Model for previous nonmelanoma skin cancer was adjusted for all demographic and socioeconomic factor variables. Models for each physical health variable were adjusted for the remaining physical health variables and all demographic and socioeconomic factor variables. Model for the 12-Item Short-Form Health Survey mental component score was adjusted for all demographic and socioeconomic factor variables. Models for the preoperative skin quality-of-life variables were adjusted for the remaining preoperative skin quality-of-life variables plus demographic, socioeconomic factor, mental health, and tumor characteristic variables. Models for each tumor characteristic variable were adjusted for the remaining tumor characteristic variables plus demographic, socioeconomic factor, and previous nonmelanoma skin cancer variables. Models for each intraoperative event variable were adjusted for the remaining intraoperative event variables plus tumor characteristic variables. Models for each postoperative variable were adjusted for the remaining postoperative variables plus intraoperative event variables*' |
| Ashley-Martin *et al.* 2015 | **Page 363:**  '*Potential covariates were identified using a causal model. The causal models were constructed using evidence regarding predictors of the exposures and predictors of the immune system biomarkers. The minimal adjustment sets for confounding were identified using DAGitty. Specific gravity was forced into the adjusted phthalate/BPA model to account for heterogeneity in urinary dilution. As childhood allergy prevalence differs by sex, analyses were also stratified by sex.*'  **Table 3 (footer):**  '*a Adjusted for maternal age, specific gravity.b Adjusted for maternal age, sex.*' |
| Bach *et al.* 2015 | **Page 3:**  '*Selection of covariates was based on a DAG (Additional file 1 Fig. 1) and included age (continuous), pre-pregnancy body mass index (BMI, continuous), socio-occupational status (higher versus middle/lower), and parity (primiparous or multiparous). For the pooled analyses of the two samples we additionally adjusted for the sample (by use of a dummy variable assigning each of the two samples with a different value) in order to account for differences in sampling and laboratories.*' |
| Badland *et al.* 2017 | **Page 242:**  '*We constructed two directed acyclic graphs (DAGs) to map hypothesised pathways and temporal contributions between variables. The DAGs are presented in Figs. 2 and 3, and guided our analysis plan. In Fig. 2, local employment was the exposure variable. In Fig. 3, mode access to employment was the exposure variable, with local employment operating as an adjusting variable. All models were adjusted for individual-level sociodemographic confounders, as these were hypothesised to impact on sedentary behaviour (i.e. sex) or self-rated health (i.e. age, marital status, education, income). The conceptual framework suggested the potential importance of ‘mode of travel to work’ and ‘vehicle miles travelled’, and these were included in the DAGs as latent variables, as these data were not collected in the VicHealth Indicators Survey…*  '*Multivariate logistic regression models were used to compare the spatial neighbourhood-level employment measures with the likelihood of reporting longer commuting times, longer sitting time, and poorer selfrated health (based on the median splits). Direct and pathway associations were examined. Neighbourhood-level employment analyses were adjusted for sex, age, education, employment, marital status, household income, and clustering at the local area…*  '*The mode access to employment models were further adjusted for local employment (as indicated in Fig. 3).*'  **Table 3 (footer):**  '*a Models adjusted for sex, age, marital status, education attainment, household income, and clustering at local area b Model adjusted for sex, age, marital status, education attainment, household income, local employment, and clustering at local area*'  **Table 4 (footer):**  '*a Models adjusted for sex, age, marital status, education attainment, household income, and clustering at local area*' |
| Bahls *et al.* 2017 | **Page 3:**  '*A directed acyclic graph was used to determine potential confounding and appropriately adjust for age, sex, previous myocardial infarction, physical inactivity and a potential statin_sex interaction (S1 Fig).*'  **Page 4:**  '*In addition, a clinical model which adjusted for age, sex, previous MI, physical inactivity, eGFR, BMI, smoking, hypertension and diabetes was calculated. All associations with O2HRmax were further adjusted for beta blocker usage…*  '*Further, as a sensitivity analysis a propensity score analysis was performed. Propensity scores based on sex, age, BMI, diabetes, hypertension, and previous myocardial infarction were calculated using a logistic regression*' |
| Barcelona de Mendoza *et al.* 2016 | **Page 1154:**  '*Potential confounders were identified via a priori knowledge and Directed Acyclic Graphs (DAGs) which utilize graphs to represent relationships between covariates and minimize bias in epidemiologic studies. Based on DAGs, the following covariates were included in multivariable models: age, education and living with a partner. Additional assessment of confounders using 10 % change in estimate methods identified a consistent set of covariates…*  '*As a sensitivity analysis, we repeated the analysis only among nulliparous participants.*'  **Table 3 (footer):**  '*a Each acculturation variable was included in independent multivariable logistic regression models adjusted for age, education and living with a partner*'  **Table 4 (footer):**  '*a Each acculturation variable was included in independent multivariable linear regression models adjusted for age, education and living with a partner*' |
| Barcelona de Mendoza *et al.* 2016A | **Page 80:**  '*Directed Acyclic Graphs and 10 percent change in estimate procedures were used to identify covariates for inclusion in multivariable models; these included age, education, living with a partner, parity, and history of preterm birth.*' |
| Barcelona de Mendoza *et al.* 2016B | **Page 637:**  '*We considered social and demographic risk factors that could confound the relationship between acculturation and anxiety via Directed Acyclic Graphs. These included age, education, parity and living with a partner. We chose not to include cigarette smoking as a covariate because it could be considered an intermediary variable (i.e., on the causal pathway) between acculturation and levels of anxiety.*'  **Table 3 (footer):**  '*Adjusted model included age, education, parity, and living with a partner*'  **Table 4 (footer):**  '*Adjusted model included age, education, parity, and living with a partner*' |
| Bernardi *et al.* 2015 | **Page 295:**  '*A multivariable linear regression analysis was performed to examine the association between CD and metabolic risk factors in early adulthood before and after controlling for covariates. The minimum set of variables to enter the adjusted model was identified in a directed acyclic graph in the DAGitty program (version 2.0 alpha, Johannes Textor). Models were adjusted for maternal schooling at birth, maternal age, maternal smoking during pregnancy, birth weight, sex, and parity (Supplemental Figure 1).*'  **Table 3 (footer):**  '*Model was adjusted for maternal schooling at birth, maternal age at childbirth, maternal smoking during pregnancy, birth weight, sex, and parity, variables identified as a minimum adjustment set in a directed acyclic graph*' |
| Birungi *et al.* 2017 | **Page 515:**  '*This study, two alternative DAGs, depicted in Figures 1-2, were used to illustrate assumed relationships between early lifecourse factors and ECC. Before being included in the DAGs, possible direct paths between all variables considered were evaluated for plausibility based on theory and previous empirical evidence. The wealth asset index was assumed to have direct effects on dental attendance, family sugar consumption, anthropometric status, family oral hygiene, breastfeeding duration and breastfeeding exclusivity. Maternal education was assumed to have direct effects on dental attendance, family sugar consumption, marital status, anthropometric status, family oral hygiene, breastfeeding duration and EBF. Marital status was assumed to have direct effects on the wealth assets index, dental attendance, family oral hygiene, breastfeeding duration and EBF. Breastfeeding duration was assumed to have direct effects on anthropometric status, family sugar consumption, enamel hypoplasia, and cariogenic bacteria. Family oral hygiene was assumed to have direct effects on cariogenic bacteria and ECC, whereas family sugar consumption was assumed to have direct effects on family oral hygiene, EBF, anthropometric status and cariogenic bacteria. Anthropometric status was assumed to have direct effects on breastfeeding exclusivity and enamel hypoplasia. Enamel hypoplasia was assumed to affect cariogenic bacteria and ECC. Cariogenic bacteria were assumed to have direct effects on ECC. Early childhood caries was assumed to have a direct effect on dental attendance. A direct effect of EBF on ECC was hypothesized; hence causal paths between EBF and ECC, mediated through cariogenic bacteria and enamel hypoplasia, were included in the DAGs. In summary, proximal individual- and family-level covariates; anthropometric status (individual level), breastfeeding duration (individual), sugar consumption (family level) and oral hygiene (family level) were assumed to have direct effects on ECC, whereas the effect of more distal family-level covariates; socioeconomic status, maternal education, and marital status were assumed to be mediated through the proximal variables. Dental attendance was assumed to be a collider variable in some paths (model Figure 1) and a mediator in other paths (model 2, Figure 2). Unmeasured variables in terms of enamel hypoplasia and cariogenic bacteria were included in the DAGs for the purpose of providing a clearer picture of the assumed causal mechanisms.*' |
| Bjertness *et al.* 2016 | **Page 4:**  '*We identified confounders for each of the association-outcome-relationships drawing Directed Acyclic Graphs (DAGs). Based on the DAG, for the associations between sociodemographic variables and hypertension all variables in Table 3 were included in analyses as confounders (i.e. age, urban/rural, region, years at school, ethnicity). For the associations between metabolic determinants (Body mass index and Waist circumference) and hypertension (Table 4), we adjusted for sociodemographic confounders (age, urban/rural, region, and years at school) and behavioral confounders (smoking status, smokeless tobacco status, daily fruit and veg. use, oil use, current alcohol drinkers, and vigorous activity at work). For the associations between behavioral determinants (smoking status, smokeless tobacco status, daily fruit and veg. use, oil use, current alcohol drinkers, and vigorous activity at work) and hypertension we adjusted for socio demographic confounders (age, urban/rural, region, years at school).*' |
| Bliddal *et al.* 2016 | **Page 1159:**  '*Potential confounders were chosen according to a directed acyclic graph (19) based on a literature review (results are available from the corresponding author upon request).*'  **Table 2 (footer):**  '*Adjusted for smoking, exercise, and socio-occupational status in pregnancy, and for the time-dependent variables years since start of follow-up, pregnancy, and parity.*'  **Table 3 (footer):**  '*Adjusted for smoking, exercise, and socio-occupational status in pregnancy, and for the time-dependent variables years since start of follow-up, age, and pregnancy. The follow-up period was from conception to 14 years postpartum. † Also adjusted for pre-pregnancy body mass index (BMI). ‡ Adjusted for parity.*  **Table 4 (footer):**  '*aAdjusted for smoking, exercise, and socio-occupational status in pregnancy, and for the time-dependent variables years since start of follow-up, age, and pregnancy. The follow-up period was from childbirth to 14 years postpartum for pre-pregnancy body mass index (BMI) and from 6 months postpartum to 14 years postpartum for pregnancy-related weight changes. † Adjusted for smoking, exercise, socio-occupational status in pregnancy, and pre-pregnancy BMI and for the time-dependent variables years since start of follow-up, age, and pregnancy. ‡ Adjusted for smoking, exercise, socio-occupational status in pregnancy, and gestational weight gain (GWG) and for the time-dependent variables years since start of follow-up, age, and pregnancy. § Adjusted for smoking, exercise, socio-occupational status in pregnancy, and postpartum weight retention (PPWR) and for the time-dependent variables years since start of follow-up, age, and pregnancy.*' |
| Blighe *et al.* 2017 | **Page 1096:**  '*We built**a directed acyclic graph (DAG) with DAGitty 2.3 software (http://dagitty.net/) to identify the appropriate confounding variables to adjust for (Supplemental Table 1, Supplemental Figure 4).*'  **Figure 2 (footer)**:  '*In addition, through a multinomial logistic regression model with sample-to-cluster assignment as outcome and adjusting for significant confounders, sample storage time, maternal age, and education, and the child’s asthma status and vitamin D level at age 3 y, the postsupplement concentration of vitamin D was a significant predictor of cluster grouping*' |
| Blom *et al.* 2015 | **Page** **3:**  '*Causal models were developed by all authors using the free online tool* '*DAGitty*' *(Additional files 1 and 2). The minimally sufficient adjustment set for addressing all three outcomes consisted of time of year, time of week, and shift (time of day). The adjustment set was entered into the logistic equation using the entry method instead of a stepwise method. Interaction terms included were based on empirical knowledge and comprised occupancyashift. Independent variables of the minimally sufficient adjustment set not significantly associated with the outcome were retained to prevent bias. Interaction terms of weaker association with the outcome than p = .05 upon inclusion in the model were omitted…*  '*To improve the face validity of the multivariable models, sensitivity analysis was performed by expanding the minimally sufficient adjustment set (step 1) in two subsequent steps (steps 2, 3). In step 2, triage category, age, and sex were added to the list of covariates. In step 3, three variables—the first indicating whether the patient entered the ED via primary triage, the second indicating whether ED input was high during the simultaneous shift, and the third indicating year 2013, which captures the introduction of RETTS©, increased responsibility for surgical patients at the EM facility, and the introduction of an observation unit— were added to the list of covariates. The selection of variables for expansion was based on knowledge of risk factors for admission in the present dataset and the possibility of a wider spectrum of underlying disease in females suffering from abdominal pain.*' |
| Blomberg *et al.* 2013 | **Page 1014:**  '*By drawing a directed acyclic graph based on potential confounding variables, the minimum set of variables needed to adjust for was identified. This minimal sufficient set of adjustment variables for estimating the total effect of PHTLS on mortality consisted of: calendar year, to account for a possible period effect (such as changes in trauma care over time during the study period); region, to account for possible differences in trauma care between regions; and receiving hospital for outcomes other than prehospital mortality, to account for possible differences in trauma care between hospitals. We assumed other covariates would affect only exposure or outcomes. We also derived more complex multivariable models with variables generally considered to be potential confounders in a trauma study. These models included the variables age, sex, injury severity, role of the injured victim, and comorbidity.*' |
| Bodén *et al.* 2015 | **Page 729:**  '*We used directed acyclic graphs and the program DAGitty (www.dagitty.net) to identify bias-minimized models. The minimal set of covariates for investigating the direct effect of the exposure was considered to be age, gender, smoking, diabetes, previous cardiovascular disease (defined as hypertension, congestive heart failure, stroke or peripheral vascular disease), type and severity of myocardial infarction and treatment (Fig. S1). We performed the analysis in four steps to investigate the effect of each set of covariates using a complete case analysis: (i) model 1, adjusting for age (continuous) and gender (n = 209 592); (ii) model 2, additionally adjusting for cardiovascular disease risk factors [smoking: never smoker (reference), ex-smoker, current smoker; diabetes; previous hypertension; heart failure; stroke; and peripheral vascular disease (all dichotomous)] (n = 199 423); (iii) additionally adjusting for myocardial infarction type and severity [infarction type: STEMI/LBBB versus NSTEMI; Killip classification: no rales (reference), rales in ≤50% of lung fields, rales in >50% of lung fields/pulmonary oedema, cardiogenic shock; and biomarker levels, in quartiles with first quartile as reference] (n = 179 405); and (iv) additionally adjusting for treatment [acute treatments and secondary preventive drugs (all dichotomous)]*' |
| Bowatte *et al.* 2017 | **Page 125:**  '*Covariates were selected for the models after considering alternative causal models using a directed acyclic graph in DAGitty software. Socioeconomic status (defined using education), smoking status, gas cooking, gas heating, keeping windows open more than 1 hour per week, and rural or urban status (using Accessibility/Remoteness Index of Australia 2006) were included in the regression models…*  '*Initially, associations were examined between the exposure variables and allergic sensitization, asthma, wheeze, and lung function. To investigate the potential effect modification role by genetic polymorphisms, categorical variables of GST genotypes were then added as an interaction term to the regression models*' |
| Bowatte *et al.* 2017A | **Page 4:**  '*A directed acyclic graph produced using DAGitty software was constructed to identify possible confounders. This showed what adjustment was required in the regression models in order for the estimated measures of association to have a causal interpretation (under the assumption that the resulting model was correctly specified). Socioeconomic status (defined using educational attainment), smoking status, type of cooking (gas and electric), type of heating (wood/coal, gas room heating and other (electric, ducted heating and reverse cycle air conditioning)) and rural or urban location (using the accessibility/remoteness index of Australia 2006) were included in the models. The same confounders that were included in the main models were adjusted for in the interaction models. In a sensitivity analysis we included sampling weights and all the other cofounding variables described previously in the model to test whether the findings were influenced by the sampling.*' |
| Boyle *et al.* 2015 | **Page 874:**  '*Directed acyclic graph (causal diagram) was used to inform the choice of other covariates. The following known or possible NHL risk factors were included on the causal diagram: alcohol consumption, autoimmune disorders, educational attainment (as a proxy for socioeconomic status), energy intake, ethnicity, family history of NHL, immune function, obesity, smoking, sun exposure, and vitamin D intake (see Supplementary Fig. S1). Associations between these variables, and the direction of the associations, were based on the published literature. Immune function, sun exposure, and obesity were assumed to be on the causal pathway. Ethnicity, educational attainment, and autoimmune disease were identified as a minimal sufficient set of confounders, and a model containing these three variables and the frequency-matching variables was considered the* '*fully-adjusted*' *model. To increase precision, we then used the backward deletion strategy to remove confounders until the change-in-estimate from the* '*fully adjusted*' *model was greater than 5%. Ethnicity (along with the frequency-matching variables) was the only covariate that remained in the model following this procedure.*' |
| Boyle *et al.* 2016 | **Page 737:**  '*Age and study site (WA, BC or Ontario) were included as covariates in all analyses. A causal diagram (directed acyclic graph) was used to select a sufficient set of confounders (ie, a set of confounders that ‘blocked’ all backdoor (confounding) pathways, based on the assumptions made in the directed acyclic graph). The following known or possible breast cancer risk factors were considered as potential confounders and were included in the causal diagram: ethnicity (based on self-reported ethnicity in the CBCS and country of birth in the BCEES), education, family history of breast cancer, age at menarche, number of births, age at first birth, breast feeding, use of oral contraceptives, use of hormone therapy, smoking status, alcohol consumption, recreational physical activity in early adulthood, recent recreational physical activity, body mass index in early adulthood, recent body mass index, total duration of employment, ever worked shift work and years worked in an active occupation (see online supplementary figure S2). Based on the assumptions made in the directed acyclic graph, the following variables were considered to comprise an optimal sufficient set of confounders: education, ethnicity, recreational physical activity in early adulthood, body mass index in early adulthood, number of births, breastfeeding status, shift work status and years worked in an active occupation. In sensitivity analyses, smoking status and alcohol consumption were added to the final pooled models, and their inclusion did not change the observed risk estimates.*' |
| Buchner & Rehfuess 2015 | **Page 3:**  '*A published causal diagram of child ALRI determinants distinguishes vulnerability (i.e. nutritional status such as stunting, breastfeeding, birthweight, vaccination, HIV status), exposure to risks (i.e. household air pollution, environmental tobacco smoke, outdoor air pollution, housing, crowding, handwashing), access to health care (i.e. care-seeking, transportation, affordability), household socio-economic status (i.e. maternal and paternal education, maternal and paternal occupation, wealth, income) and contextual factors (i.e. ethnicity, urban/rural location, geographical location). We attempted to populate this diagram with relevant DHS variables at individual/household level (Table 2). In addition, we included child age, sex and birth order as non-modifiable risk factors, and developed the country-level variable rainy season as a further contextual factor to indicate whether the interview took place during the rainy or dry season, with the information obtained from the CIA factbook and other relevant sources (Table 1). All coding of variables was done a priori with regard to existing evidence and likely causal mechanisms.*'  **Page 9:**  '*For model selection, we used a stepwise backward selection algorithm based on the Akaike Information Criterion (AIC), selecting the model with the smallest AIC value as the best-fitting model.*' |
| Buckley *et al.* 2016 | **Page 509:**  '*We adjusted for potential confounding variables identified using directed acyclic graphs (see Supplemental Material, Figure S1). Demographic and socioeconomic characteristics included maternal race/ ethnicity (non-Hispanic white/non-Hispanic black/Hispanic), age, education (less than college/college degree or more), and work status during pregnancy (employed/student or homemaker). We adjusted for maternal body size characteristics (prepregnancy BMI and adequacy of gestational weight gain), maternal smoking during pregnancy (yes/no), and breastfeeding (ever/never) to account for earlylife factors that are associated with childhood overweight status (Weng et al. 2012). We adjusted for calendar date of urine collection to account for temporal trends in phthalate exposure and prevalence of childhood obesity. Additionally, we adjusted for natural log creatinine (to account for urine dilution), child’s sex, months of age at follow-up, and a product term between child’s sex and months of age at follow-up. We also included strong predictors of the outcome, including maternal height and child physical activity at follow-up (active/ inactive), to improve precision.*' |
| Busert *et al.* 2016 | **Page 3:**  '*Potential confounding factors in the association between dietary diversity and conditional growth were identified with the use of a directed acyclic graph (DAG) (Supplemental Figure 1) and the software DAGitty. DAGs serve to represent visually assumed causal relations between exposures, outcomes, and covariates and are used to identify potential sources of bias in observational health research. We determined a minimally sufficient adjustment set that contained the following factors: household wealth, household food insecurity, crowding, maternal height, maternal education, child care during illness, continued breastfeeding, child infections, general care, child age, and sex. Wherever possible, we identified actual variables or proxies from our data set…*  '*We estimated the crude and mutually adjusted associations between conditional growth and covariates with the use of OLS regressions.*' |
| Byberg *et al.* 2016 | **Page 3:**  '*Potential confounders included categorical and continuous variables, i.e. sex; gestational age; birth order (firstborn or not); duration of breastfeeding (categories: none; <3; >3 months. This information was extracted from maternal questionnaire, and used in analyses for predictors at target ages ≥6 months); mother’s preeclampsia (none, mild/moderate, severe); mother’s BMI (weight at first antenatal visit and height at first followup); mother’s smoking (at first antenatal visit); mother’s doctor-diagnosed asthma; mother’s education (from maternal questionnaire: <9; 9–12; >12 years) and mother’s age. This is illustrated in a Directed Acyclic Graph (Additional file 1: Figure S1) …*  '*Each variable was entered separately into simple regression models. Next, all potential confounders were included in fully adjusted models. Backward stepwise selections were performed to remove non-significant confounders, unless there was ≥15 % change in effect size upon removal of the confounder. Last, final models for each target age included anthropometrics, physical activity, sex of the child and all remaining confounders.*' |
| Camelo *et al.* 2015 | **Page 93:**  '*We constructed a directed acyclic graph (DAG) of proposed associations between life course socioeconomic position and subclinical atherosclerosis (IMT) in adulthood to guide our analyses (Fig. 1). Following this DAG, to investigate the mediating role of job strain on the association between life course SEP and IMT, we could not condition on health-related behaviors or markers of metabolic, endocrine and immune dysregulation because all of these variables are colliders…*  '*Linear regression models were performed with IMT as the outcome of interest. First, we assessed the association of age and race with IMT. Second, we evaluated the association of job stress and each life course SEP indicator with IMT adjusted by age and race.We also examined the association between SEP indicators and IMT, including all SEP indicators simultaneously in the model. Then, we separately added into this model the job characteristics that were statistically associated with IMT and were also associated with SEP (according to the chi-square tests) to evaluate the mediating role of job stress. In addition, we evaluated the association between cumulative SEP score and IMT adjusted by age and race, and then we also separately added the job characteristics that were statistically associated with IMT and SEP indicators to evaluate the mediating role of job stress in this association.*' |
| Camlin *et al.* 2016 | **Page 497:**  '*We used directed acyclic graphs encoding our hypothesized causal relationships to guide multivariable analysis and adjustment decisions. Selection of the minimum sufficient adjustment sets to identify the effect of each dimension of factors on reengagement was aided through use of DAGitty software*'  **Table 4 (adjustment sets displayed for each exposure)**:  '*Each model required different sets of adjustment variables for estimating the total effect of the factor on reengagement.*' |
| Carlson *et al.* 2005 | **Page 256:**  '*Multivariate analyses were guided by the a priori causal model (Fig. 1), which included known and hypothesized associations between the available sociodemographic and exposure variables of primary interest for the analyses. Odds ratios (ORs) and 95% confidence intervals (CI) were calculated for risk of injury using logistic regression. Selection of confounders for multiple logistic regression, using the principles in Maldonado and Greenland [2002], was based on directed acyclic graphs (DAGs), derived from the causal model. The use of DAGs is described by Greenland et al. [1999] and illustrated by Hernan et al. [2002]. These methods identify minimal confounder sets, leading to parsimonious models, and help identify covariates that may introduce bias if entered into the regression.*'  **Table 2 (footer):**  '*aAdjusted for within-household correlation using GEEs [Liang and Zeger, 1986], excluding level for missing values, and weighted for non-response [Horvitz and Thompson, 1952; Mongin, 2001]. bAdjusted for age and state. cAdjusted for gender and state.*  *dAdjusted for age and gender. eAdjusted for age, gender, and state. fAdjusted for age, gender, state, and education. gAdjusted for age, gender, education, marital status, and prior injury status. hAdjusted for age, gender, state, education, and marital status.* |
| Carlson *et al.* 2006 | **Page 1138:**  '*To determine potential confounders, we used a causal model to guide the design and use of directed acyclic graphs, according to the methods in Greenland et al. and Hernan et al. These methods facilitated identification of covariates that may introduce bias if included in the statistical models and reduced potential of overfitting the models. For parent-related covariates, data specific to fathers or mothers were used for the respective analyses of fathers’ and mothers’ injuries. For analyses of combined parental injury history, mothers’ data were included, assuming these factors have a greater effect on children’s risk.*'  **Table 2 (footer):**  '*†Models included state, enterprise, number of children in the household, father’s age and education level, and total household hours worked on the operation. ‡Models included state, enterprise, number of children in the household, mother’s age and education level, and total household hours worked on the operation.*'  **Table 3 (footer):**  '*aModels included state, type of agricultural operation, number of children in the household, mother’s age and education level, and total household hours worked on the operation. †Adjusted for within-household correlation by using generalized estimating equations and weighted for nonresponse*' |
| Casas *et al.* 2015 | **Page 673:**  '*Directed acyclic graphs (DAGs) were used to graphically represent the direction of the relationships between covariates and to select those included in the adjusted models (see Supplemental material, Fig. S1). Covariates included in the DAG were selected based on previous evidence. Adjusted models included age and sex of the child, maternal age (<25, 25–29, 30–34, 35+ years), maternal education (primary, secondary, and university), maternal smoking during pregnancy (yes, no), maternal IQ (continuous score), and maternal consumption of canned fish (continuous in servings/week). Potential confounding by other covariates reported to be associated with neurodevelopment and/or BPA in the literature (any breastfeeding and maternal pre-pregnancy body mass index) was then evaluated in the adjusted model using forward selection procedures. These covariates were retained in the final models only if they modified the coefficient of the adjusted model by more than 10%.*' |
| Chandee *et al.* 2017 | **Page 1167:**  '*All covariates were considered as potential confounders using a directed acyclic graph (DAG) approach for each model.*'  **Page 1168:**  '*Confounders for each model were chosen a priori based on prior knowledge and existing relationships identified in the literature using a DAG (Supplementary Figs. 1–6, Supplemental Digital Content 1, http://links.lww. com/CCM/C864) approach to identify the minimum set of confounders necessary for complete adjustment*'  **Table 3 (footer):**  '*a Adjusted using the following minimum sets identified using a directed acyclic graph for each variable with imputation: Hypotension: Injury Severity Score (ISS), polytrauma, mechanism of injury; Glasgow Coma Scale (GCS): ISS, mechanism of injury, systemic hypotension; ISS: male, mechanism of injury, polytrauma; polytrauma: male, mechanism of injury.*' |
| Chattopadhyay *et al.* 2003 | **Page 159:**  '*For the present analysis, we concentrated on the main effects model, assuming that the dental insurance-dental visit relationship was uniform across the different levels of the covariates adopting a backward elimination strategy using the difference in -2 log likelihood between models as the model selecting criteria. The DAG suggested that some of the factors like income and employment might act through dental insurance. Therefore, by adjusting for dental insurance (main exposure variable), it can be argued that the true effects of these factors were diluted. We therefore assessed another model that contained all the variables, but excluded dental insurance*' |
| Cupul-Uicab *et al.* 2013 | **Page 1104:**  '*We used directed acyclic graphs (DAGs) to choose potential confounders for model adjustment. The set of variables selected for adjustment were maternal race, education, socioeconomic index, prepregnancy BMI, smoking during pregnancy, and child’s birth order. This list of variables plus study center (12 strata), child’s sex, serum levels of triglycerides and total cholesterol were the covariates selected a priori for model adjustment. Another set of potential confounders was also assessed using the change in estimate method (i.e., change in OR ≥ 10%), starting with all variables in the models with deletion of one by one in a stepwise manner. None of the additionally tested variables (i.e., maternal age, pregnancy weight gain, weeks of gestation at enrollment, and child’s age) were selected with this strategy.*' |
| Cupul-Uicab *et al.* 2014 | **Page 20:**  '*The selection of potential confounders for inclusion in the model was based on directed acyclic graphs (DAGs). Maternal age, parity, poverty status, parental smoking, and residence area were selected as the minimal sufficient set for adjustment. Other variables not selected by the DAG (i.e., birth season, child's underweight, breastfeeding, exclusive breast feeding, and maternal education) were assessed as potential confounders with the change in estimate method (i.e., change in IRR >10%), starting with all variables in the models and deletion of one by one in a stepwise manner. None of the tested variables cause a change >10% in the IRR*' |
| Curran *et al.* 2016 | **Page 535:**  '*Potential confounders were identified using previous literature and examined for inclusion using a directed acyclic graph (DAG) (Supplementary Figure 1, available as Supplementary data at IJE online)…*  '*In fully adjusted models, we also included infant gender, maternal age, gestational age, maternal and paternal citizenship, SGA, LGA, 5-min Apgar score, parity, maternal smoking during pregnancy, social welfare status, parental education, family disposable income and maternal and paternal depression, bipolar disorder and non-affective disorder.*' |
| Curran *et al.* 2017 | **Page 2:**  '*Based on previous literature and the use of a directed acyclic graph (DAG) (Additional file 1: Figure S1), the following a priori co-variates were included in the analysis: maternal age at time of birth (<25 years, 25–34 years, 35–44 years, 45+ years), birth order (first born), small for gestational age (SGA), large for gestational age (LGA) (defined as birth weight less or greater than 2 standard deviations from the mean for gestational age, respectively), gestational age (<37 weeks, 37, 38, 39, or 40 weeks, >40 weeks), maternal country of birth (Swedish, other Nordic, other), maternal depression, non-affective disorder, or bipolar disorder (never diagnosed, diagnosed before birth, diagnosed after birth), parental income at time of birth (in quintiles), and parental social welfare at time of birth (yes/no, note: available from 1983), and parental highest education (prehigh school, high school, post-high school). Though not identified as confounders in the DAG, further co-variates that were identified based on previous literature were also assessed, including: year of birth, year of school completion, smoking at time of first antenatal visit (none, 1–9 cigarettes/day, 10+ cigarettes/ day), infant gender, Apgar score at 5 min (*'*low*' *[0–3],* '*intermediate*' *[4–6],* '*high*' *[7–10]), paternal country of birth (Swedish, other Nordic, other), paternal depression, non-affective disorder, and bipolar disorder (never diagnosed, diagnosed before birth, diagnosed after birth), parental co-habitation at time of birth. All co-variates were tested individually in the logistic regression analysis to assess the potential impact on the association between mode of delivery and school performance. As no variable changed the estimate by more than 10% (only maternal age changed the estimate by more than 5%), only the variables decided on a priori were included in final analysis.*' |
| Dahl *et al.* 2012 | **Page 3:**  '*We used DAGitty, a web-based application based on the directed acyclic graph theory, to model the causal relationship between fibulin-1 and cardiovascular mortality and any confounders. This DAG program was used to identify sets of confounders that together fully adjust for confounding in multivariable modeling. Several of these sets of confounders were identified (Figure S1). The set containing age, history of diabetes, AS severity, and LVMi was used for multivariable analysis*' |
| Dalgaard *et al.* 2016 | **Page 649:**  '*We constructed causal diagrams to identify minimal sufficient adjustment sets for each variable, using the web-based software* '*DAGitty*'*. These variables were included in univariate and multivariate Cox regression models.*'  **Table 3 (adjustment sets displayed for each exposure):**  '*Minimal adjustment sets for estimating the total effect of each exposure were defined according to causal diagrams.*' |
| de Jonge *et al.* 2014 | **Page 3:**  '*We identified potential confounders based on a review of the literature. A Directed Acyclic Graph (DAG) was then used to assist in the selection of appropriate confounders by modelling the relationships between potential confounders, short birth interval and stillbirth or neonatal death [see Additional file 1]. The DAG was created using a pre-determined selection criteria in order to minimize biases that have recently been shown to be present when using traditional methods of confounder selection [20]. The final confounders used to model the effects of birth interval on birth outcome were maternal age, parity, education, religion, household assets, tea garden residence and previous adverse pregnancy outcome. A mixed effects logistic regression model was used to evaluate the association between birth interval categories and adverse outcomes of pregnancy adjusting for confounders and the clustered design of the study. The DAG was drawn in Dagitty, an online tool*' |
| di Giuseppe *et al.* 2015 | **Page 134:**  '*Directed acyclic graphs (DAG) was implemented to understand the underlying causal structure among the variables and to choose a minimal sufficient adjustment set of covariates.*'  **Page 135:**  '*The minimal sufficient adjustment set between FGF23 and CVD included the following covariates: age, gender, smoking habits, prevalent hypertension, hyperlipidemia and diabetes, dietary phosphorus an calcium intake, PTH, 25-Hydroxyvitamin D3, and eGFR (Fig. 2). A competing DAG was drawn which additionally included BMI as covariate (not shown).*' |
| Dolatowski *et al.* 2016 | **Page 254:**  '*Based on the previous literature, we conducted CRR analyses with the following independent variables: age, sex, ASA classification, cognitive impairment (as noted in the patient records), time to surgery (from hospital admission to start of surgery), and postoperative posterior tilt…*  '*A minimal adjustment set of covariates was selected using directed acyclic graphs (DAGs) by closing all biasing paths, leaving all causal paths open (Shrier and Platt 2008). The DAG diagram was drawn with DAGitty version 2.3 (http://www.dagitty.net).*' |
| Downes *et al.* 2015 | **Page 669.e2:**  '*Potential covariates for the adjusted models were explored based on previous literature, use of a directed acyclic graph, and evaluation of individual regression models; a probability value of < .10 was considered significant.*' |
| Dusingize *et al.* 2017 | **Page 1707:**  '*Using the DAGitty program, we constructed directed acyclic graphs to identify a minimum sufficient adjustment set of confounding factors to estimate the total effect of smoking on BCC or SCC. Our final models were adjusted for age, sex, private health insurance, education status, natural skin color, tanning ability, number of freckles, history of sunburn as a child, and cumulative sun exposure.*' |
| Dzhambov & Dimitrova 2015 | **Page 575:**  '*In line with previous research we used directed acyclic graphs (DAGs) and the graphical tool DAGitty v.2.2 to determine a priori the sufficient adjustment sets for estimating total and direct effects of RTN on BMI. Based on prior theory, we entered age, gender, distance to major road and noise sensitivity into the models. DAGs suggested that the total effect models needed to be adjusted for socio-economic status and ethnicity, and the direct effect models — for noise annoyance, sport, diet, sleep disturbance and smoking.*' |
| Dzhambov & Dimitrova 2016 | **Page 437:**  '*Causal diagrams were set up to determine the necessary adjustments in the multivariate models using DAGitty v. 2.3. We were interested in the total effect of self-reported occupational noise and vibration since the direct effect, albeit of theoretical importance, is not observed per se in real life. In order to investigate the complex imbrication of various environmental and individual risks for the CVD, even factors that we had no data on (i.e., diet, physical activity, heredity, body mass index, residential exposures) were included in the diagrams. Their absence, however, was found to be acceptable, since they were necessary covariates for the direct rather than the total effect model. The suggested minimal sufficient set of covariates for estimating the total effect of self-reported occupational noise/vibration on heart disease included: age, gender, workweek hours, length of service, shift work, and other co-exposures. We additionally forced into the models (as proxies for participants’ socioeconomic status) the number of household members and the age at which full-time education was finished*' |
| Eichler *et al.* 2016 | **Page 3**:  '*Potentially confounding variables were integrated using Directed Acyclic Graphs. Regarding tobacco consumption, SES, age, sex, and household size were considered as potentially confounding variables; regarding alcohol consumption, SES, age, sex, TNM classification, cancer site, and household size were considered as potential confounders. According to the Directed Acyclic Graphs model, comorbidities were a potential confounding variable for alcohol consumption as well, but unfortunately, this information was not collected routinely in our study, so there was no possibility to adjust for it.*' |
| Ellison & De Wet 2016 | **Page 134**:  '*A causal path model was then developed (in the form of a Directed Acyclic Graph [DAG] drawn using www.dagitty.net open source software) to distinguish between confounders and mediators in any relationships between each of the sociodemographic and socioeconomic variables and health, assuming that only variables relating to pre-existing characteristics and past events could act as potential confounders (see Figure 1). This model assumed that age, sex and population group classification were all determined simultaneously (at birth) and that these all influenced educational attainment, migration status, employment status, type of dwelling, household crowding, household services and assets, adult food insecurity and, thereafter, health in a sequential fashion. This model then prescribed the sequence with which potential confounders were entered into multivariable logistic analyses examining the relationship between each sociodemographic and socioeconomic variable and health, in order to adjust for potential confounding. This involved including sex, age and population classification in all analyses examining the relationship between these three sociodemographic variables and health; while each of the variables relating to household services and assets were adjusted only for preceding sociodemographic and socioeconomic characteristics (including type of dwelling and crowding) and not for other household services or assets, on the grounds that these were likely to be highly collinear. Finally, to test the possibility that self-reported disability preceded educational attainment and thereafter migration status (rather than being a contemporaneous measure of recent/current health status), the multivariable logistic regression analyses were repeated to assess the impact of adjusting for disability (i.e. assuming this to be a potential confounder) in the relationships between individual- and household-level socioeconomic status and health (see Figure 1).*' |
| Emeny *et al.* 2014 | **Page 60:**  '*Confounder selection was performed using causal diagrams with variables entered into the model based upon reported covariates of well-being (Sodergren et al., 2014) and growth hormone. We identified minimally sufficient adjustment sets using diagrams that represent the relations among the exposure, outcome, and other variables. All previously mentioned covariates were considered potential adjustment variables for the association between well-being and growth hormone. The minimally sufficient adjustment set for the direct association of IGF-I and IGFBP-3 with well-being included age, sex, physical activity, sleep patterns, BMI, smoking and cognition (see Supplemental Fig. 1).*' |
| Erkal *et al.* 2009 | **Page 422:**  '*Separate, theoretical causal models, were developed for children/youth and adults, based on previous research and expert knowledge. This enabled modeling of dependence of horse-related injury on each exposure of interest, while controlling for potential confounders. Selection of confounders for each exposure of interest/respective model was based on directed acyclic graphs (DAGs) established from these models (Supplementary Figures 1 and 2, available online). Each model indicated potential confounders controlled for, following reputable methods. This enabled identification of parsimonious models, excluding covariates that should not be included lest they introduce bias.*'  **T*a*ble 3 (footer):**  '*∗Adjusted for age and gender. §Adjusted for age and state. ‡Adjusted for gender and state (and head of household age for children). ¶Children: Adjusted for child’s age, head of household age, gender, head of household education, prior agricultural injury. Adults: Adjusted for age, gender, marital status, education, prior injury. †Children: Adjusted for age, state, gender, head of household education. Adults: Adjusted for age, gender, state, marital status, education. ∗∗Children: Adjusted for head of household age, state. Adults: Adjusted for age, gender and state. ¥Adjusted for age, gender, state, education*' |
| Escobar *et al.* 2017 | **Page 7**:  '*To do this, we created a* '*causal model*' *using our variables (like a flow diagram) leading to acute kidney injury using DAGitty software.*'*;*  **Page 10**:  '*DAGitty analysis of the diagram determined that to estimate the total effect of AJ on AKI required statistical analysis of the following groups of variables: age and arterial indication, age and chronic renal failure (CRF) and diabetes (DM), arterial indication versus venous indication, CRF and DM and venous indication. To determine the direct effect of AJ and the risk of developing AKI, statistical models should also include age, indication for procedure, hematocrit drop and surgery; as each of these could independently cause AKI. Individual statistical modeling for each of these groups of variables was not statistically significant (data available upon request) and our findings were unchanged after DAG analysis.*' |
| Evandt *et al.* 2017 | **Page 2**:  '*In the procedure of selecting covariates to the statistical models, we used a directed acyclic graph (DAG). The DAG was developed using the Web-based software DAGitty by including factors relevant for the association between traffic noise exposure and sleep disturbances, based on literature review of previous research. In Figure 1, we have presented a simplified version of the DAG. The variables identified by the DAG were age, sex, marital status, alcohol use, smoking, physical activity, and night-shift work, in which age and sex were specified as compulsory variables for the final adjustment set. Further, it has been found that socially deprived individuals are exposed to higher noise levels than more socially advantaged individuals. Thus, we added the socioeconomic factors educational level and household income to the final adjustment set.*' |
| Evandt *et al.* 2017A | **Page 3:**  '*In the procedure of selecting covariates to the statistical models, we used a directed acyclic graph (DAG). The DAG was developed using the web-based software DAGitty. In order to decide which variables to be included in the DAG, we reviewed previous relevant re- search. Since we considered sleep medication use as an indicator for sleep disturbances, we included variables relevant for the association between traffic noise exposure and sleep disturbances. In Additional file 1: Figure S1, we have presented a simplified version of the DAG. The full version of the DAG can be accessed at www.dagitty.net/ mAeotvC. The minimal sufficient adjustment set we used for the statistical analyses included the variables age, sex, population density, marital status, alcohol use, smoking status, physical activity, night shift work, and rail traffic noise. Age and sex were specified as compulsory variables for the final adjustment set. Further, it has been found that socially deprived individuals are exposed to higher noise levels than more socially advantaged individuals. Thus, we added the socioeconomic factors educational level and household income to the final adjustment set.*' |
| Fan *et al.* 2016 | **Page 3:**  '*Potential confounders were adjusted a priori using direct acyclic graph (DAG) and biological plausibility. DAG was used to identify suitable minimally sufficient adjustment sets. Using the 6-step DAG approach for confounder selection is likely to reduce the degree of bias for the effect estimate in the chosen statistical model, compared with the traditional epidemiological methods.*' |
| Feda *et al.* 2010 | **Page 465:**  '*Directed acyclic graphs (DAGs) served as a visual guide to identify variables pertinent to the multivariate models and potential confounders on the outcome of interest. DAGs allowed us to include variables believed to be causal to the exposure of interest and exclude variables that could not be causal. The DAG, Figure 1, shows relations among the exposures of interest, the outcomes, and other covariates. During the analysis, each policy used a separate analytical model, based on the assumptions illustrated in the DAG. The enforcement of policies, for example, although important, falls on the causal pathway between written polices and physical assault and, therefore, was not included in analyses.*'  **Table 3 (footer):**  '*aMultivariate model adjusted for school type; school location; student age; student impairment status: drugs, illness/injury, and disability; administration’s attitude toward violence. bMultivariate model adjusted for above variables and weighted for probabilities of response and eligibility-based data from the Minnesota Department of Education.*' |
| Ferraro *et al.* 2017 | **Page 4:**  '*To avoid overadjustment covariates were selected with the aid of a directed acyclic graph (DAG) (Supporting Information Figure S1), following current practice in life course epidemiology, which highlights the risks of overadjustment by factors that might be intermediate in the pathways under consideration, and are therefore not confounders. Each variable was ordered according to its specific temporal relationship: pre-conception (maternal skin colour; occupation of the head of the family, maternal age and schooling); antenatal (child’s gender and maternal smoking during pregnancy); perinatal (maternal hypertension at delivery, type of delivery; BMI, length and gestational age); school age (type of school; BMI and height); adulthood (adult age, occupation of the head of the family; physical activity, smoking, alcohol consumption, sodium intake; blood pressure, BMI and height). Identifying temporal order facilitates understanding relationship between variables. We then built pathways based on temporality and theoretical assumptions. The resulting model was built upon these associations in a browser-based environment (http://www.dagitty.net). Adult risk factors for HT (physical activity, alcohol and sodium intake) did not fulfil the criteria do be considered confounders, because they are not antecedents of the exposures. This approach to deal with confounding covariates is increasingly being used.*'  **Table 5 (footer):**  '*Model 1 is adjusted for gender, type of delivery, preterm birth, maternal hypertension, adult smoking, adult age and adult occupation of the head of the family. Model 2 includes all anthropometric variables entered together in model I.*'  **Table 6 (footer):**  '*Model 1 BMI and length/height entered into separate models that were adjusted for gender, type of delivery, gestational age, maternal hypertension, adult smoking, adult age and occupation of the head of the family in adulthood. Model 2 BMI and length/height were adjusted for each other and for gender, type of delivery, gestational age, maternal hypertension, adult smoking, adult age and occupation of the head of the family in adulthood.*' |
| Ferraro *et al.* 2017A | **Page 3:**  '*Confounders for which models were adjusted were chosen based on the assumption that they must be antecedent of exposure and outcome. Since the violence measurement only encompassed the last 12 months, the independent variables that fulfilled this assumption were maternal schooling, maternal migration, family socioeconomic status, and being an adolescent mother. Finally, we tested whether the associations could be explained by the presence of maternal risk behaviours, namely smoking, drinking, inadequate prenatal care, and inadequate weight gain. Stressful events could increase the likelihood of these behaviours, which in turn could affect foetal development. If the association disappears when these variables are included in the models it means that these variables completely explain the link between mental disorders/domestic violence and neonate outcomes, since they would be in the pathway between exposure and outcome.*'  **Table 2 (footer):**  '*aSame adjustments plus for maternal risk behaviours: smoking during gestation, drinking during gestation, inadequate number of prenatal care visits for the gestational age and inadequate weight gain for the gestational age considering the pre-gestational BMI aAdjusted for socio-demographic variables: maternal schooling, economic class, adolescent childbearing, maternal migration*'  **Table 3 (footer):**  '*aSame adjustments plus for maternal risk behaviours: smoking during gestation, drinking during gestation, inadequate number of prenatal care visits for the gestational age and inadequate weight gain for the gestational age considering the pre-gestational BMI aAdjusted for socio-demographic variables: maternal schooling, economic class, adolescent childbearing, maternal migration*' |
| Figueiredo *et al.* 2017 | **Page 3:**  '*The confounders were selected based on a directed acyclic graph (DAG) that was constructed with DAGitty; an online free software. The DAG is a graphical model that functions to identify minimal sufficient adjustment to estimate causal effects between exposure and outcome. The DAG for the causal relation- ship between 25(OH)D and depressive symptoms suggested that the following variables should be adjusted: gestational age, age, pre-pregnancy body mass index, self-reported skin color, previous history of depression, education, alcohol intake and smoking habit (Supplemental Fig. 2).*' |
| Filippidis *et al.* 2017 | **Page 1102**:  '*We have drawn a detailed directed acyclic graph with DAGitty to illustrate potential causal pathways from cigarette prices to infant mortality, identify potential sources of confounding, and guide covariate selection (eFigure 2 in the Supplement).*'  **Page 1102**:  '*Our final model included within-year and 1-year lagged terms for the median cigarette price and the price differential, adjusting for potential confounders (gross domestic product per capita, unemployment, TCS smoke-free score, educational level, and maternal age), and time (quadratic term) to account for underlying nonlinear time trends. Mediators of the association between cigarette prices and infant mortality, such as population-level cigarette consumption, smoking in pregnancy, exposure to SHS in pregnancy and infancy, congenital anomalies, and preterm birth were not included in the model, consistent with our directed acyclic graph*' |
| Filteau *et al.* 2016 | **Page 3:**  '*Analyses were guided by the directed acyclic graph in figure 1, which focused on vitamin D status, anthropometry and bone health as well as probable contributors to these: socioeconomic status (SES) at follow-up, diet, recent morbidity, sun exposure and early growth. Analyses followed principles of causal inference and were aided by DAGitty, an online tool.*'  **Page 4**:  '*We used the directed acyclic graph in figure 1 to investigate further the effects on motor function of the three proximal factors of key interest: current vitamin D status, anthropometry and bone health. The minimal set of potential confounders needed to determine the direct effect of vitamin D status included SES, sun exposure, HAZ, tibia Z score and recent morbidity. Control for these had little effect on associations between 25OHD and motor outcomes (comparing table 3 with table 2), except that, following adjustment, higher 25OHD was associated with fewer squats. The minimal set of potential confounders for HAZ and AMA included SES, tibia Z score, recent morbidity and 25OHD; and for tibia Z score, included SES, 25OHD, HAZ and recent morbidity; again adjustment in both cases had little influence on the results.*'  **Figure 1:**  '*Conceptual framework for factors affecting motor development. Variables included under each of the group headings are: (1) motor performance: Ages and Stages Questionnaire pass/fail category, maximum grip strength, minimum run time, maximum number of squats in 15 s; (2) anthropometry: height and body mass index Z scores, arm muscle area; (3) vitamin D status: serum 25-hydroxyvitamin D; (4) bone health: radius and tibia quantitative ultrasound Z scores; (5) sun exposure: h/day; (6) diet: animal food groups; (7) infant growth: birth weight tertile, length-for-age Z score at 6 months, change in length-for-age Z score from birth to 6 months; (8) recent morbidity: reported symptoms in the past 3 days; (9) season of interview: 4-month divisions; (10) sociodemographic factors: quintiles from principle components analysis and (11) vitamin D in infancy: treatment group allocation in Delhi Infant Vitamin D Supplementation (DIVIDS)-1 trial.*' |
| Frederiksen *et al.* 2009 | **Page 1109**:  '*Before analysing the data we set up a series of causal diagrams to summarize visually the hypothetical relationship between the variables of interest for the research question, paying special attention to the direction of each arrow and thereby distinguishing confounders and intermediates. The first diagram (Fig. 1) illustrates the pathways between the different indicators of SES and the outcome and guided which indicators were to be included in the statistical models to obtain total effects. Estimation of total effects requires adjustment for confounders, but must not include mediators. We represent SES by three primary variables of interest, i.e. education, income, and housing status. Since education precedes income and housing status temporally, the effect of education on survival is mediated though these two factors; which explains why models of education will not be adjusted for income or housing status. The association between income and survival is confounded by education, so education will be included in models investigating income. We further hypothesize that housing status is confounded by education and income. Cohabitation status is regarded as a confounder when income and housing status are primary variables of interest and as an intermediate when education is investigated, since current cohabitation status normally follows education temporally. It will thus be included only in models of income and housing status. Furthermore, age, sex and year of operation are confounders of the association between SES and survival (as illustrated in Fig. 2). To investigate any mediating effect of patient, disease, or treatment factors we calculated the direct effect of SES on the outcome, which is the effect of SES on the outcome that is not going through the mediator. This was done by controlling the mediator at a constant level by adjusting for it. We thus obtained an estimate of the controlled direct effect.*'  '*The selection of potential mediators was made on the basis of known prognostic factors potentially unevenly distributed among social groups and the available data in the database. These included lifestyle, comorbidity, stage of disease at diagnosis, mode of admission, type of operation, specialization of the surgeon, and whether the surgery was curative. Fig. 2 shows a simplistic diagram including both confounders and categories of mediators. In the diagram we write only SES, instead of income, education or housing status. Fig. 3 illustrates the hypothetical interrelations between exposure, specified mediators and outcome. As illustrated by the slim arrows mediators may also affect each other. In the analyses mediators were added stepwise in a series of models. Factors placed more ‘upstream’ in the causal pathway from SES to the outcome had to be included before the more ‘downstream’ ones. For instance, the ‘upstream’ factor of lifestyle affects the more ‘downstream’ factor of comorbidity, eg. smoking may cause COPD. Therefore, when investigating any attenuating effect of lifestyle on the association between SES and survival, comorbidity should not be included, since it is an intermediate. However, when investigating the impact of comorbidity on the association between SES and survival, lifestyle should stay in the model to adjust for confounding of the effect of comorbidity on survival. Consequently, this allows us to estimate the controlled direct effect of SES on survival, which is not mediated through lifestyle and/or comorbidity. These considerations are to be transferred to the interrelation of all the mediators and determine their inclusion in the models.*'  **Table 3 (footer):**  '*a Confounders: Income is adjusted for age, sex, year of operation, cohabiting status, and education. Education is adjusted for age, sex, and year of operation. Housing status is adjusted for age, sex, year of operation, cohabiting status, education, and income.*' |
| Gagliardi *et al.* 2009 | **Page 585:**  '*We adjusted for the possible confounders listed in Table 1 and in Fig. 1. This was done in successive steps, to help understand the effect of various adjustments. We followed the DAG in a chronological order (prenatal–perinatal–postnatal variable adjustment) by adjusting first for GA using a dummy variable for each gestational week. We next adjusted for illness severity and other perinatal variables, and then added ventilation, one major antecedent of BPD and other postnatal variables. Ventilation was subdivided into four categories: no ventilation; ventilation <24 h; ventilation >24 h and <7 day; ventilation >7 days. We also calculated a propensity score for ventilation (longer than 24 h), to adjust for confounding by indication, that is, attributing to ventilation what should be attributed to factors or covariates that influence the likelihood of receiving ventilation treatment…To design the propensity score of receiving ventilation, we performed a logistic regression using as independent variables pre- and perinatal factors (GA, sex, being SGA, twin gestation, location of birth, mode of delivery, Apgar score at 1 min, antenatal steroids and intubation in delivery room). For each infant, the predicted probability of receiving the treatment was calculated. This probability (propensity score) was used as a covariate in subsequent logistic models that had WMD as the outcome variable, and BPD, ventilation and other factors, as independent variables…*  '*If, however, there exists an unobserved factor that causes low GA, and – through other pathways – both BPD and PVL, there will be residual confounding. This scenario could be represented by a cause of preterm birth (e.g. intrauterine infection/chorioamnionitis) that can cause preterm delivery, and both BPD and PVL by pathways that are different from those controlled for (see Fig. 2). Under such model (DAG 2), early-onset sepsis, that is not directly linked to either WMD or BPD, could act as a proxy for intrauterine infection (‘U’ in the graph), so that conditioning on it could help reduce confounding.*' |
| Gascon *et al.* 2015 | **Page 372**:  '*To determine the covariates to be included in multivariate models for each exposure variable, we applied directed acyclic graphs (DAGs) using DAGity software. Covariates included in the respective DAGs if they were described to be associated with the exposure or the outcome in previous literature, and such associations were shown in bivariate analyses of our data (P ≤ .1, see Table E2 in this article’s Online Repository at www.jacionline. org). According to the DAGs, final multivariate models for BPA included maternal education, number of siblings, and maternal smoking during pregnancy, and multivariate models for phthalate metabolites additionally included maternal prepregnancy body mass index and maternal history of asthma, allergy, or both (see Figs E1 and E2 in this article’s Online Repository at www.jacionline.org).*' |
| Gaspar *et al.* 2017 | **Page 077006-2:**  '*A directed acyclic graph (DAG) was generated to conceptualize the estimation of serum levels and interventions and to identify potential confounders (Figure S1)*'  **Page 077006-2:**  **'***To estimate [E(Y|A,W) and E(A,W), we used the Super Learner algorithm, an ensemble machine learning algorithm that uses a weighted combination of algorithms to return a prediction function that minimizes cross-validated mean squared error… we used the Super Learner algorithm with the following candidate algorithms: generalized linear models, generalized additive models, Bayesian linear model, support vector machine, re cursive partitioning and regression trees, elastic net, neural network, local polynomial regression, and random forest.*' |
| Gerberich *et al.* 2011 | **Page 296:**  '*A causal model, based on literature, including a similarly designed study and expert knowledge, was based on the epidemiological model of human damage involving the dynamic interactions of host(s), agent(s), and vehicle(s) (or vectors) within the environment. The associations between demographic factors or environmental exposures and PA or NPV were examined by using logistic regression models. Directed acyclic graphs (DAGs) were derived from the causal model and used to select the minimum sufficient set of potential confounders for the relevant exposures of interest, following the methods described by Greenland et al*'  **Table 5 (footer):**  '*‡Adjusted for gender, race, and age. §Adjusted for gender, race, age, and marital status. ¶Adjusted for gender, race, age, marital status, and education. ∗∗Adjusted for education, gender, race, age, marital status, school type, years worked in current school, and grade taught. ††Adjusted for education, gender, race, age, marital status, job class, and total years worked. ‡‡Adjusted for education, gender, race, age, marital status, total years worked, activity, and job class. §§Adjusted for education, gender, age, race, marital status, job class, total years worked, years worked in current school, and school type. ¶¶Adjusted for education, gender, race, age, marital status, years worked in current school, total years worked, activity, job class, and school type. ∗∗∗Adjusted for education, gender, race, age, years worked in current school, total years worked, activity, job class, and school type.*' |
| Gerberich *et al.* 2014 | **Page 327**:  '*For each exposure of interest, confounders were selected a priori for multivariable logistic regression, using the counterfactual principles in Maldonado and Greenland and based on directed acyclic graphs (DAGs). The assumption behind the regressions is that adjustment for confounders can create a valid counterfactual substitute for the comparison of exposed with unexposed members of the target population. A master DAG was created for the outcome of interest; then for each causal factor of interest, the DAG was reduced to the relevant pathways to identify a minimal set of confounders to be included in the regression. This method identifies parsimonious models and excludes covariates that should not be entered into the regression lest they introduce bias.*'  **Table 1 (footer):**  '*z Age, gender, race. x Age, gender, education level. jj Age, gender, race, education level, years worked as educator, years worked in school, school type. { Age, gender, race, education level, primary professional activity, years worked as educator, years worked in school, school type, grades taught at school. # Age, gender, race, education level, years worked as educator, years worked in school, school type, grades taught at school. aa Type of school, school location, job classification, grades taught, student impairment status. yy Other personnel in classroom, primary professional activity, job classification, number of students on average per day, hours worked during month. zz Type of school, school location, primary professional activity, job classification. xx Type of school, school location, primary professional activity, job classification, grades taught at school. jjjj Type of school, school location, primary professional activity, grades taught. {{ Age, gender, race, education level, years worked as educator, years worked in school, school type, grades taught at school. ## Age, gender, race, education level. aaa Age, education level, type of school, school location. yyy Age, gender, race, education level, years worked as educator, years worked in school, type of school, school location, job classification, grades taught at school. zzz Type of school, school location, school size, grade levels taught at school, policies, administrators’ attitude, student demographics, student impairment status, percentage of students receiving free lunches, other personnel in classroom. xxx Type of school, school location, school size, student impairment status, percentage of students receiving free lunches. jjjjjj Type of school, school size, primary professional activity, job classification, grades taught at school, number of students on average per day, hours worked during month.*' |
| Gillam *et al.* 2017 | **Page 4:**  '*Confounders were included, based on clinical knowledge and bias assessment using the method for directed acyclic graphs (DAG) outlined by Shrier and Platt (2008) and a DAG graphical tool combined with a change in estimate approach.*'  **Table 3 (footer):**  '*a Men: Heart failure hospitalization – adjusted for (RxRisk-V) age, cement, arrhythmia, hypertension, ischemic heart disease angina, and ischemic heart disease hypertension. Death – adjusted for (RxRisk-V) age and cement. b Women: Heart failure hospitalization – adjusted for (RxRisk-V) age, arrhythmia, hypertension, and IHD hypertension. Death – adjusted for (RxRisk-V) age and cement.*' |
| Gillott *et al.* 2017 | **Page 357:**  '*The minimum sufficient set of confounders was identified using a Directed Acyclic Graph (DAG) tool, with guidance from a priori clinical knowledge. The continuous variable age was discretized to reflect those categories considered to be of clinical relevance (,65, 65–74, and ≥75); the remaining covariates were included dichotomously, denoting whether or not the diagnosis was present. Data were then stratified by ethnicity, and subgroup analyses were performed to investigate the differential odds of AF, given each of the potential risk factors; separate multivariable logistic regression models were fit to characterize the exposure–outcome relationships for each risk factor as directed by individual DAGs.*' |
| Gocke *et al.* 2014 | **Page 353**:  '*For regression analyses, directed acyclic graphs (DAGs) have been set up to minimize bias in confounder selection. DAGs contain single-headed arrows which characterize causal and temporal relationships between variables. They represent a new graphical and analytical approach to explore causal structures, identify confounders and determine adjustment sets. We used DAGitty for DAG creation and determination of minimal sufficient adjustment sets. The final DAG is presented in Suppl. Fig. 1. Accordingly, regression models were adjusted for age, gender, education, smoking status, abdominal obesity, alcohol consumption, physical activity, diabetes and dyslipidaemia assessed at baseline. Abdominal obesity was identified as an effect modifier (P for interaction <0.10) and thus, stratified analyses were presented.*' |
| Gray *et al.* 2017 | **Page 446:**  '*A base model was constructed using Directed Acyclic Graph (DAG) for confounder selection using graphical interface software DAGitty (http://www.dagitty.net V.2.2, 2014), (see online supplementary figure S2). DAG minimal adjusted set of variables were selected using a step-by-step approach…*  '*Confounders and interactions were included in the final model for each outcome if they were associated with p value of <0.5 and/or the association had biological plausibility based on previous literature, as shown in online supplementary tables S2–S10.*' |
| Gray *et al.* 2017A | **Page 213**:  '*The base model was constructed for lung function outcomes using directed acyclic graphs, which graphically illustrate assumptions made regarding the causal relationships between variables of interest with the goal of minimizing bias for the effect estimates in our chosen models. Initial relationships between clinically relevant variables were entered into a directed acyclic graphs based on previous literature and univariate associations in this data set. It was implemented in a graphical interface software DAGitty (www.dagitty.net version 2.2, 2014). A minimal set of confounders was selected by automated approach in DAGitty described here. As a result, for each lung function outcome the base model consisted of matched lung function at 6 weeks, sex, body mass index for age z score, ethnicity, socioeconomic status quartile, and gestation length.*' |
| Grice *et al.* 2007 | **Page 793:**  '*A priori causal models and directed acyclic graphs (DAGs) guided selection of potentially confounding variables.*'  **Table 4 (footer):**  '*aAdjusted for total hours worked, type of delivery (vaginal vs. cesarean section), satisfaction with work balance, leave status, role preference, job satisfaction, supervisor support, coworker support, marital status, ability to take time off, ability to change hours, ability to take work home, household income, education, race, and age. yAdjusted for planned vs. unplanned pregnancy, delivery type, satisfaction with work balance, leave status, role preference, job satisfaction, supervisor support, co-worker support, marital status, ability to take time off, ability to change hours, ability to take work home, household income, education, race, age, and number of children.*' |
| Grice *et al.* 2010 | **Page 43:**  '*The causal model (Figure 1) illustrates which environmental factors and personal characteristics may influence the likelihood of experiencing PA in the workplace. A priori causal models and directed acyclic graphs (DAGs) guided selection of potentially confounding covariates included in the multivariate logistic regression. This method is described by Greenland, Pearl and Robins and illustrated by Hernán, Hernandez-Diaz, Werler, & Mitchell. To control for potential biases associated with non-response and unknown eligibility, database characteristics such as age, gender, licence type and home address that were available for both responders and non-responders were used to adjust for differential response and eligibility; this technique is widely used to minimize such bias*'  **Table 2 (footer):**  '*b Model 2: Multivariate model was adjusted for age, hours of patient contact, years worked, licence type, primary population, primary professional activity, education, unit worked, light level and stress level. c Model 3 (adjusted multivariate): Adjusted for all variables in Model 2 and non-response and eligibility.*' |
| Grill *et al.* 2014 | **Page 5:**  '*To ascertain whether the proposed association between GSE and vHI was affected by confounding, intermediate variables, or common effects, DAGs were used. The graph was based on characteristics with known association to GSE and vHI, specifically anxiety, depression, somatization, age, alcohol consumption and level of education. The resulting minimally sufficient adjustment set was then entered into a multiple logistic regression model to determine the effect of GSE and covariates on primary and secondary outcomes.*'  **Page 6:**  '*Fig. 1 shows the DAG along with references describing the empirically confirmed association between two variables. The DAG algorithm identified a minimally sufficient adjustment set of GSE score and age. Sensitivity analyses with different DAG structures yielded similar sets.*' |
| Grundy *et al.* 2017 | **Page 240:**  '*We selected confounders using a directed acyclic graph. Directed acyclic graphs (DAGs) are causal diagrams that illustrate the direction of relationships between variables of interest and other unknown confounders. They have been suggested as an alternative to traditional epidemiological methods of confounder identification as they explicitly display and facilitate the causal-inference process. Specifically, in contrast to statistically driven methods of model building, DAGs focus on the theoretical causal relationships between variables when identifying potential confounders. They are used in epidemiology to identify a confounder set (minimally sufficient set) that will control for potential confounding between an exposure and an outcome, given the hypothesized causal relationships. We used DAGitty software to create a DAG and identify a minimally sufficient adjustment set for the association between shift work and overweight and obesity. The DAG is shown in Figure 1; we identified a minimally sufficient adjustment set that included age and socioeconomic status, the latter as determined by education and family income, and adjusted all multivariate models for these variables.*' |
| Guimarães *et al.* 2016 | **Page 12**:  '*According to our conceptual framework that links social mobility to carotid IMT (Fig. 1), we used a directed acyclic graph (DAG) to ascertain the minimal sufficient adjustment set of variables for estimating the total and direct effects of social mobility on IMT (Westreich and Greenland, 2013). In theory, a collider variable (i.e., a common cause of our mediating factors and the outcome IMT) would not be likely in our DAG, allowing us to condition on lifestyle and cardiovascular factors without introducing collider bias. We used the software DAGitty, available on http://www.dagitty.net/. First, we assessed the association between background characteristics and IMT. Second, we assessed the association of lifestyle and cardiovascular factors with IMT, adjusting for background characteristics. Then, following our DAG (Fig. 1), we assessed the association of social mobility with IMT, adjusting for background characteristics (total effect). Next, we added in this model, one at a time, those lifestyle and cardiovascular factors that were statistically associated with IMT (p < 0.05) and were also associated with social mobility (p < 0.05, according to chi-square tests) (direct effect).*' |
| Gunathilake *et al.* 2016 | **Page 3**:  '*We used the DAGGITY program to construct a causal diagram to determine the necessary confounder adjustments (Supplementary Figure 1). The minimum set of confounders needed to control for the mediated effect of both triglycerides and CRP were cardiovascular disease, alcohol use, depression, and physical activity. The minimum set of confounders necessary to control for the total effect of BMI were age, diet, education, and gender.*' |
| Harris-Adamson *et al.* 2016 | **Page 729**:  '*To help guide this analysis, we constructed causal diagrams (directed acyclic graph (DAG)) to visually encode the hypothesised relationships between covariates, time-varying exposures and CTS (figure 1A, B), and used them to perform a pathway analysis. DAGs are useful tools to identify potential confounders that may bias observed exposure–response associations and to distinguish confounders from intermediate variables on the causal pathway for which adjustment is inappropriate.*'  **Page 730**:  '*Guided by DAGs, the models were adjusted for potential confounding by personal factors related to exposure and outcome that were not on the causal pathway. Using the forward stepwise procedure, variables were retained in the model if inclusion resulted in a change of the effect estimate of the primary exposure variable by 10% or more17. Ultimately, age, gender, BMI and study site were included in all models. Models where specific biomechanical exposures were the primary exposure of interest were adjusted for dissimilar biomechanical exposures (ie, exposures of a different type).9 For example, the relationship between peak hand force and CTS was adjusted for total repetition rate and wrist posture, whereas the model assessing the relationship between forceful repetition rate and CTS was only adjusted for wrist posture. Assessment of confounding of one class of exposures by another (eg, biomechanical, work psychosocial) used the same process and criteria described above.*' |
| Harskamp-van Ginkel *et al.* 2015 | **Page 4**:  '*We constructed a directed acyclic graph (DAG) to identify confounders and effect modifiers of the relationship between maternal BMI and ever asthma at age 7–8 years, using DAGitty (S1 Fig). Vectors are created for each expected effect a variable has on other variables. For example: in our cohort, ethnicity is a known predictor for offspring BMI, so a vector is pointing from ethnicity to offspring BMI. Prepregnancy BMI has been associated with higher rates of pre-eclampsia and preterm birth, higher rates of caesarean section and extreme high birth weight. Ethnicity and maternal education are correlated and have similar effects. The minimal adjustment set for estimating the total effect of maternal BMI on ever asthma at age 7–8 years is: non-western ethnicity; maternal age; maternal education; and parental asthma.*' |
| Harville *et al.* 2017 | **Page 229**:  '*Generally, three sets of models were examined. The first was unadjusted. The second included covariates identified based on a directed acyclic graph (DAG) using the DAGitty program. The same covariates were included in each model: age, sex, race, smoking, alcohol use, education, income and physical activity. Depending on one’s causal model, adult BMI could be a confounder (if higher genetic propensity to be large leads to higher birthweight and higher adult BMI) or an intermediate (if intrauterine undernutrition leads to later adiposity). In the first case, adjustment would be appropriate; in the second, it could in fact create a spurious association. The final set of models includes the BMI adjustment.*' |
| Herich *et al.* 2017 | **Page 60**:  '*To identify the covariates to be included in the model, we drafted a causal diagram (directed acyclic graph) representing the hypothesized causal relations relevant to our main research question (Figure 1; available at www.jpeds.com). As suggested by Williamson et al, we considered as potential confounders the common causes of both exposure, maternal education, and breastfeeding outcomes. Thus, we adjusted for maternal age and country of birth (coded as Italy, Western Europe and US, or other countries); region was included as a random effect in multilevel models.*'  **Table III (footer):**  '*†The aORs for education are adjusted for mother's country of birth and age. ‡The aORs for education are adjusted for mother's country of birth, age, and exclusive maternal milk at initial feedings. §The aORs for education are adjusted for mother's country of birth and age, and stratified by exclusive maternal milk at initial feedings because of the interaction*' |
| Hickson *et al.* 2017 | **Page 268**:  '*In order to select appropriate potential confounders, and to make explicit our assumptions about the causal connections between the demographic variables, we drew a directed acyclical graph, 15 16 or DAG (see Fig. 1) using DAGitty software. The arrows represent assumed causal links. Education and migration status are both ‘past’ and therefore have been placed higher in the causal pathways; ethnicity is considered conditional on being in the study and therefore migrant status was assumed to be causally upstream. All of these variables are also thought to have a causal relationship with the outcomes, which is not shown in the diagram. We used the DAGitty software (http://www.dagitty.net/) to identify minimal sufficient adjustment sets of potential confounders. These confounders were used in logistic regression. By identifying confounders in this way, as opposed to including potential confounders on the basis of association with the exposure of interest and the outcome, or simply including all other variables, we aimed to avoid over-fitting the model with variables on the causal pathway in the adjusted models.17 As a result, each of the associations between the variables and the outcomes was adjusted for by a different set of potential confounders.*'  **Table 1 (footer):**  '*In the adjusted models, associations with ethnicity were adjusted by age, geography and migrant status; education was adjusted by age, ethnicity and living in London; income was adjusted by age, ethnicity, education, migrant status and living in London; household was adjusted by age, ethnicity, education, migrant status and living in London; migrant status was adjusted by age; living in London was adjusted by age and migrant status; and being attracted to women was adjusted by age, ethnicity, education, migrant status, living in London and income.*' |
| Hinkle *et al.* 2013 | **Page 1345**:  '*We used a directed acyclic graph to inform decisions as to which variables to include in the models (Figure 1). We made the assumption that an unobserved latent ‘lifestyle’ variable influenced both mother’s prepregnancy BMI and children’s development (through family enrichment). Based on the latter assumption, the minimally sufficient set of variables required to assess the total effect of prepregnancy BMI on development was determined to be demographics, smoking and enrichment.*' |
| Hirsch *et al.* 2016 | **Page 62**:  '*We selected model covariates (age, gender, education, and having access to a vehicle) using a Directed Acyclic Graph (DAG) and a priori knowledge on sociodemographic and resource characteristics that act as confounders.*' |
| Hirsch *et al.* 2017 | **Page S61:**  '*Adjustment for confounding variables was determined a priori using first Directed Acyclic Graphs and DAGitty and then statistical tests of associations with both the exposure and outcomes. To estimate the association between Street Smart WalkScore™ and the MOVES we developed a linear regression models, which included MOVES and each subdomain as the dependent variables, Street Smart Walk Score™ as independent variables and age, sex, education, retirement status, and self-perceived health as relevant covariates. As no covariate significantly (p<0.1) modified the association between Street Smart Walk Score™ and MOVES, we did not include interaction terms in the final models*' |
| Hoyer *et al.* 2015 | ***Page 842:***  '*Directed acyclic graphs (DAGs) were used to select the confounders included in the final models using DAGitty software. All final multivariate models were adjusted for maternal age at birth (continuous, years), parity (dichotomous, 1, 2, > 2), maternal smoking during pregnancy (dichotomous, serum cotinine ≤ 10/> 10 ng/mL), maternal education (dichotomous, unskilled/skilled) and maternal prepregnancy BMI (continuous, kilograms per meter squared). The models of WHtR were additionally adjusted for child age (continuous, weeks) and sex. The pooled analysis was furthermore adjusted for country.*' |
| Jacobson *et al.* 2017 | **Page 191:**  '*Adjusted models included site geographic region and maternal covariates that were associated with the outcome with P < 0.20 in univariable analyses and deemed to be confounders if they changed the estimate of exposure >10% or improved estimate precision for that outcome in the adjusted analysis.*'  **Page 191:**  '*Based on a priori knowledge, directed acyclic graphs were drawn using DAGitty v2.0 (Utrecht, The Netherlands) to identify potential confounders of the relationship between the main exposure (ARV type) and growth outcomes at 2 years of age. Potential confounders measured at or before cART initiation included mothers’ age, being born in mainland US, race/ethnicity, speaking only English at home, household income, first-trimester use of illicit drugs, alcohol or tobacco during the pregnancy, CD4% and HIV RNA > 10,000 copies/mL before cART initiation in pregnancy, prepregnancy BMI, ever-used ARV before this pregnancy, birth year of child and geographic region of the clinic.*'  **Table 4 (footer):**  '*Adjusted models include the following covariates for each outcome by trimester of cART initiation: WTZ 1st (site region, alcohol, tobacco and language at home); WTZ 2nd (site region and language at home); LNZ 1st (site region, alcohol and tobacco); LNZ 2nd (site region, tobacco, language at home and living arrangement); WFLZ 1st (site region, alcohol and language at home); WFLZ 2nd (site region, tobacco and language at home); TSFZ 1st (site region, alcohol, tobacco and birth year); TSFZ 2nd (site region and living arrangement); HCZ 1st (site region, alcohol and tobacco); HCZ 2nd (site region, tobacco, income and language at home). Marital status, race/ethnicity, birth outside the mainland US and prior ARV use did not cause confounding and were not included*' |
| Jämsä *et al.* 2017 | **Page 371:**  '*To create a multivariable model, we used a directed acyclic graph (DAG) to establish a causal relationship between variables and to find a minimal adjustment set to minimize bias. Due to the complexity of the causal relationships among all variables associated with AKI, we used the Dagitty tool (Textor et al. 2011) to create the multivariable model. We chose duration of operation as an exposure variable and AKI as an outcome variable. The Dagitty model showed that BMI, fixation technique, bilateral operation, and operation type and joint was the minimal sufficient adjustment set to minimize bias. We also included the ASA classifications and preoperative eGFR in the multivariable model according to clinical experience (see Supplementary data) and because these variables were interesting for our study hypothesis. We performed a sensitivity analysis that included only the minimal adjustment set to make sure that the results remained unchanged when the ASA classifications and eGFR were added to the model.*' |
| Janitz *et al.* 2017 | **Page 169**:  '*To evaluate confounding, we used a directed acyclic graph including the variables of race/ethnicity, age at diagnosis, gender, birth order, exposure to electromagnetic fields, urbanization, and maternal variables of education, age, and smoking during pregnancy, which was analyzed using DAGitty. The minimally sufficient set of confounding variables included urbanization, maternal education, and maternal tobacco use during pregnancy. We used backwards selection to quantitatively evaluate whether covariates changed the odds ratio (OR) > 20% after removal from the model. Because tobacco use was only collected from 1991 forward, we evaluated confounding among children born from 1991 to 2010 and observed < 10% change in the OR. Therefore, we analyzed the association between benzene and acute leukemia without tobacco as a confounder using all available data. Furthermore, because it was not clear whether urbanization may be a possible surrogate for socioeconomic status (SES) (Tselios, 2013) or benzene exposure, we evaluated models with and without urbanization as a potential confounder.*' |
| Jankowiak *et al.* 2016 | **Page 1171:**  '*Based upon this literature overview, relevant sets of confounders for night shift work and the cardiovascular outcomes were identified using acyclic directed graphs (Textor et al. 2011). The following covariates were included block by block in the different regression models:*'  **Page 1172:**  '*Based on the above-mentioned confounders and covariates, four different models were calculated for each outcome separately: an unadjusted, crude model (model 0); a basic model including adjustment for age and sex (model 1); a model including age and sex + occupational variables (model 2); and a model based on the adjustments of model 2 as well as of lifestyle, SES, and dispositional characteristics (model 3). The results will focus on model 2 as it contains the most relevant variables: Age and sex as well as occupational influences expressed by job complexity level, being a manager, overtime work, and noise. Model 3 will be considered as a conservative approach that covers influences of a broad spectrum, partly correcting for similar influences twice.*' |
| Johri *et al.* 2016 | **Page 3**:  '*Analytic approaches were prespecified by using covariates drawn from the scientific literature and Directed Acyclic Graphs (34) to clarify causal relations (Supplemental Figures 1 and 2, Supplemental Methods). Analyses were implemented separately for rural and urban sites. For the rural site, we analyzed data with the use of a multilevel logistic regression model with children, mothers, and households (level 1) nested in villages specified as random effects (level 2). We first estimated a model containing only a random intercept to assess clustering of variance. Model 1 included maternal health literacy as a predictor of the outcomes of interest. Model 2 represented the minimum adjustment set, adding maternal age and wealth quintile as potential confounding factors. Model 3 included a full set of level 1 covariates (maternal age, parental education, child’s birth order, child’s age, child’s sex, religion of the household, presence of a toilet facility, and wealth quintile). Model 4 included all model 3 variables and 3 additional village-level variables (village electrification, proportion of households practicing open defecation, and number of Integrated Child Development Services centers). For the urban site, we used logistic regression with fixed effects to represent measured and unmeasured sources of neighborhood variation. Models 1, 2, and 3 were otherwise identical to those for the rural site.*' |
| Jonker *et al.* 2012 | **Page 2**:  '*We used DAGitty, a web-based application based on the Directed Acyclic Graph theory, to model the causal relationship between iron status, malaria risk and any potential (measured and unmeasured) confounders. This DAG-program was used to identify sets of confounders that together fully adjust for confounding in multivariable modeling. Several of these sets of confounders (minimally sufficient adjustment sets) were identified from figure 1. The set containing HIV-status, socio economic status, age, nutritional status and study site was used for analysis, as these variables were available in our data set. Socio economic status was scored on parents’ education, job and number of assets. Chronic malnutrition defined as height-for-age, SD was used as the marker for nutritional status.*' |
| Jusko *et al.* 2010 | **Page 390**:  '*To select potential confounding variables for our regression models, we employed directed acyclic graphs (DAGs). This method provides a graphical approach to causal modeling which allows one to identify a minimally sufficient set of adjustment variables that will adequately reduce confounding while avoiding adjustment for inappropriate variables, which may actually induce confounding. To do this, two separate DAGs [one for perinatal (maternal/cord) and one for postnatal exposure] were created. We did not consider separate DAGs for individual PCB congeners or antibodies as we regarded the causal structure of these models to be similar. Thus, we selected two different sets of confounders for our models. These were: (1) ethnicity (Roma vs. other), maternal smoking before or during pregnancy (yes/no), and maternal age at child’s birth (years) for maternal and cord PCB models; and (2) sex, ethnicity (Roma vs. other), maternal smoking at 6 months (yes/no), maternal age at child’s birth (years), and infant age at the time of 6-month blood draw (days) for 6-month infant PCB models. In secondary analyses, we also adjusted for: child’s age (days) (maternal and cord PCB models), number of vaccine doses received (1 vs. 2), time since most recent vaccination (days), infant sex (maternal and cord PCB models), and Z-score birth weight, in addition to those variables already in our primary models formed by the DAG. Variables for our secondary models were selected from other potential confounders reported in the literature and from variables noted in our DAGs to be predictors of the outcome, but which did not necessarily meet the criteria of a confounder, i.e., were not associated with PCB concentrations.*' |
| Kalapatapu *et al.* 2017 | **Page 186**:  '*The variables selected for inclusion in the adjusted analyses were based on the previous publication on this topic and depicted using a directed acyclic graph (DAG; using http://dagitty.net/)... Age, race and sex were conceptualised as confounding variables. PTSD and depressive symptoms were conceptualized as mediating variables of trauma exposure (Figure 2). Analyses were partially adjusted for baseline age, baseline sex, and baseline race. Then, analyses were fully adjusted for baseline age, baseline sex, baseline race, year-updated PCL sum score and year-updated PHQ-9 score.*' |
| Kalapatapu *et al.* 2017A | **Page 40**:  '*We used a directed acyclic graph (DAG)-based approach to select the variables to be included in the regression models… We conceptualized age, education, sex, race, socioeconomic status (i.e., income and occupation), and smoking to confound the association between alcohol use and cognitive performance; most of these variables were adjusted for in the regression models (see Results section for further comments on income and occupation). Whereas, we conceptualized medical problems (e.g., heart problems, diabetes), poor sleep and depression to partially mediate the association between alcohol use and cognitive performance; mediators were not adjusted for in the regression models, since adjustment for mediators would decrease the total effect of alcohol use on cognitive performance.*' |
| Karahalios *et al.* 2014 | **Page 2**:  '*A causal diagram was used to choose confounding variables; these were: country of birth, sex, baseline body size measurement, quintile of socioeconomic status, cumulative smoking status, and the following lifestyle measures at baseline and wave 2: an indicator variable of whether the participant lived alone, Mediterranean diet score, and physical activity (Figure S1).*' |
| Karahalios *et al.* 2016 | **Page 2:**  '*A causal diagram was developed and the following confounding variables were included in the models: country of birth, sex, quintile of socioeconomic status, family history of any cancer, the anthropometric measurement at baseline, cumulative smoking status, physical activity and Mediterranean diet score at baseline and wave 2 (Additional file 1).*' |
| Karlsen *et al.* 2017 | **Page 5:**  '*We built multivariable-adjusted models including a set of covariates defined by directed acyclic graphs (DAGs). These initial adjusted models for maternal serum-POP concentrations included maternal nationality, age at delivery, prepregnancy BMI, gestational weight gain, parity, maternal smoking and fish intake during pregnancy, child sex and birth weight. The initial adjusted model for the child’s 5-year serum-POP concentrations included maternal nationality, age at delivery, prepregnancy BMI, maternal smoking during pregnancy, child sex, exclusive breastfeeding duration and the child’s fish intake at age 5 years. We then evaluated additional potential confounders in the associations of maternal serum-POP concentrations (gestational diabetes, maternal alcohol consumption, type of delivery, year of birth) or child serum-POP concentrations (year of birth) and the study outcomes in the initial adjusted models using forward selection (i.e., adding one variable at the time in the model). From those covariates, only the type of delivery changed the coefficients for the associations of maternal serum-ΣPCBs, p,p’-DDE, PFHxS and PFNA with child’s BMI by more than 10%, and it was therefore retained in all maternal serum- POP models.*' |
| Kebede *et al.* 2017 | **Page 2:**  '*To model the putative association among exposure, outcome, and covariates in the present study, directed acyclic graphs (DAGs) were used (Appendix Fig. 2)… For the present analyses, we constructed a series of slightly different DAGs (e.g., inclusion/exclusion of arrows with limited evidence) to identify a robust confounder set. Finally, with periodontal destruction as the exposure and mortality as the outcome, the minimal sufficient adjustment set for the primary DAG (Appendix Fig. 2) included the following: age, sex, socioeconomic status, obesity, smoking, physical activity, and oral health behaviour.*'  **Page 3:**  '*All models were adjusted for the minimal sufficient adjustment set, which was derived from DAG analyses…*  **'***Mediation models were built to analyze whether periodontal destruction has an indirect effect on mortality via diabetes. Following the concepts of MacKinnon et al. (2007) and Valeri and Vanderweele (2013), we estimated the direct, indirect, and marginal total effects.*' |
| Kendrick *et al.* 2016 | **Page 147**:  '*Directed acyclic graphs included age, sex, IMD, and distance from hospital as adjusted variables and the potential confounding variables, which included the following: the number of children in family; race/ethnic group (white/other); single- adult household (yes/no); the Child Behavior Questionnaire Score (activity and high-intensity pleasure subscales, linear term); Hospital Anxiety and Depression Scale (linear term); Parenting Daily Hassles Scale (parenting tasks subscale, linear term); hours of out-of-home child care per week (linear term); ability to climb, measured using 8 questions with 3-point Likert scale responses from not likely to very likely (grouped as all 8 responses, not likely; at least 1, quite likely; 0, very likely; and at least 1, very likely); first child (yes/no); and the starred exposures listed above. Unemployment, receipt of benefits, nonowner occupation, overcrowding, child health, and quality of life were not included in DAGs because the IMD contained unemployment, income, housing, and health do- mains. Not having a car was not included in DAGs because analyses were adjusted for IMD and distance from hospital.*'  **Page 148**:  '*Odds ratios (ORs) and 95% confidence intervals were estimated using conditional logistic regression adjusted for neighborhood deprivation, distance from hospital, and confounders identified from DAGs.*' |
| Kerschberger *et al.* 2012 | **Page 4**:  '*Potential confounders were determined a priori using directed acyclic graphs (DAGs, described in Figure S1), and were included in a multivariate proportional hazards Cox regression model.*'  **Page 4**:  '*We also performed other sensitivity analyses (SA). Though not identified in DAGs as potential confounders, we included the variables TB classification and TB patient category (SA 1), excluded all patients who initiated TB treatment in another facility (SA 2), and categorized continuous variables (SA 5).* '  **Table 3 (footer):**  '*Adjusted hazard ratios (HR) of ART initiation after integration compared to before integration in alternative Cox proportional hazards models. The baseline model is the one presented in table 2 and includes the following variables: gender, age, CD4 count and previous TB initiation. SA, Sensitivity analysis; (n), number; SA 1: inclusion of the variables TB classification (pulmonary TB; extrapulmonary TB; both pulmonary and extra-pulmonary TB) and TB patient category (new TB case; re-treatment TB case) into the model; SA 2: exclusion of patients transferred in from other TB services; SA 3: exclusion of patients with unknown exact ART initiation date during TB treatment; SA 4: patients with unknown exact ART initiation date during TB treatment assumed to have initiated ART in the middle of TB treatment; SA 5: categorization of continues variables (age, sex, TB Rx start outside of clinic); SA 6: only patients with CD4 cell counts #200 considered as according to national guidelines*'*.* |
| Khalifa *et al.* 2016 | **Page 3:**  '*We constructed an initial directed acyclic graph (DAG) for a number of variables of interest (see figure 2).*'  **Page 3:**  '*A multivariable regression model was then built using a forward regression selection approach. The variable with the smallest overall p value from the crude analysis was selected first and incorporated into the model. Next, each variable was included in the model in turn and a likelihood ratio test (LRT) was performed. The process was repeated until only variables with p>0.05 remained.*'  **Table 3 (footer):**  '*Adjusted for history of violence, exclusive breast feeding and regular prenatal vitamins to give a direct effect of age on PND according to the DAG. §Adjusted for age in years according to the DAG.*' |
| Kharmats *et al.* 2014 | **Page 1008**:  '*Regression models controlled for age as a continuous variable, sex as a categorical variable, educational attainment as an ordinal variable (0 = less than high school, 1 = high school equivalent, and 2 = some college or beyond), and income ratio to the 2012 federal poverty threshold as a continuous variable. The federal poverty threshold was calculated on the basis of an estimated household income by taking the midpoint of participants’ income category and dividing it by the 2012 federal poverty threshold. The poverty threshold was based on the number of household members, number of children ,18 y old, and the respondent’s age. The decision to control for these variables was made after developing a directed acyclic graph (see Supplemental Figure 2 under* '*Supplemental data*' *in the online issue) and finding the minimally sufficient set of covariates (20, 21).*' |
| Kim *et al.* 2013 | **Page 512**:  '*To identify a minimal adjustment set of covariates that are sufficient to control confounding, a directed acyclic diagram (DAG) was drawn using DAGitty v.1.1 (http://www.dagitty.net). Final multivariable models included regular use of NSAIDs, percent of energy intake from saturated fat, BMI, and family history of breast cancer as covariates.*' |
| Kim *et al.* 2015 | **Page 581:**  **'***Multiple linear regression models were used to adjust for the following potential confounding factors identified using a directed acyclic graph (DAG) analysis*'  **Page 581:**  '*In addition to the aforementioned variables, we adjusted for the residual confounding effects of correlated life-style habits by including race (non-Hispanic white or others),education (≤high school, some college, college degree, or advanced degree) and physical activity (<2.1 2.1–<9.6. 9.6–<20, or ≥20 metabolic equivalent [MET]-h/wk) in additional multivariable model*' |
| Klassen *et al.* 2014 | **Page 1359:**  '*The models were adjusted for covariates selected on the basis of the theory of directed acyclic graphs, i.e. age at diagnosis, pre-diagnosis BMI (17 – < 25, 25 to < 30, < 30 kg/m 2 ), smoking status at diagnosis (current, former, never), sports in the year before diagnosis (none, < 0 – 15 MET ∗ h/wk, < 15 – 35 MET ∗ h/wk, < 35 MET ∗ h/wk), sports during adolescence (none, non-competitive, competitive), walking (0 – 1 5 h/wk, < 1 – 3 5 h/wk, < 3 – 5 5 h/wk, < 5 h/wk) and cycling (none, < 0 – 1 h/wk, < 1 – 3 h/wk, < 3 h/wk) in the year before diagnosis, use of beta-blockers, or pre-existing cardiac diseases. Sensitivity analyses were performed by including hemoglobin level, trastuzumab treatment, hormone treatment, or type of chemotherapy (taxanes, anthracyclines), where the causal direction of the association with cardiopulmonary fitness is unclear. We also checked the parsimonious models including only significant covariates and those that changed the treatment estimate by < 10%, but there were no substantial changes in the results. In addition, type of chemotherapeutic agent was further evaluated among patients who have started chemotherapy using a model that included categorized the variable as taxane use only, anthracycline use only, or use of both.*' |
| Kobayashi *et al.* 2017 | **Page 252:**  '*Covariates were selected based on associations between dependent and independent variables observed in our data or previous studies: in DNA methylation models, we adjusted for maternal age, maternal education, infant sex, maternal smoking during pregnancy, and blood sampling period; in birth size models, we adjusted for gestational age, maternal age, prepregnancy BMI, parity, maternal education, maternal smoking during pregnancy, and infant sex. We additionally implemented directed acyclic graphs using DAGitty version 2.3 for covariate selection, and confirmed that the covariates selected above were neither colliders nor intermediates.*'  '*Adjusted for maternal age, maternal education, maternal smoking during pregnancy, infant sex, and maternal blood sampling period*' |
| Kowall *et al.* 2016 | **Page 2:**  '*We used the software DAGitty to identify a minimally sufficient adjustment set.The minimal adjustment set included age (continuous), sex, weight at baseline, alcohol intake (g day-1), smoking (current, former, never), accordance with dietary guidelines (low, medium, high), physical activity (T0: metabolic equivalents per week; T1: amount of sport assessed as ⩾2h, 1–2h, 0–<1h), school education (low/medium/high), marital stage (married and living together (yes/no)), subjective health status (‘very good/good’ versus ‘satisfactory/poor/very poor’) and stress (continuous). The minimal adjustment set additionally included sleep duration for siesta as the exposure of interest, and regular sleep disturbances for sleep duration as the exposure of interest. Coffee consumption, depression and hypertension were included in the directed acyclic graph but were not selected for the minimal adjustment set. In the linear regression analyses, adjustment for potential confounders was performed for age, sex and weight at T0 (and weight change between T0 and T1, respectively) (model 1), and for the minimally sufficient adjustment set (model 2).*' |
| Kowall *et al.* 2016A | **Page 9**:  '*We used the software DAGitty to find a minimally sufficient adjustment set. This set included age (continuous), sex, smoking (current, former, never), alcohol consumption (g/d), daily coffee consumption (yes/no), dietary pattern index (low, medium, high), metabolic equivalents per week, school education (low, medium, high), depression (CES-D scale without sleep item, continuous), stress (continuous), BMI, systolic blood pressure, diastolic blood pressure, and use of antihypertensive medication. Adjustment for potential confounders was performed for age and sex (model 1), and for the minimally sufficient adjustment set (model 2).*' |
| Kroenke *et al.* 2016 | **Page E3:**  '*Potential confounding variables in models were selected based on subject matter expertise encoded in directed acyclic graphs, diagrams that help elucidate the causal structure relating variables under study.*'  **Page E3:**  '*To address concerns about the potential for collider bias because of the restriction of analyses to diagnosed CRC patients (eFigure 1 in the Supplement), we adjusted for prediagnosis BMI when evaluating the effects of both at-diagnosis and postdiagnosis BMI. Adjustment for chemotherapy and radiation were not suggested by the directed acyclic graph in the analysis of at-diagnosis BMI based on the time order of covariates. We nonetheless included these variables in models based on convention; adjustment had no substantive effect on associations. We considered adjustment for comorbidity but sought to avoid overadjustment because CRC and comorbidities have mechanisms in common related to BMI.*' |
| Kverneng Hultberg *et al.* 2017 | **Page 1909**:  '*The covariates included in the adjustment set were determined from a causal diagram (Supplementary Fig. 1), using directed acyclic graphs. Smoking was not included, on account of 57% missing data. This resulted in a model with the following covariates: age (years at surgery), sex (male or female), ASA class (I-IV), operating hospital, neoadjuvant therapy (none, short-course radiotherapy or chemoradiotherapy), intraoperative bleeding ( 400 ml or >400 ml), presence of diverting stoma (yes or no) and type of mesorectal excision (total or partial). Intraoperative bleeding was categorized using the median as cut-off. To account for the fact that patients operated at the same hospital did not constitute independent observations, the standard errors were adjusted for clustering of patients within hospitals, generally making the confidence intervals less narrow.*' |
| Lai *et al.* 2016 | **Page 57:**  '*We conducted time dependent Cox proportional hazards regression analyses to estimate the HR and 95% CI of the association between ambient air pollution and active TB, adjusting for major TB risk factors We used the causal diagram to decide which variables to include in the multivariable model.*'  **Table 3 (footer):**  '*aAdjusted for sex, age, body mass index, education, marital status, alcohol use, smoking status, betel nut use, personal history of TB, history of TB in family, and median annual income.*'  **Table 4 (footer):**  '*Adjusted for sex, age, body mass index, education, marital status, alcohol use, smoking status, betel nut use, personal history of TB, history of TB in family, and median annual income.*' |
| Launay *et al.* 2014 | ***Page 2:***  '*To identify confounding variables, we built a theorical causal diagram between optimality of care and outcome (dead/alive at discharge from hospital) based on the published pathophysiological concepts of severe sepsis (Figure S1) and adapted from this a ‘‘realistic’’ causal diagram between optimality of initial care (before admission to a PICU) and outcome considering the available data and using DAGitty software (Figure S2).*'  ***Page 3:***  '*Covariables tested on univariate analysis were age of children, diagnosis, sign of severe disease at the first consultation, and first consultation by a mobile medical unit. Relevant variables according to the causal diagram were included in multivariate analyses.*'  ***Page 3:***  '*We included variables considered associated with suboptimal care (Figure S2).*' |
| Lehnich *et al.* 2016 | **Page 5**:  '*To address confounding, we set up a directed acyclic graph with the covariates age, gender, diseases as a group, de- pressed mood, education level, employment, physical activity, alcohol intake, intake of hypnotics and intake of drugs with a sleep promoting effect. The derived minimally sufficient adjustment set included age, gender, diseases, alcohol consumption and education. In the model statement we re- placed the variable diseases with the variables for coronary heart disease, diabetes mellitus, arthrosis, asthma, thyroid diseases and overall health status.*' |
| Liang *et al.* 2017 | **Page 4**:  '*The minimum set of variables to enter the adjusted model was identified in a directed acyclic graph using the DAGitty program required to enter the adjusted model was identified in a directed acyclic graph using the DAGitty (version 2.0 alpha, Johannes Textor, Luebeck, Germany). Models were adjusted for birth program (version 2.0 alpha, Johannes Textor, Luebeck, Germany). Models were adjusted for weight, gestational age, maternal age at childbirth, maternal education level, paternal education level, birth weight, gestational age, maternal age at childbirth, maternal education level, paternal region, sex, and year of birth (Figure S1).*' |
| Liebers *et al.* 2011 | **Page 920:**  '*Geschlecht, Alter und der Komplex ungemessener Störgrößen (U) qualifi zieren sich entsprechend der DAG-Theorie als minimal suffizientes Variablen-Set, für das adjustiert werden sollte, um die Beziehung Beruf→AU Arzt (→ AU GKV ) ohne Störung zu messen.*' *[Gender, age and the complex of unmeasured disturbance variables (U) qualify according to the DAG theory as a minimally sufficient set of variables, for which adjustments should be made to measure the relationship profession → AU doctor (→ AU GKV) without disturbance.]*  **Page 920:**  '*Als MSAS werden durch das Programm die Variablen „Alter*'*, „Geschlecht*' *und „U*' *vorgeschlagen. Bei gezielter Vorgabe einer Adjustierung auch auf den Faktor „GKV*' *werden als MSAS die Variablen „Alter*'*, „Geschlecht*'*, „U*' *und „GKV*' *ausgewiesen. Das im Projekt verwendete Set (Alter, Geschlecht, GKV) wird als nicht ausreichend aufgrund der fehlenden Adjustierung nach U bewertet. Es erfolgt der klare Hinweis, dass die Adjustierung eine Kovariate auf einem kausalen Pfad (Beruf→GKV→AU GKV ) beinhaltet. Da die Adjustierung nach Krankenkasse aus inhaltlichen Gründen bewusst gewählt wurde, bestätigt die softwaregestützte Analyse des DAG die Interpretation per Hand.*' *[The program suggests the variables* '*Age*'*,* '*Gender*' *and* '*U*' *as MSAS. If an adjustment is also made to the factor* '*SHI*'*, the variables* '*Age*'*,* '*Gender*'*,* '*U*' *and* '*SHI*' *shown. The set used in the project (age, gender, statutory health insurance) is assessed as insufficient due to the lack of adjustment according to U. There is a clear indication that the adjustment includes a covariate on a causal path (profession → SHI → AU SHI). Since the adjustment according to health insurance was deliberately chosen for reasons of content, the software-supported analysis of the DAG confirms the interpretation by hand]* |
| Lima *et al.* 2017 | **Page 593:**  '*Logistic regression was used to estimate odds ratios (OR) adjusted for the confounding factors: maternal age and the number of children for the outcome child malnutrition. The models were adjusted for maternal schooling, number of children and family income for the outcome child excess weight. The level of significance was set at 0.05. The variables suggested to be included in the minimum set of adjustment for confounding were identified by means of a directed acyclic graph (DAG) in the DAGitty® version 2.3 program (Figure 1A and 1B). DAG is a graphical tool that assists in choosing variables to be included in the adjustment following the back door criterion that minimizes problems of bidirectional covariance*' |
| Linde *et al.* 2017 | **Page 81:**  '*Using a Directed Acyclic Graph, the following covariates (written in Italic) were included in the statistical model:*'  **Table 2 (legend):**  '*The finally adjusted relationship includes selected main effects by stepwise exclusion based on the significance value.*' |
| Lupattelli *et al.* 2015 | **Page 5**:  '*Confounding and mediating factors were identified with the aid of directed acyclic graphs (DAGs) using DAGitty version 2.2 (one DAG for each medication-outcome pair). Our assumptions were: eating disorder status before and/or during pregnancy precedes maternal symptoms of depression and anxiety during pregnancy; eating disorder status before and/or during pregnancy determines BMI at conception. These assumptions applied to all the eating disorder subtypes.*'  **Page 6**:  '*Then, we entered in Model 1 the minimal sufficient adjustment set of variables (i.e., age, socioeconomic, status and educational level for all medication groups) for estimating the total association between eating disorders and the outcomes of interest. In a sensitivity analysis we included BMI at conception as additional covariate in Model 1 (because of the uncertainty in the direction of the association between BMI and eating disorders); however, the observed results did not differ substantially from the main analyses. In Model 2 we entered the set of confounders from Model 1 plus additional covariates (e.g., maternal depressive and anxiety symptoms, BMI, weight gain in pregnancy, alcohol use during early pregnancy and smoking until gestational week 30) in order to estimate the direct association between eating disorders and the outcomes of interest.*' |
| Lytsy *et al.* 2013 | **Page 937:**  '*In order to minimize potential bias, the directed acyclic graph approach was used to identify appropriate models, considering variables previously reported to influence BP tracking and endothelial function. Two sets of models were investigated: a crude model and a model adjusting for age, sex, smoking status, diabetes mellitus, creatinine, baseline BMI and change in BMI during follow-up.*' |
| Magadi & Magadi 2017 | **Page 314:**  '*The perceived link between the study variables is shown in the directed acyclic graph (DAG) in Fig. 1, with direction of arrows representing perceived causal pathway, based on theoretical considerations…The modeling involved introducing various background demographic and socioeconomic characteristics (i.e., age, number of living children, marital status, education, wealth, urban/rural residence, region, ethnicity and religion) and proximate factors (i.e., desired fertility and sexual activity) directly associated with contraceptive use in the models in successive stages to investigate potential pathways of the relationships*'  **Page 314:**  '*The second part of our multivariate analysis is based on explanatory predictive modeling and focuses on predictors of contraceptive uptake among HIV-positive women. It involves a comparison of factors associated with contraceptive use between HIV-positive and HIV-negative women (Objective ii). All predictors, including factors considered as confounders and modifiers in the association between HIV/AIDS and contraceptive uptake outlined above, are considered of interest*'  **Table 2 (footer):**  '*1 Model 1 – no other factors controlled for, besides significant individual and cluster-level HIV/AIDS exposure variables. 2 Model 2 – controlling for HIV/AIDS and background confounders (i.e., age group, number of living children, educational attainment level, household wealth index, religious affiliation, ethnic group, region and urban/rural residence). 3 Model 3 – controlling for HIV/AIDS exposure factors, background confounders and proximate factors (i.e., fertility intention, marital status and recent sexual activity).*' |
| Maier *et al.* 2015 | **Page 3:**  '*Due to the complex network of potential confounders and the high potential for multicollinearity between the variables beta-blocker therapy, atrial fibrillation and hypertension, directed acyclic graphs (DAG) approach was applied using DAGitty. This was done in addition to a standard statistical approach for model building (confounder selection based on changes in the point estimate of the exposure of interest) in order to derive a minimal sufficient adjustment set of confounders that was not affected by multicollinearity. Under the assumptions made for the DAG approach (based on literature review) age, sex and stroke severity (estimated by baseline NIHSS) were selected as the primary adjustment set of choice. While hypercholesterolemia and statin therapy were not considered as confounders of an association between*' |
| Maika *et al.* 2015 | **Page 222**:  '*Under the assumption of no unmeasured confounders, the causal DAG shown in Figure 2 presents the association between confounders, exposure and outcome… Both the exposure and the outcome were measured at two time points (2000 and 2007), whereas a series of baseline confounders were measured in 1993, 1997 and 2000. The confounders included: caregiver’s age, education and employment status; household size; economic hardship; housing conditions (access to electricity, the main drinking water source and type of toilet); and place of residence. The use of confounding information measured at three survey rounds allowed for the situations where a child was raised by a different caregiver, or lived in different housing environments over the course of the study period. This model specification may also help to reduce measurement error by having multiple indicators over time. In the DAG, the child’s current schooling and caregiver’s mental health in 2000 were included because they indicate the presence of time-varying confounding so that, given past household PCE, current schooling and maternal mental health predict subsequent exposure and outcome. In addition, the DAG reflects the proposition that a child’s current schooling affects completion of at least 8 years of education and also affects cognitive function in 2007. Similarly, caregiver’s mental health in 2000 affects mental health and in turn affects child’s cognitive function in 2007.*' |
| Maretty-Nielsen *et al.* 2014 | **Page 3:**  '*The adjustment covariates were selected based on a modified version (Figure 1) of a directed acyclic graph constructed by Maretty-Nielsen et al. and included as seen in Tables 1 and 2.*'  **Table 1 (footer)**:  '*aAdjusted for age, gender, and level of comorbidity.*'  **Table 5 (footer)**:  '*aAdjusted for age, level of comorbidity, compartmentalization, depth, grade, type, location and size.*' |
| Maretty-Nielsen *et al.* 2014A | **Page 325**:  '*Directed acyclic graphs were used to depict a possible causal relationship between the prognostic factors selected, possible confounding variables, and the outcomes (Figure 2, see Supplementary data).*'  **Figure 4 (footer):**  '*Adjustment covariates were selected based on Figure 2 (see Supplementary data); no covariates were included in the analysis of age; duration of symptoms was adjusted for age and grade; tumor size was adjusted for duration of symptoms and grade.*'  **Table 3 (footer**):  '*Confounding variables were selected based on the directed acyclic graph depicted in Figure 2 (see Supplementary data): location was adjusted for histological type; depth was adjusted for duration of symptoms, size, and histological type; grade was adjusted for age and histological type; margin was adjusted for age, size, depth, location, compartmentalization, grade, and year of diagnosis; radiotherapy was adjusted for age, depth, grade, margin, and year of diagnosis; no adjustments were included in the analysis of year of diagnosis.*' |
| Martin *et al.* 2017 | **Page 2155**:  '*Using the causal relationships in the causal diagram, with the assistance of a computer graphical interface DAGitty, age at the time of diagnosis, SEER region, and year of diagnosis were determined to be the set of variables for adjustment required to minimize confounding.*' |
| Matser *et al.* 2013 | **Page 3:**  '*We constructed a causal DAG in which to map the assumed pathways between ethnicity and CT diagnosis (Figure 1). We assumed three possible main routes, two direct and one indirect (ie, through other variables). The direct pathways are biological (ie, susceptibility) and sexual risk behaviour (eg, unprotected sex); the indirect pathway is mediated by socio-economic status. Age and gender were assumed to be possible confounders of the association between sexual risk behaviour and CT, between socio-economic status and CT, and between ethnicity and CT.*'  **Page 3:**  '*To capture sexual risk behaviour, we included the following covariates in multivariable analysis: condom use, number of partners in the preceding year, average duration of partnerships, and concurrency. Ethnic mixing was considered a possible confounder of the association between sexual risk behaviour and CT. We therefore distinguished participants with only assortatively mixed partnerships from those with only disassortatively mixed partnerships and those with both types. Educational level and neighbourhood were also taken into account, because these two covariates are markers of socio-economic status*' |
| McCulloh *et al.* 2015 | **Page 4:**  '*Models used subject-matter knowledge from published literature and a priori clinical assumptions, analyzed by directed acyclic graphs (DAG), to guide statistical modeling assumptions.[ 15,16] The DAG analysis served as the basis for constructing propensity scores*' |
| Mebrahtu *et al.* 2015 | **Page 3**:  '*In order to minimise bias due to confounding and overadjustment, Directed Acyclic Graphs (DAGs) were used and models were tested using DAGitty software. Drawing a relationship between variables of interest (ie, confounding and main variables) was guided by epidemiological, biological and clinical knowledge. Online supplementary figures S1 and S2 illustrate the schematic view of adjustment and output for the list of ‘minimally sufficient’ confounding sets using DAGitty software. n assessing the effect of birth weight on wheezing disorders, ethnicity, family asthma, gender, gestational age, maternal smoking, number of live births, parity and SES were selected as ‘minimally sufficient’ set of confound- ing variables. In assessing the effect of childhood growth on wheezing disorders, birth weight, ethnicity, family asthma, breast feeding, gender, maternal smoking, parity and SES were selected as ‘minimally sufficient’ set of confounding variables.*' |
| Medenwald *et al.* 2014 | **Page 2:**  '*Respecting previous findings, we adjusted our analyses for age, anti- arrhythmic (ATC code: C01B) and anti-phlogistic medication (ATC code: A07), current smoking status, high density lipoprotein (HDL), cholesterol, glucose blood level, alcohol intake, body mass index, thyroid stimulating hormone (TSH), systolic blood pressure, and potentially QT prolonging drugs (see www.qtdrugs.org) after reevaluating possible confounders by using directed acyclic graphs (DAG). With the assumed DAG model (see Figure S1) it is possible to estimate the total effect of inflammation on QTc. However, as mediation by electrolytes (not assessed in our study) is likely the direct effect cannot be estimated.*'  **Figure S1**:  '*Directed acyclic graphs of parameters potentially influencing the association of inflammation and QT time. Minimal sufficient adjustment to estimate the total effect of inflammation of corrected QT time: age, blood pressure, blood fats/cholesterol, QT- prolonging drugs, smoking habit, and thyroid function. Minimal sufficient adjustment to estimate the direct effect of inflammation of corrected QT time: age, blood pressure, blood fats/cholesterol, QT- prolonging drugs, smoking habit, thyroid function, electrolytes.*' |
| Medenwald *et al.* 2015 | **Page 258:**  '*Considering directed acyclic graphs, we adjusted models of the education–anthropometric parameter association (Fig. 1) for the parameters listed in Table 1 (column C1). The model examining the association of anthropometric parameters with sTNF-R1 was adjusted for the confounders grouped in the column C2 in Table 1. Both sets of confounders, C1 and C2 (C = [C1, C2]), were used for the mediation analysis (see ‘Mediation analysis’).*' |
| Medenwald *et al.* 2016 | **Page 430**:  '*Regression models were adjusted for sex, systolic blood pressure, glomerular filtration rate estimated by means of the CKD-EPI formula, HbA1c, total and high-density cholesterol, anti-diabetic drugs/ self-reported diabetes mellitus, body surface area heart rate, NT-pro brain natriuretic peptide (NT-proBNP), heart rate, potentially QT prolonging drugs (see www.qtdrugs.org), sportive activity in hours per week, LVMI in non-subgroup analyses, and for interactions of considered covariates and echocardiographic/electrocardiographic tertiles if statistically significant. Covariates were identified using directed acyclic graphs.*' |
| Medenwald *et al.* 2016A | **Page 817:**  '*Variable selection and modelling was based on directed acyclic graphs*''*We adjusted for age and neighbourhood as potential confounders of the association of education and parameters of metabolic syndrome (pretreatment covariates, confounders 1 in Fig. 1). Confounders of the association of the latter and ventricular mass or ejection fraction are likely to be affected by educational level and thus have the additional character of a mediator… Potential confounders, which need to affect metabolic parameters and cardiac structure or function independently from BMI in order to be considered as confounders of the mediation analysis, were sport index[20], daily smoked cigarettes, thyroid medication, hyperthyreosis, and BMI (only when BMI was not the main mediator)*' |
| Messerlian *et al.* 2017 | **Page 722**:  '*Covariates were selected a priori as potential confounders based on substantive knowledge using a directed acyclic graph (DAG) to identify covariates associated with both exposure and outcome that were not intermediates on the causal path (see Supplemental Appendix Figure 2A). We applied two sets of main models: one for the maternal preconception window of exposure and the other for the paternal preconception window of exposure. Maternal preconception base models included: maternal age and BMI (continuous), maternal education (< college, college, graduate degree), smoking status (never vs. ever), and IVF treatment vs. non-IVF treatment. Paternal pre- conception base models included paternal and maternal age and BMI (continuous), paternal and maternal smoking (never vs. ever), maternal education (< college, college, graduate degree), and IVF treatment vs. non-IVF treatment.*'  '*We also accounted for potential confounding by partner's exposure and additionally adjusted for partners’ preconception phthalate metabolite concentrations in sensitivity analyses.*' |
| Møller *et al.* 2016 | **Page 45:**  '*Potential confounders and mediators of the association between sedentary work and IHD were identified in the existing scientific literature on general sedentary behavior and cardiovascular disease. Decisions on which confounders to include in the analyses were taken in accordance with the theory of directed acyclic graphs (DAG), see figure 1.*'  **Page 46:**  '*According to the theory of DAG, it would be sufficient to adjust the analyses for SES and wave, and stratify on age, gender and physical activity. Confounding effects of other health behaviors and BMI are already taken into account when adjusting for SES. However, due to low statistical power some deviations from this optimal design were made. Age was instead treated as a confounder in all analyses and gender was treated as a confounder when studying IHD. Because physical activity is not available before the 2000 wave, BMI was treated as a confounder in the main analyses as a proxy of physical activity. Sensitivity analyses included physical activity and were further adjusted for smoking status, alcohol consumption and decision latitude.*' |
| Mook *et al.* 2016 | **Page 3:**  '*To determine which covariates to include in analysis, variable relationships were modeled in directed acyclic graphs. We used DAGitty software to determine the minimally sufficient set of confounders needed in each model.*'  **Page 3:**  '*Model building began with the full set of hypothesized confounders including age, income, and education level (Model 1). Each model was then tested for potential effect modification by food security status and the main independent variable of interest; all significant interactions were retained. This model was then tested for interactions between each confounder and food security status or among the confounders.*'  **Page 3:**  '*We performed a separate analysis (Model 2) to investigate only the minimally sufficient set of confounders necessary for each independent variable.*' |
| Murphy *et al.* 2015 | **Page 3**:  '*We evaluated the potential for confounding by age, sex, smoking, alcohol use, NSAID use, diet (total energy, fat, and fiber intake, and red meat consumption), physical activity, and family history of CRC using a directed acyclic graph (DAG) to identify a minimally sufficient adjustment set of covariates. In addition to race/ethnicity, DAG analyses suggested the model be adjusted for sex, age, total fiber intake, total fat intake, red meat consumption, smoking, and physical activity.*' |
| Napier *et al.* 2017 | **Page 4**:  '*We used directed acyclic graphs (visualized using DAGity) to evaluate potential confounding factors plausibly associated with poor water quality and illness. The final models included age (0–4, 5–11, 12–19, 20– 34, ≥35), beach (categorical: Fairhope, Goddard, Huntington, Silver, West, Washington Park), mean number of bathers (continuous), rainfall totals from 3:00 PM the previous day to 8:00 AM on the current day (continuous), sand exposure (binary where 1 = digging in sand or burying body in sand), and, for GI illness and diarrhea, water temperature (continuous). Indicator variables representing beach were included in all models to control for differences in baseline illness among beaches.*' |
| Ng *et al.* 2017 | **Page 110**:  '*The confounders to be adjusted in the analyses were identified through a directed acyclic graph (DAG, Supplementary Fig. S1), built using DAGitty v.2.3. All relevant variables in the DAG were identified through our prior knowledge and previous research publications in the subject areas, irrespective of their availability in the dataset. According to our DAG, the minimal sufficient adjustment set for producing an unbiased estimate of the total effect of weight change on daytime sleepiness includes the following baseline covariates: age, sex, race, socioeconomic position, circadian rhythm sleep-wake disorder, diabetes status, poor night sleep, smoking status, weight, use of antidepressants, anxiolytics and hypnotics/ sedatives, weight and level of daytime sleepiness. We used education as a proxy for socioeconomic position, subjective sleep duration and sleep disturbance as a proxy for poor night sleep, and use of benzodiazepines as a proxy for use of anxiolytics and hypnotics/sedatives. The presence of circadian rhythm sleep-wake disorders was not measured in the SHHS. We did not identify any other exposure-mediator, or mediator- outcome confounders that were not already considered as exposure-outcome confounders.*'  **Page 112**:  '*We assessed the relationship between five-year weight change and daytime sleepiness at five-year follow-up using multiple linear regression, adjusting for baseline level of daytime sleepiness, and in another model, additionally adjusting for the minimum set of available confounders identified through our DAG: age, sex, edu- cation, diabetes, poor night sleep, smoking status, weight, and use of antidepressants and benzodiazepines at baseline. We did not adjust for race because 97% of the included study sample were white.*'  **Page 112**:  '*Each potential mediator was assessed in separate models. We adjusted for baseline level of the mediator in each model, in addition to the exposure-outcome confounders adjusted for in the main analysis (age, sex, education, diabetes, poor night sleep, smoking status, weight, level of daytime sleepiness, and use of antidepressants and benzodiazepines at baseline). We did not identify any other exposure-mediator or mediator-outcome confounders that were not already included as exposure-outcome confounders.*' |
| Ng *et al.* 2017A | **Page 1452**:  '*Individuals from SOReg and Itrim were matched on treatment year and the set of covariates identified through a directed acyclic graph (Supporting Information Figure S1) built using DAGitty v.2.3 (DAGitty software, Utrecht, Netherlands). The minimal sufficient adjustment set for producing an unbiased estimate of the total effect of bariatric surgery on sleep problems included age, sex, socioeconomic status, baseline BMI, and sleep problems prior to treatment. We used data on education levels as a proxy for socioeconomic status.*'  **Page 1453:**  '*We repeated the main analysis in the matched data set, additionally adjusting for the original matching variables (continuous age, continuous BMI, cumulative DDDs within 2 years prior to treatment, and treatment year), indicators of poor mental health, and history of health care contacts from 1 year to 2 years prior to treatment (measured through history of inpatient stays, outpatient visits, and filled prescription for any medications).*' |
| Ngueta *et al.* 2016 | **Page 390:**  '*For this, we used the DAGitty software and the algorithm developed for this browser-based environment, as described in detail elsewhere. The minimal sufficient adjustment sets identified for estimating the association between CWLEI and BPb included child’s age (categorized into quintiles), child’s sex, child’s ethnicity (Caucasian, other), duration of breastfeeding (in months), mother’s education level (< secondary, secondary, postsecondary), frequency of child care attendance (days per week), number of meals per day (≤ 2, > 2), and the season of blood collection (autumn or winter). We finally adjusted for these variables in the first modeling. We also performed a second set of models that were additionally adjusted for lead in paint (XRF < 1 mg/cm2, XRF ≥ 1 mg/cm2 or paint chips < 5,000 mg/kg, ≥ 5,000 mg/kg), floor dust lead loading (micrograms per square feet), and windowsill dust loading (micrograms per square feet), with dust and windowsill lead loadings modeled as categorical variables in quintiles.*' |
| Nobre *et al.* 2016 | **Page 497**:  '*The covariates were selected with the aid of a direct acyclic graph (DAG). For the adjusted association between maternal covariates and nutritional characteristics of the preschool child, each was ordered according to their specific temporal relationship: gender, maternal education, maternal age, marital status, region of residence, economic class, depressive symptoms, type of school, and the child’s age. The relationship of understanding between variables is facilitated by the identification of the temporal order. With that, we build paths based on temporality and theoretical assumptions. The model resulting from the associations was built using a DAGitty browser and the minimal sufficient adjustment to estimate the total effect of maternal exposures on the nutritional characteristics of the preschoolers included: economic class, maternal education, marital status, age of child, maternal age, and depressive symptoms.*' |
| Norris *et al.* 2017 | **Page 4**:  '*The set of variables identified by Daggity as necessary for adjustment were socioeconomic status (SES), maternal employment, smoking status, alcohol intake, BMI, level of education, maternal age and whether the mother’s job was physically active (see online supplementary figure 1 for analysis DAG). These variables were then incorporated into multivariable regression models.*' |
| Nourbakhsh *et al.* 2016 | **Page 30:**  '*We used a directed acyclic graph (DAG) to decide about the confounding factors that had to be adjusted for in the models (Fig. 1). As it is clear on the DAG; age and sex seems to be important confounder on the association between brain atrophy measures and fatigue. While physical disability and depression might be causally related to the severity of fatigue; but they are not likely to affect the volume of different brain structure.*' |
| Nowak *et al.* 2016 | **Page 244:**  '*A directed acyclic graph (DAG) was generated using DAGitty to identify the minimal set of a priori confounders (sexual behavior, size of personal network, and age) needed to adjust for the association between HIV infection and HR-HPV without introducing selection bias from over adjustment (see Figure, Supplemental Digital Content 2, http://links.lww.com/OLQ/A129, for DAG illustration). The final multivariate model adjusted for years since anal sexual debut, sexual positioning, concurrency, and size of personal network. Female partners was not included in the final model because it did not confound the main association (<10% change in estimated effect) and was significantly associated with concurrency. Age was not included in the model because it was positively correlated with years since sexual debut.*' |
| Nygaard *et al.* 2014 | **Page 477e7:**  '*We performed logistic regression with variable selection guided by an updated directed acyclic graph (DAG), in which BMI was depicted as an intermediate variable, developed using DAGitty version 2.0. Required adjustment variables were education and the age match variable. Cough and constipation were also suggested, but the cell sizes for these were too small to include. We further adjusted for number of vaginal deliveries and hysterectomy status, based on past literature, which was permissible per the DAG.*' |
| Nygaard *et al.* 2015 | **Page 40e2:**  '*We performed logistic regression with variable selection guided by an updated directed acyclic graph, in which BMI was depicted as an intermediate variable, developed using DAGitty version 2.0. (Johannes Textor, Theoretical Biology & Bioinformatics group, University of Utrecht, Utrecht, the Netherlands). We adjusted for education and age and further adjusted for number of vaginal deliveries and hysterectomy status, based on past literature, which was permissible per the directed acyclic graph.*' |
| Oddo *et al.* 2017 | **Page 3**:  '*We identified confounding factors a priori using a directed acyclic graph, which is a causal diagram used to characterize the relationship between the exposure and outcome based on theorized relationships and relationships documented in the literature. Confounders included maternal age (years), marital status (married or living together versus single, widowed, divorced), parity, number of household members, child age (months) and childcare support. Living with ones’ mother, mother-in- law, or sister served a proxy for childcare support (a binary variable).*'  **Figure 2 (footer):**  '*All models were adjusted for maternal age (years), parity, marital status (married, not married), number of household members, child age (months), and substitute childcare provider (yes, no). Models included an employmentXeducation interaction term (< primary education, ≥ primary education completed)*' |
| Oddo *et al.* 2017A | **Page 2525:**  '*We identified confounding factors a priori using a directed acyclic graph, which is a causal diagram used to characterize the relationship among variables thought to influence the primary independent and the dependent variables based on both theorized and documented relationships (see Fig. 1)(37). In all models, confounders included maternal age (years), marital status (married or living together v. single, widowed, divorced), number of household members (a continuous variable), child’s age (months), child’s sex, and living with ones’ mother, mother-in-law or sister (as a proxy variable for childcare support).*' |
| Olsen *et al.* 2015 | **Page 762:**  '*Hypothesised confounders and mediators were identified in a causal diagram (Figure 2). A three-step statistical model was used to ensure the transparency of results and to observe changes. In model 1, the estimates were adjusted for age, gender and period of diagnosis. Model 2 included further adjustment for relevant socioeconomic factors according to the causal diagram (Figure 2). In model 3, all estimates were further adjusted for comorbidity (HN-CCI), in order to observe a potential mediating role of comorbidity.*'  **Table 2 (footer):**  '*aAll estimates are adjusted for: age, gender, period of diagnosis and HN-CCI diseases. The estimates for age, gender, period of diagnosis and HN-CCI are adjusted further for educational level, cohabitation status, disposable income and degree of urbanisation. The estimates for cohabitation status are adjusted further for level of education. The estimates for disposable income are adjusted further for level of education and cohabitation status. The estimates for degree of urbanisation are adjusted further for level of education, cohabitation status and disposable income.*' |
| Olsson *et al.* 2017 | **Page 938**:  '*Covariates included in our main model (model 2) were selected on the basis of directed acyclic graphs. For plasma 25(OH)D, model 1 included age and the season of blood collection. Model 2 further included BMI, education, and physical activity. Model 3 included covariates in model 2 and smoking, diabetes, hypertension, hypercholesterolemia (as categorical variables), vitamin D supplements, and alcohol intake (yes or no). For vitamin D intake, model 1 included age and energy intake. Model 2 further included BMI, education, physical activity, and smoking. Model 3 included covariates in model 2 and diabetes, hypertension, and hypercholesterolemia (as categorical variables). For the GRS, model 1 included crude data, and model 2 included age, season of blood collection, BMI, education, and physical activity.*' |
| O'Neill *et al.* 2016 | **Page 635:**  '*The effects of all covariates based on previous literature were tested individually (supplementary figure 1). As no individual variable appeared to have a confounding effect, the fully adjusted model controlled for a priori defined variables gestational age, SGA, Apgar score, maternal age, and maternal psychiatric history.*' |
| Orban *et al.* 2016 | **Page 580:**  '*The adjustment sets were selected a priori based on a directed acyclic graph (see Supplemental Material, Figure S1) created with DAGitty. In model 1, we adjusted for age (continuous), sex, education (four categories), income (quartiles), economic activity (three categories), neighborhood-level SES (unemployment rate, continuous) and traffic proximity (continuous). In Model 2, we additionally adjusted for the potential confounders BMI (continuous) and smoking, and in Model 3, the potential confounders/ intermediates comorbidities (0, 1, or ≥ 2) and insomnia (yes/no) were added.*' |
| Osler *et al.* 2015 | **Page 6**:  '*The covariables included in the multiple regression models were selected based on the directed acyclic graph presented in Fig 1.*'  ***Table 3 (footer):***  '*aMatched on gender, age, and municipality on time of ACS; aaAdjusted for confounders: age and gender. aaa Adjusted for confounders: age and gender and mediating factors: somatic comorbidity, previous and incident depression*' |
| Oswald *et al.* 2017 | **Page 251:**  '*Potential confounders were identified, from the community-level, household and individual covariates recorded in the surveys (Table 1), based on the results of a literature review, an evaluation of directed acyclic graph… We used a sequential modelling approach to explore confounding –indicated by change in exposure estimates – and changes in residual variance.*' |
| Oswald *et al.* 2017A | **Page 4**:  '*An evaluation of directed acyclic graphs (DAGs) identified the same minimal sufficient set of covariates to estimate associations of community sanitation usage with each STH infection. A sequential modeling approach, removing covariates at each level from fully-adjusted models, was also used to identify confounders based on changes in exposure estimates. All models controlled for survey round to account for year and possible differences. Results are presented from crude, DAG-based, and fully-adjusted models for comparison.*'  **Table 2 (footer)**:  '*aCrude model only controlled for survey round bDAG-based model was adjusted for elevation; population density; community mean total of wealth indicators per household; soil moisture; and survey round cFull model for A. lumbricoides and T. trichuris adjusted for age; sex; anthelmintic treatment; bathing water source < 30 min; improved drinking water source; household owns: radio, television, mobile phone, iron roof, and has access to electricity; household education; elevation; soil moisture; community mean total of wealth indicators per household; population density; and survey round. Full model for hookworm adjusted for the same covariates in addition to shoe wearing.*' |
| Pattloch *et al.* 2017 | **Page 214:**  '*Unter verschiedenen Ansätzen des „machine learning*' *wählten wir daher das „model-based boosting*'*, das bislang vorwiegend in der Auswertung hochdimensionaler Daten, wie z. B. genomweiten Assoziationsstudien, eingesetzt wird. Unter mehreren Modellklassen wurde zur Untersuchung der Therapieentscheidung eine binär-abhängige Variable ausgewählt. Dadurch können Kovariableneffekte nach Transformation analog zur multiplen logistischen Regression als Odds Ratios (OR) interpretiert werden. Wir übersetzen Odds mit „Chance*' *im neutralen statistischen Sinn. Für technische Details von Boosting-Algorithmen verweisen wir auf. In Kürze dargestellt: Die Variablenselektion erfolgt bei diesem Ansatz implizit. Beginnend mit einem Nullmodell (d. h. ohne Kovariablen), werden sukzessive nur solche Kovariablen hinzugefügt, die maximal zu einer Modellverbesserung beitragen. Anhand der Reihenfolge der Kovariablenaufnahme ist eine Beurteilung der Relevanz der einzelnen Einflussfaktoren möglich. Sobald keine Modellverbesserung mehr erreicht wird, stoppt der Algorithmus. Das Stopp-Kriterium der Boosting-Iterationen wurde in dieser Untersuchung mit der Funktion cvrisk aus dem Programmpaket mboost bestimmt. Um die Variablenselektion gegenüber Stichprobenvariationen zu stabilisieren und damit die Generalisierbarkeit der Ergebnisse zu erhöhen, wurde das Boosting- Verfahren mit einem Resampling- Verfahren („stability selection*'*) kombiniert.*'  *[We therefore chose* '*model-based boosting*'*, which has so far been used primarily in the evaluation of high-dimensional data, such as genome-wide association studies, among various approaches of* '*machine learning* '*. Among several model classes, a binary was used to examine the therapy decision -dependent variable selected. As a result, covariable effects after transformation can be interpreted as odds ratios (OR) analogous to multiple logistic regression. We translate odds with* '*chance*' *in a neutral statistical sense. For technical details of boosting algorithms, we refer to. Briefly presented: The variable selection is implicit with this approach. Starting with a null model (i.e. without covariables), only those covariates are added that gradually contribute to a model improvement. On the basis of the order of the covariant recording, an assessment of the relevance of the individual influencing factors is possible. As soon as no model improvement is achieved, the algorithm stops. The stop criterion of the boosting iterations was determined in this study using the cvrisk function from the mboost program package. In order to stabilize the variable selection against sample variations and thus increase the generalizability of the results, the boosting method was combined with a resampling method (*'*stability selection*'*).]*  **Page 215:**  '*Die notwendigen Adjustierung Variablen wurden mit gerichteten azyklischen Graphen ausgewählt, unterstützt durch das webbasierte Hilfsmittel DAGitty*' *[The necessary adjustment variables were selected with directed acyclic graphs, supported by the web-based tool DAGitty]* |
| Paulson *et al.* 2006 | **Page 961:**  '*A causal model (Fig. 1), based on previous research and expert knowledge, identified complex hypothesized interactions among potential confounders. Selection of confounders for each exposure of interest was based on directed acyclic graphs (DAGs) established from the causal model*'  ***Table 4 (footer):***  '*†Adjusted for age and gender. ‡Adjusted for gender and state. §Adjusted for age and state. Adjusted for age, gender and state. ¶Adjusted for age, gender, state, and education. aaAdjusted for age, gender, marital status, education, and state. ††Adjusted for education, marital status, prior injury, age, and state.*' |
| Phillips *et al.* 2017 | **Page 2072:**  '*To avoid bias by adding covariates to the model that are intermediate factors or common effects, directed acyclic graphs (DAGs) were constructed to identify confounding factors in the association of the exposure (sarcopenia) and the outcome (disability). DAGs allow to identify a minimal adjustment set that contains the smallest set of covariates needed to estimate the effect of sarcopenia on disability without confounding. Unadjusted and adjusted models were fitted. Analysis was carried out using R version 3.0.3. DAGs were constructed using DAGitty, a browser-based environment for creating, editing, and analyzing causal models (directed acyclic graphs). The full DAG model with references and a more detailed explanation can be seen in the Online Resource Fig. 2.*'  **Electronic supplementary material:**  '*A DAG is a theoretical visualization of a whole causal network that links exposure and outcome. It consists of nodes representing variables (e.g., sarcopenia, disability, age, sex, BMI, etc.) and arrows representing causal associations between these variables. In a situation of complex causal associations of variables, a DAG can be constructed based on previous knowledge, e.g. from literature or from experts. The graph can then be analyzed to determine the minimally sufficient adjustment set using predefined rules. The resulting minimally sufficient adjustment set (age, sex, SES, BMI, diabetes, physical activity, heart diseases, malnutrition, neurological diseases, stroke, and cognitive status) was then entered into a linear mixed effects regression model to determine the association of sarcopenia and covariates with disability scores.*' |
| Pink *et al.* 2015 | **Page 991:**  '*In order to minimize bias in con- founder selection, distinct directed acyclic graphs (DAGs) were con- structed. DAGs represent a new graphical and analytical approach to explore causal structures, identify confounders and determine minimal sufficient adjustment sets. DAG construction and analysis was performed using DAGitty. Accordingly, age, gender, education, smoking status, abdominal obesity, alcohol consumption, physical activity, diabetes and dyslipidaemia were considered as covariates in fully adjusted models.*' |
| Preußel *et al.* 2015 | **Page 4:**  '*Because analytical adjustment for potential confounding variables can create bias where none exist, we deduced the minimally sufficient adjustment set to minimize bias using the graphical interface DAGitty. Second, we then employed a manual forward stepwise selection strategy with the forced-in variables of the minimally sufficient adjustment set and food-items as candidate variables that had a p-value <0.05 in univariable analysis. Variables for points of food purchase and supply were not considered for multivariable modelling because of too many missing values. We set the p-value for candidate variables eligible for multivariable modeling narrowly to p<0.05 to reduce the risk of increasing bias and model non-convergence due to too few outcome events per explanatory variable*' |
| Protudjer *et al.* 2015 | **Page 476:**  '*Potential confounding variables were considered using directed acyclic graphs (DAG). Using DAGs generated via DAGitty, two potential confounders were identified for the association between asthma and pubertal staging (Fig. S1): actual age at the 12-year questionnaire, given that age influences pubertal staging and asthma, and BMI z-score at 8 years, given the association between high BMI and pubertal debut and obesity and asthma by the 12-year questionnaire. Similarly, these potential confounders were found for the association between asthma and height, with an additional confounder of socioeconomic status, based on parent-reported maternal and paternal highest level of education (Fig. S2).*' |
| Pyko *et al.* 2015 | **Page 595**:  '*Potential confounders were identified based on a literature search and by development of a directed acyclic graph (DAG). Besides nodes of road traffic, railway and aircraft noise, the DAG included age, gender, physical activity, dietary habits, alcohol consumption, psychological distress, occupational status, job strain, shift work, educational level, marital status, individual income level, smoking status, sleep disturbance, noise sensitivity, road traffic noise annoyance and road traffic air pollution as well as contextual socioeconomic status (see eFigure 1). We considered age and gender as the main adjustment variables which were included in all models. Information on the remaining variables was obtained either directly from single questions in the survey questionnaire, or combined from several questions (quality of diet, psychological distress and job strain). Furthermore, information on household mean income in small geographical units with an average population of 1000–2000 individuals was obtained from registers held by Statistics Sweden and used to account for potential contextual confounding by socioeconomy.*'  **Page 596**:  '*The dagitty.net software was used to determine a minimal sufficient adjustment set of covariates for estimating the possible total effect of noise on markers of obesity. Finally, the fully adjusted model included age (continuous), gender, diet (indices of recommended and non-recommended food scores), alcohol consumption (daily, weekly, seldom, never), education (primary school, upper secondary school, university education), physical activity during leisure time (sedentary: regular exercise less than 2 h/ week, moderate: regular exercise at least 2 h/week, regular: regular exercise at least 30 min 1–2 times per week, frequent regular: at least 30 min 3 times or more per week), smoking status (never, former, current), psychological distress (yes/no) and job strain (yes/no), shift work (yes/no), and noise sensitivity (less sensitive than others, equally sensitive as others, more sensitive than others). In sensitivity analyses, we investigated how the association between road traffic noise and obesity markers was affected by adjustment for contextual confounding, other sources of noise as well as air pollution from local road traffic. The analyses of contextual confounding were performed based on mean income in 138 small areas considered homogeneous with respect to socioeconomic characteristics.*' |
| Pyko *et al.* 2017 | **Page 117005-3:**  '*The covariates evaluated as confounders were identified based on a literature search and by development of a directed acyclic graph (DAG) with DAGitty.net software (see Figure S2). We used the DAG to select a set of confounders for assessment of the direct effect of transportation noise on the development of obesity.*'  **Page 117005-3:**  '*First, a crude model is used with adjustment for only sex and age (35/40/45/50/55 y of age). Second, a fully adjusted model is presented with additional adjustment for dietary habits, physical activity during leisure time, alcohol consumption, education level, physical activity, smoking status, psychological distress, job strain, and shift work.*' |
| Rajappan *et al.* 2017 | **Page 1293:**  '*Potential confounders were included in a directed acyclic graph (DAG) to identify the most appropriate adjustment set (Figure S1A); this set comprised maternal age, education, parity, and smoking in pregnancy. To account for possible unmeasured genetic, environmental, and lifestyle factors shared within families which might confound the relationship between maternal BMI and infant infections, maternally reported paternal BMI was included in a second model as a descendant of unmeasured confounders (Figure S1B).*'  **Page 1293:**  '*To assess the direct effect of maternal BMI we considered a further model adjusting for potential postnatal mediators of the effects of maternal obesity. In this model we adjusted for birthweight and adiposity gain in early infancy in addition to the adjustment set identified by the DAG and paternal BMI.*' |
| Rancière *et al.* 2017 | **Page 739:**  '*Covariates were selected for inclusion in the statistical models using a directed acyclic graph (DAG) built using DAGitty version 2.2. The DAG is presented in Figure S1. Relationships between each of the variables were assigned based on knowledge of the literature regarding these associations. Given the assumptions described in the DAG theory, we identified the minimal sufficient set of adjustment variables for estimating the direct effect of TRAP exposure on respiratory health. Covariates selected for inclusion in the multivariable models were sex, birth weight (continuous), family SES (low, medium, high), maternal education (high school education or less, at least some college), exclusive breastfeeding during the first 3 months (no, yes), type of day care during the first 6 months (no day care, at home, at a childminder’s home, in a day care center), maternal smoking during pregnancy (no, yes), exposure to environmental tobacco smoke at home during the first year (no, yes), body mass index ≥ 85th percentile for age and sex at 2–3 years (no, yes), visible mold in the home at birth (no, yes), gas for cooking/heating in the home at birth (no, yes), and stressful family events (no, yes). Given our research question, models were also adjusted for maternal and paternal history of allergy (no, yes), which did not result in any biasing path.*' |
| Ratanawongsa *et al.* 2013 | **Page 212**:  '*Our modeling was guided by a directed acyclic graph, which depicts causal relationships between measured variables in the analysis (Figure 2). Directed acyclic graphs help avoid errors caused by confounding, blocking (adjustment for a variable on a causal pathway between exposure and outcome), and colliding (adjusting for variables affected by both exposure and outcome, leading to spurious associations). We reviewed existing literature and theory about causal relationships and temporal ordering among patient, health care provider, relationship, and system variables that could affect the relationship between communication and medication refill adherence. We used established rules for determining the necessary covariates to estimate the direct effect of communication on medication refill adherence (Figure 2). A sensitivity analysis including number of medications for chronic conditions did not affect the point estimates for our analyses, suggesting that this variable’s exclusion based on the directed acyclic graph was correct.*'  **Table 3 (footer):**  '*b Adjusted for age, sex, race/ethnicity, educational attainment, English language proficiency, functional health literacy, income, prescription and outpatient copayment requirements, depression, Charlson comorbidity index, external locus of control, and conscientiousness.*' |
| Rebelo *et al.* 2016 | **Page 173**:  '*A directed acyclic graph (DAG) was constructed using the DAGitty online software. The purpose of the DAG is to improve our understanding of the association between the exposure and outcome and all possible confounding factors. This approach allowed us to identify the minimal sufficient adjustment set to estimate the direct effect of the plasma adiponectin levels on the symptoms of depression, which included: BMI, education, marital status, desire to become pregnant, physical activity, sleep duration, social support and time elapsed after conception (Figure S1 – Supplemental Digital Content).*' |
| Rebelo *et al.* 2016A | **Page 5**:  '*A Direct Acyclic Graph (DAG), a type of causal diagram, was constructed using the DAGitty program in order to improve our understanding of the correlation between exposure and outcome and all possible confounding factors. According to this program, the minimal sufficient adjustment set for estimating the direct effect of mode of delivery on plasma adiponectin included birthweight, BP, gestational weight gain, gestational age at delivery and BMI (see S1 Fig, Supporting Information).*'  '*An additional sensitivity analysis was performed: variables with different distributions between groups of exposure (p < 0.20, according to results of Table 1) were included in the model one by one. However, none of them changed the magnitude of the effect, the direction of the association, or the level of significance. Consequently, they were not kept in the final model.* ' |
| Rêgo *et al.* 2016 | **Page 3**:  '*A directed acyclic graph (DAG) was con- structed to identify a minimum set of confounding adjustment in the DAGitty program (Figure 1). Interrelations between variables were constructed based on Takito & Benicio’s theoretical hierarchical model. Models were then adjusted for maternal schooling, economic class, living with partner, and living with children.*' |
| Reiner *et al.* 2016 | ***Page 159:***  '*An a priori causal model or directed acyclic graph (DAG) (Fig. 1), depicting potential causal associations between the large machinery-related injury outcome and environmental and sociodemographic variables, was used to identify potential confounders for each exposure of interest in multivariate models and to exclude other variables that may introduce bias if included in the analyses. Some causal links shown in the DAG have been well established while others are hypothesized, based on expert investigator knowledge.*'  ***Table 5 (footer):***  '*yAdjusted for within-household correlation using GEEs and weighted for nonresponse. zAdjusted for age and state. §Adjusted for gender and state. jjAdjusted for gender and age. {Adjusted for gender, age, and state. #Adjusted for gender, age, state, educational status, and marital status. aaAdjusted for gender, age, state, educational status, marital status, and prior injury.*' |
| Rhea *et al.* 2014 | **Page 198:**  '*For each risk factor, a directed acyclic graph (DAG) was used to evaluate potential confounding by covariates, including other risk factors, and create a minimally sufficient covariate set. Directed acyclic graph analysis indicated that for each risk factor, the model should be adjusted for all other risk factors and for patient age.*' |
| Ribeiro *et al.* 2017 | **Page 535**:  '*According to heuristic rules applied to the DAG, to estimate the total effect of between being overweight or obese and having ECC, adjustments for all variables presented were necessary (mother’s education level, eco- nomic class, birthweight, nutritional status at twelve months, and frequency of added sugar consumption). Child age was also included in model adjustment.*' |
| Risch *et al.* 2014 | **Page 95**:  '*A six-step directed acyclic graph (DAG) approach was used for covariate selection to reduce the potential for and degree of bias in effect measure estimation in the final chosen adjusted statistical models. With the increased complexity of hypothesized relationships among sibling characteristics, CM outcomes and potential confounding variables, consideration of association based on a priori expert knowledge is necessary to avoid adjusting for variables that may increase the risk of introducing bias where none existed. The DAG approach allows for the assessment and incorporation of such knowledge and/or assumptions in the selection of a minimally sufficient adjustment set of covariates needed to estimate an unbiased effect of neonatal complications on CM. Informed by the DAG covariate selection process and existing literature, race and marital status were not considered as potential confounding variables and, thus, not included in the final models. Selected covariates were included in our final analysis models to also attenuate or eliminate any bias that could potentially be introduced in the person time (time to CM event) available for analysis.*'  **Table 2 (footer):**  '*Adjusted for a child’s age, sibling status (NICU infant vs. Sibling), number of living children, Medicaid enrollment, and maternal age. bAdjusted for a child’s age, sibling status (NICU infant vs. Sibling), number of living children, Medicaid enrollment, and prior child protective services (CPS) involvement. cAdjusted for sibling status (NICU infant vs. sibling), number of living children, Medicaid enrollment, maternal age, and prior CPS involvement.*' |
| Rusconi *et al.* 2011 | **Page 663 (introduction):**  '*We also try to explore the relationship between paracetamol and antibiotics administration and wheezing by using causal diagrams [directed acyclic graphs (DAG)], a powerful tool to help answer causal queries.*'  **Page 664 (results):**  '*In Figure 1, the DAG summarizes possible relationships between the variables, taking into account what is known on this topic. The (confounded) association between paracetamol/antibiotics and wheeze in the first 2 years of life (persistent wheezing) through infection and fever is shown in the DAG by the arrows from ‘viral infection/fever’ to ‘wheezing P’, and to ‘paracetamol/antibiotics’. According to causal theory, we need not postulate a direct arrow (representing a causal effect) from paracetamol/antibiotics in the first year to outcomes at the age of 6–7 years, to explain an association, if there are ‘open’ paths between paracetamol/ antibiotics and outcome. In other words, if there are non-causal (biasing) paths open, a non-null association will be found independently of the true effect of paracetamol/antibiotics. The DAG shows that such paths exist: [backdoor path ‘paracetamol/antibiotics-infection-wheezing’ at school age]. To block this path and to obtain unconfounded estimates of the association between paracetamol/ antibiotics and wheeze at school age (if any), we should condition on (stratify for or adjust for) infection*' |
| Rutegård *et al.* 2016 | **Page 580**:  '*Based on previous research, causal diagrams and directed acyclic graphs were constructed to identify a minimally sufficient adjustment set of confounders. For the outcome anastomotic leakage, the included confounders were sex (male, female), ASA class (I–II, III–IV), preoperative radiotherapy (no, yes), and intraoperative adverse events (no, yes). For the outcome permanent stoma, adjustment was made for age (<65, 65–75, and >75), ASA class, radiotherapy, and tumor stage (I, II, III, IV). Since diverting stoma reduces the risk of anastomotic leakage, this variable was not considered a confounder, but rather a mediator of the effect of diverting stoma on the risk of a permanent stoma.*' |
| Rutegård *et al.* 2016A | **Page 130**:  '*A minimally sufficient adjustment set of covariates was determined from a causal diagram, using directed acyclic graphs. These included ASA class (I–II, or III), tumour height (0–6, 7–12, and 13–15 cm), tumour stage (I, II, III, or IV), preoperative radiotherapy (yes, no), preoperative chemotherapy (yes, no), perioperative bleed (≤400 or >400 ml), and diverting stoma (yes, no). Tumour height was divided into clinically meaningful categories, emulating anastomotic height and type of mesorectal excision; perioperative bleed was categorised using the median as cut-off. Using this adjustment set, the variables sex and age were redundant and were therefore not included in the regression mod- els; however, these covariates were included in a sensitivity analysis. All analyses were adjusted for clustering of patients within hospitals, taking into account that these patients do not constitute independent observations, therefore making the confidence intervals more conservative.*' |
| Sage *et al.* 2010 | **Page 3:**  '*Directed acyclic graphs (DAGs) were developed for each of the exposures of interest to facilitate selection of potential confounding variables for control*'  **Table 4 (footer):**  '*a Model adjusted for: school type and location; number of students enrolled; grade levels taught at the school; student race; % students eligible for free/reduced price lunch; % special education students; % limited English proficiency; average property tax per home in the school district; adjusted for with-in correlations using GEEs; and, weighted for non-response and unknown eligibility. b Model adjusted for: covariates listed above; and, all other categorical percentage expenditure allocation covariates.*' |
| Salti *et al.* 2017 | **Page 366:**  '*Second, distinct directed acyclic graphs (DAGs) were constructed. DAGs are a graphical and analytical approach to explore causal structures. They are based on prior knowledge about causal relations among variables of interest and are able to identify confounders and determine minimal sufficient adjustment sets. DAG construction and analysis was performed, using DAGitty. The main DAG, based on which relevant confounders were selected for main analyses in this study, is presented in Figure S1. Both methods resulted in the same adjustment set. Accordingly, age and gender were considered as covariates in fully adjusted models (Tables 2 and 3).* ' |
| Samson *et al.* 2016 | **Page 548:**  '*To reduce common errors made in confounder selection, the analytic approach was based on a causal diagram. The causal diagram in Fig. 1 shows the variables as vertices connected by directed edges or arrows. There are no directed cycles in the figure. These properties are characteristic of a directed acyclic graph (DAG). There is a causal pathway between the exposure (diabetes) and outcome of interest (BrCA stage), which represents a direct effect of T2DM on BrCA stage-at-diagnosis. In observational studies, a common approach to estimating direct effects yields biased information. Using DAGs, researchers can identify potential confounders and improved understanding of bias that may have occurred. Race is an effect modifier and was examined by stratifying analyses by race, i.e., AA and EA women considered separately.*'  **Page 548:**  **'***Nonmodifiable risk factors were fit in the model to reduce the effect of confounding.*' |
| Schipf *et al.* 2011 | **Page 906:**  '*Zur Konzeptualisierung von Confounding wurde ein gerichteter azyklischer Graph (Directed Acyclic Graph, DAG) erstellt (Abb. 1), um die kausalen Strukturen zwischen Testosteron, der Exposition von Interesse (E), und einem inzidenten Typ-2-Diabetes mellitus, dem Outcome (D), unter Berücksichtigung von weiteren Kovariaten (C 1 –C n ) als potenzielle Confounder zu visualisieren und ein minimally sufficient adjustment set (MSAS) abzuleiten.*' *[To conceptualize confounding, a directed acyclic graph (DAG) was created (Fig. 1) to show the causal structures between testosterone, the exposure of interest (E), and an incident type 2 diabetes mellitus, the Outcome (D), visualizing further covariates (C 1 –C n) as potential confounders and deriving a minimally sufficient adjustment set (MSAS).]*  **Page 907:**  '*Finden des minimally sufficient adjustment sets (MSAS) (bevorzugt mit gemessenen Kovariaten): Es konnten 2 MSAS mit folgenden Kovariaten bestimmt werden: 1. Set: Taillenumfang, Alter, Rauchstatus und SHBG; 2. Set: Taillenumfang, körperliche Aktivität, Rauchstatus und SHBG.*' *[Finding the minimally sufficient adjustment set (MSAS) (preferably with measured covariates): 2 MSAS with the following covariates could be determined: 1st set: waist circumference, age, smoking status and SHBG; 2nd set: waist circumference, physical activity, smoking status and SHBG.]* |
| Schliep *et al.* 2011 | **Page 390:**  '*We used the DAGitty program to determine the minimal sufficient adjustment set. Individual assessments of male or female BMI on outcomes were adjusted for male and female age, parity, and partner BMI, whereas for analyses considering the association of couple BMI categories and pregnancy/live birth, we only adjusted for male and female age and parity because partner BMI was incorporated into the couple BMI category. Our male BMI models did not stratify or adjust by fertilization method (conventional insemination vs. ICSI) or by infertility diagnosis (male factor) because we considered these factors to be along the causal pathway from BMI to pregnancy/live birth outcome. For our female BMI models, we did conduct a sensitivity analysis additionally adjusting for reason for infertility treatment (endometriosis, ovulation disorders, diminished ovarian reserve, tubal disease, uterine disorders, or unexplained infertility) because although these factors are also most likely on the causal pathway between BMI and pregnancy success, it is possible that they are additionally or alternatively common causes of both BMI and pregnancy success, and thus potential confounders. An additional sensitivity analysis was conducted on all models additionally adjusting for male and female alcohol (yes/no), daily exercise (yes/no), caffeine (caffeinated beverages per day), and smoking (yes/no) for the random subsample (n = 70) containing lifestyle information.*' |
| Schmidt *et al.* 2013 | **Page 1433:**  '*All analyses were stratified by study center and adjusted for covariables selected on the basis of the theory of directed acyclic graphs, i.e. age at diagnosis and the established prognostic factors: tumor size (<2 cm, 2–5 cm, ≥5 cm, growth into chest wall, neoadjuvant chemotherapy treated carcinoma, in situ carcinoma), nodal status (0, 1–3, 4–9, ≥10 affected lymph nodes, neoadjuvant chemotherapy treated carcinoma, in situ carcinoma), histological grade (1+2, 3+4, in situ carcinoma, neoadjuvant chemotherapy treated carcinoma), ER/PR status (in situ carcinoma, ER1/ PR1, ER1/PR2 or ER2/PR1, ER2/PR2, neoadjuvant chemotherapy-treated carcinoma), radiotherapy (yes/no), breast cancer detection type (physician-detected by clinical examination/mammography/ultrasound, self-detected by palpation/ secretion/pain), use of menopausal hormone therapy at diagnosis (current, never/past), pre-diagnosis BMI (<18.5, 18.5 to <25, 25 to <30, ≥30 kg/m2), smoking status at diagnosis (current, ex, never) and packyears, as well as pre-existing angina pectoris. Models for overall mortality and for other deaths were adjusted in addition for pre-existing hypertension (yes/no), previous stroke (yes/no) and use of insulin (yes/no). Other adjustment sets considering the following covariates were also investigated, but did not alter the HRs for the PA variables by more than 10%: type of surgery (mastectomy, breast conserving), chemotherapy (yes/no), hormone therapy (yes/no), HER2-neu status, postmenopausal at diagnosis (yes/no), marital status (married, single, separated, divorced, widowed), alcohol use at diagnosis (0, >0 to <19, ≥19 g/day), previous myocardial infarction (yes/no), peripheral arterial obstructive disease (yes/no), osteoporosis (yes/ no), rheumatoid arthritis (yes/no), venous thrombosis (yes/ no), previous pulmonary embolism (yes/no), chronic lung disease (yes/no), chronic liver (yes/no), gastric or renal diseases (yes/no), psychological disorders (yes/no), migraine (yes/no), thyroid disorders (yes/no), previous myoms (yes/ no) or ovarian cysts (yes/no).*'  **Table 2 (footer):**  '*1All models were adjusted for tumor size, nodal status, tumor grading, ER/PR status, radiotherapy, screening-detected tumor, HT use at diagnosis, age at diagnosis, BMI pre-diagnosis, smoking status and packyears and pre-existing angina pectoris. In addition, models for overall mortality and for other deaths were adjusted for pre-existing hypertension, previous stroke and use of insulin*' |
| Schwahn *et al.* 2013 | **Page 1431:**  '*Many risk factors have been suggested to confound the relationship between the number of teeth and mortality (eFig. 1). The most clinically important confounder for the relationship between unreplaced teeth and mortality is the number of remaining teeth. If the number of remaining teeth is included in the model, additional adjustment for risk factors for tooth loss may lead to over-adjustment. To reduce a high number of confounders appropriately, causal diagrams are recommended. In many settings, causal diagrams based on directed acyclic graphs (also known as DAGs) are superior to conventional methods such as the change-in-estimate procedure and could lead to different confounder sets. Using the number of unreplaced teeth as the exposure, few confounders remained in the following minimal adjustment set: number of teeth, age, sex, social determinants (education, income, marital status, and partnership), and oral health behaviour. In a series of causal diagrams, the inclusion of the number of remaining teeth suspended other potential confounders, such as smoking status. For causal diagrams of exposures other than the number of unreplaced teeth, please, see the Online-Only Material of this work and those of a related review.*' |
| Sehrndt *et al.* 2011 | **Page 903:**  '*Zur Veranschaulichung der kausalen Zusammenhänge und zur Ermittlung eines minimalen und hinreichenden Sets von Adjustierungsvariablen, für die in den statistischen Analysen adjustiert werden sollte, wurde die Methode der kausalen Diagramme oder auch Directed Acyclic Graphs (DAGs) angewandt.*' *[The method of causal diagrams or Directed Acyclic Graphs (DAGs) was used to illustrate the causal relationships and to determine a minimal and sufficient set of adjustment variables for which adjustments should be made in the statistical analyses]*  **Page 903:**  '*Nach Auswertung der DAGs und Bestimmung der minimally sufficient adjustment sets (MSAS) mit dem DAG program Version 0.2.1 erfolgten mehrere multiple lineare Regressionsanalysen mit der abhängigen Variablen Lebensqualität.*' *[After evaluating the DAGs and determining the minimally sufficient adjustment sets (MSAS) with the DAG program version 0.2.1, we performed several multiple linear regression analyzes with the dependent variable quality of life]*  **Page 903:**  '*Dementsprechend enthalten die MSAS jeweils einzig die Variable Geschlecht. Die Auswertung des präoperativen DAGs ergab 947 Backdoor-Pfade, wovon 31 nicht blockiert waren. Pfadabhängige Collider waren Depression, Angst, soziale Unterstützung, Schweregrad der koronaren Herzkrankheit (KHK) and Begleiterkrankungen. Die Auswertung des DAGs der Δ Lebensqualität ergab 1979 Backdoor-Pfade, wovon 35 nicht blockiert waren. Pfadabhängige Collider waren Δ Depression, Δ Angst, soziale Unterstützung postoperativ, postoperative Komplikationen, postoperative Dyspnoe und Schweregrad der KHK.*' *[Accordingly, the MSAS contained only the variable gender. The evaluation of the preoperative DAG resulted in 947 backdoor paths, of which 31 were not blocked. Path-dependent colliders were depression, anxiety, social support, severity of coronary heart disease (CHD) and comorbidities. The evaluation of the DAG's Δ quality of life revealed backdoor paths, 35 of which were not blocked, in 1979. Path-dependent colliders were Δ depression, Δ anxiety, social support postoperative, postoperative complications, postoperative dyspnea and severity of CHD.]* |
| Senkomago *et al.* 2015 | **Page 1431:**  '*To reduce a high number of confounders appropriately, causal diagrams are recommended. In many settings, causal diagrams based on directed acyclic graphs (also known as DAGs) are superior to conventional methods such as the change-in-estimate procedure and could lead to different confounder sets. Using the number of unreplaced teeth as the exposure, few confounders remained in the following minimal adjustment set: number of teeth, age, sex, social determinants (education, income, marital status, and partnership), and oral health behaviour. In a series of causal diagrams, the inclusion of the number of remaining teeth suspended other potential confounders, such as smoking status. For causal diagrams of exposures other than the number of unreplaced teeth, please, see the Online-Only Material of this work and those of a related review.*' |
| Senkomago *et al.* 2016 | ***Page 574:***  '*Potential confounders were identified from the literature and analyzed using a directed acyclic graph. All models were adjusted for educational attainment and bathing frequency at baseline; HPV infection type (incident or prevalent infection), HIV infection status and infection with multiple HPV types at detection of HPV16/HPV18; and condom use, number of sex partners, and infection with other sexually transmitted infection (STIs) in the previous 6 months*'  **Page 574:**  '*Additional analyses restricted to high viral load infections (with quantifiable viral load values >250 copies/scrape) were performed to examine the association between the natural log HPV viral load at detection of HPV16 or HPV18 infection, and the rate of HPV clearance after 6 months at each site. Sensitivity analyses were conducted restricting analyses to β-globin–positive results*' |
| Seward *et al.* 2015 | **Page 6:**  '*Directed acyclic graphs (DAGs) were used to inform the statistical modelling of the relationships between each of the separate clean delivery practices, maternal mortality and potential confounders to ensure that the confounders selected were appropriate. The DAGs supported the appropriateness of all selected confounders for inclusion in the models. Details of confounder selection can be found in S1 Text*'  **Table 2 (footer):**  '*b Adjusted for maternal age, maternal education, parity, number of antenatal care visits, household assets, and for the pooled analysis, study site*'  **S1 Text:**  '*Directed acyclic graphs (DAGs) were used to model the associations between selected confounders with each other, with the individual clean delivery practices (exposures), and with the outcome of post-natal maternal death. These DAGs then informed the statistical modelling of the relationship between each of the separate clean delivery practices and maternal mortality, taking confounders into account.1 In order to better approximate the causal relationships, the DAGs were modelled in relation to the pregnancy timeline from the pre-conception period to the post-natal period. Figure 1 shows the relationship between handwashing and post-partum maternal death and shows the appropriateness of all confounders. Figure 2 shows the relationship between using a clean delivery kit and post-partum maternal death and, contrary to Figure 1 that illustrates the inappropriateness of including individual clean delivery practices as potential confounders.*' |
| Shah *et al.* 2015 | **Page 80:**  '*We used a directed acyclic graph (DAG) to summarize our prior understanding of the causal relationships between exposures of interest and prediabetes and diabetes. We then analyzed the DAG using an online tool to identify a minimal sufficient adjustment set (MSAS) of confounders for each exposure of interest. Under the causal assumptions encoded in the DAG and provided that the regression model for the outcome is correctly specified, adjusting for an MSAS is sufficient to obtain an unconfounded estimate of the overall effect of each exposure of interest on the outcome, without adjusting away indirect effects via mediators. Predictors included a traditional cultural beliefs scale (the base question was* '*How much would you wish these traditions from South Asia would be practiced in America?*' *and the seven items included: performing religious ceremonies; serving sweets at ceremonies; fasting on specific occasions; living in a joint family; having an arranged marriage; eating a staple diet of chapatis, rice, dal, vegetable, and yogurt; using spices for health and healing), socioeconomic status (SES; education and family income), fasting and dietary pattern, chronic burden and psychological disorders (depression and anxiety), sedentary behavior (time spent watching television) and physical activity (total exercise in metabolic equivalent-min/wk). Multinomial logistic regression was used to estimate the causal effects of each exposure of interest on pre-diabetes and type II diabetes, adjusting for the exposure-specific MSAS detailed in the Appendix. All models were further adjusted for study site, a strong independent correlate of these outcomes, and a potential marker for unmeasured confounding (Appendix).*' |
| Shaw *et al.* 2018 | **Page 83:**  '*We used the theoretical framework of pathways between fuel price and health (Fig. 1) to inform a causal diagram for the analysis (Appendix A). The causal diagram guided the choice of covariates relevant on the weekly timescale of the analysis. Public holidays were included in the model as potential confounders (in causal diagram terminology, to block backdoor pathways between exposure and outcome via car use and public transport). Weather variables were not included in the main analysis as the causal diagram indicated they would not act as confounders (in causal diagram terminology, the pathways were already blocked by public transport and car use acting as colliders).*' |
| Sheikh *et al.* 2014 | **Page 3:**  '*The identification of confounders was based on a priori knowledge of the association between the variables under study. The diagram is illustrated in Figure 1, to distinguish: i) exposure-outcome confounders (variables that potentially confound the association between CSES and health and wellbeing in adulthood); ii) exposure-mediator confounders (variables that potentially confound the association between CSES and respondents’ education), and; iii) mediator-outcome confounders (variables that potentially confound the association between respondents’ education and health and wellbeing in adulthood). Age was considered a potential exposure-outcome confounder, mediator-outcome confounder, as well as an exposure-mediator confounder in all analyses. When childhood financial conditions was used as an exposure, fathers’ education, mothers’ education and spouse’s education were included in the model as potential mediator-outcome confounders. When mothers’ and fathers’ education were used as an exposure, childhood financial conditions and spouse’s education were included in the model as potential mediator-outcome confounders. Similarly, mothers’ education was included in the models when fathers’ education was used as an exposure, and fathers’ education was included in the model when mothers’ education was used as an exposure.*' |
| Shoaff *et al.* 2016 | **Page 53:**  **'***A directed acyclic graph (DAG) was drawn a priori to assess potential confounders associated with both phthalate exposure and birth outcomes (Supplemental Fig.1). We considered socio-demographic, nutritional, environmental, and perinatal factors.*'  ***Page 53:***  **'***Our final adjusted models included maternal race, age, income, education, marital status, insurance status, parity, cotinine, food security, BMI, prenatal vitamin use, fish consumption, fruit/vegetable consumption, and depressive symptoms. When assessing head circumference, mode of delivery was added to the model.*' |
| Shoaff *et al.* 2017 | **Page 097008-2:**  '*We used a directed acyclic graph to select covariates and considered maternal sociodemographic, perinatal, nutritional, environmental, and child factors (Figure S5). Our final adjusted models included maternal age at delivery, race, marital status, insurance, income, education, parity, cotinine, depressive symptoms, mid pregnancy BMI, food security, fruit/vegetable and fish consumption during pregnancy, prenatal vitamin use, child sex, and child age at the 8-y visit.*' |
| Skretteberg *et al.* 2013 | **Page 204:**  '*Significant variables in univariate analyses ( p < 0.05) were entered into multivariate analysis and a prediction model for diabetes was reached by stepwise backward elimination (Table 2 and Supplementary Table S2). In order to minimize potential bias from intermediate variables when specifically studying the association between FTG and diabetes, the Directed Acyclic Graph (DAG) approach was used to identify a suitable main final model for adjustments (Tables 3 and 4). The DAG method is designed to provide suitable models for assessing the causal effect of one variable upon another. Given a list of potential causal relations between the total available set of variables, the DAG approach selects a set of variables to be included in the model that minimize bias by retaining in the model potential confounders while eliminating intermediate variables. The main final model identified by DAGs included adjustment for age, FBG, family history of maternal diabetes mellitus and PF. To evaluate the impact of PF separately, we first adjusted for age, FBG and maternal diabetes (Model 1), and then further for physical fitness. In order to obtain results that could be comparable to previous studies of triglycerides (TG) and diabetes risk, we also performed additional adjustments for BMI.*' |
| Smallwood *et al.* 2017 | **Page 708:**  '*The selection of subjects and variables for statistical modelling was based on our Directed Acyclic Graph of hypothesised causal influences linking nitrate excretion and measured blood pressure (see Supplementary Appendix A).*'  '*Regression models were adjusted initially for age-group and gender, and then additionally for estimated daily potassium intake from a diet diary, high-sensitivity C-reactive protein, diabetes, current smoking, activity level in the last year, and highest educational attainment. Further adjustment was made for 24-hour urinary sodium excretion. Models were also adjusted for antihypertensive medication, with separate terms for ACE inhibitors, diuretics, beta-blockers, peripheral vasodilators, and other antihypertensives.*' |
| Sohn *et al.* 2015 | **Page 584:**  '*We used causal diagrams to select important covariates for inclusion in the logistic regression model; specifically, the following covariates were included: age, sex, race, geographic region, household income, the year of index date, health care utilization intensity, and medical history during the pre-index period.*' |
| Solmi *et al.* 2017 | **Page 1661:**  '*We employed causal diagrams, known as directed acyclic graphs (DAGs), to identify variables which could confound the association between cat ownership and psychotic symptoms. We modelled hypothesized associations between a broad initial set of potential child- and mother-based variables, cat ownership in either pregnancy or childhood and PEs using the DAGitty web based software. Our DAGs (Supplementary Figs S1 and S2) suggested that it was inappropriate to control for some of these variables, either because they did not meet criteria for confounding (e.g. child gender, stressful life events, maternal depression, pet ownership other than dogs), or because adjustment for other variables (e.g. paternal age, dog ownership in pregnancy) provided sufficient control for any other causal paths (e.g. maternal age, dog ownership at age 4 years). From our DAGs we were able to identify the minimal sufficient number of confounders of the relationship between exposure to cat ownership in pregnancy and childhood and PEs at ages 13 and 18 years. These included: child ethnicity (white/non-white – including Black African, Black Caribbean, Other Black, Indian, Pakistani, Bangladeshi, Chinese, Other, mixed); paternal age (at the time of mother’s pregnancy); maternal marital status in pregnancy (single, separated, divorced, or widowed/married); highest maternal academic education in pregnancy (vocational course/secondary schooling/university degree or higher); maternal social class (manual v. non-manual profession); number of house moves up to age 47 months (∼4 years); housing type (detached, semidetached semidetached, terraced/flat, other); household crowding index (range 0–1); and dog ownership in pregnancy.*'  **Page 1661:**  '*We then fitted univariable and multivariable logistic regression models adjusting for all variables identified as relevant confounders using DAGs (Supplementary Figs S1 and S2), i.e.: dog ownership in pregnancy; housing type; household crowding; maternal education, social class, and marital status; paternal age; number of house moves. When the studied exposure was cat ownership in childhood, we further adjusted for maternal cat ownership in pregnancy*' |
| Spillane *et al.* 2013 | **Page 1366:**  '*Prior knowledge, literature review, and causal diagrams were used to identify potential covariates from among the available patient, tumor, and treatment variables for inclusion in the multivariate model of colorectal cancer–specific survival (29, 30). Cancer treatment variables were included as time-varying covariates. The final multivariate model was selected using backward elimination on the basis of a maximum cumulative change in the risk estimates of 10% (31, 32). Analyses were also conducted stratifying by metformin dosing intensity (low/high) and by receipt of metformin exclusively or in combination with no metformin ADDs. This process was repeated for overall (all-cause) survival. Finally, analyses were repeated as above in the full cohort, that is, with the inclusion of nondiabetic patients as the reference group in place of diabetic patients who did not receive metformin.*' |
| Spillane *et al.* 2014 | **Page 80:**  '*Prior knowledge, literature review and causal diagrams were used to identify potential covariates for inclusion in the multivariate model. The final multivariate model was then selected using backwards elimination based on a maximum cumulative change in the odds ratio of 10%. Analyses were also conducted stratifying by (i) metformin dosing intensity and (ii) receipt of metformin exclusively or in combination with non-metformin ADDs and (iii) combinations of dosing intensity and metformin/non-metformin ADDs. Finally, analyses were repeated as above comparing metformin-exposed diabetics with non-diabetics. The confounder selection process for these analyses was carried out in the same manner as that for the primary analyses.*' |
| Ssewanyana *et al.* 2015 | **Page 353:**  '*A DAG aided in identifying variables for adjustment, while estimating effect. DAGs are designed to deal with causal chains that may involve multiple interrelated variables and also to explain and assess the effect of commonly unstated simplifications in research. The use of DAGs has also been recommended to systematically address the possibility that selected covariates may potentially introduce conditional associations known as collider bias. The DAG of the possible exposure – outcome relationship is presented in Figure 1. We used the open-access software DAGitty to identify sufficient adjustment sets. Using DAGitty, a DAG explaining the probable exposure – outcome process was generated and so were minimum sufficient adjustment sets (MSAS) of covariates, which were adjusted for in the final effects model. A multiple logistic regression model was used to establish the effect in terms of the odds ratio (OR) of romantic relationship status on condom use during vaginal, anal and oral sex in the previous 30 days. Separate analytical models were conducted for each form of sexual activity. The variables that comprised the MSAS were adjusted for in each logistic regression model.*' |
| Starling *et al.* 2014 | **Page 107:**  '*Covariates were selected through the construction of a directed acyclic graph (DAG) representing the existing literature, and the identification of a minimally sufficient set of variables to control confounding. The DAG was primarily based on expected confounders of the PFAS–HDL association but was also generalizable to the other lipid outcomes. The minimally sufficient adjustment set was identified using DAGitty v1.0 (www.dagitty.net). All covariates were modeled as categorical variables to allow for non-linear associations. The categorization of covariates was as follows: maternal age (b24, 25–29, 30–34, and ≥35 years); pre-pregnancy BMI (b25, 25–29.99, and ≥30 kg/m2); maternal education (less than high school, completed high school, some college, 4 or more years of college); smoking at mid-pregnancy (yes/no), oily fish consumed (0–3.0, 3.1–7.5, 7.6–14.9, and 15–100 g/day); gestational age at blood draw (12–16, 17–18, 19–20, and 21–37 weeks); nulliparous or most recent inter-pregnancy interval (nulliparous, 4–23, 24–47, and 48–245 months); and breastfeeding duration in previous pregnancy (nulliparous or b1, 1–5, 6–11, 12–17, and 18–36months). Subjects were excluded from the analysis if they had missing values for any modeled covariates. Additionally, a sensitivity analysis was performed with the inclusion of weight gain (kg) from pre-pregnancy to mid-pregnancy as a continuous covariate, although this variable was not part of the original DAG.*' |
| Starling *et al.* 2017 | **Page 503:**  '*The following set of potential confounders was selected based on a directed acyclic graph generated from published literature (Supplementary Figure 2): maternal age (years), maternal pre-pregnancy BMI (kg m−2), smoking during pregnancy (any vs none), race/ethnicity (non-Hispanic white, non-Hispanic black, Hispanic, other), education completed (<12th grade, high school or GED completed, some college or associate’s degree, four year college, graduate degree), gravidity (any previous pregnancies vs none). Adjustment for predicted gestational weight gain to 39 weeks was included in secondary models. Additional adjustment for average second trimester BP was performed as a sensitivity analysis. Separate multivariable univariate linear regression models were constructed to estimate associations of second to third trimester changes in average systolic and diastolic BP with each of the three body composition variables: neonatal fat mass, fat-free mass and percent fat mass. All previously mentioned potential confounders, and additionally infant sex and gestational age at birth, were included in the adjusted model. Adjustment for predicted gestational weight gain to 39 weeks was included in secondary models. We conducted sensitivity analyses to explore the potential for an association between change in average maternal BP and offspring gestational age at birth. The association between second to third trimester change in maternal BP and gestational age at birth was explored by fitting univariate Cox proportional hazards models for the length of pregnancy (days), with second to third trimester change in systolic BP or diastolic BP as the predictor and with cesarean deliveries (n = 154) counted as censored.*' |
| Stratakis *et al.* 2016 | **Page 383:**  '*To select the confounders for adjustment in multivariable models, we used a directed acyclic graph approach based on prior knowledge about parental and child covariates that may be related to child adiposity and/or fish intake in pregnancy. We constructed the graph using DAGitty version 2.1 (DAGitty) to identify minimally sufficient adjustment sets of covariates and chose the set on which we had the best available information (eFigure 2 in the Supplement). We included the following variables in multivariable models: maternal education, maternal prepregnancy BMI, maternal smoking during pregnancy, maternal age at delivery, and birth weight.*' |
| Strutz *et al.* 2014 | **Page 91:**  '*We included the following measures as potential confounders from a hypothesized causal model generated with DAGitty software: Respondent’s age and parity (nulliparous/parous) at the time of the birth, nativity (foreign-born to foreign-born parent, native-born to foreignborn parent, or native-born to native-born parent), and two indicators of respondent’s childhood socioeconomic status from both the respondent and the parent interviews: Her mother’s educational attainment (less than high school, general educational development certificate (GED), high school diploma, some college or trade school, or completed college or more), and any household receipt of public assistance before age 18 (yes/no)… We then used multiple multinomial logistic regression to obtain adjusted odds ratios (ORs) with 95% confidence intervals (CIs) for LBW and macrosomia including the preconception health trajectories to evaluate whether inclusion of preconception indicators attenuated associations between race/ethnicity and birth weight.*'  **Page 91:**  '*We used additional multiple multinomial logistic regression models to examine direct effects of race/ethnicity and preconception indicators controlling for potential confounders and prenatal factors. We then re-estimated models including interaction terms between race/ethnicity and the preconception indicators to determine effect modification of preconception health trajectories on birth weight. To maximize statistical power given the small sample sizes for some racial/ethnic groups, we included potential confounders but not the prenatal factors in the latter models.*' |
| Sunol *et al.* 2015 | **Page 6:**  '*A directed acyclic graph (DAG) was used to depict our knowledge and assumptions about the (plausible) relations between predictors: hospital quality management measures, quality activities at the department level, and clinical practice indicators. Variable selection for the statistical models in this paper was guided by the DAG shown in Fig 1.*'  **Page 7:**  '*Our models were adjusted for confounding fixed effects at the country level (country), hospital level (number of beds, teaching status, ownership), and patient level (age, gender, education level), in addition to quality measures as dictated by the DAG in Fig 1.*' |
| Sunyer *et al.* 2015 | **Page 6:**  '*This model was further adjusted for potential confounders selected with directed acyclic graphs. Based on all socio-demographic and contextual covariables mentioned above, we used the program DAGitty 2.0, with a priori definition of the temporal direction of the events, to draw causal diagrams. The final adjusted model (model 2) included additional coefficients for sex, maternal education (less than/primary/secondary/university), residential neighborhood socioeconomic status, and air pollution exposure at home*' |
| Tamimi *et al.* 2017 | **Page 73:**  '*In order to reduce the possibility of confounding by myriad factor, three directed acyclic graphs (using the Dagitty 2.2 software) were used to identify the confounders requiring control for each of the three different study outcomes. The following set of confounders was identified for each outcome: age, diabetes, duration of AD, ischemic heart disease, renal disease, and use of ACEIs, diuretics, SSRI, PPI, statins, and hypnotics.*' |
| Tassiopoulos *et al.* 2017 | **Page 921:**  '*Potential confounders were evaluated in directed acyclic graphs (DAGs) using DAGitty to examine the causal relationships between variables and to identify a minimally sufficient subset to include in multivariable analyses.*'  **'***Potential confounders were added individually to the univariable model, and variables that changed the effect estimate of BPb by>10% were kept in the final multivariable model. We decided a priori to include maternal education, age at developmental assessment, and geographic region in all models*.' |
| Tedla & Bautista 2016 | **Page 773:**  '*We drew a priori a directed acyclic graph (DAG) and used Pearl’s back-door criterion, as implemented in DAGitty to select a minimal sufficient adjustment set of variables to control confounding by measured variables, while avoiding selection bias resulting from conditioning on a collider. The DAG (Figure 1) was built by identifying all known factors affecting side effects or adherence, and then including all common causes of any pair of variables already in the DAG. Pearl’s back-door criterion is based on the identification of noncausal (backdoor) paths connecting the exposure to the outcome. Noncausal paths must be blocked by adjustment/conditioning to obtain a valid association between the exposure and the outcome. The resulting minimal sufficient adjustment set included antihypertensive medication class, number of antihypertensive medications, comorbidity, and use of medications for other diseases as potential confounders. We adjusted for these variables as well as age, sex, and race in all our regression models.*' |
| Tedla *et al.* 2017 | **Page 862:**  '*We drew a directed acyclic graph, and applied Pearl’s backdoor criterion, using the program DAGitty (Johannes Textor, Theoretical Biology & Bioinformatics Group, University of Utrecht, The Netherlands), to identify potential confounders for the association between BP control and arterial stiffness. Potential confounders identified were age, sex, race, smoking, physical activity, alcohol intake, BMI, hypercholesterolemia, diabetes, GFR, BP drug class, lipid medication, and baseline arterial stiffness. Mean BP was not required as an adjustment factor because the exposure variable ‘BP control status’ was derived from mean BP (e.g. controlled BP was defined as systolic BP of <140 and diastolic BP of <90mmHg). Carotid distensibility coefficient and YEM were included in separate regression models. Number of visits with controlled BP during visits 1 to 4 was used as a relevant exposure for change in arterial stiffness between visits 1 and 5.*' |
| Tedla *et al.* 2017A | **Page 119:**  '*To identify potential confounders, we drew a priori directed acyclic graph and applied Pearl’s back-door criterion using DAGitty. Potential confounders identified were age, sex, race, smoking, physical activity, alcohol intake, BMI, hypercholesterolemia, diabetes mellitus, GFR, C-reactive protein, menopausal status, and baseline arterial stiffness. In addition, mean arterial pressure and cumulative BP were adjusted when the exposure was SD of SBP and cumulative BP when the exposure was CV of SBP. SD, VIM and CV of SBP across visits 1 to 5 were included in separate regression models.*' |
| Traeger *et al.* 2016 | **Page 2770:**  '*To identify the most important covariates to include in our model, we constructed a directed acyclic graph (DAG) according to Shrier et al. The DAG was constructed using free software available at www.dagitty.net. In accordance with our DAG (see Online Resource 1, Figs. 1–5), we adjusted for selected predisposing factors (age, gender, socioeconomic status, past history, cultural background), compensation status, and illness factors (pain, disability, comorbid illness), to minimize bias in our estimate.*'  '*The emotional distress variables were entered in the first block of the model. In the second block of the model, we adjusted for the following covariates (see Online Resource 1, Fig. 3): pain intensity (1–6 scale), disability (Roland–Morris Score or 0–10 scale), co-morbid illness (self-rated general health on a 1–5 scale), age, gender, socioeconomic status (Socio-Economic Indexes for Areas (SEIFA) score, based on postal code), past history of low back pain (y/n), compensation status (y/n), cultural background (born in Australia, y/n—measured in Study 2 only).*'  '*To select important confounders of the relationship between pain and disability (exposure) and healthcare use (outcome), we used the same DAG used in for the primary models (see Online Resource 1, Fig. 2). Covariates adjusted for in the moderation model (see Online Resource 1, Fig. 5) were age, gender, socioeconomic status [Socio-Economic Indexes for Areas (SEIFA) decile score, based on postal code], past history of low back pain (y/n), cultural background (born in Australia y/n—measured in Study 2 only), and pain intensity (1–6 scale).*' |
| Upson *et al.* 2013 | **Page 93:**  '*A directed acyclic graph (DAG), informed by prior studies on sources of phthalate exposure and risk factors for endometriosis, was used to identify variables necessary for adjustment in the logistic regression model (Supplemental Materials, Fig. 1). Based on the proposed DAG, we adjusted for natural logarithm-transformed urinary creatinine, age, and reference year.*' |
| Urquia *et al.* 2011 | **Page 3:**  '*We based our choice of covariates for confounder control based on a theoretical model assisted with the use of directed acyclic graphs (DAGs).*'  '*Variables for confounder control were maternal age (< 20 years, 20 to 34 years and 35 years and more), marital status (single, divorced, separated versus married or common-law), and immigrations status (Canadianborn and foreign-born). Socioeconomic position (SEP) was approximated by the Low income after-tax cut-off (LICO-AT) (Above LICO-AT, at or below LICO-AT and missing).*' |
| Valvi *et al.* 2015 | **Page 1023:**  '*We selected the covariates retained in the final models using a combined approach of directed acyclic graphs (DAGs) and change-in- estimate procedures. The initial DAGs included maternal determinants of phthalate metabolite concentrations in this population: country of origin, education, social class [coded based on occupation using the International Standard Classification of Occupations (ISCO)–88 system], prepregnancy BMI, smoking, frequency of organic food consumption, and use of bleach during pregnancy. Other covariates were included based on previous literature: maternal age at delivery, parity, gestational weight gain, gestational diabetes (self-reported; yes, no), maternal physical activity (in metabolic equivalents per hour per day), alcohol consumption (yes, no), maternal urine BPA concentrations during pregnancy, paternal BMI, birth weight, exclusive breastfeeding duration, and child’s dietary habits [i.e., fast-food (< once/week, ≥ once/week) or sugar-sweetened beverage (< once/month, once to four times/month, > four times/ month) consumptions and total caloric intake (kilocalories/ day)] and time spent watching TV or playing videogames (≤ 1 hr/week, > 1 hr/week) at ages 4 and 7 years. Child sex and exact age were included in all crude and adjusted statistical models. To evaluate whether the assumed relationships and the minimum adjustment sets provided by the DAGs are supported by our data, we conducted forward and backward 10% change-in-estimate procedures departing from the minimum adjustment sets following the methods suggested by Evans et al. (2012). The overall DAG of the assumed or known causal relationships between covariates included in the final models is shown in Supplemental Material, Figure S1.*' |
| Wang & Bautista 2015 | **Page 143:**  '*We used directed acyclic graphs (DAG) and Pearl’s back-door criterion, as implemented in DAGitty, to select a minimal sufficient adjustment set (MSAS) of variables that would allow the identification of an unconfounded effect of bilirubin on BP. The DAG (Figure 1) was built by identifying all known factors affecting bilirubin or BP, and then including all common causes of any pair of variables already in the DAG. Variables in the MSAS blocked all non-causal but not the causal pathway between bilirubin and BP, and included age, gender, race, abdominal obesity, alcohol use, education (≥high school) and serum creatinine, albumin and uric acid.*'  **Page 144:**  '*All variables in the MSAS and only those variables were included in our full models*'  **Page 145:**  '*We conducted sensitivity analyses to evaluate the robustness of our results to different assumptions and potential biases. A particularly strong assumption was that an unconfounded effect of bilirubin on BP could be estimated by adjusting only for those variables in the MSAS. To test this assumption we conducted a calibrated simulation based sensitivity analysis based on propensity score matching, as proposed by Ichino et al. and implemented by Nannicini. Briefly, we assessed the reasonableness of this assumption by comparing our estimate of the effect of the bilirubin with estimates that were further adjusted for simulated potential confounders (SPC) drawn from the distribution of observed variables (see Supplementary for details).*' |
| Waterhouse *et al.* 2016 | **Page 1473:**  '*We used directed acyclic graphs to guide selection of potential confounding factors to be included in adjusted models. For associations between surgeon or hospital volume and outcomes (excluding complications), we also fitted models with a continuous propensity score as the only adjustment variable. Propensity scores were estimated using logistic regression models that included age, sex, state and remoteness of residence, SES, smoking status, family history of cancer, Charlson comorbidity index, ECOG performance status, TNM stage, positive lymph nodes, tumour differentiation and site, pre-operative WBC count and albumin level, jaundice and weight loss at diagnosis, any stenting prior to resection surgery and hospital type. We also estimated a propensity score for chemotherapy treatment using the model above but with preoperative WBC and albumin level omitted and surgeon*' |
| Webb *et al.* 2015 | **Page 110:**  '*All models were adjusted for age at diagnosis. We also present HRs and 95% CIs adjusted for state of residence, BMI (in kg/m2; ,18.5, 18.5–24.9, 25–29.9, 30+, unknown), and smoking status at diagnosis (current smoker yes/no) because causal diagrams (directed acyclic graphs) constructed by using the DAGitty program v2.0 (www. dagitty.net) suggested that inclusion of these variables was sufficient to control for potential confounding by other variables related to serum 25(OH)D (season of blood collection, time spent outside, physical activity, and diet) and/or survival (histology, grade of disease, and residual disease after surgery).*'  '*Analyses were performed for the entire study group and also stratified by timing of blood collection (at diagnosis or after the end of treatment and before recurrence). Because the outcome for women with advanced disease at diagnosis and/or who do not respond to primary treatment is very poor and thus potentially less likely to be influenced by aspects of lifestyle, we conducted additional analyses stratified by these variables.*' |
| Wei *et al.* 2016 | **Page 89:**  '*To assess the conditional effects of variables on recent HIV testing, we fitted 3 separate multivariable logistic regression models, where inclusion of covariates was guided by Poundstone et al’s socioepidemiologic framework, literature review, and causal diagrams (Fig. 1)*'  **Page 89:**  '*The first model assessed sociodemographic correlates of HIV testing behavior, variables with a P value higher than 0.3 and did not have a priori research interest were excluded in subsequent models. Then a second model, restricted to those who reported ever having anal sex with a man, was fitted to assess the conditional effects of sexual behavioral risk factors associated with recent HIV testing. A third model was fitted to assess psychosocial factors associated with recent HIV testing. Before modeling, we multiplied and imputed the missing values (n = 188, 35%) on subjective norm toward HIV testing, otherwise complete-case analyses were used for all other variables and models… To assess whether depression would mediate the association between experiences of homophobia and recent HIV testing, and whether subjective norms would mediate the relationship between HIV stigma and recent HIV testing, we conducted causal mediation parametric analysis with a counterfactual framework, which is robust against more modelling misspecifications such as interaction and nonlinearity. Using the STATA command (paramed) developed by Valeri and VanderWeele, we estimated the natural direct effect (NDE) and the natural indirect effect (NIE) by fitting a logistic regression for HIV testing conditional on experiences of homophobia and a set of covariates including age, marital status, employment status, hukou, place of residence, and reported condomless receptive anal sex. We then fitted a linear regression model for depression conditional on experiences of homophobia and the same set of covariates. The same procedure was used for subjective norms and HIV stigma.*' |
| Weyde *et al.* 2017 | **Page 4:**  '*Covariates were selected using Directed Acyclic Graphs (DAG; see Figure S1). DAG is a tool used for covariate selection to minimize the magnitude of bias. The DAG was developed after an extensive literature review, documenting the covariates’ associations with exposure and outcome. Based on the DAG, a minimal adjustment set (the minimal selection of variables to be adjusted for in order to avoid a biased result) was suggested using dagitty.net. This minimal adjustment set included gross household income, season when the questionnaire was completed, and urbanity. An additional set of variables was considered important to include in the full model because of their well-established association with exposure and outcome, and because they were well measured (few missing values, registry based, etc.): mother’s education, ethnicity, siblings in child’s household, and type of residential building.*'  **Page 5:**  '*First, a crude model was fitted, including only road traffic noise, age, and gender. Second, a minimal adjustment set model was fitted, additionally including income, season, and urbanity. The full model included all covariates listed above.*' |
| Weyde *et al.* 2017A | **Page 4:**  '*Covariates were selected using Directed Acyclic Graphs. DAG is a tool used for covariate selection to minimize the magnitude of bias. Two different DAGs were developed after an extensive literature review, suggesting the covariates’ associations with exposure and outcome. One DAG was made for the analyses with pregnancy noise, and a second was made for the analyses with postnatal noise (see Additional files 1 and 2 in supplementary material). Based on the DAGs, minimal adjustment sets (the minimal selection of variables to be adjusted for in order to avoid a biased result) were suggested using the web based software program dagitty.net. The minimal adjustment set from the DAG for the postnatal sample included gross household income (continuous; postnatal sample: at age 8 years and five-year average) and urbanity (at age 8/five-year average; indicating proximity to city center). An additional set of covariates was considered important to include in the full model because of their well-established association with exposure and outcome, and because they were well measured (few missing values, registry based, etc.). This would increase the chances that potential confounders were included (it is unlikely that the inclusion of only two covariates will account for all confounding). The additional covariates included maternal education (more than 4 years of university/college; less than 4 years of university/college, but more than high school; maximum high school), ethnicity, maternal alcohol consumption during pregnancy (consumed alcohol once a week or more during either of the trimesters), maternal smoking during pregnancy (smoked sometimes or daily during the last trimester), prematurity (gestational length of <259 or > = 259 days) and birth weight (> = vs < 2500 g).*'  '*In addition, air pollution during pregnancy was included in the analyses on the postnatal sample. The minimal adjustment set from the DAG for the pregnancy sample included the same covariates as listed above, but covering the pregnancy period only. In both samples, inclusion of rail traffic noise and air pollution was limited to sensitivity analyses.*'  '*A crude model (containing road traffic noise, gender and age), a minimal adjustment set model (adding household income and urbanity), and a full model (further adding maternal education, ethnicity, maternal alcohol consumption and smoking during pregnancy, low birth weight and prematurity) were fitted.*' |
| Williams *et al.* 2016 | **Page 135:**  '*We identified potential confounders based on past literature and descriptive statistics from our cohort, using directed acyclic graphs (see Supplemental Figure 1, http:// links.lww.com/QAD/A803) [28–30].We considered the following maternal covariates to be potential confounders: age and race, prepregnancy BMI, chronic health conditions such as pregestational diabetes, HIV viral load andCD4þ cell counts early in pregnancy, and first trimester substance use (including alcohol, tobacco, and illicit drugs [22]). In addition, socioeconomic measures were evaluated including household income and caregiver education levels. We descriptively summarized pregnancy outcome [low birth weight (LBW,<2500 g), preterm birth (<37 weeks gestation) and mode of birth (vaginal or cesarean delivery)] by case status, but our primary analyses excluded these characteristics and also maternal health measures later in pregnancy, as they may be on the causal pathway given prior evidence of their association with maternal antiretrovirals.*'  '*Covariates identified a priori as potential confounders and with P<0.20 in unadjusted models for case status were included in initial multivariable models, and those with P<0.10 and/or which changed antiretroviral exposure estimates by more than 10% were retained in final adjusted models.*' |
| Wilson *et al.* 2016 | **Page 4:**  '*Directed Acyclic Graph (DAG) (Fig. 2) was created to help identify and adjust for potential confounders and minimize bias assessing individual predictors of vaccine initiation (1+ dose) and series completion (3 doses). An online tool, DAGitty http://www.dagitty.net), was used to produce potential DAGs and derive minimally sufficient adjustment sets for predictor variables. Univariate and multivariable logistic regression models were used to calculate odds ratios (ORs) to identify variables related to vaccine initiation and series completion*' |
| Windsor *et al.* 2016 | **Page 219:**  '*Smoking status was an a priori confounder, and age and sex were considered as a priori effect modifiers. Any variable which was related to both COPD exacerbations and stroke was included. Other variables were added sequentially. Variables taken forward to the full model were identified using a causal modeling approach, clinical knowledge, or they were identified from the univariable analysis, having been strongly associated*' |
| Yau *et al.* 2015 | **Page 418:**  '*Set of variables to control for confounding was selected using directed acyclic graph (DAG) analysis with DAGitty software (http://www.dagitty.net/). After assessing all potential confounders, one minimally sufficient subset was found—the number of suspects, the number of customers, and weapon use. The final multivariable models included this subset.*'  '*Previous research suggests that customer injury risk differs between retail and service businesses. Thus, we evaluated industry type as an effect measure modifier using a Wald test statistic to specifically evaluate the industry type by employee resistance interaction term. Industry type was not an effect measure modifier at α = 0.15 and therefore was not treated as an effect measure modifier in this study.*'  '*A sensitivity analysis was conducted to estimate the potential range of effects of employee resistance on customer injury risk by building two additional models. In the incidents where the number of customers was unknown, we ran models assuming that all incidents had (1) one or (2) multiple customers present.*' |
| Yin *et al.* 2014 | **Page e1106:**  '*Models were constructed by using published literature and hypothesis-driven a priori assumptions to guide statistical assumptions, then reduced by backward elimination using P < .15 and <10% change-in-estimate criteria. Models estimating baseline CD4% main effects were adjusted for age, gender, and race. Models estimating age main effects were adjusted for gender and race. Interactions between immunologic classification and age were assessed and retained in models if P < .15. Models were not adjusted for adherence or viral failure because both variables are on the causal pathway to CD4% recovery. Adjustment for adherence or viral failure would control for information unavailable to clinicians when making decisions about treatment initiation and would add bias to estimates. However, we evaluated viral failure to assess the degree to which immunologic outcomes may be explained by poor viral control. Viral failure was defined as (1) failure to suppress HIV RNA viral load to <400 copies/mL by week 24 of HAART or (2) after initial viral suppression, viral load >400 copies/mL for 2 consecutive measurements, the first occurring by week 192. Analyses used Cox models for testing associations and stratified proportional hazards models to estimate adjusted Kaplan-Meier failure probabilities. CD4% main effect estimates were adjusted for age, continent, and baseline viral load. Age main effect estimates were adjusted for continent.*' |
| Zhang *et al.* 2008 | **Page 1309:**  '*We estimated the relative risk (RR) and 95% confidence intervals (95% CI) for lung cancer associated with use of antibiotics compared with nonuse by means of conditional logistic regression models stratified for age, sex, and calendar year. Other potential risk factors, such as smoking status (current, past, or never smoker by index date), smoking cessation interventions, episodes of respiratory and urinary tract infection and other types of infection, history of chronic obstructive pulmonary disease (COPD), asthma, body mass index (weight in kilograms divided by height in meters squared), use of other drugs, alcohol intake, and indicators of health care utilization (e.g., frequency of visits to the general practitioner), were introduced into the multivariate models.*'  **Page 1310:**  '*Investigators can propose various hypothetical causal networks linking antibiotic use, cancer, and other relevant factors. Diagrams known as directed acyclic graphs can be used to represent those networks as shown in Figs. 2A to D. The diagrams link variables (nodes) by arrows (directed edges) that represent direct causal effects (protective or causative) of one variable on another. Directed acyclic graphs are acyclic because the arrows never point from a given variable to any other variable in its past (i.e., causes precede their effects) and, thus, one can never start from one variable and, following the direction of the arrows, end up at the same variable. The absence of an arrow between two variables indicates that the investigator believes that there is no direct effect (i.e., a causal effect not mediated through other variables in the directed acyclic graphs) of one variable on the other.*' |
| Zhang *et al.* 2010 | **Page 1250:**  '*We hypothesise that the total effect of maternal height or parity on perinatal mortality consists of a direct effect and an indirect effect (i.e. an effect mediated by SGA). However, estimates of direct and indirect effects from standard regression methods with adjustment for intermediate variables can be biased in the presence of an interaction between SGA and maternal short stature or primiparity. Marginal structural models, using inverse probability weighting for the exposure and intermediate variables, enables appropriate adjustment for confounding factors, and provides valid estimates of the controlled direct effects for both SGA and non-SGA infants. We tested interactions between maternal short stature or primiparity and SGA, and estimated total effects and effects independent of birthweight (controlled direct effects) for both SGA and non-SGA births using marginal structural Cox models. Weights were obtained by estimating two sets of weights, one for short stature or primiparity and one for SGA, by logistic regression. Weight models included infant sex, maternal age, maternal BMI, mother’s country of birth [Nordic (i.e. Denmark, Finland, Iceland, Norway and Sweden) versus non-Nordic], smoking, and maternal education (school years completed)*'  **'***We also estimated controlled direct effects of maternal height and parity on perinatal mortality, mediated by continuous sex-specific EFWGA z-scores in place of dichotomous SGA, using marginal structural Cox models including a quadratic term for z-scores.*' |

# **Supplementary Table S6.**

| Checklist of recommendations for studies using DAGs to identify confounders. | | | | |
| --- | --- | --- | --- | --- |
| **Section** | **Recommendation number** | **Recommendation details** | **Page** |  |
| **Introduction** | | | | |
|  | 1 | The focal relationship(s) and estimand(s) of interest are stated in the study aims |  |  |
| **Methods** | | | | |
|  | 2 | DAGs for all focal relationships and estimands of interest are provided |  |  |
|  | 3 | DAGs include all relevant variables, including those where direct measurements are unavailable |  |  |
|  | 4 | DAGs are arranged so that all constituent arcs flow in the same direction |  |  |
|  | 5 | Missing arcs have been carefully considered. Optionally, these are justified with theory and/or evidence. |  |  |
|  | 6 | DAG-implied adjustment set(s) for all estimand(s) of interest are clearly stated, including any unobserved confounders |  |  |
|  | 8a | Alternative adjustment set(s) are clearly described and justified |  |  |
|  | 8b | Optionally, the consistency of all DAGs with the observed data has been explored. Subsequently modified DAGs are reported separately |  |  |
| **Results** | | | | |
|  | 7a | Estimate(s) from unmodified DAG-implied adjustment set(s) - or the nearest approximation thereof - are provided |  |  |
|  | 7b | Optionally, the impact of unobserved confounders has been estimated, and bias-adjusted estimates are reported |  |  |
|  | 8c | Estimates from alternative adjustment set(s) are reported separately to those obtained from DAG-implied adjustment sets |  |  |
